# Supplementary material for: Identification of Trace Components in Sauce-Flavor Baijiu by High-Resolution Mass Spectrometry
Source: Molecules. 2023 Jan 28;28(3):1273. doi: 10.3390/molecules28031273 (PMC9920578; doi:10.3390/molecules28031273)
Supplement: Supplementary file 1 [file molecules-28-01273-s001.zip › molecules-2160660-supplementary.pdf]

Supporting information:

Identification of trace components in sauce-flavor Baijiu by high resolution mass spectrometry

Jinfeng Ge[1], Yulin Qi[1,2,*], Wenrui Yao[1], Daohe Yuan[1], Qiaozhuan Hu[1], Chao Ma[1], Dietrich A. Volmer[3], Cong-Qiang Liu[1,2]

[1] Institute of Surface-Earth System Science, School of Earth System Science, Tianjin University, Tianjin 300072, China

[2] Tianjin Key Laboratory of Earth Critical Zone Science and Sustainable Development in Bohai Rim, Tianjin University, Tianjin 300072, China

[3] Department of Chemistry, Humboldt-Universität zu Berlin, Berlin 12489, Germany

[*] Corresponding author: Yulin Qi (yulin.qi@tju.edu.cn)

Contents of this file:

Supplementary tables:

Table S1 Compounds contained in different sauce-flavor Baijiu measured by FT-ICR MS in combination with both ESI+ and ESI-

Table S1 Compounds contained in different sauce-flavor Baijiu measured by FT-ICR MS in combination with both ESI+ and ESI-

MT-08	MT-15	MT-18	LJ	IS	XJ
C41H73N	C41H77N	C41H77N	C41H77N	C41H77N	C41H77N
C21H19N	C41H73N	C41H73N	C41H73N	C12H15N	C41H73N
C22H21N	C21H19N	C18H11N	C16H13N	C13H17N	C41H69N
C18H11N	C22H21N	C19H13N	C18H20N2	C15H13N	C20H23N
C19H13N	C18H11N	C25H25N	C19H22N2	C16H15N	C41H65N
C32H25N	C19H13N	C32H25N	C27H36N2	C17H17N	C21H19N
C35H31N	C32H25N	C35H31N	C30H18N2	C18H19N	C22H21N
C37H35N	C35H31N	C47H45N	C22H18N2O	C16H13N	C19H13N
C45H39N	C37H27N	C38H23N	C35H74N2O10	C17H15N	C32H25N
C38H23N	C45H39N	C43H21N	C35H72N2O10	C18H17N	C10H16N2
C38H21N	C38H23N	C10H16N2	C23H20N2O10	C19H19N	C11H18N2
C43H21N	C47H41N	C11H18N2	C20H12N2O10S2	C18H15N	C12H20N2
C10H16N2	C45H35N	C12H20N2	C27H24N2O10S2	C19H17N	C13H22N2
C11H18N2	C43H21N	C13H22N2	C26H14N2O10S2	C22H21N	C7H10N2
C12H20N2	C10H16N2	C7H10N2	C32H20N2O10S2	C23H23N	C8H12N2
C13H22N2	C11H18N2	C8H12N2	C13H14N2O11S2	C25H25N	C50H72N2
C7H10N2	C12H20N2	C9H14N2	C15H16N2O11S2	C26H21N	C36H40N2
C8H12N2	C13H22N2	C31H24N2	C18H22N2O11S2	C28H23N	C41H50N2
C20H14N2	C7H10N2	C30H18N2	C32H28N2O11S2	C32H25N	C50H68N2
C39H44N2	C8H12N2	C38H32N2	C12H12N2O12S3	C28H13N	C35H36N2
C44H54N2	C30H18N2	C10H16N2O	C25H12N2O13	C29H15N	C37H40N2
C33H26N2	C10H16N2O	C8H12N2O	C25H10N2O13	C30H17N	C30H24N2
C38H36N2	C9H14N2O	C9H14N2O	C31H10N2O2	C34H23N	C35H34N2
C30H18N2	C22H18N2O	C9H12N2O	C22H40N2O2S	C44H37N	C30H18N2
C30H16N2	C29H14N2O	C22H18N2O	C10H14N2O2S	C37H21N	C40H38N2
C31H18N2	C30H16N2O	C35H34N2O	C14H18N2O2S	C39H19N	C38H32N2
C10H16N2O	C36H28N2O	C30H16N2O	C23H36N2O2S	C46H23N	C46H48N2
C8H12N2O	C34H16N2O	C36H22N2O	C10H18N2O2S2	C8H12N2	C49H54N2
C9H14N2O	C26H48N2O10	C34H14N2O	C11H20N2O2S2	C12H16N2	C37H26N2
C22H18N2O	C21H16N2O10	C38H20N2O	C12H22N2O2S2	C13H18N2	C10H16N2O
C22H16N2O	C23H20N2O10	C23H20N2O10	C25H48N2O2S2	C14H20N2	C13H22N2O
C22H14N2O	C26H14N2O10S2	C26H14N2O10S2	C26H50N2O2S2	C17H20N2	C9H14N2O
C36H32N2O	C40H20N2O10S2	C40H20N2O10S2	C9H16N2O2S2	C14H10N2	C22H18N2O
C34H28N2O	C13H14N2O11S2	C43H12N2O10S2	C21H36N2O2S2	C16H12N2	C34H28N2O
C37H32N2O	C15H16N2O11S2	C13H14N2O11S2	C22H38N2O2S2	C18H14N2	C39H36N2O
C33H24N2O	C18H22N2O11S2	C15H16N2O11S2	C23H40N2O2S2	C20H18N2	C33H24N2O
C36H30N2O	C21H22N2O11S2	C25H12N2O13	C17H26N2O2S2	C23H16N2	C35H28N2O
C29H14N2O	C13H14N2O11S3	C11H18N2O2	C22H36N2O2S2	C43H34N2	C36H30N2O
C30H16N2O	C15H32N2O13	C12H20N2O2	C17H22N2O2S2	C33H10N2	C30H16N2O
C36H28N2O	C28H52N2O13	C10H14N2O2	C10H18N2O2S3	C34H12N2	C41H38N2O

C36H22N2O	C15H24N2O13	C11H16N2O2	C12H22N2O2S3	C16H14N2O	C47H50N2O
C36H12N2O	C26H18N2O13	C12H18N2O2	C13H24N2O2S3	C17H16N2O	C49H54N2O
C21H16N2O10	C25H12N2O13	C13H16N2O2	C14H26N2O2S3	C17H10N2O	C36H28N2O
C23H20N2O10	C25H10N2O13	C15H20N2O2	C11H18N2O2S3	C22H18N2O	C31H16N2O
C20H12N2O10	C28H16N2O13	C22H14N2O2	C13H22N2O2S3	C19H10N2O	C35H18N2O
C16H36N2O10S	C12H20N2O2	C8H12N2O2S	C10H16N2O2S3	C20H10N2O	C37H22N2O
C17H30N2O10S	C49H94N2O2	C9H12N2O2S	C13H20N2O2S3	C14H24N2O10	C13H26N2O10
C20H30N2O10S	C11H16N2O2	C21H34N2O2S	C14H22N2O2S3	C15H26N2O10	C20H40N2O10
C29H10N2O10S	C12H18N2O2	C23H36N2O2S	C15H24N2O2S3	C16H28N2O10	C15H26N2O10
C30H12N2O10S	C15H20N2O2	C23H34N2O2S	C16H26N2O2S3	C16H26N2O10	C13H14N2O11S2
C13H14N2O11S2	C22H14N2O2	C20H20N2O2S	C17H28N2O2S3	C18H30N2O10	C15H16N2O11S2
C15H16N2O11S2	C21H34N2O2S	C10H18N2O2S2	C21H36N2O2S3	C23H40N2O10	C15H24N2O13
C18H22N2O11S2	C22H36N2O2S	C11H20N2O2S2	C9H12N2O2S3	C15H24N2O10	C25H12N2O13
C25H12N2O13	C23H34N2O2S	C12H22N2O2S2	C10H12N2O2S3	C20H28N2O10	C28H18N2O13
C11H18N2O2	C23H14N2O2S	C13H24N2O2S2	C21H34N2O2S3	C17H20N2O10	C17H12N2O18
C12H20N2O2	C13H28N2O2S2	C9H16N2O2S2	C24H40N2O2S3	C22H28N2O10	C20H18N2O18
C11H16N2O2	C13H26N2O2S2	C18H32N2O2S2	C13H14N2O2S3	C33H30N2O10	C32H10N2O18
C12H18N2O2	C10H18N2O2S2	C19H34N2O2S2	C21H18N2O2S3	C34H32N2O10	C11H18N2O2
C14H18N2O2	C11H20N2O2S2	C20H36N2O2S2	C23H20N2O2S3	C37H34N2O10	C22H36N2O2
C15H20N2O2	C12H22N2O2S2	C21H38N2O2S2	C21H40N2O3	C30H18N2O10	C30H38N2O2
C22H14N2O2	C13H24N2O2S2	C22H40N2O2S2	C44H68N2O3	C38H34N2O10	C22H14N2O2
C31H10N2O2	C19H36N2O2S2	C15H24N2O2S2	C23H14N2O3	C40H32N2O10	C42H34N2O2
C41H28N2O2	C9H16N2O2S2	C16H26N2O2S2	C21H36N2O3S	C10H18N2O10S	C36H18N2O2
C42H12N2O2	C18H32N2O2S2	C17H28N2O2S2	C21H34N2O3S	C33H14N2O10S	C8H12N2O2S
C21H34N2O2S	C19H34N2O2S2	C18H30N2O2S2	C13H16N2O3S	C15H26N2O10S3	C23H40N2O2S
C22H36N2O2S	C20H36N2O2S2	C19H32N2O2S2	C26H54N2O3S2	C17H30N2O10S3	C9H12N2O2S
C23H36N2O2S	C21H38N2O2S2	C20H34N2O2S2	C27H56N2O3S2	C16H18N2O10S3	C22H36N2O2S
C23H14N2O2S	C22H40N2O2S2	C21H36N2O2S2	C19H26N2O3S2	C11H22N2O11S	C23H38N2O2S
C16H36N2O2S2	C15H24N2O2S2	C22H38N2O2S2	C14H14N2O3S2	C12H22N2O11S	C23H36N2O2S
C13H28N2O2S2	C16H26N2O2S2	C23H40N2O2S2	C9H20N2O3S3	C16H28N2O11S	C23H34N2O2S
C10H18N2O2S2	C17H28N2O2S2	C17H26N2O2S2	C13H22N2O3S3	C18H20N2O11S	C18H18N2O2S
C11H20N2O2S2	C18H30N2O2S2	C20H32N2O2S2	C10H14N2O3S3	C18H30N2O12	C10H18N2O2S2
C12H22N2O2S2	C19H32N2O2S2	C20H30N2O2S2	C12H18N2O3S3	C23H28N2O12	C11H20N2O2S2
C13H24N2O2S2	C20H34N2O2S2	C17H22N2O2S2	C14H22N2O3S3	C26H20N2O12	C12H22N2O2S2
C14H26N2O2S2	C21H36N2O2S2	C18H24N2O2S2	C11H16N2O3S3	C36H10N2O13S3	C13H24N2O2S2
C19H36N2O2S2	C22H38N2O2S2	C19H26N2O2S2	C13H20N2O3S3	C44H86N2O2	C9H16N2O2S2
C9H16N2O2S2	C23H40N2O2S2	C20H28N2O2S2	C25H26N2O3S3	C11H18N2O2	C18H32N2O2S2
C13H22N2O2S2	C17H26N2O2S2	C22H32N2O2S2	C27H28N2O3S3	C9H14N2O2	C19H34N2O2S2
C14H24N2O2S2	C20H32N2O2S2	C18H22N2O2S2	C30H34N2O3S3	C12H18N2O2	C20H36N2O2S2
C18H32N2O2S2	C17H22N2O2S2	C19H24N2O2S2	C11H22N2O4	C12H12N2O2	C15H24N2O2S2
C19H34N2O2S2	C18H24N2O2S2	C22H30N2O2S2	C12H22N2O4	C13H14N2O2	C16H26N2O2S2
C20H36N2O2S2	C19H24N2O2S2	C34H16N2O2S2	C10H12N2O4	C14H16N2O2	C17H28N2O2S2
C21H38N2O2S2	C31H48N2O2S2	C12H24N2O2S3	C21H26N2O4	C20H24N2O2	C18H30N2O2S2
C22H40N2O2S2	C22H24N2O2S2	C10H18N2O2S3	C27H28N2O4	C26H10N2O2	C19H32N2O2S2

C16H26N2O2S2	C23H26N2O2S2	C11H20N2O2S3	C11H14N2O4S	C27H12N2O2	C20H34N2O2S2
C17H28N2O2S2	C23H24N2O2S2	C12H22N2O2S3	C21H34N2O4S	C32H16N2O2	C21H36N2O2S2
C18H30N2O2S2	C10H18N2O2S3	C13H24N2O2S3	C33H14N2O4S	C33H18N2O2	C22H38N2O2S2
C19H32N2O2S2	C11H20N2O2S3	C14H26N2O2S3	C42H16N2O4S	C36H14N2O2	C23H40N2O2S2
C20H34N2O2S2	C12H22N2O2S3	C16H30N2O2S3	C45H22N2O4S	C23H40N2O2S	C14H20N2O2S2
C21H36N2O2S2	C13H24N2O2S3	C9H16N2O2S3	C26H50N2O4S2	C23H38N2O2S	C15H22N2O2S2
C22H38N2O2S2	C14H26N2O2S3	C10H16N2O2S3	C19H28N2O4S2	C14H18N2O2S	C17H26N2O2S2
C23H40N2O2S2	C15H28N2O2S3	C11H18N2O2S3	C21H32N2O4S2	C18H26N2O2S	C19H30N2O2S2
C24H42N2O2S2	C16H30N2O2S3	C13H22N2O2S3	C18H34N2O4S3	C23H36N2O2S	C20H32N2O2S2
C25H44N2O2S2	C9H16N2O2S3	C14H24N2O2S3	C13H18N2O4S3	C16H16N2O2S	C21H34N2O2S2
C17H26N2O2S2	C10H16N2O2S3	C23H42N2O2S3	C24H22N2O4S3	C18H20N2O2S	C22H36N2O2S2
C20H32N2O2S2	C11H18N2O2S3	C12H20N2O2S3	C26H26N2O4S3	C19H18N2O2S	C23H38N2O2S2
C24H40N2O2S2	C12H20N2O2S3	C15H26N2O2S3	C12H26N2O5	C21H22N2O2S	C19H28N2O2S2
C23H36N2O2S2	C13H22N2O2S3	C16H28N2O2S3	C10H20N2O5	C21H10N2O2S	C20H30N2O2S2
C24H38N2O2S2	C14H24N2O2S3	C20H36N2O2S3	C34H32N2O5	C33H22N2O2S	C21H32N2O2S2
C25H40N2O2S2	C16H28N2O2S3	C26H48N2O2S3	C13H28N2O5S	C10H18N2O2S2	C22H34N2O2S2
C26H42N2O2S2	C17H30N2O2S3	C8H12N2O2S3	C5H10N2O5S	C11H20N2O2S2	C23H36N2O2S2
C17H22N2O2S2	C18H32N2O2S3	C9H14N2O2S3	C39H68N2O5S	C12H22N2O2S2	C19H26N2O2S2
C22H32N2O2S2	C24H44N2O2S3	C11H16N2O2S3	C28H16N2O5S	C9H16N2O2S2	C20H28N2O2S2
C23H34N2O2S2	C8H12N2O2S3	C12H18N2O2S3	C23H44N2O5S2	C14H24N2O2S2	C21H30N2O2S2
C24H36N2O2S2	C9H14N2O2S3	C13H20N2O2S3	C24H46N2O5S2	C15H26N2O2S2	C22H32N2O2S2
C26H40N2O2S2	C12H18N2O2S3	C14H22N2O2S3	C23H42N2O5S2	C9H14N2O2S2	C23H34N2O2S2
C27H42N2O2S2	C13H20N2O2S3	C15H24N2O2S3	C23H18N2O5S3	C11H16N2O2S2	C24H36N2O2S2
C28H44N2O2S2	C14H22N2O2S3	C16H26N2O2S3	C9H20N2O6	C13H20N2O2S2	C25H38N2O2S2
C21H28N2O2S2	C15H24N2O2S3	C10H14N2O2S3	C22H36N2O6	C15H24N2O2S2	C32H52N2O2S2
C23H32N2O2S2	C16H26N2O2S3	C17H28N2O2S3	C27H42N2O6	C16H26N2O2S2	C20H26N2O2S2
C24H34N2O2S2	C20H34N2O2S3	C18H30N2O2S3	C24H30N2O6	C17H28N2O2S2	C21H28N2O2S2
C26H38N2O2S2	C10H14N2O2S3	C19H32N2O2S3	C22H12N2O6	C18H30N2O2S2	C22H30N2O2S2
C18H20N2O2S2	C11H16N2O2S3	C21H36N2O2S3	C33H12N2O6	C19H32N2O2S2	C23H32N2O2S2
C21H26N2O2S2	C17H28N2O2S3	C25H44N2O2S3	C36H18N2O6	C20H34N2O2S2	C31H48N2O2S2
C22H28N2O2S2	C24H42N2O2S3	C8H10N2O2S3	C39H24N2O6	C22H38N2O2S2	C32H50N2O2S2
C23H30N2O2S2	C9H12N2O2S3	C9H12N2O2S3	C12H26N2O6S	C9H12N2O2S2	C32H48N2O2S2
C24H32N2O2S2	C14H20N2O2S3	C12H16N2O2S3	C13H28N2O6S	C17H26N2O2S2	C27H36N2O2S2
C25H34N2O2S2	C15H22N2O2S3	C13H18N2O2S3	C22H44N2O7	C20H32N2O2S2	C10H20N2O2S3
C25H30N2O2S2	C18H28N2O2S3	C14H20N2O2S3	C22H42N2O7	C21H34N2O2S2	C10H18N2O2S3
C25H26N2O2S2	C20H32N2O2S3	C15H22N2O2S3	C19H24N2O7	C22H36N2O2S2	C11H20N2O2S3
C10H18N2O2S3	C27H46N2O2S3	C10H12N2O2S3	C19H12N2O7	C17H22N2O2S2	C12H22N2O2S3
C11H20N2O2S3	C10H12N2O2S3	C11H14N2O2S3	C30H34N2O7	C17H18N2O2S2	C13H24N2O2S3
C12H22N2O2S3	C11H14N2O2S3	C16H24N2O2S3	C21H42N2O7S	C16H14N2O2S2	C14H26N2O2S3
C13H24N2O2S3	C13H18N2O2S3	C21H34N2O2S3	C33H62N2O7S	C29H36N2O2S2	C9H16N2O2S3
C14H26N2O2S3	C17H26N2O2S3	C9H10N2O2S3	C35H62N2O7S	C27H24N2O2S2	C10H16N2O2S3
C16H30N2O2S3	C9H10N2O2S3	C14H18N2O2S3	C39H14N2O7S	C29H28N2O2S2	C11H18N2O2S3
C9H16N2O2S3	C15H20N2O2S3	C15H20N2O2S3	C23H22N2O8	C27H14N2O2S2	C12H20N2O2S3
C10H16N2O2S3	C13H16N2O2S3	C11H12N2O2S3	C25H12N2O8	C10H18N2O2S3	C13H22N2O2S3

C11H18N2O2S3	C17H22N2O2S3	C12H14N2O2S3	C10H20N2O8S	C12H22N2O2S3	C15H26N2O2S3
C12H20N2O2S3	C13H14N2O2S3	C13H16N2O2S3	C11H22N2O8S	C14H26N2O2S3	C17H30N2O2S3
C13H22N2O2S3	C14H16N2O2S3	C17H22N2O2S3	C32H58N2O8S	C19H36N2O2S3	C19H34N2O2S3
C14H24N2O2S3	C15H18N2O2S3	C13H14N2O2S3	C30H14N2O8S	C15H24N2O2S3	C8H12N2O2S3
C17H30N2O2S3	C16H20N2O2S3	C15H18N2O2S3	C20H16N2O9	C17H28N2O2S3	C9H14N2O2S3
C15H26N2O2S3	C13H12N2O2S3	C13H12N2O2S3	C22H20N2O9	C9H12N2O2S3	C12H18N2O2S3
C16H28N2O2S3	C14H14N2O2S3	C14H14N2O2S3	C24H22N2O9	C26H22N2O2S3	C13H20N2O2S3
C18H32N2O2S3	C15H16N2O2S3	C16H18N2O2S3	C22H12N2O9	C10H20N2O3	C14H22N2O2S3
C19H34N2O2S3	C16H18N2O2S3	C17H18N2O2S3	C33H16N2O9	C11H22N2O3	C10H14N2O2S3
C20H36N2O2S3	C18H22N2O2S3	C18H20N2O2S3	C23H42N2OS	C12H24N2O3	C11H16N2O2S3
C21H38N2O2S3	C18H20N2O2S3	C20H24N2O2S3	C8H12N2OS	C14H28N2O3	C15H24N2O2S3
C8H12N2O2S3	C13H10N2O2S3	C16H14N2O2S3	C15H24N2OS	C6H12N2O3	C16H26N2O2S3
C9H14N2O2S3	C16H14N2O2S3	C25H28N2O2S3	C16H26N2OS	C9H18N2O3	C17H28N2O2S3
C12H18N2O2S3	C18H18N2O2S3	C26H30N2O2S3	C23H40N2OS	C8H16N2O3	C26H46N2O2S3
C13H20N2O2S3	C23H22N2O2S3	C25H22N2O2S3	C8H10N2OS	C10H18N2O3	C8H10N2O2S3
C14H22N2O2S3	C23H20N2O2S3	C27H26N2O2S3	C9H12N2OS	C11H20N2O3	C9H12N2O2S3
C15H24N2O2S3	C25H22N2O2S3	C13H22N2O3	C11H12N2OS	C8H14N2O3	C12H16N2O2S3
C10H14N2O2S3	C11H16N2O3	C11H16N2O3	C23H36N2OS	C11H18N2O3	C14H20N2O2S3
C11H16N2O2S3	C21H10N2O3	C12H14N2O3	C15H14N2OS	C22H38N2O3	C10H12N2O2S3
C16H26N2O2S3	C23H14N2O3	C21H10N2O3	C17H18N2OS	C11H16N2O3	C11H14N2O2S3
C17H28N2O2S3	C22H12N2O3	C23H14N2O3	C19H22N2OS	C11H14N2O3	C13H18N2O2S3
C18H30N2O2S3	C20H34N2O3S	C22H12N2O3	C20H24N2OS	C14H20N2O3	C15H22N2O2S3
C19H32N2O2S3	C21H36N2O3S	C36H28N2O3	C22H12N2OS	C15H22N2O3	C16H24N2O2S3
C20H34N2O2S3	C21H34N2O3S	C9H16N2O3S	C38H78N2OS2	C14H18N2O3	C17H26N2O2S3
C27H48N2O2S3	C34H12N2O3S	C21H36N2O3S	C12H20N2OS2	C13H14N2O3	C18H28N2O2S3
C8H10N2O2S3	C12H26N2O3S2	C21H34N2O3S	C14H24N2OS2	C15H12N2O3	C9H10N2O2S3
C9H12N2O2S3	C12H22N2O3S2	C13H16N2O3S	C15H26N2OS2	C16H14N2O3	C13H16N2O2S3
C12H16N2O2S3	C14H26N2O3S2	C21H32N2O3S	C17H30N2OS2	C25H26N2O3	C15H20N2O2S3
C13H18N2O2S3	C19H34N2O3S2	C34H12N2O3S	C19H34N2OS2	C30H32N2O3	C21H32N2O2S3
C14H20N2O2S3	C20H36N2O3S2	C12H26N2O3S2	C21H38N2OS2	C29H28N2O3	C24H38N2O2S3
C15H22N2O2S3	C18H30N2O3S2	C12H24N2O3S2	C12H18N2OS2	C23H14N2O3	C13H14N2O2S3
C10H12N2O2S3	C19H32N2O3S2	C13H26N2O3S2	C14H22N2OS2	C25H12N2O3	C14H16N2O2S3
C11H14N2O2S3	C20H34N2O3S2	C12H22N2O3S2	C15H24N2OS2	C45H50N2O3	C13H12N2O2S3
C16H24N2O2S3	C21H36N2O3S2	C14H26N2O3S2	C16H26N2OS2	C28H14N2O3	C14H14N2O2S3
C17H26N2O2S3	C22H38N2O3S2	C19H34N2O3S2	C17H28N2OS2	C28H12N2O3	C15H16N2O2S3
C18H28N2O2S3	C17H26N2O3S2	C20H36N2O3S2	C18H30N2OS2	C33H22N2O3	C16H14N2O2S3
C19H30N2O2S3	C22H36N2O3S2	C19H32N2O3S2	C19H32N2OS2	C32H10N2O3	C24H20N2O2S3
C20H32N2O2S3	C22H34N2O3S2	C20H34N2O3S2	C20H34N2OS2	C34H14N2O3	C23H16N2O2S3
C22H36N2O2S3	C19H26N2O3S2	C21H36N2O3S2	C21H36N2OS2	C37H20N2O3	C13H22N2O3
C25H42N2O2S3	C14H14N2O3S2	C22H38N2O3S2	C22H38N2OS2	C36H16N2O3	C12H14N2O3
C9H10N2O2S3	C16H18N2O3S2	C23H40N2O3S2	C24H42N2OS2	C37H18N2O3	C23H14N2O3
C14H18N2O2S3	C20H24N2O3S2	C17H26N2O3S2	C26H46N2OS2	C13H16N2O3S	C22H12N2O3
C15H20N2O2S3	C11H18N2O3S3	C18H28N2O3S2	C17H26N2OS2	C18H18N2O3S	C47H44N2O3
C11H12N2O2S3	C12H20N2O3S3	C19H30N2O3S2	C21H34N2OS2	C20H20N2O3S	C21H36N2O3S

C12H14N2O2S3	C13H22N2O3S3	C20H30N2O3S2	C22H36N2OS2	C38H30N2O3S	C21H34N2O3S
C13H16N2O2S3	C15H26N2O3S3	C19H26N2O3S2	C23H38N2OS2	C16H28N2O3S2	C13H16N2O3S
C18H26N2O2S3	C17H30N2O3S3	C20H28N2O3S2	C20H28N2OS2	C19H34N2O3S2	C34H12N2O3S
C21H32N2O2S3	C9H14N2O3S3	C14H14N2O3S2	C22H30N2OS2	C20H36N2O3S2	C12H26N2O3S2
C23H36N2O2S3	C12H18N2O3S3	C11H20N2O3S3	C28H40N2OS2	C16H26N2O3S2	C14H26N2O3S2
C16H20N2O2S3	C10H14N2O3S3	C12H20N2O3S3	C22H26N2OS2	C18H30N2O3S2	C18H32N2O3S2
C17H22N2O2S3	C11H16N2O3S3	C13H22N2O3S3	C23H28N2OS2	C20H34N2O3S2	C20H36N2O3S2
C13H14N2O2S3	C13H20N2O3S3	C15H26N2O3S3	C11H22N2OS3	C12H16N2O3S2	C19H32N2O3S2
C14H16N2O2S3	C14H22N2O3S3	C22H40N2O3S3	C12H24N2OS3	C13H18N2O3S2	C21H36N2O3S2
C15H18N2O2S3	C16H26N2O3S3	C9H14N2O3S3	C13H26N2OS3	C15H22N2O3S2	C22H38N2O3S2
C19H26N2O2S3	C23H40N2O3S3	C11H18N2O3S3	C14H28N2OS3	C16H24N2O3S2	C17H26N2O3S2
C13H12N2O2S3	C20H32N2O3S3	C11H16N2O3S3	C10H18N2OS3	C17H26N2O3S2	C18H28N2O3S2
C14H14N2O2S3	C12H16N2O3S3	C12H18N2O3S3	C11H20N2OS3	C18H28N2O3S2	C19H30N2O3S2
C15H16N2O2S3	C13H18N2O3S3	C14H22N2O3S3	C12H22N2OS3	C19H30N2O3S2	C18H26N2O3S2
C16H18N2O2S3	C14H20N2O3S3	C10H14N2O3S3	C13H24N2OS3	C20H32N2O3S2	C19H28N2O3S2
C15H14N2O2S3	C25H42N2O3S3	C13H20N2O3S3	C14H26N2OS3	C21H34N2O3S2	C20H30N2O3S2
C16H14N2O2S3	C14H18N2O3S3	C15H24N2O3S3	C15H28N2OS3	C22H36N2O3S2	C19H26N2O3S2
C18H18N2O2S3	C21H32N2O3S3	C16H26N2O3S3	C9H16N2OS3	C11H12N2O3S2	C21H30N2O3S2
C26H30N2O2S3	C14H16N2O3S3	C17H28N2O3S3	C10H16N2OS3	C13H16N2O3S2	C14H14N2O3S2
C23H22N2O2S3	C15H18N2O3S3	C21H36N2O3S3	C12H20N2OS3	C19H26N2O3S2	C18H22N2O3S2
C29H30N2O2S3	C13H12N2O3S3	C22H38N2O3S3	C13H22N2OS3	C20H12N2O3S2	C18H20N2O3S2
C21H10N2O3	C15H16N2O3S3	C27H48N2O3S3	C14H24N2OS3	C27H16N2O3S2	C20H24N2O3S2
C23H14N2O3	C18H22N2O3S3	C9H12N2O3S3	C8H12N2OS3	C13H22N2O3S3	C12H20N2O3S3
C22H12N2O3	C16H16N2O3S3	C11H14N2O3S3	C11H18N2OS3	C15H26N2O3S3	C13H22N2O3S3
C20H34N2O3S	C17H18N2O3S3	C12H16N2O3S3	C15H26N2OS3	C9H14N2O3S3	C11H18N2O3S3
C21H36N2O3S	C18H20N2O3S3	C13H18N2O3S3	C16H28N2OS3	C14H22N2O3S3	C9H14N2O3S3
C22H38N2O3S	C17H16N2O3S3	C14H20N2O3S3	C18H32N2OS3	C10H14N2O3S3	C10H14N2O3S3
C20H32N2O3S	C24H24N2O3S3	C15H22N2O3S3	C21H38N2OS3	C12H18N2O3S3	C11H16N2O3S3
C21H34N2O3S	C25H26N2O3S3	C16H24N2O3S3	C25H46N2OS3	C10H20N2O4	C12H18N2O3S3
C22H36N2O3S	C26H28N2O3S3	C17H26N2O3S3	C26H48N2OS3	C11H22N2O4	C13H20N2O3S3
C13H16N2O3S	C26H26N2O3S3	C17H24N2O3S3	C9H14N2OS3	C8H16N2O4	C14H22N2O3S3
C22H34N2O3S	C21H12N2O3S3	C14H18N2O3S3	C14H22N2OS3	C9H18N2O4	C15H24N2O3S3
C34H12N2O3S	C7H14N2O4	C15H16N2O3S3	C10H14N2OS3	C7H14N2O4	C16H26N2O3S3
C10H22N2O3S2	C10H12N2O4	C16H18N2O3S3	C11H16N2OS3	C10H18N2O4	C17H28N2O3S3
C12H26N2O3S2	C11H14N2O4	C17H20N2O3S3	C13H20N2OS3	C11H20N2O4	C18H30N2O3S3
C13H28N2O3S2	C30H52N2O4	C16H16N2O3S3	C16H26N2OS3	C8H14N2O4	C9H12N2O3S3
C12H24N2O3S2	C32H50N2O4	C17H16N2O3S3	C19H32N2OS3	C9H16N2O4	C11H14N2O3S3
C14H26N2O3S2	C22H16N2O4	C24H24N2O3S3	C21H36N2OS3	C10H16N2O4	C12H16N2O3S3
C19H34N2O3S2	C23H18N2O4	C25H26N2O3S3	C8H10N2OS3	C11H18N2O4	C13H18N2O3S3
C20H36N2O3S2	C33H10N2O4	C26H28N2O3S3	C9H12N2OS3	C13H22N2O4	C14H20N2O3S3
C17H28N2O3S2	C34H12N2O4	C25H24N2O3S3	C14H18N2OS3	C9H14N2O4	C15H22N2O3S3
C19H32N2O3S2	C6H12N2O4S	C24H20N2O3S3	C15H20N2OS3	C12H20N2O4	C17H26N2O3S3
C20H34N2O3S2	C21H34N2O4S	C25H20N2O3S3	C12H12N2OS3	C14H24N2O4	C19H30N2O3S3
C21H36N2O3S2	C24H24N2O4S	C21H12N2O3S3	C17H22N2OS3	C7H10N2O4	C13H16N2O3S3

C22H38N2O3S2	C32H18N2O4S	C10H14N2O4	C19H26N2OS3	C8H12N2O4	C14H18N2O3S3
C17H26N2O3S2	C42H16N2O4S	C10H12N2O4	C13H12N2OS3	C10H14N2O4	C15H20N2O3S3
C19H30N2O3S2	C45H22N2O4S	C11H14N2O4	C21H18N2OS3	C12H18N2O4	C20H30N2O3S3
C42H76N2O3S2	C21H36N2O4S2	C17H16N2O4	C24H24N2OS3	C11H16N2O4	C21H32N2O3S3
C19H28N2O3S2	C12H18N2O4S3	C37H56N2O4	C21H16N2OS3	C13H20N2O4	C23H36N2O3S3
C20H30N2O3S2	C17H20N2O4S3	C12H26N2O4S2	C23H20N2OS3	C8H10N2O4	C15H16N2O3S3
C19H26N2O3S2	C17H18N2O4S3	C27H52N2O4S2	C25H24N2OS3	C9H12N2O4	C17H18N2O3S3
C20H28N2O3S2	C24H22N2O4S3	C11H18N2O4S2	C26H26N2OS3	C10H12N2O4	C17H16N2O3S3
C24H36N2O3S2	C25H24N2O4S3	C16H28N2O4S2	C8H17N3	C12H16N2O4	C18H18N2O3S3
C18H22N2O3S2	C10H20N2O5	C21H36N2O4S2	C25H33N3	C14H20N2O4	C24H24N2O3S3
C11H18N2O3S3	C9H18N2O5	C19H30N2O4S2	C17H13N3	C11H14N2O4	C25H26N2O3S3
C12H20N2O3S3	C13H22N2O5	C12H18N2O4S3	C29H35N3	C13H18N2O4	C27H30N2O3S3
C13H22N2O3S3	C30H54N2O5	C13H18N2O4S3	C31H39N3	C11H12N2O4	C8H16N2O4
C15H26N2O3S3	C8H18N2O5S	C15H22N2O4S3	C32H41N3	C14H18N2O4	C10H18N2O4
C23H42N2O3S3	C28H16N2O5S	C16H20N2O4S3	C33H43N3	C13H14N2O4	C10H16N2O4
C17H30N2O3S3	C13H28N2O5S2	C18H18N2O4S3	C25H25N3	C14H16N2O4	C11H18N2O4
C9H14N2O3S3	C14H30N2O5S2	C23H20N2O4S3	C29H33N3	C16H20N2O4	C10H14N2O4
C11H16N2O3S3	C15H16N2O5S3	C24H22N2O4S3	C31H37N3	C17H22N2O4	C13H20N2O4
C12H18N2O3S3	C22H22N2O5S3	C25H24N2O4S3	C33H41N3	C43H74N2O4	C10H12N2O4
C14H22N2O3S3	C23H18N2O5S3	C27H28N2O4S3	C29H31N3	C16H18N2O4	C11H14N2O4
C10H14N2O3S3	C46H18N2O5S3	C29H32N2O4S3	C33H39N3	C15H16N2O4	C11H12N2O4
C13H20N2O3S3	C8H18N2O6	C26H24N2O4S3	C27H25N3	C17H12N2O4	C14H16N2O4
C15H24N2O3S3	C9H20N2O6	C18H32N2O5	C38H25N3	C23H22N2O4	C17H16N2O4
C16H26N2O3S3	C9H18N2O6	C30H54N2O5	C7H15N3O	C27H28N2O4	C38H34N2O4
C17H28N2O3S3	C18H34N2O6	C10H22N2O5S	C39H67N3O	C23H16N2O4	C21H34N2O4S
C19H32N2O3S3	C9H12N2O6	C5H10N2O5S	C39H65N3O	C25H14N2O4	C42H16N2O4S
C20H34N2O3S3	C26H42N2O6	C13H28N2O5S2	C39H61N3O	C17H28N2O4S	C45H22N2O4S
C23H40N2O3S3	C27H42N2O6	C14H30N2O5S2	C39H57N3O	C18H20N2O4S	C26H52N2O4S2
C9H12N2O3S3	C22H12N2O6	C23H18N2O5S3	C25H25N3O	C22H28N2O4S	C27H54N2O4S2
C20H32N2O3S3	C33H12N2O6	C25H22N2O5S3	C26H27N3O	C25H12N2O4S	C16H28N2O4S2
C11H14N2O3S3	C36H18N2O6	C26H24N2O5S3	C27H29N3O	C29H14N2O4S	C19H32N2O4S2
C12H16N2O3S3	C39H24N2O6	C28H28N2O5S3	C29H33N3O	C13H22N2O4S2	C21H36N2O4S2
C13H18N2O3S3	C39H16N2O6	C25H20N2O5S3	C31H35N3O	C15H26N2O4S2	C21H34N2O4S2
C14H20N2O3S3	C41H14N2O6	C8H18N2O6	C40H41N3O	C17H30N2O4S2	C9H16N2O4S3
C15H22N2O3S3	C9H16N2O6S	C9H20N2O6	C16H29N3O10	C15H24N2O4S2	C12H18N2O4S3
C16H24N2O3S3	C34H16N2O6S	C9H18N2O6	C17H31N3O10	C16H26N2O4S2	C13H18N2O4S3
C17H26N2O3S3	C21H44N2O6S2	C18H34N2O6	C16H27N3O10	C17H28N2O4S2	C23H38N2O4S3
C18H28N2O3S3	C27H56N2O6S2	C9H16N2O6	C17H29N3O10	C18H30N2O4S2	C24H40N2O4S3
C19H30N2O3S3	C20H42N2O6S2	C9H12N2O6	C17H27N3O10	C19H32N2O4S2	C16H22N2O4S3
C23H38N2O3S3	C12H24N2O6S2	C16H16N2O6	C36H65N3O10	C20H34N2O4S2	C16H16N2O4S3
C25H42N2O3S3	C13H26N2O6S2	C22H12N2O6	C44H51N3O10	C21H36N2O4S2	C18H18N2O4S3
C12H14N2O3S3	C14H28N2O6S2	C33H12N2O6	C22H45N3O10S2	C17H26N2O4S2	C24H22N2O4S3
C13H16N2O3S3	C15H30N2O6S2	C36H18N2O6	C29H33N3O10S2	C19H30N2O4S2	C26H26N2O4S3
C14H18N2O3S3	C16H32N2O6S2	C39H24N2O6	C36H35N3O10S2	C20H32N2O4S2	C10H20N2O5

C15H20N2O3S3	C38H76N2O6S2	C13H28N2O6S2	C33H13N3O10S2	C21H34N2O4S2	C10H18N2O5
C22H34N2O3S3	C13H24N2O6S2	C12H24N2O6S2	C15H23N3O11	C10H18N2O5	C11H18N2O5
C27H44N2O3S3	C15H28N2O6S2	C13H26N2O6S2	C21H21N3O11	C11H20N2O5	C12H20N2O5
C13H14N2O3S3	C29H54N2O6S2	C14H28N2O6S2	C38H53N3O11	C12H22N2O5	C13H22N2O5
C14H16N2O3S3	C31H58N2O6S2	C15H30N2O6S2	C30H37N3O11	C13H24N2O5	C14H24N2O5
C15H18N2O3S3	C17H20N2O6S2	C16H32N2O6S2	C36H43N3O11	C9H16N2O5	C15H26N2O5
C16H20N2O3S3	C22H12N2O6S2	C13H24N2O6S2	C32H15N3O11	C14H26N2O5	C16H28N2O5
C21H30N2O3S3	C37H20N2O6S2	C15H28N2O6S2	C20H31N3O11S	C7H12N2O5	C17H30N2O5
C13H12N2O3S3	C15H32N2O7	C24H44N2O6S2	C29H17N3O11S	C8H14N2O5	C8H12N2O5
C14H14N2O3S3	C8H18N2O7	C23H40N2O6S2	C16H35N3O12	C10H16N2O5	C11H16N2O5
C15H16N2O3S3	C7H12N2O7	C17H20N2O6S2	C16H31N3O12	C12H20N2O5	C14H20N2O5
C16H18N2O3S3	C8H14N2O7	C8H18N2O7	C28H55N3O12	C13H22N2O5	C14H16N2O5
C17H20N2O3S3	C9H16N2O7	C7H12N2O7	C30H55N3O12	C11H18N2O5	C16H20N2O5
C18H22N2O3S3	C19H24N2O7	C8H14N2O7	C30H51N3O12	C14H24N2O5	C10H22N2O5S
C16H16N2O3S3	C33H48N2O7	C9H16N2O7	C37H43N3O12	C15H26N2O5	C19H28N2O5S
C17H18N2O3S3	C22H10N2O7	C19H24N2O7	C42H47N3O12	C17H30N2O5	C23H46N2O5S2
C18H20N2O3S3	C30H16N2O7	C33H24N2O7	C35H13N3O12	C8H12N2O5	C25H50N2O5S2
C19H22N2O3S3	C34H12N2O7	C34H12N2O7	C33H53N3O13	C9H14N2O5	C26H52N2O5S2
C17H16N2O3S3	C36H16N2O7	C35H14N2O7	C29H43N3O13	C15H24N2O5	C27H54N2O5S2
C18H18N2O3S3	C37H18N2O7	C36H16N2O7	C36H57N3O13	C11H16N2O5	C29H58N2O5S2
C17H14N2O3S3	C10H20N2O7S	C37H18N2O7	C34H39N3O13	C12H18N2O5	C27H52N2O5S2
C18H16N2O3S3	C20H38N2O7S	C10H20N2O7S	C40H51N3O13	C13H20N2O5	C29H56N2O5S2
C19H18N2O3S3	C22H42N2O7S	C11H22N2O7S	C30H61N3O14	C14H22N2O5	C40H78N2O5S2
C24H24N2O3S3	C23H18N2O7S	C12H22N2O7S	C35H39N3O14	C16H26N2O5	C25H42N2O5S2
C25H26N2O3S3	C28H12N2O7S	C20H38N2O7S	C30H59N3O15	C9H12N2O5	C15H20N2O5S2
C26H26N2O3S3	C39H14N2O7S	C28H14N2O7S	C29H45N3O15	C11H14N2O5	C16H22N2O5S2
C27H28N2O3S3	C40H82N2O7S2	C12H24N2O7S2	C27H39N3O15	C12H16N2O5	C17H24N2O5S2
C28H30N2O3S3	C12H24N2O7S2	C12H22N2O7S2	C34H53N3O15	C13H18N2O5	C18H26N2O5S2
C10H12N2O4	C12H22N2O7S2	C13H24N2O7S2	C27H35N3O15	C14H20N2O5	C20H16N2O5S2
C11H14N2O4	C14H26N2O7S2	C14H26N2O7S2	C31H57N3O16	C9H10N2O5	C13H18N2O5S3
C32H50N2O4	C15H28N2O7S2	C15H28N2O7S2	C33H35N3O16	C14H18N2O5	C23H18N2O5S3
C17H16N2O4	C16H30N2O7S2	C16H30N2O7S2	C28H59N3O17	C11H12N2O5	C25H22N2O5S3
C23H18N2O4	C17H32N2O7S2	C17H32N2O7S2	C27H41N3O17	C13H16N2O5	C28H28N2O5S3
C30H14N2O4	C37H14N2O7S2	C14H24N2O7S2	C27H37N3O17	C13H14N2O5	C8H18N2O6
C36H68N2O4S	C15H32N2O8	C37H14N2O7S2	C21H11N3O17S2	C14H16N2O5	C9H20N2O6
C21H34N2O4S	C10H18N2O8	C19H24N2O8	C27H43N3O18	C16H20N2O5	C9H18N2O6
C21H30N2O4S	C19H24N2O8	C19H22N2O8	C25H33N3O19	C17H22N2O5	C18H34N2O6
C42H16N2O4S	C16H12N2O8	C25H12N2O8	C23H45N3O19S2	C18H24N2O5	C9H16N2O6
C45H22N2O4S	C11H22N2O8S	C10H20N2O8S	C11H13N3O2	C16H18N2O5	C12H20N2O6
C11H24N2O4S2	C28H16N2O8S	C15H28N2O8S2	C23H25N3O2	C17H20N2O5	C11H18N2O6
C12H26N2O4S2	C20H32N2O9	C14H24N2O8S2	C23H21N3O2	C16H16N2O5	C13H22N2O6
C26H54N2O4S2	C20H16N2O9	C13H14N2O8S2	C29H21N3O2	C10H16N2O5S	C14H24N2O6
C16H28N2O4S2	C22H20N2O9	C20H34N2O9	C39H25N3O2	C20H34N2O5S	C15H26N2O6
C19H32N2O4S2	C22H12N2O9	C20H32N2O9	C49H35N3O2	C15H20N2O5S	C9H12N2O6

C21H36N2O4S2	C19H34N2O9S	C19H14N2O9	C25H39N3O20	C16H22N2O5S	C10H14N2O6
C16H24N2O4S2	C19H32N2O9S	C20H16N2O9	C12H25N3O2S2	C23H24N2O5S	C11H16N2O6
C17H26N2O4S2	C23H42N2OS	C37H46N2O9	C14H29N3O2S2	C48H32N2O5S	C12H18N2O6
C12H18N2O4S3	C8H12N2OS	C30H10N2O9	C11H21N3O2S2	C19H32N2O5S2	C13H20N2O6
C14H22N2O4S3	C23H40N2OS	C33H16N2O9	C15H29N3O2S2	C21H36N2O5S2	C14H22N2O6
C15H24N2O4S3	C8H10N2OS	C25H52N2O9S	C16H31N3O2S2	C20H32N2O5S2	C15H24N2O6
C12H16N2O4S3	C9H12N2OS	C19H34N2O9S	C10H17N3O2S2	C22H36N2O5S2	C16H26N2O6
C13H18N2O4S3	C23H38N2OS	C20H36N2O9S	C8H13N3O2S2	C38H62N2O5S2	C12H16N2O6
C14H20N2O4S3	C23H36N2OS	C20H34N2O9S	C11H17N3O2S3	C25H32N2O5S2	C14H20N2O6
C15H22N2O4S3	C15H14N2OS	C33H24N2O9S	C23H37N3O2S3	C30H30N2O5S2	C16H24N2O6
C16H24N2O4S3	C17H18N2OS	C8H12N2OS	C43H65N3O2S3	C32H28N2O5S2	C26H42N2O6
C17H26N2O4S3	C19H22N2OS	C23H40N2OS	C14H29N3O3	C9H20N2O6	C27H42N2O6
C18H28N2O4S3	C20H24N2OS	C8H10N2OS	C36H19N3O3	C11H20N2O6	C17H22N2O6
C20H32N2O4S3	C21H26N2OS	C9H12N2OS	C40H11N3O3	C12H22N2O6	C24H30N2O6
C22H36N2O4S3	C22H28N2OS	C23H38N2OS	C12H17N3O3S	C8H14N2O6	C45H68N2O6
C23H38N2O4S3	C19H20N2OS	C15H14N2OS	C43H71N3O3S	C10H18N2O6	C33H12N2O6
C24H40N2O4S3	C21H24N2OS	C17H18N2OS	C40H13N3O3S	C11H18N2O6	C36H18N2O6
C26H44N2O4S3	C35H72N2OS2	C18H20N2OS	C11H23N3O3S2	C12H20N2O6	C21H44N2O6S2
C23H36N2O4S3	C10H20N2OS2	C19H22N2OS	C8H17N3O3S2	C13H22N2O6	C28H58N2O6S2
C13H16N2O4S3	C11H22N2OS2	C21H26N2OS	C10H19N3O3S2	C10H16N2O6	C38H78N2O6S2
C14H18N2O4S3	C9H18N2OS2	C19H20N2OS	C11H21N3O3S2	C14H24N2O6	C26H52N2O6S2
C16H22N2O4S3	C12H20N2OS2	C10H20N2OS2	C12H23N3O3S2	C15H26N2O6	C29H58N2O6S2
C17H24N2O4S3	C14H24N2OS2	C9H18N2OS2	C13H25N3O3S2	C8H12N2O6	C27H50N2O6S2
C18H26N2O4S3	C15H26N2OS2	C12H20N2OS2	C14H27N3O3S2	C9H14N2O6	C38H72N2O6S2
C26H42N2O4S3	C16H28N2OS2	C14H24N2OS2	C15H29N3O3S2	C12H18N2O6	C40H76N2O6S2
C16H20N2O4S3	C17H30N2OS2	C15H26N2OS2	C17H33N3O3S2	C14H22N2O6	C15H18N2O6S2
C17H22N2O4S3	C19H34N2OS2	C16H28N2OS2	C18H35N3O3S2	C10H14N2O6	C15H32N2O7
C20H28N2O4S3	C20H36N2OS2	C17H30N2OS2	C23H45N3O3S2	C11H16N2O6	C8H18N2O7
C15H16N2O4S3	C21H38N2OS2	C18H32N2OS2	C25H49N3O3S2	C13H20N2O6	C22H42N2O7
C16H18N2O4S3	C22H40N2OS2	C19H34N2OS2	C8H15N3O3S2	C15H24N2O6	C8H14N2O7
C19H24N2O4S3	C12H18N2OS2	C20H36N2OS2	C13H23N3O3S2	C16H26N2O6	C12H20N2O7
C16H16N2O4S3	C14H22N2OS2	C21H38N2OS2	C15H27N3O3S2	C8H10N2O6	C13H22N2O7
C17H18N2O4S3	C15H24N2OS2	C22H40N2OS2	C16H29N3O3S2	C9H12N2O6	C12H18N2O7
C19H22N2O4S3	C16H26N2OS2	C12H18N2OS2	C17H31N3O3S2	C10H12N2O6	C13H20N2O7
C17H16N2O4S3	C17H28N2OS2	C13H20N2OS2	C18H33N3O3S2	C11H14N2O6	C14H22N2O7
C24H26N2O4S3	C18H30N2OS2	C14H22N2OS2	C19H35N3O3S2	C12H16N2O6	C15H24N2O7
C22H20N2O4S3	C19H32N2OS2	C15H24N2OS2	C20H37N3O3S2	C14H20N2O6	C16H26N2O7
C23H20N2O4S3	C20H34N2OS2	C16H26N2OS2	C21H39N3O3S2	C15H22N2O6	C17H28N2O7
C24H22N2O4S3	C21H36N2OS2	C17H28N2OS2	C25H47N3O3S2	C16H24N2O6	C16H24N2O7
C26H24N2O4S3	C22H38N2OS2	C18H30N2OS2	C27H51N3O3S2	C13H16N2O6	C19H24N2O7
C10H22N2O5	C23H40N2OS2	C19H32N2OS2	C27H49N3O3S2	C14H18N2O6	C34H12N2O7
C9H20N2O5	C22H36N2OS2	C20H34N2OS2	C21H35N3O3S2	C15H20N2O6	C36H16N2O7
C10H20N2O5	C25H42N2OS2	C21H36N2OS2	C22H47N3O3S3	C16H22N2O6	C11H24N2O7S
C9H18N2O5	C18H26N2OS2	C22H38N2OS2	C18H19N3O3S3	C17H24N2O6	C9H20N2O7S

C30H52N2O5	C19H28N2OS2	C23H40N2OS2	C21H25N3O3S3	C14H16N2O6	C20H38N2O7S
C13H16N2O5	C31H52N2OS2	C15H22N2OS2	C27H15N3O3S3	C15H18N2O6	C22H42N2O7S
C24H12N2O5	C19H26N2OS2	C22H36N2OS2	C41H69N3O4	C16H20N2O6	C23H40N2O7S
C10H20N2O5S	C22H32N2OS2	C25H42N2OS2	C41H65N3O4	C17H22N2O6	C27H54N2O7S2
C32H60N2O5S2	C21H28N2OS2	C18H26N2OS2	C41H61N3O4	C16H18N2O6	C40H80N2O7S2
C33H62N2O5S2	C21H26N2OS2	C20H30N2OS2	C39H51N3O4	C17H20N2O6	C42H84N2O7S2
C28H50N2O5S2	C28H40N2OS2	C22H34N2OS2	C33H37N3O4	C19H24N2O6	C37H14N2O7S2
C16H24N2O5S2	C27H36N2OS2	C26H42N2OS2	C37H17N3O4	C24H30N2O6	C15H32N2O8
C22H36N2O5S2	C27H34N2OS2	C27H44N2OS2	C34H69N3O4S	C23H18N2O6	C19H24N2O8
C15H20N2O5S2	C24H26N2OS2	C28H46N2OS2	C32H55N3O4S	C28H26N2O6	C10H20N2O8S
C16H22N2O5S2	C26H30N2OS2	C19H26N2OS2	C32H53N3O4S	C24H16N2O6	C11H22N2O8S
C17H24N2O5S2	C25H24N2OS2	C22H32N2OS2	C42H27N3O4S	C31H26N2O6	C19H34N2O8S
C18H26N2O5S2	C10H20N2OS3	C26H40N2OS2	C10H19N3O4S2	C35H28N2O6	C38H68N2O8S
C19H28N2O5S2	C11H22N2OS3	C20H26N2OS2	C12H21N3O4S2	C33H12N2O6	C26H50N2O8S2
C20H30N2O5S2	C12H24N2OS3	C21H28N2OS2	C22H41N3O4S2	C35H16N2O6	C13H14N2O8S2
C16H20N2O5S2	C13H26N2OS3	C22H30N2OS2	C21H25N3O4S2	C40H14N2O6	C13H28N2O9
C18H24N2O5S2	C14H28N2OS3	C23H32N2OS2	C46H63N3O4S2	C23H40N2O6S2	C22H42N2O9
C20H16N2O5S2	C15H30N2OS3	C21H26N2OS2	C12H17N3O5	C20H32N2O6S2	C17H28N2O9
C16H22N2O5S3	C16H32N2OS3	C27H36N2OS2	C31H51N3O5	C11H22N2O7	C20H32N2O9
C19H28N2O5S3	C10H18N2OS3	C19H18N2OS2	C33H47N3O5	C12H24N2O7	C20H16N2O9
C21H32N2O5S3	C11H20N2OS3	C26H32N2OS2	C21H21N3O5	C10H16N2O7	C26H12N2O9
C18H24N2O5S3	C12H22N2OS3	C10H20N2OS3	C25H29N3O5	C12H20N2O7	C19H34N2O9S
C21H26N2O5S3	C13H24N2OS3	C11H22N2OS3	C25H27N3O5	C13H22N2O7	C19H32N2O9S
C22H28N2O5S3	C14H26N2OS3	C12H24N2OS3	C35H37N3O5	C14H24N2O7	C26H18N2O9S
C23H18N2O5S3	C15H28N2OS3	C13H26N2OS3	C30H21N3O5	C11H18N2O7	C8H12N2OS
C25H22N2O5S3	C16H30N2OS3	C14H28N2OS3	C45H37N3O5	C16H28N2O7	C9H14N2OS
C46H18N2O5S3	C17H32N2OS3	C15H30N2OS3	C21H47N3O5S	C15H24N2O7	C23H40N2OS
C8H18N2O6	C21H40N2OS3	C16H32N2OS3	C14H31N3O5S	C10H14N2O7	C8H10N2OS
C9H20N2O6	C9H16N2OS3	C10H18N2OS3	C34H69N3O5S	C11H16N2O7	C9H12N2OS
C19H38N2O6	C10H16N2OS3	C11H20N2OS3	C18H31N3O5S	C12H18N2O7	C23H38N2OS
C9H18N2O6	C12H20N2OS3	C12H22N2OS3	C26H43N3O5S	C13H20N2O7	C23H36N2OS
C14H26N2O6	C13H22N2OS3	C13H24N2OS3	C15H27N3O5S2	C14H22N2O7	C15H14N2OS
C9H16N2O6	C14H24N2OS3	C14H26N2OS3	C19H35N3O5S2	C16H26N2O7	C17H18N2OS
C9H12N2O6	C15H26N2OS3	C15H28N2OS3	C12H21N3O5S2	C9H12N2O7	C18H20N2OS
C26H42N2O6	C16H28N2OS3	C17H32N2OS3	C9H15N3O5S2	C11H14N2O7	C19H20N2OS
C15H12N2O6	C21H38N2OS3	C21H40N2OS3	C19H31N3O5S2	C12H16N2O7	C48H94N2OS2
C19H20N2O6	C11H18N2OS3	C9H16N2OS3	C20H43N3O5S3	C13H18N2O7	C12H20N2OS2
C24H30N2O6	C17H30N2OS3	C20H38N2OS3	C7H13N3O5S3	C14H20N2O7	C14H24N2OS2
C19H12N2O6	C18H32N2OS3	C10H16N2OS3	C19H31N3O6	C15H22N2O7	C15H26N2OS2
C25H12N2O6	C20H36N2OS3	C11H18N2OS3	C24H23N3O6	C16H24N2O7	C16H28N2OS2
C49H44N2O6	C8H12N2OS3	C12H20N2OS3	C27H15N3O6	C12H14N2O7	C17H30N2OS2
C33H12N2O6	C9H14N2OS3	C13H22N2OS3	C29H17N3O6	C13H16N2O7	C18H32N2OS2
C36H18N2O6	C11H16N2OS3	C14H24N2OS3	C27H55N3O6S	C14H18N2O7	C19H34N2OS2
C12H26N2O6S	C12H18N2OS3	C15H26N2OS3	C15H27N3O6S	C15H20N2O7	C20H36N2OS2

C13H28N2O6S	C13H20N2OS3	C16H28N2OS3	C30H39N3O6S	C13H14N2O7	C21H38N2OS2
C14H30N2O6S	C14H22N2OS3	C21H38N2OS3	C14H31N3O6S2	C15H18N2O7	C12H18N2OS2
C11H16N2O6S	C15H24N2OS3	C8H12N2OS3	C24H51N3O7	C17H22N2O7	C14H22N2OS2
C34H16N2O6S	C16H26N2OS3	C18H32N2OS3	C49H41N3O7	C18H24N2O7	C15H24N2OS2
C29H58N2O6S2	C19H32N2OS3	C20H36N2OS3	C14H29N3O7S	C16H18N2O7	C16H26N2OS2
C28H52N2O6S2	C20H34N2OS3	C23H42N2OS3	C24H39N3O7S	C17H20N2O7	C17H28N2OS2
C29H54N2O6S2	C10H14N2OS3	C26H48N2OS3	C24H51N3O8	C19H24N2O7	C18H30N2OS2
C31H58N2O6S2	C17H28N2OS3	C27H50N2OS3	C24H49N3O8	C17H18N2O7	C19H32N2OS2
C32H60N2O6S2	C18H30N2OS3	C9H14N2OS3	C24H47N3O8	C19H22N2O7	C20H34N2OS2
C34H64N2O6S2	C23H40N2OS3	C10H14N2OS3	C46H75N3O8	C27H12N2O7	C21H36N2OS2
C24H42N2O6S2	C27H48N2OS3	C11H16N2OS3	C33H53N3O8S	C8H14N2O7S	C22H38N2OS2
C31H56N2O6S2	C8H10N2OS3	C12H18N2OS3	C38H41N3O8S	C17H24N2O7S	C23H40N2OS2
C15H20N2O6S2	C9H12N2OS3	C13H20N2OS3	C30H21N3O8S	C33H24N2O7S	C24H42N2OS2
C18H26N2O6S2	C12H16N2OS3	C14H22N2OS3	C29H19N3O8S2	C32H20N2O7S	C14H20N2OS2
C15H18N2O6S2	C14H20N2OS3	C15H24N2OS3	C39H13N3O8S2	C18H30N2O7S2	C15H22N2OS2
C16H20N2O6S2	C15H22N2OS3	C16H26N2OS3	C11H23N3O9	C19H32N2O7S2	C17H26N2OS2
C17H22N2O6S2	C26H44N2OS3	C17H28N2OS3	C12H21N3O9	C15H22N2O7S2	C20H32N2OS2
C18H24N2O6S2	C10H12N2OS3	C18H30N2OS3	C19H33N3O9	C19H30N2O7S2	C21H34N2OS2
C19H26N2O6S2	C11H14N2OS3	C19H32N2OS3	C45H67N3O9	C20H32N2O7S2	C22H36N2OS2
C20H28N2O6S2	C9H10N2OS3	C20H34N2OS3	C40H39N3O9	C33H34N2O7S2	C23H38N2OS2
C21H30N2O6S2	C14H18N2OS3	C21H36N2OS3	C31H67N3OS2	C40H30N2O7S2	C24H40N2OS2
C17H36N2O7	C15H20N2OS3	C23H40N2OS3	C35H71N3OS2	C10H18N2O8	C25H42N2OS2
C8H18N2O7	C16H22N2OS3	C8H10N2OS3	C7H13N3OS2	C12H22N2O8	C15H20N2OS2
C7H14N2O7	C17H24N2OS3	C9H12N2OS3	C11H19N3OS2	C15H26N2O8	C18H26N2OS2
C8H14N2O7	C14H16N2OS3	C12H16N2OS3	C21H39N3OS2	C12H20N2O8	C20H30N2OS2
C20H34N2O7	C15H18N2OS3	C13H18N2OS3	C35H67N3OS2	C13H22N2O8	C21H32N2OS2
C19H24N2O7	C16H20N2OS3	C14H20N2OS3	C9H15N3OS2	C12H18N2O8	C22H34N2OS2
C20H26N2O7	C12H12N2OS3	C15H22N2OS3	C16H27N3OS2	C13H20N2O8	C23H36N2OS2
C18H18N2O7	C13H14N2OS3	C10H12N2OS3	C21H37N3OS2	C14H22N2O8	C25H40N2OS2
C34H12N2O7	C16H18N2OS3	C11H14N2OS3	C23H41N3OS2	C15H24N2O8	C26H42N2OS2
C35H14N2O7	C12H11N3	C9H10N2OS3	C23H39N3OS2	C16H26N2O8	C27H44N2OS2
C36H16N2O7	C25H33N3	C14H18N2OS3	C24H41N3OS2	C12H16N2O8	C28H46N2OS2
C10H20N2O7S	C17H13N3	C15H20N2OS3	C26H45N3OS2	C13H18N2O8	C16H20N2OS2
C28H12N2O7S	C39H57N3	C17H24N2OS3	C22H17N3OS3	C16H24N2O8	C17H22N2OS2
C39H14N2O7S	C31H39N3	C12H12N2OS3	C44H51N3OS3	C15H20N2O8	C18H24N2OS2
C15H20N2O7S2	C31H37N3	C14H16N2OS3	C50H67NO	C16H20N2O8	C19H26N2OS2
C16H18N2O7S2	C31H33N3	C15H18N2OS3	C50H63NO	C19H26N2O8	C20H28N2OS2
C17H20N2O7S2	C41H49N3	C16H20N2OS3	C29H17NO	C18H20N2O8	C21H30N2OS2
C37H14N2O7S2	C36H37N3	C13H14N2OS3	C28H53NO10	C25H20N2O8	C22H32N2OS2
C40H20N2O7S2	C41H45N3	C12H11N3	C17H29NO10	C35H30N2O8	C23H34N2OS2
C14H30N2O7S3	C42H47N3	C25H33N3	C28H51NO10	C36H16N2O8	C27H42N2OS2
C8H14N2O7S3	C41H43N3	C17H13N3	C28H49NO10	C15H24N2O8S	C20H26N2OS2
C22H20N2O7S3	C27H13N3	C39H57N3	C17H25NO10	C15H24N2O8S2	C21H28N2OS2
C15H32N2O8	C33H23N3	C27H25N3	C25H39NO10	C17H28N2O8S2	C22H30N2OS2

C19H24N2O8	C41H33N3	C27H13N3	C28H45NO10	C19H32N2O8S2	C23H32N2OS2
C21H20N2O8	C41H29N3	C33H23N3	C14H17NO10	C14H26N2O9	C10H20N2OS3
C25H12N2O8	C7H15N3O	C41H33N3	C38H65NO10	C22H42N2O9	C11H22N2OS3
C36H16N2O8	C10H11N3O	C7H15N3O	C19H25NO10	C13H22N2O9	C12H24N2OS3
C10H20N2O8S	C22H29N3O	C10H13N3O	C29H45NO10	C14H24N2O9	C13H26N2OS3
C11H22N2O8S	C39H61N3O	C10H11N3O	C25H35NO10	C12H18N2O9	C14H28N2OS3
C27H42N2O8S	C22H25N3O	C22H25N3O	C38H45NO10	C13H20N2O9	C9H18N2OS3
C26H36N2O8S	C39H57N3O	C39H57N3O	C41H41NO10	C15H24N2O9	C10H18N2OS3
C31H22N2O8S	C25H27N3O	C25H25N3O	C47H43NO10	C16H26N2O9	C11H20N2OS3
C45H16N2O8S	C27H29N3O	C40H41N3O	C35H67NO10S	C17H28N2O9	C12H22N2OS3
C27H52N2O8S2	C29H33N3O	C38H31N3O	C32H33NO10S	C15H22N2O9	C13H24N2OS3
C15H28N2O8S2	C30H35N3O	C38H23N3O	C28H45NO10S3	C16H24N2O9	C14H26N2OS3
C13H14N2O8S2	C28H17N3O	C42H29N3O	C33H33NO11	C18H24N2O9	C15H28N2OS3
C20H32N2O9	C40H41N3O	C47H23N3O	C41H49NO11	C19H24N2O9	C9H16N2OS3
C20H30N2O9	C40H37N3O	C47H19N3O	C30H39NO11S3	C31H24N2O9	C10H16N2OS3
C19H14N2O9	C38H31N3O	C14H31N3O10	C23H35NO12	C36H18N2O9	C11H18N2OS3
C20H16N2O9	C38H23N3O	C10H21N3O10	C17H17NO12	C38H22N2O9	C12H20N2OS3
C22H20N2O9	C42H29N3O	C16H29N3O10	C26H37NO12S3	C13H22N2O9S	C13H22N2OS3
C33H36N2O9	C42H23N3O	C17H31N3O10	C21H17NO12S3	C22H16N2O9S	C14H24N2OS3
C26H20N2O9	C47H23N3O	C17H29N3O10	C30H49NO13S3	C21H36N2O9S2	C16H28N2OS3
C33H16N2O9	C47H19N3O	C11H17N3O10	C25H37NO14	C19H14N2O9S2	C15H26N2OS3
C47H42N2O9	C14H31N3O10	C16H25N3O10	C30H43NO14	C23H18N2O9S2	C17H30N2OS3
C19H34N2O9S	C10H21N3O10	C17H27N3O10	C38H47NO14	C23H40N2OS	C19H34N2OS3
C19H32N2O9S	C20H41N3O10	C16H23N3O10	C32H29NO14	C8H10N2OS	C27H50N2OS3
C31H16N2O9S	C18H35N3O10	C17H25N3O10	C30H19NO14	C9H12N2OS	C8H12N2OS3
C8H12N2OS	C16H29N3O10	C13H17N3O10	C36H29NO14	C23H36N2OS	C9H14N2OS3
C23H40N2OS	C17H31N3O10	C36H61N3O10	C41H23NO14	C13H12N2OS	C11H16N2OS3
C8H10N2OS	C17H29N3O10	C16H19N3O10	C28H25NO14S3	C15H14N2OS	C12H18N2OS3
C9H12N2OS	C39H73N3O10	C20H19N3O10	C40H49NO15	C17H18N2OS	C13H20N2OS3
C23H36N2OS	C16H25N3O10	C28H23N3O10	C28H39NO16	C19H22N2OS	C14H22N2OS3
C40H68N2OS	C17H27N3O10	C14H31N3O10S2	C31H45NO16	C19H20N2OS	C16H26N2OS3
C15H14N2OS	C36H65N3O10	C26H39N3O10S2	C28H35NO16	C16H12N2OS	C10H14N2OS3
C17H18N2OS	C13H17N3O10	C14H31N3O11	C26H19NO16	C24H16N2OS	C15H24N2OS3
C19H22N2OS	C16H21N3O10	C15H25N3O11	C36H15NO16	C35H20N2OS	C17H28N2OS3
C21H26N2OS	C36H61N3O10	C16H27N3O11	C17H33NO17	C12H20N2OS2	C19H32N2OS3
C19H20N2OS	C20H19N3O10	C16H25N3O11	C19H35NO17	C14H24N2OS2	C21H36N2OS3
C20H22N2OS	C30H35N3O10	C16H23N3O11	C32H37NO17	C17H30N2OS2	C27H48N2OS3
C22H14N2OS	C28H23N3O10	C21H21N3O11	C26H31NO18	C19H34N2OS2	C8H10N2OS3
C10H22N2OS2	C34H17N3O10	C27H31N3O11	C8H17NO2	C21H38N2OS2	C9H12N2OS3
C10H20N2OS2	C11H23N3O10S	C27H27N3O11	C11H21NO2	C12H18N2OS2	C12H16N2OS3
C11H22N2OS2	C23H39N3O10S	C29H31N3O11	C13H25NO2	C13H20N2OS2	C14H20N2OS3
C9H18N2OS2	C14H31N3O10S2	C20H31N3O11S	C22H43NO2	C14H22N2OS2	C10H12N2OS3
C12H20N2OS2	C26H39N3O10S2	C16H35N3O12	C6H11NO2	C15H24N2OS2	C11H14N2OS3
C14H24N2OS2	C26H35N3O10S2	C18H37N3O12	C13H23NO2	C16H26N2OS2	C15H22N2OS3

C15H26N2OS2	C29H33N3O10S2	C19H39N3O12	C33H61NO2	C17H28N2OS2	C14H18N2OS3
C16H28N2OS2	C20H39N3O11	C16H31N3O12	C14H21NO2	C18H30N2OS2	C15H20N2OS3
C17H30N2OS2	C15H25N3O11	C20H39N3O12	C11H13NO2	C19H32N2OS2	C17H24N2OS3
C18H32N2OS2	C16H27N3O11	C32H61N3O12	C12H15NO2	C20H34N2OS2	C14H16N2OS3
C19H34N2OS2	C16H25N3O11	C30H55N3O12	C13H17NO2	C21H36N2OS2	C12H12N2OS3
C20H36N2OS2	C16H23N3O11	C15H23N3O12	C13H15NO2	C15H22N2OS2	C15H18N2OS3
C21H38N2OS2	C21H21N3O11	C30H51N3O12	C35H53NO2	C20H32N2OS2	C23H20N2OS3
C22H40N2OS2	C27H31N3O11	C41H59N3O12	C18H17NO2	C16H22N2OS2	C25H24N2OS3
C23H42N2OS2	C27H27N3O11	C42H47N3O12	C18H11NO2	C20H30N2OS2	C34H38N2OS3
C24H44N2OS2	C38H35N3O11	C35H13N3O12	C42H59NO2	C21H32N2OS2	C12H11N3
C12H18N2OS2	C32H15N3O11	C17H35N3O13	C26H25NO2	C29H34N2OS2	C25H33N3
C14H22N2OS2	C20H31N3O11S	C13H15N3O13	C42H57NO2	C28H30N2OS2	C17H13N3
C15H24N2OS2	C12H13N3O11S	C34H39N3O13	C39H49NO2	C30H34N2OS2	C39H57N3
C16H26N2OS2	C25H47N3O11S2	C16H33N3O14	C39H47NO2	C13H26N2OS3	C30H37N3
C17H28N2OS2	C16H35N3O12	C28H43N3O14	C35H37NO2	C12H20N2OS3	C50H77N3
C18H30N2OS2	C18H37N3O12	C35H43N3O14	C43H39NO2	C13H22N2OS3	C25H25N3
C19H32N2OS2	C19H39N3O12	C35H39N3O14	C43H13NO2	C14H24N2OS3	C26H27N3
C20H34N2OS2	C16H31N3O12	C13H23N3O15	C16H35NO2S	C10H16N2OS3	C27H29N3
C21H36N2OS2	C30H55N3O12	C29H49N3O15	C16H33NO2S	C13H20N2OS3	C29H33N3
C22H38N2OS2	C15H23N3O12	C27H39N3O15	C39H77NO2S	C14H22N2OS3	C31H37N3
C23H40N2OS2	C30H51N3O12	C14H25N3O16	C9H13NO2S	C15H24N2OS3	C25H21N3
C24H42N2OS2	C30H47N3O12	C26H35N3O16	C18H21NO2S	C31H32N2OS3	C27H25N3
C25H44N2OS2	C41H59N3O12	C33H35N3O16	C15H29NO2S2	C17H13N3	C31H33N3
C15H22N2OS2	C42H47N3O12	C19H21N3O16S	C11H19NO2S2	C27H25N3	C25H15N3
C17H26N2OS2	C35H13N3O12	C28H59N3O17	C12H15NO2S2	C27H21N3	C25H13N3
C22H36N2OS2	C21H39N3O12S	C20H35N3O17	C27H31NO2S2	C27H11N3	C30H21N3
C25H42N2OS2	C29H17N3O12S	C27H41N3O17	C35H31NO2S2	C33H23N3	C33H23N3
C26H44N2OS2	C13H15N3O13	C27H37N3O17	C49H51NO2S2	C35H25N3	C7H15N3O
C27H46N2OS2	C34H39N3O13	C25H31N3O17	C50H43NO2S2	C41H19N3	C7H13N3O
C28H48N2OS2	C16H33N3O14	C33H43N3O17	C11H23NO2S3	C42H21N3	C10H11N3O
C31H54N2OS2	C14H27N3O14	C27H43N3O18	C18H37NO2S3	C7H15N3O	C11H11N3O
C18H26N2OS2	C28H47N3O14	C25H33N3O19	C32H47NO2S3	C37H29N3O	C39H67N3O
C24H38N2OS2	C28H43N3O14	C11H17N3O2	C41H39NO2S3	C42H25N3O	C39H65N3O
C26H42N2OS2	C35H43N3O14	C11H13N3O2	C41H35NO2S3	C42H23N3O	C39H61N3O
C27H44N2OS2	C35H39N3O14	C23H25N3O2	C12H23NO3	C47H23N3O	C23H23N3O
C22H32N2OS2	C30H59N3O15	C37H53N3O2	C9H17NO3	C44H15N3O	C24H25N3O
C24H36N2OS2	C13H23N3O15	C25H27N3O2	C10H19NO3	C50H27N3O	C26H29N3O
C25H38N2OS2	C29H49N3O15	C23H21N3O2	C11H21NO3	C47H19N3O	C27H31N3O
C26H40N2OS2	C27H39N3O15	C25H25N3O2	C14H27NO3	C10H21N3O10	C28H33N3O
C27H42N2OS2	C34H53N3O15	C23H17N3O2	C7H11NO3	C16H29N3O10	C25H25N3O
C20H26N2OS2	C27H35N3O15	C25H13N3O2	C9H15NO3	C17H31N3O10	C26H27N3O
C21H28N2OS2	C32H35N3O15	C36H35N3O2	C11H17NO3	C24H45N3O10	C28H31N3O
C22H30N2OS2	C14H25N3O16	C41H25N3O2	C12H17NO3	C16H27N3O10	C29H33N3O
C23H32N2OS2	C26H39N3O16	C41H21N3O2	C13H19NO3	C17H29N3O10	C24H21N3O

C26H38N2OS2	C33H35N3O16	C37H11N3O2	C10H13NO3	C16H25N3O10	C25H23N3O
C20H24N2OS2	C33H31N3O16	C44H23N3O2	C11H15NO3	C26H43N3O10	C26H25N3O
C21H26N2OS2	C31H35N3O16S	C49H29N3O2	C13H15NO3	C25H25N3O10	C28H29N3O
C23H30N2OS2	C28H59N3O17	C25H39N3O20	C14H13NO3	C11H17N3O11	C29H31N3O
C25H34N2OS2	C27H41N3O17	C25H35N3O20	C18H17NO3	C12H19N3O11	C24H19N3O
C17H16N2OS2	C33H43N3O17	C18H27N3O2S	C16H13NO3	C16H27N3O11	C28H27N3O
C23H28N2OS2	C27H43N3O18	C22H33N3O2S	C22H23NO3	C31H55N3O11	C27H23N3O
C19H18N2OS2	C25H33N3O19	C24H37N3O2S	C34H45NO3	C22H29N3O11	C40H41N3O
C24H28N2OS2	C23H23N3O19	C45H69N3O2S	C42H61NO3	C24H27N3O11	C38H31N3O
C22H20N2OS2	C36H71N3O2	C10H17N3O2S2	C26H25NO3	C37H39N3O11	C47H23N3O
C30H34N2OS2	C11H13N3O2	C8H13N3O2S2	C26H21NO3	C36H35N3O11	C14H31N3O10
C10H20N2OS3	C31H43N3O2	C10H15N3O2S2	C24H15NO3	C38H35N3O11	C26H55N3O10
C11H22N2OS3	C23H25N3O2	C24H37N3O2S2	C34H35NO3	C32H13N3O11	C10H21N3O10
C12H24N2OS3	C25H29N3O2	C28H41N3O2S2	C8H17NO3S	C20H31N3O11S	C20H41N3O10
C13H26N2OS3	C24H25N3O2	C24H31N3O2S2	C20H39NO3S	C15H13N3O11S	C16H31N3O10
C14H28N2OS3	C25H27N3O2	C11H17N3O2S3	C17H17NO3S	C29H19N3O11S	C16H29N3O10
C15H30N2OS3	C23H21N3O2	C20H13N3O2S3	C19H21NO3S	C36H49N3O11S2	C17H31N3O10
C16H32N2OS3	C25H25N3O2	C21H15N3O2S3	C25H33NO3S	C34H39N3O11S2	C16H27N3O10
C10H18N2OS3	C26H27N3O2	C22H17N3O2S3	C27H37NO3S	C22H31N3O12	C17H29N3O10
C11H20N2OS3	C31H37N3O2	C13H27N3O3	C41H65NO3S	C18H19N3O12	C39H73N3O10
C12H22N2OS3	C23H17N3O2	C41H63N3O3	C16H13NO3S	C34H41N3O12	C16H25N3O10
C13H24N2OS3	C27H25N3O2	C22H23N3O3	C27H29NO3S	C43H15N3O12	C17H27N3O10
C14H26N2OS3	C34H33N3O2	C23H25N3O3	C30H57NO3S2	C19H31N3O12S	C36H65N3O10
C15H28N2OS3	C27H17N3O2	C27H33N3O3	C30H53NO3S2	C20H33N3O12S	C28H49N3O10
C17H32N2OS3	C36H35N3O2	C28H35N3O3	C40H73NO3S2	C10H19N3O12S2	C17H25N3O10
C21H40N2OS3	C39H25N3O2	C23H23N3O3	C26H43NO3S2	C21H25N3O12S2	C23H37N3O10
C9H16N2OS3	C41H21N3O2	C24H25N3O3	C26H41NO3S2	C13H27N3O13	C13H17N3O10
C10H16N2OS3	C37H11N3O2	C25H27N3O3	C26H39NO3S2	C11H17N3O13	C36H61N3O10
C12H20N2OS3	C25H39N3O20	C26H29N3O3	C19H37NO3S3	C39H61N3O13	C20H19N3O10
C13H22N2OS3	C18H27N3O2S	C20H17N3O3	C13H23NO3S3	C23H27N3O13	C41H59N3O10
C14H24N2OS3	C45H69N3O2S	C26H13N3O3	C23H33NO3S3	C34H39N3O13	C11H23N3O10S
C15H26N2OS3	C26H29N3O2S	C40H11N3O3	C18H23NO3S3	C41H35N3O13	C23H39N3O10S
C16H28N2OS3	C10H17N3O2S2	C16H31N3O3S	C26H23NO3S3	C42H33N3O13	C31H53N3O10S
C8H12N2OS3	C8H13N3O2S2	C40H13N3O3S	C22H43NO4	C16H33N3O14	C20H39N3O11
C11H18N2OS3	C10H15N3O2S2	C12H27N3O3S2	C31H61NO4	C17H35N3O14	C15H25N3O11
C17H30N2OS3	C38H65N3O2S2	C10H21N3O3S2	C10H17NO4	C18H35N3O14	C16H27N3O11
C18H32N2OS3	C48H85N3O2S2	C11H23N3O3S2	C11H19NO4	C14H23N3O14	C16H25N3O11
C19H34N2OS3	C28H41N3O2S2	C15H31N3O3S2	C12H21NO4	C15H25N3O14	C21H21N3O11
C21H38N2OS3	C24H31N3O2S2	C10H19N3O3S2	C13H23NO4	C16H27N3O14	C32H15N3O11
C9H14N2OS3	C24H29N3O2S2	C11H21N3O3S2	C14H25NO4	C20H27N3O14	C20H31N3O11S
C11H16N2OS3	C28H37N3O2S2	C13H25N3O3S2	C9H15NO4	C28H33N3O14	C12H13N3O11S
C12H18N2OS3	C32H35N3O2S2	C14H27N3O3S2	C7H11NO4	C32H37N3O14	C16H35N3O12
C13H20N2OS3	C23H39N3O2S3	C8H15N3O3S2	C8H13NO4	C34H33N3O14	C32H65N3O12
C14H22N2OS3	C46H71N3O2S3	C14H25N3O3S2	C10H15NO4	C36H33N3O14	C16H31N3O12

C15H24N2OS3	C21H15N3O2S3	C21H39N3O3S2	C11H17NO4	C28H35N3O14S	C38H75N3O12
C16H26N2OS3	C13H27N3O3	C21H35N3O3S2	C13H21NO4	C16H31N3O15	C30H55N3O12
C10H14N2OS3	C41H63N3O3	C15H17N3O3S2	C11H15NO4	C29H49N3O15	C15H23N3O12
C17H28N2OS3	C24H27N3O3	C22H33N3O3S3	C10H13NO4	C32H39N3O15	C32H57N3O12
C18H30N2OS3	C26H31N3O3	C18H19N3O3S3	C13H19NO4	C35H33N3O15	C30H51N3O12
C19H32N2OS3	C27H33N3O3	C21H25N3O3S3	C14H21NO4	C39H37N3O15	C41H59N3O12
C20H34N2OS3	C28H35N3O3	C20H11N3O3S3	C11H13NO4	C17H37N3O16	C32H39N3O12
C21H36N2OS3	C23H23N3O3	C22H15N3O3S3	C12H15NO4	C17H27N3O16	C42H47N3O12
C25H44N2OS3	C24H25N3O3	C23H17N3O3S3	C14H19NO4	C22H37N3O16	C32H65N3O13
C8H10N2OS3	C26H29N3O3	C41H65N3O4	C13H15NO4	C25H35N3O16	C34H59N3O13
C9H12N2OS3	C27H31N3O3	C21H21N3O4	C37H61NO4	C34H43N3O16	C13H15N3O13
C12H16N2OS3	C32H35N3O3	C29H37N3O4	C25H33NO4	C37H35N3O16	C34H39N3O13
C13H18N2OS3	C26H13N3O3	C25H27N3O4	C24H31NO4	C22H37N3O17	C29H25N3O13
C14H20N2OS3	C40H11N3O3	C26H29N3O4	C23H19NO4	C34H57N3O17	C16H33N3O14
C10H12N2OS3	C16H31N3O3S	C27H31N3O4	C32H29NO4	C20H25N3O17	C30H61N3O14
C11H14N2OS3	C12H17N3O3S	C28H33N3O4	C41H39NO4	C25H35N3O17	C32H65N3O14
C15H22N2OS3	C43H71N3O3S	C21H17N3O4	C36H15NO4	C29H39N3O17S	C38H73N3O14
C17H26N2OS3	C40H13N3O3S	C37H17N3O4	C24H45NO4S	C30H35N3O17S	C24H37N3O14
C18H28N2OS3	C10H21N3O3S2	C10H19N3O4S2	C48H93NO4S	C35H31N3O17S	C28H43N3O14
C21H34N2OS3	C11H23N3O3S2	C13H25N3O4S2	C20H35NO4S	C21H43N3O17S2	C30H59N3O15
C9H10N2OS3	C8H17N3O3S2	C16H29N3O4S2	C22H39NO4S	C24H39N3O18	C13H23N3O15
C14H18N2OS3	C10H19N3O3S2	C11H17N3O4S2	C13H19NO4S	C19H25N3O18	C32H61N3O15
C15H20N2OS3	C11H21N3O3S2	C15H25N3O4S2	C18H21NO4S	C20H29N3O19	C27H39N3O15
C16H22N2OS3	C12H23N3O3S2	C16H27N3O4S2	C25H33NO4S	C25H37N3O19	C27H35N3O15
C17H24N2OS3	C13H25N3O3S2	C13H19N3O4S2	C25H31NO4S	C27H37N3O19	C30H49N3O16
C12H14N2OS3	C14H27N3O3S2	C17H25N3O4S2	C43H67NO4S	C30H37N3O19	C26H35N3O16
C13H16N2OS3	C15H29N3O3S2	C14H13N3O4S2	C38H51NO4S	C43H85N3O2	C33H35N3O16
C22H34N2OS3	C25H49N3O3S2	C30H35N3O4S2	C26H21NO4S	C18H17N3O2	C28H59N3O17
C12H12N2OS3	C8H15N3O3S2	C21H35N3O4S3	C10H21NO4S2	C23H25N3O2	C25H39N3O17
C14H16N2OS3	C14H25N3O3S2	C19H19N3O4S3	C15H15NO4S2	C25H25N3O2	C27H41N3O17
C15H18N2OS3	C21H39N3O3S2	C21H23N3O4S3	C29H37NO4S2	C30H25N3O2	C25H31N3O17
C16H20N2OS3	C21H35N3O3S2	C20H13N3O4S3	C25H23NO4S2	C29H13N3O2	C33H43N3O17
C13H14N2OS3	C35H63N3O3S2	C23H15N3O4S3	C18H19NO4S3	C30H15N3O2	C18H27N3O19
C18H24N2OS3	C28H47N3O3S2	C40H21N3O4S3	C40H43NO4S3	C32H19N3O2	C26H43N3O19
C20H28N2OS3	C12H27N3O3S3	C21H25N3O5	C26H15NO4S3	C47H25N3O2	C18H21N3O19
C16H18N2OS3	C22H33N3O3S3	C33H47N3O5	C35H21NO4S3	C20H39N3O20	C11H17N3O2
C21H16N2OS3	C18H19N3O3S3	C21H21N3O5	C20H41NO5	C13H13N3O2S	C36H61N3O2
C20H14N2OS3	C23H17N3O3S3	C25H29N3O5	C22H45NO5	C14H15N3O2S	C31H43N3O2
C27H26N2OS3	C28H13N3O3S3	C27H33N3O5	C24H49NO5	C19H19N3O2S	C23H25N3O2
C33H38N2OS3	C13H27N3O4	C25H27N3O5	C11H21NO5	C34H27N3O2S	C24H25N3O2
C33H36N2OS3	C39H77N3O4	C26H29N3O5	C20H39NO5	C10H17N3O2S2	C26H29N3O2
C35H40N2OS3	C39H65N3O4	C25H25N3O5	C22H43NO5	C11H19N3O2S2	C23H21N3O2
C18H27N3	C41H69N3O4	C46H67N3O5	C11H19NO5	C8H13N3O2S2	C24H23N3O2
C11H11N3	C41H65N3O4	C28H19N3O5	C8H13NO5	C36H67N3O2S2	C27H29N3O2

C12H11N3	C21H21N3O4	C35H31N3O5	C10H15NO5	C15H21N3O2S2	C31H37N3O2
C25H33N3	C24H27N3O4	C41H27N3O5	C12H19NO5	C16H23N3O2S2	C23H19N3O2
C17H13N3	C25H27N3O4	C12H27N3O5S	C8H11NO5	C17H25N3O2S2	C23H17N3O2
C25H29N3	C26H29N3O4	C8H17N3O5S	C14H21NO5	C22H35N3O2S2	C27H25N3O2
C39H57N3	C27H31N3O4	C10H17N3O5S	C11H13NO5	C20H27N3O2S2	C28H25N3O2
C26H29N3	C21H17N3O4	C11H19N3O5S	C12H15NO5	C20H23N3O2S2	C50H69N3O2
C30H37N3	C47H59N3O4	C8H13N3O5S	C13H17NO5	C23H23N3O2S2	C25H17N3O2
C33H43N3	C30H19N3O4	C9H15N3O5S	C15H21NO5	C35H45N3O2S2	C40H31N3O2
C29H33N3	C34H13N3O4	C20H33N3O5S	C13H15NO5	C44H31N3O2S2	C39H25N3O2
C30H35N3	C37H17N3O4	C45H83N3O5S	C14H17NO5	C21H31N3O2S3	C41H29N3O2
C39H53N3	C48H29N3O4	C26H43N3O5S	C15H19NO5	C22H33N3O2S3	C39H21N3O2
C33H39N3	C50H29N3O4	C42H67N3O5S	C16H19NO5	C18H17N3O2S3	C44H23N3O2
C27H25N3	C12H27N3O4S	C36H33N3O5S	C32H41NO5	C23H25N3O2S3	C25H39N3O20
C29H29N3	C11H19N3O4S	C15H27N3O5S2	C22H17NO5	C10H15N3O3	C18H27N3O2S
C31H33N3	C10H15N3O4S	C19H35N3O5S2	C48H69NO5	C13H13N3O3	C45H69N3O2S
C32H35N3	C19H31N3O4S	C12H21N3O5S2	C37H45NO5	C14H15N3O3	C8H13N3O2S2
C34H39N3	C22H37N3O4S	C9H15N3O5S2	C22H15NO5	C15H11N3O3	C10H15N3O2S2
C35H41N3	C31H51N3O4S	C15H25N3O5S2	C48H65NO5	C25H17N3O3	C24H37N3O2S2
C36H43N3	C32H53N3O4S	C16H27N3O5S2	C46H55NO5	C38H39N3O3	C38H65N3O2S2
C41H49N3	C22H33N3O4S	C14H21N3O5S2	C40H39NO5	C36H27N3O3	C28H41N3O2S2
C25H13N3	C17H13N3O4S	C7H13N3O5S3	C40H15NO5	C41H31N3O3	C24H31N3O2S2
C41H45N3	C35H71N3O4S2	C20H35N3O5S3	C12H25NO5S	C36H19N3O3	C35H47N3O2S2
C33H23N3	C10H19N3O4S2	C43H51N3O5S3	C11H13NO5S	C48H31N3O3	C10H15N3O2S3
C41H33N3	C9H17N3O4S2	C10H15N3O6	C48H63NO5S	C18H19N3O3S	C21H15N3O2S3
C41H29N3	C17H25N3O4S2	C19H31N3O6	C40H77NO5S2	C20H19N3O3S	C9H13N3O3
C7H15N3O	C10H21N3O5	C38H67N3O6	C24H39NO5S2	C21H21N3O3S	C20H33N3O3
C7H13N3O	C10H17N3O5	C19H17N3O6	C16H15NO5S2	C22H23N3O3S	C41H63N3O3
C33H59N3O	C8H13N3O5	C24H25N3O6	C21H41NO6	C40H13N3O3S	C27H33N3O3
C10H11N3O	C9H15N3O5	C24H23N3O6	C15H25NO6	C10H19N3O3S2	C23H23N3O3
C22H29N3O	C12H13N3O5	C29H33N3O6	C17H27NO6	C8H15N3O3S2	C24H25N3O3
C22H25N3O	C12H11N3O5	C34H37N3O6	C19H31NO6	C15H23N3O3S2	C25H27N3O3
C39H57N3O	C21H25N3O5	C27H15N3O6	C13H19NO6	C16H25N3O3S2	C26H29N3O3
C26H29N3O	C33H47N3O5	C40H39N3O6	C14H21NO6	C17H27N3O3S2	C28H33N3O3
C29H35N3O	C21H21N3O5	C42H37N3O6	C12H15NO6	C18H29N3O3S2	C28H31N3O3
C24H23N3O	C25H29N3O5	C10H21N3O6S	C14H19NO6	C16H23N3O3S2	C38H51N3O3
C26H27N3O	C27H33N3O5	C9H19N3O6S	C19H27NO6	C23H31N3O3S2	C47H69N3O3
C28H31N3O	C25H27N3O5	C14H25N3O6S	C13H15NO6	C10H19N3O4	C29H31N3O3
C29H33N3O	C26H29N3O5	C15H27N3O6S	C15H19NO6	C11H21N3O4	C40H11N3O3
C30H35N3O	C25H25N3O5	C13H21N3O6S2	C16H21NO6	C12H23N3O4	C49H25N3O3
C28H29N3O	C25H23N3O5	C30H43N3O6S2	C15H17NO6	C13H25N3O4	C15H27N3O3S
C32H37N3O	C49H71N3O5	C33H19N3O6S2	C17H21NO6	C7H13N3O4	C12H17N3O3S
C27H25N3O	C26H23N3O5	C15H17N3O6S3	C19H23NO6	C9H17N3O4	C36H65N3O3S
C29H29N3O	C35H37N3O5	C23H17N3O6S3	C21H25NO6	C10H17N3O4	C34H59N3O3S
C30H31N3O	C28H19N3O5	C10H17N3O7	C17H15NO6	C11H19N3O4	C30H45N3O3S

C33H37N3O	C35H31N3O5	C22H37N3O7	C23H25NO6	C12H21N3O4	C43H71N3O3S
C34H39N3O	C12H27N3O5S	C10H13N3O7	C46H71NO6	C9H13N3O4	C40H13N3O3S
C26H21N3O	C13H29N3O5S	C22H35N3O7	C19H11NO6	C10H13N3O4	C10H21N3O3S2
C29H27N3O	C14H31N3O5S	C29H41N3O7	C21H15NO6	C32H49N3O4	C11H23N3O3S2
C26H17N3O	C8H17N3O5S	C31H43N3O7	C23H19NO6	C16H17N3O4	C15H31N3O3S2
C39H35N3O	C34H69N3O5S	C23H23N3O7	C47H53NO6	C22H13N3O4	C10H19N3O3S2
C38H31N3O	C10H17N3O5S	C31H39N3O7	C45H43NO6	C29H21N3O4	C11H21N3O3S2
C42H33N3O	C8H13N3O5S	C10H21N3O7S	C6H11NO6S	C30H23N3O4	C8H15N3O3S2
C34H17N3O	C9H15N3O5S	C18H29N3O7S	C15H23NO7	C36H27N3O4	C14H25N3O3S2
C38H23N3O	C34H65N3O5S	C24H39N3O7S	C16H21NO7	C32H17N3O4	C21H39N3O3S2
C40H25N3O	C26H43N3O5S	C17H15N3O7S	C18H23NO7	C40H21N3O4	C14H23N3O3S2
C47H23N3O	C31H51N3O5S	C13H27N3O7S2	C20H13NO7	C38H13N3O4	C16H27N3O3S2
C47H19N3O	C15H31N3O5S2	C17H31N3O7S2	C22H17NO7	C49H33N3O4	C35H59N3O3S2
C14H31N3O10	C15H27N3O5S2	C14H25N3O7S2	C46H65NO7	C36H71N3O4S	C22H33N3O3S3
C10H21N3O10	C19H35N3O5S2	C17H27N3O7S2	C49H53NO7	C16H17N3O4S	C18H19N3O3S3
C20H41N3O10	C10H17N3O5S2	C30H65N3O8	C15H29NO7S	C22H41N3O4S2	C21H25N3O3S3
C21H41N3O10	C11H19N3O5S2	C13H27N3O8	C29H57NO7S	C16H27N3O4S2	C13H27N3O4
C16H29N3O10	C12H21N3O5S2	C16H31N3O8	C20H39NO7S3	C15H21N3O4S2	C10H13N3O4
C17H31N3O10	C9H15N3O5S2	C9H15N3O8	C20H37NO7S3	C16H23N3O4S2	C19H29N3O4
C17H29N3O10	C13H21N3O5S2	C34H55N3O8	C18H13NO7S3	C17H25N3O4S2	C39H65N3O4
C32H59N3O10	C15H25N3O5S2	C16H17N3O8	C21H19NO7S3	C22H33N3O4S2	C41H69N3O4
C16H25N3O10	C16H27N3O5S2	C13H27N3O8S	C19H33NO8	C14H17N3O4S2	C41H65N3O4
C17H27N3O10	C8H11N3O5S2	C13H23N3O8S	C31H57NO8	C9H15N3O4S3	C21H21N3O4
C21H35N3O10	C9H13N3O5S2	C22H43N3O9	C17H23NO8	C19H17N3O4S3	C25H29N3O4
C36H65N3O10	C31H51N3O5S2	C17H29N3O9	C27H43NO8	C26H29N3O4S3	C27H33N3O4
C28H49N3O10	C43H51N3O5S2	C18H31N3O9	C44H69NO8	C44H25N3O4S3	C25H27N3O4
C32H55N3O10	C20H43N3O5S3	C18H29N3O9	C44H65NO8	C8H15N3O5	C26H29N3O4
C13H17N3O10	C10H19N3O5S3	C15H13N3O9	C26H29NO8	C9H17N3O5	C27H31N3O4
C36H61N3O10	C7H13N3O5S3	C27H37N3O9	C33H41NO8	C12H19N3O5	C21H17N3O4
C16H19N3O10	C21H13N3O5S3	C29H39N3O9	C28H31NO8	C9H13N3O5	C39H51N3O4
C18H23N3O10	C43H51N3O5S3	C45H67N3O9	C28H29NO8	C10H15N3O5	C49H71N3O4
C20H19N3O10	C8H17N3O6	C45H63N3O9	C45H49NO8	C11H17N3O5	C33H39N3O4
C28H23N3O10	C19H31N3O6	C45H59N3O9	C49H47NO8	C13H21N3O5	C49H69N3O4
C11H23N3O10S	C38H63N3O6	C24H11N3O9	C14H27NO8S	C14H23N3O5	C37H17N3O4
C23H43N3O10S	C19H17N3O6	C36H29N3O9	C20H15NO8S	C16H27N3O5	C12H27N3O4S
C23H39N3O10S	C24H25N3O6	C21H45N3O9S2	C23H21NO8S	C17H29N3O5	C13H29N3O4S
C22H45N3O10S2	C24H23N3O6	C27H55N3O9S2	C10H21NO9	C19H33N3O5	C32H55N3O4S
C26H39N3O10S2	C29H33N3O6	C17H33N3O9S2	C16H33NO9	C9H11N3O5	C32H53N3O4S
C29H33N3O10S2	C34H37N3O6	C27H51N3O9S2	C14H25NO9	C12H17N3O5	C17H13N3O4S
C20H39N3O11	C30H25N3O6	C35H71N3OS2	C28H49NO9	C13H19N3O5	C10H19N3O4S2
C21H41N3O11	C27H15N3O6	C7H13N3OS2	C30H43NO9	C15H23N3O5	C14H25N3O4S2
C15H25N3O11	C29H17N3O6	C7H11N3OS2	C23H21NO9	C16H25N3O5	C22H41N3O4S2
C16H27N3O11	C40H39N3O6	C24H27N3OS2	C37H15NO9	C19H31N3O5	C11H17N3O4S2
C21H37N3O11	C33H25N3O6	C33H31N3OS2	C14H19NOS	C13H17N3O5	C15H25N3O4S2

C16H25N3O11	C9H19N3O6S	C15H31N3OS3	C29H45NOS	C15H21N3O5	C9H13N3O4S2
C16H23N3O11	C15H27N3O6S	C14H25N3OS3	C17H11NOS	C16H19N3O5	C17H25N3O4S2
C21H21N3O11	C27H37N3O6S	C24H37N3OS3	C25H23NOS	C20H27N3O5	C37H59N3O4S2
C43H51N3O11	C42H25N3O6S	C24H35N3OS3	C12H27NOS2	C19H23N3O5	C44H67N3O4S2
C38H35N3O11	C14H31N3O6S2	C25H29N3OS3	C13H21NOS2	C23H13N3O5	C12H23N3O5
C20H31N3O11S	C18H39N3O6S2	C22H17N3OS3	C28H47NOS2	C24H15N3O5	C14H25N3O5
C12H13N3O11S	C18H37N3O6S2	C29H23N3OS3	C40H71NOS2	C25H17N3O5	C8H13N3O5
C36H47N3O11S	C35H71N3O6S2	C10H17NO	C47H71NOS2	C35H37N3O5	C13H21N3O5
C16H35N3O12	C30H43N3O6S2	C19H11NO	C22H19NOS2	C31H27N3O5	C10H15N3O5
C18H37N3O12	C22H37N3O7	C20H13NO	C47H67NOS2	C46H29N3O5	C12H19N3O5
C19H39N3O12	C22H35N3O7	C29H17NO	C33H37NOS2	C48H33N3O5	C16H27N3O5
C16H31N3O12	C29H41N3O7	C44H31NO	C37H21NOS2	C49H33N3O5	C17H29N3O5
C15H25N3O12	C27H35N3O7	C17H29NO10	C10H21NOS3	C45H23N3O5	C12H17N3O5
C20H35N3O12	C31H43N3O7	C29H53NO10	C15H31NOS3	C40H13N3O5	C16H25N3O5
C30H55N3O12	C23H23N3O7	C39H73NO10	C7H15NOS3	C50H25N3O5	C15H21N3O5
C15H23N3O12	C31H39N3O7	C17H25NO10	C8H17NOS3	C9H17N3O5S	C19H29N3O5
C16H25N3O12	C47H67N3O7	C27H45NO10	C21H41NOS3	C34H65N3O5S	C21H31N3O5
C30H51N3O12	C39H29N3O7	C25H39NO10	C46H87NOS3	C13H19N3O5S	C19H23N3O5
C30H47N3O12	C39H23N3O7	C14H17NO10	C44H77NOS3	C22H13N3O5S	C21H25N3O5
C41H59N3O12	C14H31N3O7S	C29H45NO10	C9H20O	C15H27N3O5S2	C33H47N3O5
C42H47N3O12	C14H29N3O7S	C23H33NO10	C18H36O	C12H21N3O5S2	C21H21N3O5
C13H15N3O13	C20H31N3O7S	C25H35NO10	C16H30O	C9H15N3O5S2	C25H29N3O5
C30H37N3O13	C24H39N3O7S	C19H23NO10	C8H14O	C15H25N3O5S2	C25H27N3O5
C34H39N3O13	C17H15N3O7S	C20H25NO10	C9H16O	C31H41N3O5S2	C49H75N3O5
C16H33N3O14	C29H59N3O7S2	C20H23NO10	C10H16O	C14H25N3O6	C25H25N3O5
C26H51N3O14	C14H25N3O7S2	C22H27NO10	C11H18O	C10H17N3O6	C37H49N3O5
C30H57N3O14	C17H27N3O7S2	C21H23NO10	C15H26O	C11H19N3O6	C25H23N3O5
C16H29N3O14	C29H47N3O7S2	C22H25NO10	C16H28O	C12H21N3O6	C39H51N3O5
C24H37N3O14	C39H29N3O7S2	C29H37NO10	C17H30O	C13H23N3O6	C49H71N3O5
C28H43N3O14	C30H65N3O8	C27H31NO10	C18H32O	C15H27N3O6	C26H23N3O5
C35H43N3O14	C13H27N3O8	C23H23NO10	C11H16O	C12H19N3O6	C38H45N3O5
C35H39N3O14	C16H31N3O8	C28H29NO10	C12H18O	C10H15N3O6	C39H11N3O5
C36H35N3O14	C24H47N3O8	C43H45NO10	C13H20O	C11H17N3O6	C12H27N3O5S
C32H65N3O15	C9H15N3O8	C39H31NO10	C14H22O	C13H21N3O6	C13H29N3O5S
C30H59N3O15	C34H63N3O8	C32H65NO10S	C15H24O	C14H23N3O6	C10H21N3O5S
C13H23N3O15	C34H55N3O8	C12H23NO10S	C16H26O	C15H25N3O6	C12H25N3O5S
C22H31N3O15	C45H71N3O8	C18H33NO10S	C18H30O	C16H27N3O6	C13H27N3O5S
C27H39N3O15	C31H29N3O8	C27H33NO10S	C8H10O	C17H29N3O6	C8H17N3O5S
C27H35N3O15	C39H27N3O8	C32H29NO10S	C10H12O	C19H33N3O6	C34H69N3O5S
C30H13N3O15	C13H27N3O8S	C30H47NO10S2	C13H18O	C12H17N3O6	C10H17N3O5S
C24H51N3O16	C13H23N3O8S	C23H43NO10S3	C14H20O	C13H19N3O6	C11H19N3O5S
C20H23N3O16	C39H81N3O8S2	C23H39NO10S3	C18H28O	C14H21N3O6	C8H13N3O5S
C33H35N3O16	C31H41N3O8S2	C28H45NO10S3	C15H20O	C16H25N3O6	C9H15N3O5S
C33H31N3O16	C22H43N3O9	C28H39NO10S3	C29H48O	C12H15N3O6	C34H65N3O5S

C19H21N3O16S	C18H29N3O9	C21H19NO10S3	C21H28O	C38H67N3O6	C18H31N3O5S
C28H59N3O17	C29H39N3O9	C31H37NO10S3	C23H30O	C14H19N3O6	C26H43N3O5S
C25H47N3O17	C29H35N3O9	C35H39NO10S3	C31H42O	C15H21N3O6	C42H67N3O5S
C18H31N3O17	C45H67N3O9	C12H25NO11	C17H12O	C16H23N3O6	C13H29N3O5S2
C27H41N3O17	C29H31N3O9	C26H45NO11	C17H10O	C15H19N3O6	C15H27N3O5S2
C27H37N3O17	C45H63N3O9	C19H19NO11	C19H14O	C38H63N3O6	C19H35N3O5S2
C25H31N3O17	C45H59N3O9	C28H29NO11	C26H26O	C16H19N3O6	C11H19N3O5S2
C33H43N3O17	C21H45N3O9S2	C38H45NO11	C27H28O	C17H21N3O6	C12H21N3O5S2
C21H39N3O17S2	C27H55N3O9S2	C24H15NO11	C24H14O	C18H23N3O6	C9H15N3O5S2
C27H47N3O18	C17H33N3O9S2	C33H33NO11	C28H10O	C19H25N3O6	C13H21N3O5S2
C19H25N3O18	C27H51N3O9S2	C35H35NO11	C32H16O	C19H23N3O6	C16H27N3O5S2
C26H47N3O19	C37H35N3O9S2	C41H47NO11	C37H22O	C19H21N3O6	C9H13N3O5S2
C25H33N3O19	C39H39N3O9S2	C39H69NO12	C36H14O	C18H17N3O6	C19H27N3O5S2
C11H17N3O2	C17H35N3O9S3	C23H35NO12	C13H28O10	C19H19N3O6	C29H27N3O5S2
C11H13N3O2	C31H67N3OS2	C39H65NO12	C14H30O10	C20H21N3O6	C39H25N3O5S2
C25H31N3O2	C35H71N3OS2	C23H31NO12	C15H32O10	C19H17N3O6	C8H17N3O6
C31H43N3O2	C7H13N3OS2	C27H37NO12	C16H34O10	C25H27N3O6	C9H13N3O6
C23H25N3O2	C35H67N3OS2	C23H27NO12	C17H36O10	C20H11N3O6	C12H19N3O6
C24H27N3O2	C7H11N3OS2	C36H37NO12	C18H38O10	C34H27N3O6	C13H21N3O6
C25H29N3O2	C23H39N3OS2	C26H37NO12S3	C23H48O10	C36H31N3O6	C14H23N3O6
C24H25N3O2	C25H33N3OS2	C28H37NO12S3	C25H52O10	C10H21N3O6S	C15H25N3O6
C27H31N3O2	C28H33N3OS2	C35H65NO13	C14H28O10	C15H27N3O6S	C16H27N3O6
C23H21N3O2	C26H27N3OS2	C24H37NO13	C23H46O10	C8H15N3O6S2	C19H31N3O6
C26H27N3O2	C14H25N3OS3	C26H33NO13	C25H50O10	C21H29N3O6S2	C13H19N3O6
C31H37N3O2	C26H33N3OS3	C26H29NO13	C26H52O10	C28H33N3O6S2	C21H35N3O6
C23H19N3O2	C16H13N3OS3	C39H45NO13	C27H54O10	C28H31N3O6S2	C21H31N3O6
C28H29N3O2	C25H29N3OS3	C39H39NO13	C25H48O10	C28H23N3O6S2	C25H39N3O6
C31H35N3O2	C22H17N3OS3	C36H29NO14	C27H52O10	C33H19N3O6S2	C19H17N3O6
C23H17N3O2	C22H41NO	C39H31NO14	C29H56O10	C14H25N3O7	C24H25N3O6
C27H25N3O2	C10H15NO	C14H21NO14S3	C11H14O10	C12H21N3O7	C24H23N3O6
C31H33N3O2	C19H11NO	C14H31NO15	C17H26O10	C11H17N3O7	C29H33N3O6
C32H35N3O2	C50H67NO	C34H35NO15	C14H18O10	C12H19N3O7	C33H41N3O6
C33H37N3O2	C36H37NO	C38H67NO16	C16H22O10	C14H23N3O7	C27H15N3O6
C28H25N3O2	C36H35NO	C28H39NO16	C16H20O10	C15H25N3O7	C12H27N3O6S
C39H47N3O2	C50H63NO	C17H33NO17	C17H22O10	C16H27N3O7	C13H29N3O6S
C40H49N3O2	C29H17NO	C25H49NO17	C18H24O10	C17H29N3O7	C14H31N3O6S
C25H17N3O2	C36H21NO	C32H37NO17	C19H26O10	C10H13N3O7	C15H33N3O6S
C25H13N3O2	C44H31NO	C26H19NO17	C20H28O10	C11H15N3O7	C10H21N3O6S
C27H17N3O2	C44H27NO	C33H19NO17	C15H16O10	C12H17N3O7	C9H19N3O6S
C29H21N3O2	C17H29NO10	C26H37NO18	C16H18O10	C13H19N3O7	C15H27N3O6S
C36H35N3O2	C28H51NO10	C26H31NO18	C17H20O10	C14H21N3O7	C11H17N3O6S
C46H49N3O2	C17H25NO10	C8H17NO2	C18H22O10	C15H23N3O7	C20H29N3O6S
C39H25N3O2	C27H45NO10	C11H21NO2	C19H24O10	C16H25N3O7	C35H15N3O6S
C41H21N3O2	C29H49NO10	C13H25NO2	C20H26O10	C17H27N3O7	C42H25N3O6S

C49H29N3O2	C39H69NO10	C6H11NO2	C21H28O10	C18H29N3O7	C18H39N3O6S2
C25H39N3O20	C25H39NO10	C8H15NO2	C22H30O10	C13H17N3O7	C18H37N3O6S2
C24H45N3O2S	C28H45NO10	C7H11NO2	C23H32O10	C14H19N3O7	C15H27N3O6S2
C18H27N3O2S	C14H17NO10	C11H17NO2	C24H34O10	C15H21N3O7	C10H17N3O6S2
C45H69N3O2S	C29H45NO10	C12H19NO2	C17H18O10	C16H23N3O7	C10H15N3O6S2
C45H65N3O2S	C20H25NO10	C13H21NO2	C18H20O10	C17H25N3O7	C30H43N3O6S2
C24H21N3O2S	C25H35NO10	C33H61NO2	C19H22O10	C17H23N3O7	C10H17N3O7
C31H31N3O2S	C23H31NO10	C13H19NO2	C20H24O10	C16H19N3O7	C16H27N3O7
C10H17N3O2S2	C26H35NO10	C14H21NO2	C21H26O10	C37H47N3O7	C10H13N3O7
C8H13N3O2S2	C42H65NO10	C9H11NO2	C22H28O10	C27H23N3O7	C14H21N3O7
C10H15N3O2S2	C21H23NO10	C11H13NO2	C23H30O10	C26H17N3O7	C17H27N3O7
C38H65N3O2S2	C22H25NO10	C15H13NO2	C19H20O10	C27H19N3O7	C18H29N3O7
C28H41N3O2S2	C22H23NO10	C16H15NO2	C20H22O10	C34H67N3O7S	C17H25N3O7
C24H31N3O2S2	C24H27NO10	C24H17NO2	C21H24O10	C10H17N3O7S	C21H33N3O7
C37H37N3O2S2	C27H31NO10	C28H25NO2	C22H26O10	C12H23N3O8	C19H27N3O7
C17H37N3O2S3	C23H23NO10	C25H17NO2	C23H28O10	C9H13N3O8	C21H31N3O7
C21H41N3O2S3	C24H25NO10	C49H53NO2	C25H32O10	C14H23N3O8	C29H41N3O7
C11H17N3O2S3	C25H27NO10	C43H39NO2	C20H20O10	C15H25N3O8	C31H43N3O7
C23H37N3O2S3	C31H37NO10	C37H17NO2	C21H22O10	C17H29N3O8	C23H23N3O7
C46H71N3O2S3	C23H21NO10	C27H43NO20	C22H24O10	C13H19N3O8	C36H45N3O7
C20H13N3O2S3	C28H29NO10	C28H33NO2S	C25H30O10	C14H21N3O8	C47H67N3O7
C21H15N3O2S3	C42H51NO10	C11H19NO2S2	C18H12O10	C15H23N3O8	C10H21N3O7S
C23H19N3O2S3	C43H45NO10	C27H31NO2S2	C24H50O10S	C16H25N3O8	C11H23N3O7S
C13H27N3O3	C41H31NO10	C43H47NO2S2	C25H52O10S	C17H27N3O8	C24H39N3O7S
C9H19N3O3	C12H23NO10S	C48H57NO2S2	C26H54O10S	C16H23N3O8	C17H15N3O7S
C14H29N3O3	C18H29NO10S	C34H23NO2S2	C22H44O10S	C19H29N3O8	C14H25N3O7S2
C26H33N3O3	C32H57NO10S	C50H43NO2S2	C25H50O10S	C22H21N3O8	C29H51N3O7S2
C27H35N3O3	C30H47NO10S2	C50H27NO2S2	C27H54O10S	C23H21N3O8	C16H35N3O8
C41H63N3O3	C29H19NO10S2	C18H37NO2S3	C24H46O10S	C33H39N3O8	C16H31N3O8
C16H13N3O3	C23H43NO10S3	C11H21NO2S3	C26H50O10S	C26H19N3O8	C9H15N3O8
C24H27N3O3	C23H39NO10S3	C32H47NO2S3	C27H52O10S	C27H21N3O8	C16H25N3O8
C28H35N3O3	C28H45NO10S3	C48H79NO2S3	C20H36O10S	C36H31N3O8	C19H31N3O8
C23H23N3O3	C28H41NO10S3	C32H43NO2S3	C26H52O10S3	C31H19N3O8	C21H33N3O8
C24H25N3O3	C28H39NO10S3	C32H35NO2S3	C26H50O10S3	C45H33N3O8	C27H37N3O8
C26H29N3O3	C31H37NO10S3	C41H39NO2S3	C14H20O10S3	C14H23N3O9	C31H33N3O8
C27H31N3O3	C35H39NO10S3	C41H35NO2S3	C15H14O10S3	C17H29N3O9	C31H29N3O8
C28H33N3O3	C12H25NO11	C32H17NO2S3	C31H30O10S3	C15H23N3O9	C47H53N3O8
C29H35N3O3	C26H45NO11	C12H23NO3	C14H30O11	C16H25N3O9	C13H23N3O8S
C26H27N3O3	C26H41NO11	C7H13NO3	C15H32O11	C17H27N3O9	C24H39N3O8S
C27H29N3O3	C40H69NO11	C11H21NO3	C16H34O11	C16H23N3O9	C22H29N3O8S
C29H33N3O3	C39H65NO11	C8H15NO3	C17H36O11	C17H25N3O9	C29H19N3O8S2
C28H27N3O3	C12H11NO11	C9H17NO3	C18H38O11	C17H23N3O9	C22H43N3O9
C29H29N3O3	C13H13NO11	C10H17NO3	C19H40O11	C18H25N3O9	C18H29N3O9
C31H33N3O3	C17H15NO11	C14H25NO3	C20H42O11	C29H39N3O9	C18H27N3O9

C32H35N3O3	C19H19NO11	C7H11NO3	C22H46O11	C20H15N3O9	C21H31N3O9
C28H25N3O3	C22H25NO11	C9H15NO3	C23H48O11	C33H33N3O9	C29H39N3O9
C30H29N3O3	C19H15NO11	C11H17NO3	C24H50O11	C31H27N3O9	C45H67N3O9
C31H31N3O3	C28H29NO11	C12H19NO3	C25H52O11	C44H23N3O9	C45H63N3O9
C38H45N3O3	C26H25NO11	C13H21NO3	C16H32O11	C14H19N3O9S	C45H59N3O9
C40H49N3O3	C41H47NO11	C18H31NO3	C17H34O11	C22H35N3O9S	C36H29N3O9
C41H51N3O3	C13H21NO11S	C10H13NO3	C18H36O11	C18H25N3O9S	C38H33N3O9
C28H23N3O3	C24H41NO11S	C11H15NO3	C19H38O11	C40H39N3O9S2	C22H35N3O9S
C26H13N3O3	C24H37NO11S	C12H17NO3	C20H40O11	C23H41N3OS	C37H41N3O9S
C40H11N3O3	C22H41NO11S3	C13H19NO3	C21H42O11	C12H15N3OS	C35H71N3OS2
C34H59N3O3S	C12H15NO11S3	C9H11NO3	C22H44O11	C34H27N3OS	C7H13N3OS2
C30H45N3O3S	C39H69NO12	C11H13NO3	C23H46O11	C37H15N3OS	C7H11N3OS2
C43H71N3O3S	C23H35NO12	C12H15NO3	C24H48O11	C10H17N3OS2	C23H39N3OS2
C41H33N3O3S	C15H17NO12	C13H17NO3	C25H50O11	C35H67N3OS2	C25H33N3OS2
C40H13N3O3S	C23H31NO12	C14H19NO3	C26H52O11	C7H11N3OS2	C24H27N3OS2
C10H21N3O3S2	C27H37NO12	C10H11NO3	C27H54O11	C13H21N3OS2	C37H49N3OS2
C11H23N3O3S2	C23H27NO12	C13H15NO3	C21H40O11	C16H25N3OS2	C33H31N3OS2
C10H19N3O3S2	C27H33NO12	C12H11NO3	C22H42O11	C23H39N3OS2	C30H21N3OS2
C11H21N3O3S2	C23H25NO12	C17H13NO3	C24H46O11	C15H21N3OS2	C15H33N3OS3
C12H23N3O3S2	C36H37NO12	C22H23NO3	C25H48O11	C22H35N3OS2	C15H31N3OS3
C13H25N3O3S2	C38H37NO12	C20H17NO3	C26H50O11	C48H31N3OS2	C12H17N3OS3
C14H27N3O3S2	C41H19NO12	C34H45NO3	C27H52O11	C21H29N3OS3	C16H13N3OS3
C15H29N3O3S2	C26H37NO12S3	C42H61NO3	C29H56O11	C22H31N3OS3	C39H29N3OS3
C23H45N3O3S2	C24H45NO13	C26H25NO3	C12H10O11	C25H35N3OS3	C45H35N3OS3
C25H49N3O3S2	C37H71NO13	C42H57NO3	C18H22O11	C24H27N3OS3	C10H17NO
C8H15N3O3S2	C12H21NO13	C32H35NO3	C19H24O11	C31H29N3OS3	C22H41NO
C14H25N3O3S2	C35H65NO13	C26H21NO3	C20H26O11	C23H13N3OS3	C24H45NO
C21H39N3O3S2	C31H51NO13	C33H33NO3	C23H32O11	C48H35N3OS3	C20H25NO
C16H27N3O3S2	C26H29NO13	C34H35NO3	C20H24O11	C24H47NO	C44H67NO
C9H13N3O3S2	C39H45NO13	C33H31NO3	C21H26O11	C22H41NO	C50H67NO
C21H35N3O3S2	C34H33NO14	C34H31NO3	C22H28O11	C22H39NO	C36H37NO
C15H21N3O3S2	C30H19NO14	C36H29NO3	C23H30O11	C25H37NO	C36H35NO
C17H25N3O3S2	C31H19NO14	C42H23NO3	C19H20O11	C16H15NO	C50H63NO
C28H47N3O3S2	C36H29NO14	C23H33NO3S	C20H22O11	C17H15NO	C29H17NO
C18H19N3O3S3	C28H25NO14S3	C17H17NO3S	C21H24O11	C18H17NO	C36H21NO
C21H25N3O3S3	C14H31NO15	C25H33NO3S	C22H26O11	C19H19NO	C17H29NO10
C20H11N3O3S3	C20H37NO15	C27H37NO3S	C23H28O11	C23H15NO	C28H51NO10
C22H15N3O3S3	C29H33NO15	C21H23NO3S	C24H30O11	C50H63NO	C39H73NO10
C23H17N3O3S3	C34H41NO15	C27H29NO3S	C22H24O11	C29H17NO	C41H75NO10
C27H13N3O3S3	C34H35NO15	C30H57NO3S2	C23H26O11	C30H13NO	C17H25NO10
C41H65N3O4	C35H71NO16	C18H29NO3S2	C24H28O11	C33H13NO	C29H49NO10
C27H35N3O4	C38H67NO16	C16H23NO3S2	C26H32O11	C38H21NO	C31H53NO10
C21H21N3O4	C28H39NO16	C26H43NO3S2	C26H30O11	C35H13NO	C25H39NO10
C26H29N3O4	C28H35NO16	C26H41NO3S2	C42H10O11	C42H19NO	C28H45NO10

C27H31N3O4	C36H15NO16	C23H33NO3S2	C19H38O11S	C45H19NO	C14H17NO10
C28H33N3O4	C19H35NO17	C26H39NO3S2	C37H16O11S	C10H19NO10	C29H45NO10
C29H35N3O4	C18H33NO17	C32H41NO3S2	C21H22O11S2	C12H23NO10	C25H35NO10
C21H17N3O4	C21H17NO17	C28H27NO3S2	C30H24O11S2	C13H25NO10	C22H29NO10
C27H25N3O4	C32H37NO17	C44H53NO3S2	C28H20O11S2	C14H25NO10	C20H23NO10
C48H61N3O4	C23H39NO18	C42H39NO3S2	C26H12O11S2	C15H27NO10	C25H33NO10
C30H19N3O4	C23H35NO18	C42H35NO3S2	C19H22O11S3	C16H29NO10	C42H65NO10
C37H17N3O4	C29H37NO18	C43H35NO3S2	C18H12O11S3	C18H33NO10	C27H31NO10
C47H31N3O4	C8H17NO2	C33H69NO3S3	C14H30O12	C13H23NO10	C29H33NO10
C12H27N3O4S	C11H21NO2	C13H27NO3S3	C15H32O12	C13H21NO10	C32H17NO10
C13H29N3O4S	C13H25NO2	C19H39NO3S3	C21H26O12	C14H21NO10	C37H13NO10
C10H15N3O4S	C6H11NO2	C19H37NO3S3	C22H24O12	C17H25NO10	C32H65NO10S
C11H17N3O4S	C7H13NO2	C13H23NO3S3	C25H30O12	C25H39NO10	C18H33NO10S
C19H31N3O4S	C8H15NO2	C19H33NO3S3	C31H12O12	C22H27NO10	C13H23NO10S
C20H29N3O4S	C10H15NO2	C13H19NO3S3	C12H12O12S	C32H43NO10	C27H37NO10S
C22H33N3O4S	C12H19NO2	C18H23NO3S3	C23H24O12S	C19H13NO10	C12H25NO10S3
C20H41N3O4S2	C33H61NO2	C26H23NO3S3	C14H28O13	C19H11NO10	C23H39NO10S3
C10H19N3O4S2	C13H19NO2	C31H31NO3S3	C15H28O13	C21H15NO10	C28H45NO10S3
C20H39N3O4S2	C14H21NO2	C6H13NO4	C18H12O13	C30H33NO10	C28H39NO10S3
C22H41N3O4S2	C11H13NO2	C7H15NO4	C24H22O13S2	C32H29NO10	C12H25NO11
C15H25N3O4S2	C26H25NO2	C8H17NO4	C34H18O15	C40H33NO10	C33H65NO11
C16H27N3O4S2	C38H47NO2	C9H19NO4	C17H12O16	C45H41NO10	C21H25NO11
C17H29N3O4S2	C39H49NO2	C10H19NO4	C35H46O16	C45H27NO10	C15H25NO11S
C9H13N3O4S2	C46H63NO2	C11H21NO4	C34H24O16	C23H33NO10S3	C13H21NO11S
C12H17N3O4S2	C22H13NO2	C9H17NO4	C22H36O17	C28H41NO10S3	C38H37NO11S
C16H25N3O4S2	C38H45NO2	C31H61NO4	C31H14O17	C28H39NO10S3	C23H35NO12
C17H25N3O4S2	C26H21NO2	C10H17NO4	C27H22O17S2	C31H43NO10S3	C15H17NO12
C30H35N3O4S2	C43H39NO2	C11H19NO4	C28H56O19	C12H25NO11	C23H31NO12
C21H35N3O4S3	C43H35NO2	C12H21NO4	C20H18O19	C14H29NO11	C27H33NO12
C11H15N3O4S3	C37H17NO2	C13H23NO4	C23H24O19	C14H25NO11	C36H37NO12
C12H17N3O4S3	C48H19NO2	C14H25NO4	C18H38O2	C15H25NO11	C18H37NO12S
C19H19N3O4S3	C16H33NO2S	C9H15NO4	C5H10O2	C16H27NO11	C37H71NO13
C21H23N3O4S3	C8H13NO2S	C18H33NO4	C6H12O2	C15H23NO11	C35H65NO13
C44H55N3O4S3	C18H21NO2S	C7H11NO4	C7H14O2	C27H29NO11	C39H39NO13
C44H51N3O4S3	C49H81NO2S	C8H13NO4	C11H22O2	C21H13NO11	C21H33NO14
C14H25N3O5	C20H23NO2S	C10H15NO4	C8H16O2	C21H11NO11	C30H19NO14
C8H13N3O5	C28H29NO2S	C11H17NO4	C9H18O2	C40H35NO11	C36H29NO14
C17H25N3O5	C36H43NO2S	C12H19NO4	C13H24O2	C40H33NO11	C34H39NO15
C23H37N3O5	C11H19NO2S2	C13H21NO4	C22H42O2	C44H35NO11	C38H67NO16
C12H13N3O5	C29H37NO2S2	C14H23NO4	C23H44O2	C28H45NO11S	C28H39NO16
C16H17N3O5	C27H31NO2S2	C18H31NO4	C6H10O2	C25H29NO11S	C17H33NO17
C21H25N3O5	C45H57NO2S2	C8H11NO4	C7H12O2	C14H27NO12	C32H37NO17
C26H35N3O5	C35H31NO2S2	C9H13NO4	C10H18O2	C16H31NO12	C24H45NO18
C26H33N3O5	C43H47NO2S2	C11H15NO4	C11H20O2	C16H25NO12	C26H31NO18

C33H47N3O5	C36H25NO2S2	C12H17NO4	C12H22O2	C23H35NO12	C14H29NO2
C21H21N3O5	C50H47NO2S2	C13H19NO4	C8H14O2	C23H31NO12	C5H11NO2
C25H29N3O5	C50H43NO2S2	C14H21NO4	C9H16O2	C26H35NO12	C6H13NO2
C27H33N3O5	C49H35NO2S2	C15H23NO4	C11H18O2	C27H27NO12	C8H17NO2
C28H35N3O5	C10H21NO2S3	C16H25NO4	C12H20O2	C22H15NO12	C11H21NO2
C25H27N3O5	C18H37NO2S3	C10H13NO4	C14H24O2	C40H33NO12	C12H23NO2
C49H75N3O5	C14H17NO2S3	C9H11NO4	C15H26O2	C41H35NO12	C13H25NO2
C25H25N3O5	C15H19NO2S3	C11H13NO4	C17H30O2	C44H33NO12	C22H43NO2
C25H23N3O5	C16H21NO2S3	C12H15NO4	C21H38O2	C21H45NO12S3	C26H51NO2
C26H23N3O5	C18H25NO2S3	C13H17NO4	C22H40O2	C21H43NO12S3	C6H11NO2
C26H19N3O5	C28H41NO2S3	C14H19NO4	C23H42O2	C26H37NO12S3	C8H15NO2
C35H37N3O5	C20H23NO2S3	C10H11NO4	C7H10O2	C31H47NO12S3	C9H17NO2
C28H19N3O5	C32H47NO2S3	C14H17NO4	C10H16O2	C15H29NO13	C13H23NO2
C35H31N3O5	C32H43NO2S3	C15H19NO4	C20H36O2	C18H35NO13	C14H25NO2
C43H37N3O5	C32H39NO2S3	C16H21NO4	C8H12O2	C21H41NO13	C22H41NO2
C12H27N3O5S	C32H35NO2S3	C27H43NO4	C9H14O2	C17H33NO13	C10H15NO2
C13H29N3O5S	C41H39NO2S3	C11H11NO4	C12H18O2	C17H31NO13	C11H17NO2
C14H31N3O5S	C43H43NO2S3	C12H13NO4	C13H20O2	C17H27NO13	C12H19NO2
C10H21N3O5S	C41H35NO2S3	C13H15NO4	C14H22O2	C21H33NO13	C13H21NO2
C11H23N3O5S	C32H17NO2S3	C37H61NO4	C17H28O2	C26H41NO13	C33H61NO2
C13H27N3O5S	C42H29NO2S3	C12H11NO4	C10H14O2	C33H37NO13	C9H13NO2
C14H29N3O5S	C44H13NO2S3	C13H13NO4	C11H16O2	C36H43NO13	C11H15NO2
C8H17N3O5S	C12H23NO3	C14H15NO4	C8H10O2	C40H35NO13	C14H21NO2
C10H17N3O5S	C10H19NO3	C15H17NO4	C12H16O2	C15H29NO14	C15H23NO2
C11H19N3O5S	C11H21NO3	C16H19NO4	C13H18O2	C16H31NO14	C9H11NO2
C8H13N3O5S	C8H15NO3	C17H21NO4	C17H26O2	C12H23NO14	C14H19NO2
C9H15N3O5S	C9H17NO3	C22H29NO4	C18H28O2	C17H31NO14	C11H13NO2
C34H65N3O5S	C10H17NO3	C14H13NO4	C9H10O2	C19H35NO14	C49H77NO2
C10H15N3O5S	C11H19NO3	C15H15NO4	C10H12O2	C15H25NO14	C42H57NO2
C11H17N3O5S	C14H25NO3	C16H17NO4	C11H14O2	C17H29NO14	C26H25NO2
C18H31N3O5S	C7H11NO3	C17H19NO4	C20H32O2	C18H31NO14	C38H47NO2
C19H33N3O5S	C9H15NO3	C22H27NO4	C11H12O2	C19H33NO14	C39H49NO2
C34H61N3O5S	C11H17NO3	C23H29NO4	C12H14O2	C16H25NO14	C25H19NO2
C11H13N3O5S	C13H21NO3	C25H33NO4	C13H16O2	C14H21NO14	C38H45NO2
C26H43N3O5S	C10H15NO3	C31H45NO4	C20H30O2	C18H27NO14	C39H47NO2
C42H67N3O5S	C18H31NO3	C15H13NO4	C28H44O2	C25H37NO14	C28H25NO2
C36H23N3O5S	C8H11NO3	C17H17NO4	C23H32O2	C18H13NO14	C25H17NO2
C15H27N3O5S2	C9H13NO3	C22H25NO4	C32H44O2	C19H13NO14	C36H35NO2
C19H35N3O5S2	C10H13NO3	C23H27NO4	C17H12O2	C38H33NO14	C43H39NO2
C10H17N3O5S2	C11H15NO3	C23H25NO4	C18H14O2	C39H31NO14	C45H37NO2
C11H19N3O5S2	C12H17NO3	C17H11NO4	C24H26O2	C41H29NO14	C50H29NO2
C12H21N3O5S2	C13H19NO3	C18H13NO4	C34H46O2	C16H29NO15	C43H13NO2
C9H15N3O5S2	C9H11NO3	C23H23NO4	C18H12O2	C17H31NO15	C16H33NO2S
C13H21N3O5S2	C11H13NO3	C19H11NO4	C19H14O2	C18H33NO15	C8H13NO2S

C16H27N3O5S2	C12H15NO3	C20H13NO4	C24H24O2	C19H35NO15	C26H41NO2S
C9H13N3O5S2	C13H17NO3	C23H19NO4	C26H28O2	C20H37NO15	C20H23NO2S
C14H21N3O5S2	C14H19NO3	C25H23NO4	C29H34O2	C21H39NO15	C49H77NO2S
C31H51N3O5S2	C10H11NO3	C25H21NO4	C22H18O2	C17H29NO15	C11H19NO2S2
C43H51N3O5S2	C13H15NO3	C32H33NO4	C29H32O2	C18H31NO15	C40H77NO2S2
C20H43N3O5S3	C11H11NO3	C22H13NO4	C31H36O2	C19H33NO15	C19H27NO2S2
C10H19N3O5S3	C12H13NO3	C32H31NO4	C36H46O2	C20H35NO15	C27H31NO2S2
C7H13N3O5S3	C15H19NO3	C33H33NO4	C28H28O2	C21H37NO15	C45H57NO2S2
C20H37N3O5S3	C16H21NO3	C32H29NO4	C30H32O2	C22H39NO15	C44H53NO2S2
C21H39N3O5S3	C12H11NO3	C32H27NO4	C26H20O2	C23H41NO15	C35H31NO2S2
C20H35N3O5S3	C14H13NO3	C41H39NO4	C29H10O2	C14H21NO15	C43H47NO2S2
C21H13N3O5S3	C23H29NO3	C39H33NO4	C39H12O2	C19H15NO15	C18H37NO2S3
C10H15N3O6	C20H17NO3	C11H19NO4S	C19H40O2S	C35H45NO15	C18H25NO2S3
C11H17N3O6	C34H45NO3	C22H39NO4S	C19H38O2S	C38H29NO15	C19H27NO2S3
C8H11N3O6	C25H25NO3	C23H41NO4S	C10H16O2S	C15H25NO16	C32H47NO2S3
C19H31N3O6	C42H59NO3	C13H19NO4S	C11H18O2S	C20H35NO16	C32H39NO2S3
C23H37N3O6	C26H25NO3	C22H37NO4S	C13H22O2S	C21H37NO16	C41H39NO2S3
C23H35N3O6	C34H41NO3	C22H35NO4S	C16H28O2S	C19H31NO16	C41H35NO2S3
C23H33N3O6	C42H57NO3	C23H37NO4S	C18H32O2S	C20H33NO16	C44H31NO2S3
C24H25N3O6	C26H23NO3	C22H33NO4S	C16H20O2S	C21H35NO16	C22H45NO3
C24H23N3O6	C26H21NO3	C23H35NO4S	C26H40O2S	C22H37NO16	C24H49NO3
C29H33N3O6	C26H19NO3	C24H37NO4S	C31H50O2S	C22H29NO16	C12H23NO3
C26H25N3O6	C33H33NO3	C23H33NO4S	C18H22O2S	C24H33NO16	C14H27NO3
C34H37N3O6	C33H31NO3	C18H21NO4S	C31H48O2S	C30H39NO16	C15H29NO3
C33H29N3O6	C34H31NO3	C23H29NO4S	C33H52O2S	C25H17NO16	C22H43NO3
C27H15N3O6	C27H15NO3	C26H31NO4S	C35H56O2S	C36H35NO16	C7H13NO3
C29H17N3O6	C36H29NO3	C26H21NO4S	C12H10O2S	C16H29NO17	C9H17NO3
C40H39N3O6	C44H35NO3	C50H43NO4S	C15H16O2S	C19H31NO17	C10H19NO3
C33H25N3O6	C42H23NO3	C10H21NO4S2	C16H18O2S	C22H35NO17	C11H21NO3
C12H27N3O6S	C42H19NO3	C27H45NO4S2	C17H20O2S	C15H29NO18	C8H15NO3
C13H29N3O6S	C41H69NO3S	C29H37NO4S2	C27H40O2S	C20H37NO18	C10H17NO3
C14H31N3O6S	C17H19NO3S	C25H23NO4S2	C33H50O2S	C21H39NO18	C11H19NO3
C15H33N3O6S	C25H35NO3S	C39H47NO4S2	C35H54O2S	C23H43NO18	C12H21NO3
C10H21N3O6S	C19H21NO3S	C48H39NO4S2	C37H58O2S	C24H45NO18	C14H25NO3
C11H23N3O6S	C27H37NO3S	C18H19NO4S3	C43H70O2S	C23H41NO18	C22H41NO3
C12H25N3O6S	C41H65NO3S	C30H39NO4S3	C45H74O2S	C22H37NO18	C7H11NO3
C13H27N3O6S	C26H33NO3S	C40H43NO4S3	C13H10O2S	C21H39NO19	C8H13NO3
C7H15N3O6S	C27H35NO3S	C39H31NO4S3	C14H12O2S	C23H39NO19	C9H15NO3
C9H19N3O6S	C25H29NO3S	C35H21NO4S3	C15H14O2S	C25H39NO19	C11H17NO3
C9H17N3O6S	C26H31NO3S	C10H21NO5	C16H16O2S	C29H21NO19	C12H19NO3
C15H27N3O6S	C27H33NO3S	C11H23NO5	C17H18O2S	C5H11NO2	C13H21NO3
C12H21N3O6S	C41H61NO3S	C18H37NO5	C18H20O2S	C6H13NO2	C10H13NO3
C10H15N3O6S	C26H29NO3S	C20H41NO5	C27H38O2S	C8H17NO2	C11H15NO3
C11H17N3O6S	C27H29NO3S	C22H45NO5	C29H42O2S	C13H25NO2	C12H17NO3

C12H19N3O6S	C41H57NO3S	C24H49NO5	C30H44O2S	C22H43NO2	C13H19NO3
C27H37N3O6S	C30H57NO3S2	C25H51NO5	C32H46O2S	C26H51NO2	C9H11NO3
C42H25N3O6S	C30H53NO3S2	C8H17NO5	C33H48O2S	C6H11NO2	C11H13NO3
C14H31N3O6S2	C16H23NO3S2	C9H19NO5	C35H52O2S	C13H23NO2	C12H15NO3
C30H43N3O6S2	C26H43NO3S2	C10H19NO5	C36H54O2S	C22H41NO2	C13H17NO3
C28H31N3O6S2	C26H39NO3S2	C11H21NO5	C37H56O2S	C10H15NO2	C14H19NO3
C23H17N3O6S2	C26H35NO3S2	C12H23NO5	C43H68O2S	C11H17NO2	C10H11NO3
C33H19N3O6S2	C32H41NO3S2	C13H25NO5	C45H72O2S	C11H15NO2	C13H15NO3
C20H39N3O6S3	C28H31NO3S2	C20H39NO5	C14H10O2S	C9H11NO2	C25H39NO3
C15H17N3O6S3	C45H65NO3S2	C22H43NO5	C16H14O2S	C16H13NO2	C11H11NO3
C23H17N3O6S3	C28H27NO3S2	C24H47NO5	C18H18O2S	C17H15NO2	C16H19NO3
C22H37N3O7	C45H61NO3S2	C7H13NO5	C25H30O2S	C18H17NO2	C12H11NO3
C10H13N3O7	C26H13NO3S2	C9H17NO5	C28H36O2S	C19H19NO2	C13H13NO3
C22H35N3O7	C42H35NO3S2	C16H31NO5	C29H38O2S	C26H25NO2	C14H13NO3
C23H37N3O7	C43H35NO3S2	C10H17NO5	C45H70O2S	C24H21NO2	C42H63NO3
C29H41N3O7	C33H69NO3S3	C11H19NO5	C47H74O2S	C23H17NO2	C34H45NO3
C31H43N3O7	C13H27NO3S3	C13H23NO5	C16H12O2S	C24H19NO2	C26H25NO3
C23H23N3O7	C19H37NO3S3	C14H25NO5	C18H16O2S	C25H21NO2	C42H57NO3
C25H27N3O7	C33H65NO3S3	C15H27NO5	C19H18O2S	C26H23NO2	C32H35NO3
C31H39N3O7	C13H23NO3S3	C12H21NO5	C22H24O2S	C30H31NO2	C26H21NO3
C31H37N3O7	C19H33NO3S3	C18H33NO5	C27H32O2S	C23H15NO2	C33H31NO3
C31H35N3O7	C13H19NO3S3	C8H13NO5	C45H68O2S	C39H47NO2	C34H31NO3
C36H45N3O7	C18H23NO3S3	C9H15NO5	C47H72O2S	C31H27NO2	C8H17NO3S
C47H67N3O7	C26H23NO3S3	C10H15NO5	C26H30O2S	C36H17NO2	C14H23NO3S
C10H21N3O7S	C31H31NO3S3	C11H17NO5	C19H14O2S	C43H25NO2	C12H19NO3S
C11H23N3O7S	C24H13NO3S3	C12H19NO5	C22H18O2S	C41H19NO2	C21H29NO3S
C9H19N3O7S	C35H33NO3S3	C13H21NO5	C39H40O2S	C45H27NO2	C25H35NO3S
C24H39N3O7S	C20H41NO4	C14H23NO5	C41H42O2S	C5H11NO2S	C47H79NO3S
C17H15N3O7S	C8H17NO4	C20H35NO5	C9H14O2S2	C13H23NO2S	C17H17NO3S
C14H25N3O7S2	C9H19NO4	C8H11NO5	C11H10O2S2	C16H17NO2S	C25H33NO3S
C14H23N3O7S2	C10H19NO4	C9H13NO5	C12H12O2S2	C21H25NO2S	C27H37NO3S
C17H27N3O7S2	C11H21NO4	C13H19NO5	C13H14O2S2	C24H25NO2S	C26H33NO3S
C29H47N3O7S2	C20H39NO4	C14H21NO5	C14H16O2S2	C26H29NO2S	C17H15NO3S
C24H51N3O8	C22H43NO4	C10H13NO5	C13H12O2S2	C38H51NO2S	C25H29NO3S
C13H27N3O8	C9H17NO4	C11H15NO5	C24H32O2S2	C40H77NO2S2	C26H31NO3S
C16H31N3O8	C31H61NO4	C12H17NO5	C22H24O2S2	C25H31NO2S2	C27H33NO3S
C23H37N3O8	C10H17NO4	C19H31NO5	C23H26O2S2	C19H17NO2S2	C26H29NO3S
C23H35N3O8	C11H19NO4	C9H11NO5	C25H18O2S2	C35H31NO2S2	C27H29NO3S
C31H29N3O8	C12H21NO4	C13H17NO5	C33H18O2S2	C50H55NO2S2	C27H27NO3S
C35H15N3O8	C13H23NO4	C14H19NO5	C12H26O2S3	C37H21NO2S2	C30H57NO3S2
C13H23N3O8S	C14H25NO4	C15H21NO5	C13H28O2S3	C36H13NO2S2	C18H29NO3S2
C33H67N3O8S2	C20H37NO4	C10H11NO5	C14H30O2S3	C17H35NO2S3	C30H53NO3S2
C18H13N3O8S2	C9H15NO4	C11H13NO5	C16H34O2S3	C16H19NO2S3	C16H23NO3S2
C12H23N3O9	C18H33NO4	C12H15NO5	C17H36O2S3	C17H21NO2S3	C26H43NO3S2

C22H43N3O9	C10H15NO4	C16H23NO5	C12H24O2S3	C18H23NO2S3	C26H39NO3S2
C22H41N3O9	C11H17NO4	C12H13NO5	C15H30O2S3	C20H27NO2S3	C20H25NO3S2
C18H31N3O9	C12H19NO4	C13H15NO5	C16H32O2S3	C32H47NO2S3	C43H71NO3S2
C18H29N3O9	C13H21NO4	C16H21NO5	C20H40O2S3	C33H43NO2S3	C32H41NO3S2
C18H27N3O9	C14H23NO4	C11H11NO5	C14H26O2S3	C30H31NO2S3	C42H43NO3S2
C19H29N3O9	C15H25NO4	C14H17NO5	C16H30O2S3	C41H39NO2S3	C13H27NO3S3
C18H25N3O9	C19H33NO4	C15H19NO5	C17H32O2S3	C49H41NO2S3	C19H37NO3S3
C29H39N3O9	C31H57NO4	C17H23NO5	C18H34O2S3	C18H39NO3	C13H23NO3S3
C45H67N3O9	C8H11NO4	C47H81NO5	C13H22O2S3	C13H27NO3	C44H85NO3S3
C45H63N3O9	C9H13NO4	C13H13NO5	C16H12O2S3	C16H33NO3	C19H33NO3S3
C45H59N3O9	C10H13NO4	C14H15NO5	C22H18O2S3	C17H35NO3	C18H23NO3S3
C45H55N3O9	C11H15NO4	C15H17NO5	C33H18O2S3	C18H37NO3	C26H23NO3S3
C39H25N3O9	C12H17NO4	C16H19NO5	C11H24O3	C22H45NO3	C38H13NO3S3
C27H51N3O9S2	C13H19NO4	C17H21NO5	C12H26O3	C24H49NO3	C20H41NO4
C39H39N3O9S2	C14H21NO4	C18H23NO5	C13H28O3	C12H23NO3	C22H45NO4
C16H35N3O9S3	C15H23NO4	C13H11NO5	C13H26O3	C20H39NO3	C6H13NO4
C32H31N3OS	C16H25NO4	C14H13NO5	C14H28O3	C22H43NO3	C7H15NO4
C25H15N3OS	C19H31NO4	C15H15NO5	C17H34O3	C7H13NO3	C8H17NO4
C31H67N3OS2	C9H11NO4	C16H17NO5	C19H38O3	C8H15NO3	C9H19NO4
C31H65N3OS2	C12H15NO4	C17H19NO5	C20H40O3	C9H17NO3	C10H19NO4
C35H71N3OS2	C13H17NO4	C18H21NO5	C22H44O3	C11H21NO3	C11H21NO4
C7H13N3OS2	C14H19NO4	C15H13NO5	C10H20O3	C10H17NO3	C20H39NO4
C7H11N3OS2	C10H11NO4	C16H15NO5	C11H22O3	C22H41NO3	C22H43NO4
C23H39N3OS2	C11H13NO4	C19H21NO5	C12H24O3	C44H85NO3	C24H47NO4
C25H35N3OS2	C15H21NO4	C21H23NO5	C15H30O3	C7H11NO3	C6H11NO4
C25H33N3OS2	C16H23NO4	C19H19NO5	C5H10O3	C11H17NO3	C7H13NO4
C24H27N3OS2	C14H17NO4	C28H35NO5	C6H12O3	C22H39NO3	C9H17NO4
C28H33N3OS2	C15H19NO4	C17H13NO5	C7H14O3	C10H13NO3	C13H25NO4
C26H27N3OS2	C16H21NO4	C18H15NO5	C8H16O3	C11H15NO3	C31H61NO4
C14H25N3OS3	C27H43NO4	C32H41NO5	C13H24O3	C12H17NO3	C8H15NO4
C28H49N3OS3	C11H11NO4	C18H11NO5	C15H28O3	C13H19NO3	C10H17NO4
C24H37N3OS3	C12H13NO4	C19H13NO5	C21H40O3	C9H11NO3	C11H19NO4
C25H33N3OS3	C13H15NO4	C20H15NO5	C22H42O3	C13H17NO3	C12H21NO4
C16H13N3OS3	C37H61NO4	C21H17NO5	C6H10O3	C10H11NO3	C13H23NO4
C25H29N3OS3	C12H11NO4	C22H19NO5	C14H26O3	C11H13NO3	C14H25NO4
C22H17N3OS3	C13H13NO4	C19H11NO5	C20H38O3	C25H39NO3	C15H27NO4
C10H17NO	C14H15NO4	C20H13NO5	C7H12O3	C14H13NO3	C16H29NO4
C22H41NO	C15H17NO4	C32H37NO5	C8H14O3	C48H71NO3	C22H41NO4
C13H21NO	C16H19NO4	C48H69NO5	C9H16O3	C26H25NO3	C9H15NO4
C14H13NO	C13H11NO4	C20H11NO5	C15H26O3	C23H17NO3	C7H11NO4
C27H37NO	C14H13NO4	C21H13NO5	C17H30O3	C24H19NO3	C8H13NO4
C25H29NO	C15H15NO4	C26H23NO5	C21H38O3	C25H21NO3	C10H15NO4
C19H11NO	C16H17NO4	C28H27NO5	C22H40O3	C38H41NO3	C11H17NO4
C38H49NO	C17H19NO4	C22H15NO5	C11H18O3	C31H19NO3	C12H19NO4

C36H39NO	C14H11NO4	C26H21NO5	C16H28O3	C32H21NO3	C13H21NO4
C36H37NO	C15H13NO4	C48H65NO5	C20H36O3	C50H35NO3	C14H23NO4
C36H35NO	C17H17NO4	C28H25NO5	C7H10O3	C50H29NO3	C15H25NO4
C29H17NO	C18H19NO4	C31H27NO5	C8H12O3	C5H11NO3S	C8H11NO4
C44H31NO	C18H13NO4	C34H33NO5	C9H14O3	C9H19NO3S	C9H13NO4
C47H33NO	C20H13NO4	C48H61NO5	C11H16O3	C15H23NO3S	C12H17NO4
C17H29NO10	C23H19NO4	C48H57NO5	C12H18O3	C17H17NO3S	C13H19NO4
C28H51NO10	C24H21NO4	C30H11NO5	C22H38O3	C21H23NO3S	C14H21NO4
C39H73NO10	C26H25NO4	C39H21NO5	C8H10O3	C17H15NO3S	C15H23NO4
C17H25NO10	C32H31NO4	C40H17NO5	C10H14O3	C18H17NO3S	C16H25NO4
C27H45NO10	C32H29NO4	C40H15NO5	C13H20O3	C22H19NO3S	C10H13NO4
C29H49NO10	C32H27NO4	C21H35NO5S	C15H24O3	C45H73NO3S2	C11H15NO4
C25H39NO10	C29H19NO4	C22H37NO5S	C20H34O3	C29H31NO3S2	C9H11NO4
C14H17NO10	C39H33NO4	C21H33NO5S	C9H12O3	C27H21NO3S2	C12H15NO4
C16H21NO10	C36H15NO4	C22H35NO5S	C12H16O3	C36H39NO3S2	C13H17NO4
C19H27NO10	C39H19NO4	C11H13NO5S	C13H18O3	C47H35NO3S2	C14H19NO4
C29H45NO10	C20H41NO4S	C48H65NO5S	C14H20O3	C7H15NO3S3	C37H65NO4
C17H21NO10	C13H19NO4S	C41H33NO5S	C16H24O3	C13H23NO3S3	C10H11NO4
C18H23NO10	C47H87NO4S	C40H73NO5S2	C17H26O3	C17H31NO3S3	C11H13NO4
C19H25NO10	C22H35NO4S	C24H39NO5S2	C18H28O3	C29H51NO3S3	C13H15NO4
C20H27NO10	C12H13NO4S	C24H35NO5S2	C10H12O3	C26H23NO3S3	C14H17NO4
C22H31NO10	C15H19NO4S	C24H27NO5S2	C11H14O3	C23H17NO3S3	C15H19NO4
C25H35NO10	C13H13NO4S	C35H39NO5S2	C15H22O3	C10H19NO4	C16H21NO4
C16H17NO10	C15H17NO4S	C35H27NO5S2	C20H32O3	C22H43NO4	C27H43NO4
C17H19NO10	C18H21NO4S	C40H27NO5S2	C9H10O3	C6H11NO4	C11H11NO4
C18H21NO10	C24H31NO4S	C17H33NO5S3	C10H10O3	C7H13NO4	C12H13NO4
C19H23NO10	C25H33NO4S	C36H51NO5S3	C11H12O3	C8H15NO4	C37H61NO4
C20H25NO10	C25H31NO4S	C12H23NO6	C12H14O3	C9H17NO4	C12H11NO4
C21H27NO10	C26H31NO4S	C13H25NO6	C13H16O3	C31H61NO4	C13H13NO4
C22H29NO10	C26H21NO4S	C21H41NO6	C14H18O3	C10H17NO4	C14H15NO4
C23H31NO10	C43H27NO4S	C12H21NO6	C15H20O3	C11H19NO4	C15H17NO4
C24H33NO10	C10H21NO4S2	C20H37NO6	C16H22O3	C12H21NO4	C16H19NO4
C29H41NO10	C27H49NO4S2	C22H41NO6	C18H26O3	C13H23NO4	C13H11NO4
C17H17NO10	C36H59NO4S2	C10H17NO6	C20H30O3	C14H25NO4	C14H13NO4
C18H19NO10	C15H15NO4S2	C13H21NO6	C15H18O3	C22H41NO4	C15H15NO4
C19H21NO10	C29H37NO4S2	C15H25NO6	C16H20O3	C8H13NO4	C16H17NO4
C20H23NO10	C25H23NO4S2	C10H15NO6	C23H34O3	C9H15NO4	C17H19NO4
C21H25NO10	C34H41NO4S2	C11H17NO6	C25H38O3	C7H11NO4	C22H27NO4
C22H27NO10	C39H47NO4S2	C12H19NO6	C11H10O3	C11H17NO4	C23H29NO4
C23H29NO10	C48H39NO4S2	C19H31NO6	C12H12O3	C10H15NO4	C15H13NO4
C24H31NO10	C26H51NO4S3	C27H47NO6	C13H14O3	C13H21NO4	C23H27NO4
C25H33NO10	C42H83NO4S3	C10H13NO6	C14H16O3	C8H11NO4	C23H25NO4
C27H35NO10	C18H19NO4S3	C11H15NO6	C17H22O3	C9H13NO4	C18H13NO4
C42H65NO10	C30H39NO4S3	C12H17NO6	C18H24O3	C11H15NO4	C23H23NO4

C17H15NO10	C30H35NO4S3	C13H19NO6	C13H12O3	C12H17NO4	C20H13NO4
C20H21NO10	C40H43NO4S3	C14H21NO6	C19H22O3	C13H19NO4	C23H19NO4
C21H23NO10	C10H21NO5	C15H21NO6	C19H20O3	C14H21NO4	C25H23NO4
C22H25NO10	C11H23NO5	C21H33NO6	C24H26O3	C16H25NO4	C25H21NO4
C23H27NO10	C18H37NO5	C11H13NO6	C25H28O3	C18H29NO4	C32H33NO4
C24H29NO10	C20H41NO5	C12H15NO6	C26H30O3	C19H31NO4	C22H13NO4
C25H31NO10	C22H45NO5	C13H17NO6	C30H38O3	C10H13NO4	C32H31NO4
C19H17NO10	C24H49NO5	C14H19NO6	C31H40O3	C9H11NO4	C33H33NO4
C20H19NO10	C8H17NO5	C16H23NO6	C33H44O3	C13H17NO4	C32H29NO4
C21H21NO10	C9H19NO5	C15H19NO6	C19H14O3	C10H11NO4	C32H27NO4
C22H23NO10	C10H19NO5	C19H27NO6	C23H22O3	C11H13NO4	C32H25NO4
C23H25NO10	C11H21NO5	C11H11NO6	C25H26O3	C12H15NO4	C32H21NO4
C24H27NO10	C12H23NO5	C12H13NO6	C26H28O3	C29H47NO4	C36H15NO4
C25H29NO10	C13H25NO5	C13H15NO6	C27H30O3	C11H11NO4	C43H29NO4
C26H31NO10	C14H27NO5	C14H17NO6	C28H32O3	C12H13NO4	C21H41NO4S
C27H31NO10	C20H39NO5	C16H21NO6	C29H34O3	C13H15NO4	C27H51NO4S
C21H19NO10	C22H43NO5	C17H23NO6	C30H36O3	C14H17NO4	C12H19NO4S
C22H21NO10	C24H47NO5	C12H11NO6	C31H38O3	C15H19NO4	C13H19NO4S
C23H23NO10	C10H17NO5	C13H13NO6	C32H40O3	C37H61NO4	C12H15NO4S
C24H25NO10	C11H19NO5	C14H15NO6	C33H42O3	C12H11NO4	C22H31NO4S
C25H27NO10	C12H21NO5	C15H17NO6	C34H44O3	C13H13NO4	C13H13NO4S
C26H29NO10	C13H23NO5	C16H19NO6	C35H46O3	C16H19NO4	C15H17NO4S
C21H17NO10	C20H37NO5	C17H21NO6	C36H48O3	C14H13NO4	C18H21NO4S
C22H19NO10	C22H41NO5	C18H23NO6	C37H50O3	C15H15NO4	C24H31NO4S
C23H21NO10	C24H45NO5	C19H25NO6	C21H18O3	C22H27NO4	C25H33NO4S
C24H23NO10	C8H13NO5	C31H47NO6	C23H20O3	C25H21NO4	C25H31NO4S
C25H25NO10	C9H15NO5	C13H11NO6	C24H22O3	C24H17NO4	C26H31NO4S
C26H27NO10	C10H15NO5	C14H13NO6	C25H24O3	C28H25NO4	C26H21NO4S
C23H19NO10	C11H17NO5	C15H15NO6	C26H26O3	C32H33NO4	C44H57NO4S
C24H21NO10	C12H19NO5	C16H17NO6	C27H28O3	C32H19NO4	C43H27NO4S
C25H23NO10	C13H21NO5	C17H19NO6	C29H32O3	C38H23NO4	C10H21NO4S2
C27H27NO10	C14H23NO5	C18H21NO6	C31H36O3	C36H17NO4	C40H79NO4S2
C29H31NO10	C18H31NO5	C19H23NO6	C32H38O3	C38H21NO4	C24H43NO4S2
C25H21NO10	C8H11NO5	C14H11NO6	C33H40O3	C39H21NO4	C29H37NO4S2
C42H51NO10	C9H13NO5	C15H13NO6	C34H42O3	C10H17NO4S	C25H23NO4S2
C41H41NO10	C12H17NO5	C16H15NO6	C35H44O3	C13H19NO4S	C39H47NO4S2
C43H45NO10	C13H19NO5	C17H17NO6	C36H46O3	C25H33NO4S	C41H39NO4S2
C39H31NO10	C14H21NO5	C18H19NO6	C37H48O3	C43H67NO4S	C48H39NO4S2
C41H85NO10S	C15H23NO5	C19H21NO6	C38H50O3	C32H31NO4S	C30H39NO4S3
C12H23NO10S	C16H25NO5	C17H15NO6	C39H52O3	C33H29NO4S	C40H43NO4S3
C23H25NO10S	C10H13NO5	C18H17NO6	C45H64O3	C34H31NO4S	C39H31NO4S3
C30H47NO10S2	C11H15NO5	C19H19NO6	C47H68O3	C10H21NO4S2	C35H21NO4S3
C35H39NO10S2	C9H11NO5	C20H21NO6	C23H18O3	C15H31NO4S2	C10H21NO5
C29H19NO10S2	C13H17NO5	C21H23NO6	C24H20O3	C8H17NO4S2	C11H23NO5

C23H43NO10S3	C14H19NO5	C23H25NO6	C25H22O3	C9H19NO4S2	C18H37NO5
C23H39NO10S3	C15H21NO5	C19H17NO6	C27H26O3	C29H49NO4S2	C20H41NO5
C28H45NO10S3	C16H23NO5	C21H21NO6	C29H30O3	C29H33NO4S2	C22H45NO5
C28H39NO10S3	C10H11NO5	C18H11NO6	C31H34O3	C29H25NO4S2	C23H47NO5
C31H37NO10S3	C11H13NO5	C19H13NO6	C33H38O3	C43H37NO4S2	C24H49NO5
C35H39NO10S3	C12H15NO5	C20H15NO6	C34H40O3	C9H19NO4S3	C25H51NO5
C12H25NO11	C17H25NO5	C21H17NO6	C35H42O3	C19H33NO4S3	C27H55NO5
C12H11NO11	C12H13NO5	C24H23NO6	C36H44O3	C17H27NO4S3	C8H17NO5
C13H13NO11	C13H15NO5	C46H67NO6	C37H46O3	C19H23NO4S3	C9H19NO5
C14H15NO11	C14H17NO5	C19H11NO6	C38H48O3	C30H35NO4S3	C11H21NO5
C17H21NO11	C15H19NO5	C20H13NO6	C39H50O3	C39H31NO4S3	C14H27NO5
C22H31NO11	C16H21NO5	C21H15NO6	C40H52O3	C10H21NO5	C16H31NO5
C19H23NO11	C17H23NO5	C22H17NO6	C45H62O3	C6H13NO5	C20H39NO5
C20H25NO11	C11H11NO5	C23H19NO6	C47H66O3	C8H17NO5	C21H41NO5
C21H27NO11	C12H11NO5	C20H11NO6	C24H18O3	C10H19NO5	C22H43NO5
C20H23NO11	C13H13NO5	C23H17NO6	C35H40O3	C11H21NO5	C24H47NO5
C21H25NO11	C14H15NO5	C30H31NO6	C37H44O3	C22H43NO5	C25H49NO5
C22H27NO11	C15H17NO5	C28H27NO6	C23H14O3	C8H15NO5	C9H17NO5
C24H31NO11	C16H19NO5	C21H11NO6	C24H16O3	C9H17NO5	C10H17NO5
C19H19NO11	C17H21NO5	C28H25NO6	C25H18O3	C6H11NO5	C11H19NO5
C20H21NO11	C18H23NO5	C30H29NO6	C26H20O3	C7H13NO5	C12H21NO5
C21H23NO11	C13H11NO5	C30H27NO6	C47H62O3	C10H17NO5	C13H23NO5
C22H25NO11	C14H13NO5	C30H25NO6	C28H24O3	C11H19NO5	C14H25NO5
C23H27NO11	C15H15NO5	C39H31NO6	C27H20O3	C12H21NO5	C15H27NO5
C24H29NO11	C16H17NO5	C31H11NO6	C35H22O3	C13H23NO5	C20H37NO5
C25H31NO11	C17H19NO5	C9H17NO6S	C12H26O3S	C8H13NO5	C22H41NO5
C22H23NO11	C18H21NO5	C20H33NO6S	C8H18O3S	C9H15NO5	C24H45NO5
C23H25NO11	C19H23NO5	C20H31NO6S	C10H20O3S	C7H11NO5	C25H47NO5
C24H27NO11	C15H13NO5	C21H33NO6S	C5H10O3S	C10H15NO5	C8H13NO5
C25H29NO11	C16H15NO5	C15H21NO6S	C6H12O3S	C11H17NO5	C9H15NO5
C26H31NO11	C17H17NO5	C20H29NO6S	C7H14O3S	C9H13NO5	C10H15NO5
C27H33NO11	C18H19NO5	C22H45NO6S2	C8H16O3S	C8H11NO5	C11H17NO5
C22H21NO11	C19H21NO5	C19H33NO6S2	C18H34O3S	C10H13NO5	C12H19NO5
C23H23NO11	C20H23NO5	C25H45NO6S2	C19H34O3S	C11H15NO5	C13H21NO5
C24H25NO11	C15H11NO5	C21H33NO6S2	C20H36O3S	C12H17NO5	C14H23NO5
C25H27NO11	C16H13NO5	C29H19NO6S2	C22H40O3S	C13H19NO5	C15H25NO5
C26H29NO11	C17H15NO5	C33H21NO6S2	C29H54O3S	C9H11NO5	C8H11NO5
C27H31NO11	C18H17NO5	C12H25NO6S3	C18H32O3S	C10H11NO5	C9H13NO5
C23H21NO11	C19H19NO5	C19H39NO6S3	C10H14O3S	C11H13NO5	C13H19NO5
C24H23NO11	C20H21NO5	C35H39NO6S3	C19H32O3S	C12H15NO5	C14H21NO5
C25H25NO11	C22H25NO5	C13H27NO7	C28H50O3S	C13H17NO5	C15H23NO5
C26H27NO11	C28H35NO5	C18H37NO7	C8H10O3S	C15H21NO5	C16H25NO5
C27H29NO11	C17H13NO5	C13H21NO7	C9H12O3S	C11H11NO5	C10H13NO5
C28H31NO11	C18H15NO5	C16H27NO7	C15H24O3S	C12H13NO5	C11H15NO5

C24H21NO11	C28H33NO5	C18H31NO7	C20H34O3S	C13H15NO5	C12H17NO5
C25H23NO11	C32H41NO5	C11H17NO7	C11H14O3S	C14H17NO5	C9H11NO5
C26H25NO11	C32H37NO5	C15H23NO7	C18H28O3S	C15H19NO5	C10H11NO5
C27H27NO11	C48H69NO5	C11H15NO7	C19H30O3S	C16H21NO5	C13H17NO5
C26H23NO11	C22H15NO5	C13H19NO7	C9H10O3S	C12H11NO5	C14H19NO5
C41H47NO11	C28H27NO5	C14H21NO7	C17H20O3S	C13H13NO5	C15H21NO5
C13H21NO11S	C48H65NO5	C16H25NO7	C31H46O3S	C14H15NO5	C16H23NO5
C24H41NO11S	C46H59NO5	C12H15NO7	C15H14O3S	C15H17NO5	C11H13NO5
C24H37NO11S	C31H27NO5	C13H17NO7	C16H16O3S	C16H19NO5	C12H15NO5
C22H45NO11S3	C48H61NO5	C14H19NO7	C18H20O3S	C14H13NO5	C17H25NO5
C12H15NO11S3	C46H55NO5	C15H21NO7	C20H24O3S	C16H17NO5	C12H13NO5
C16H19NO11S3	C48H57NO5	C16H23NO7	C21H26O3S	C17H19NO5	C16H21NO5
C27H17NO11S3	C46H51NO5	C12H13NO7	C27H38O3S	C19H19NO5	C11H11NO5
C27H49NO12	C38H25NO5	C13H15NO7	C29H42O3S	C20H21NO5	C13H15NO5
C25H41NO12	C39H21NO5	C14H17NO7	C30H44O3S	C21H23NO5	C14H17NO5
C39H69NO12	C40H15NO5	C15H19NO7	C32H48O3S	C19H17NO5	C15H19NO5
C23H35NO12	C11H13NO5S	C16H21NO7	C31H44O3S	C17H11NO5	C17H23NO5
C27H41NO12	C12H15NO5S	C17H23NO7	C21H24O3S	C22H17NO5	C18H25NO5
C15H17NO12	C15H21NO5S	C18H25NO7	C27H36O3S	C48H69NO5	C12H11NO5
C28H43NO12	C13H13NO5S	C19H27NO7	C29H40O3S	C24H19NO5	C13H13NO5
C37H61NO12	C23H29NO5S	C20H29NO7	C17H14O3S	C22H15NO5	C14H15NO5
C39H65NO12	C25H33NO5S	C13H13NO7	C18H16O3S	C48H65NO5	C15H17NO5
C23H31NO12	C25H25NO5S	C14H15NO7	C23H26O3S	C31H19NO5	C16H19NO5
C27H37NO12	C48H65NO5S	C15H17NO7	C19H38O3S2	C34H23NO5	C17H21NO5
C26H33NO12	C16H29NO5S2	C16H19NO7	C6H12O3S2	C34H21NO5	C18H23NO5
C27H33NO12	C40H77NO5S2	C17H21NO7	C16H30O3S2	C46H31NO5	C19H25NO5
C21H21NO12	C24H39NO5S2	C18H23NO7	C8H14O3S2	C39H15NO5	C13H11NO5
C23H25NO12	C24H35NO5S2	C19H25NO7	C9H14O3S2	C12H25NO5S	C14H13NO5
C24H27NO12	C24H31NO5S2	C20H27NO7	C12H18O3S2	C7H13NO5S	C15H15NO5
C25H29NO12	C28H37NO5S2	C14H13NO7	C13H14O3S2	C8H15NO5S	C16H17NO5
C24H25NO12	C35H39NO5S2	C15H15NO7	C12H10O3S2	C10H17NO5S	C17H19NO5
C25H27NO12	C35H35NO5S2	C16H17NO7	C15H16O3S2	C9H13NO5S	C18H21NO5
C26H23NO12	C35H27NO5S2	C17H19NO7	C24H34O3S2	C21H33NO5S	C15H13NO5
C28H27NO12	C38H33NO5S2	C18H21NO7	C16H16O3S2	C17H19NO5S	C16H15NO5
C30H31NO12	C38H31NO5S2	C19H23NO7	C17H14O3S2	C25H17NO5S	C17H17NO5
C30H29NO12	C40H35NO5S2	C20H25NO7	C23H26O3S2	C19H23NO5S2	C18H19NO5
C36H37NO12	C17H33NO5S3	C16H15NO7	C19H14O3S2	C22H25NO5S2	C19H21NO5
C38H37NO12	C17H29NO5S3	C17H17NO7	C24H14O3S2	C29H33NO5S2	C18H17NO5
C18H35NO12S	C15H17NO5S3	C18H19NO7	C35H32O3S2	C26H23NO5S2	C19H19NO5
C32H17NO12S	C17H13NO5S3	C19H21NO7	C37H36O3S2	C26H17NO5S2	C28H35NO5
C26H37NO12S3	C12H23NO6	C20H23NO7	C37H34O3S2	C34H25NO5S2	C32H41NO5
C31H65NO13	C13H25NO6	C21H25NO7	C10H22O3S3	C36H71NO5S3	C32H37NO5
C37H75NO13	C21H41NO6	C16H13NO7	C11H24O3S3	C10H19NO5S3	C48H69NO5
C12H21NO13	C22H43NO6	C17H15NO7	C13H28O3S3	C19H25NO5S3	C22H15NO5

C35H65NO13	C12H21NO6	C18H17NO7	C14H30O3S3	C20H27NO5S3	C28H27NO5
C31H51NO13	C20H37NO6	C19H19NO7	C12H26O3S3	C10H21NO6	C48H65NO5
C26H29NO13	C22H41NO6	C20H21NO7	C8H16O3S3	C11H23NO6	C32H31NO5
C39H39NO13	C14H23NO6	C21H23NO7	C11H20O3S3	C8H17NO6	C31H27NO5
C15H25NO14	C15H25NO6	C18H15NO7	C13H24O3S3	C10H19NO6	C48H61NO5
C25H37NO14	C21H37NO6	C19H17NO7	C15H28O3S3	C11H21NO6	C46H55NO5
C32H29NO14	C11H17NO6	C20H19NO7	C23H44O3S3	C21H41NO6	C48H57NO5
C30H19NO14	C12H19NO6	C21H21NO7	C30H56O3S3	C8H15NO6	C40H37NO5
C36H29NO14	C13H21NO6	C30H37NO7	C13H18O3S3	C7H13NO6	C40H15NO5
C34H41NO15	C9H13NO6	C21H19NO7	C16H14O3S3	C10H17NO6	C10H21NO5S
C37H37NO15	C19H31NO6	C19H11NO7	C11H24O4	C11H19NO6	C12H25NO5S
C28H45NO16	C27H47NO6	C20H13NO7	C12H26O4	C12H21NO6	C22H43NO5S
C28H39NO16	C39H71NO6	C21H15NO7	C14H30O4	C17H31NO6	C20H37NO5S
C28H35NO16	C11H15NO6	C22H17NO7	C22H46O4	C8H13NO6	C11H13NO5S
C30H59NO16S3	C12H17NO6	C23H19NO7	C15H30O4	C21H39NO6	C14H19NO5S
C17H33NO17	C13H19NO6	C24H21NO7	C16H32O4	C7H11NO6	C25H33NO5S
C19H35NO17	C14H21NO6	C25H23NO7	C22H44O4	C9H15NO6	C48H65NO5S
C18H33NO17	C15H23NO6	C46H65NO7	C24H48O4	C10H15NO6	C28H55NO5S2
C32H37NO17	C15H21NO6	C20H11NO7	C10H20O4	C11H17NO6	C24H45NO5S2
C23H39NO18	C21H33NO6	C21H13NO7	C12H24O4	C12H19NO6	C40H73NO5S2
C26H31NO18	C10H11NO6	C22H15NO7	C13H26O4	C13H21NO6	C24H39NO5S2
C8H17NO2	C11H13NO6	C23H17NO7	C14H28O4	C8H11NO6	C24H35NO5S2
C11H21NO2	C12H15NO6	C44H55NO7	C17H34O4	C9H13NO6	C35H39NO5S2
C13H25NO2	C13H17NO6	C44H53NO7	C19H38O4	C14H21NO6	C35H27NO5S2
C22H43NO2	C14H19NO6	C25H13NO7	C20H40O4	C15H23NO6	C17H35NO6
C6H11NO2	C16H23NO6	C34H27NO7	C5H10O4	C10H13NO6	C12H23NO6
C13H23NO2	C17H25NO6	C48H29NO7	C6H12O4	C11H15NO6	C21H41NO6
C14H25NO2	C18H27NO6	C12H19NO7S	C7H14O4	C12H17NO6	C22H43NO6
C15H27NO2	C15H19NO6	C47H35NO7S	C8H16O4	C13H19NO6	C8H15NO6
C22H41NO2	C16H21NO6	C22H35NO7S2	C9H18O4	C9H11NO6	C12H21NO6
C7H11NO2	C17H23NO6	C26H41NO7S2	C21H40O4	C20H31NO6	C20H37NO6
C10H15NO2	C19H27NO6	C18H21NO7S2	C23H44O4	C21H33NO6	C22H41NO6
C11H17NO2	C11H11NO6	C25H31NO7S2	C24H46O4	C22H35NO6	C10H17NO6
C12H19NO2	C12H13NO6	C36H23NO7S2	C25H48O4	C10H11NO6	C13H21NO6
C13H21NO2	C13H15NO6	C20H39NO7S3	C26H50O4	C11H13NO6	C14H23NO6
C33H61NO2	C14H17NO6	C20H37NO7S3	C38H74O4	C12H15NO6	C15H25NO6
C9H13NO2	C18H25NO6	C26H43NO7S3	C15H28O4	C13H17NO6	C16H27NO6
C14H21NO2	C12H11NO6	C27H45NO7S3	C17H32O4	C14H19NO6	C17H29NO6
C9H11NO2	C13H13NO6	C18H13NO7S3	C19H36O4	C15H21NO6	C10H15NO6
C10H13NO2	C14H15NO6	C21H19NO7S3	C20H38O4	C21H31NO6	C11H17NO6
C10H11NO2	C15H17NO6	C28H21NO7S3	C22H42O4	C11H11NO6	C12H19NO6
C11H13NO2	C16H19NO6	C19H33NO8	C27H52O4	C12H13NO6	C8H11NO6
C12H15NO2	C17H21NO6	C31H57NO8	C6H10O4	C13H15NO6	C9H13NO6
C15H13NO2	C18H23NO6	C11H17NO8	C7H12O4	C14H17NO6	C14H21NO6

C28H33NO2	C19H25NO6	C19H29NO8	C20H36O4	C15H19NO6	C16H25NO6
C20H15NO2	C20H27NO6	C14H19NO8	C21H38O4	C16H21NO6	C17H27NO6
C26H25NO2	C31H47NO6	C17H23NO8	C22H40O4	C12H11NO6	C19H31NO6
C38H47NO2	C13H11NO6	C27H43NO8	C23H42O4	C14H15NO6	C27H47NO6
C39H49NO2	C14H13NO6	C14H17NO8	C24H44O4	C15H17NO6	C10H13NO6
C39H47NO2	C15H15NO6	C15H19NO8	C25H46O4	C16H19NO6	C11H15NO6
C25H17NO2	C16H17NO6	C16H21NO8	C36H68O4	C17H21NO6	C12H17NO6
C35H33NO2	C17H19NO6	C18H25NO8	C11H18O4	C14H13NO6	C13H19NO6
C43H39NO2	C18H21NO6	C19H27NO8	C17H30O4	C15H15NO6	C15H23NO6
C37H17NO2	C19H23NO6	C21H29NO8	C19H34O4	C16H17NO6	C15H21NO6
C27H43NO20	C20H25NO6	C14H15NO8	C7H10O4	C17H19NO6	C16H23NO6
C16H33NO2S	C21H27NO6	C15H17NO8	C8H12O4	C18H21NO6	C10H11NO6
C27H31NO2S	C15H13NO6	C16H19NO8	C9H14O4	C22H23NO6	C11H13NO6
C24H21NO2S	C16H15NO6	C17H21NO8	C11H16O4	C22H19NO6	C12H15NO6
C28H29NO2S	C17H17NO6	C18H23NO8	C22H38O4	C23H19NO6	C13H17NO6
C8H17NO2S2	C18H19NO6	C19H25NO8	C23H40O4	C30H27NO6	C14H19NO6
C11H19NO2S2	C19H21NO6	C20H27NO8	C24H42O4	C31H29NO6	C17H25NO6
C27H31NO2S2	C20H23NO6	C42H71NO8	C8H10O4	C25H11NO6	C18H27NO6
C45H65NO2S2	C15H11NO6	C17H19NO8	C9H12O4	C38H37NO6	C15H19NO6
C40H53NO2S2	C16H13NO6	C29H43NO8	C10H14O4	C34H23NO6	C27H43NO6
C43H55NO2S2	C17H15NO6	C39H63NO8	C12H18O4	C35H25NO6	C11H11NO6
C45H57NO2S2	C18H17NO6	C15H15NO8	C15H24O4	C36H27NO6	C12H13NO6
C35H31NO2S2	C19H19NO6	C16H17NO8	C16H26O4	C9H19NO6S2	C13H15NO6
C43H47NO2S2	C20H21NO6	C18H21NO8	C17H28O4	C22H41NO6S2	C14H17NO6
C36H25NO2S2	C21H23NO6	C19H23NO8	C21H36O4	C10H17NO6S2	C16H21NO6
C50H47NO2S2	C22H25NO6	C21H27NO8	C10H12O4	C18H21NO6S2	C17H23NO6
C50H43NO2S2	C23H25NO6	C41H65NO8	C11H14O4	C18H19NO6S2	C18H25NO6
C49H33NO2S2	C46H71NO6	C17H17NO8	C15H22O4	C46H53NO6S2	C19H27NO6
C10H21NO2S3	C17H13NO6	C18H19NO8	C16H24O4	C36H27NO6S2	C20H29NO6
C11H23NO2S3	C18H15NO6	C19H21NO8	C21H34O4	C48H37NO6S2	C12H11NO6
C18H37NO2S3	C19H17NO6	C20H23NO8	C22H36O4	C10H21NO6S3	C13H13NO6
C11H21NO2S3	C20H19NO6	C21H25NO8	C23H38O4	C12H25NO6S3	C14H15NO6
C12H23NO2S3	C21H21NO6	C22H27NO8	C12H16O4	C14H25NO6S3	C15H17NO6
C14H17NO2S3	C18H11NO6	C27H35NO8	C13H18O4	C18H29NO6S3	C16H19NO6
C15H19NO2S3	C19H13NO6	C44H69NO8	C18H28O4	C19H31NO6S3	C17H21NO6
C16H21NO2S3	C21H17NO6	C19H19NO8	C20H32O4	C31H41NO6S3	C18H23NO6
C18H25NO2S3	C46H67NO6	C20H21NO8	C9H10O4	C33H21NO6S3	C19H25NO6
C19H27NO2S3	C19H11NO6	C21H23NO8	C11H12O4	C10H19NO7	C20H27NO6
C32H47NO2S3	C46H63NO6	C22H25NO8	C14H18O4	C11H21NO7	C27H41NO6
C32H43NO2S3	C28H27NO6	C20H19NO8	C17H24O4	C12H23NO7	C13H11NO6
C32H39NO2S3	C30H31NO6	C44H65NO8	C18H26O4	C14H27NO7	C14H13NO6
C32H35NO2S3	C30H29NO6	C21H19NO8	C19H28O4	C15H29NO7	C15H15NO6
C41H39NO2S3	C31H29NO6	C23H23NO8	C20H30O4	C24H47NO7	C16H17NO6
C43H43NO2S3	C28H17NO6	C33H41NO8	C22H34O4	C26H51NO7	C17H19NO6

C41H35NO2S3	C39H31NO6	C21H13NO8	C10H10O4	C28H55NO7	C18H21NO6
C32H17NO2S3	C31H11NO6	C22H15NO8	C12H14O4	C30H59NO7	C19H23NO6
C22H45NO3	C38H21NO6	C23H17NO8	C13H16O4	C8H15NO7	C20H25NO6
C12H23NO3	C16H31NO6S	C41H39NO8	C15H20O4	C9H17NO7	C14H11NO6
C14H27NO3	C6H11NO6S	C42H37NO8	C16H22O4	C10H17NO7	C15H13NO6
C15H29NO3	C22H41NO6S	C43H39NO8	C27H44O4	C11H19NO7	C16H15NO6
C22H43NO3	C20H21NO6S	C31H13NO8	C12H12O4	C14H25NO7	C17H17NO6
C24H47NO3	C22H45NO6S2	C36H15NO8	C13H14O4	C16H29NO7	C18H19NO6
C7H13NO3	C21H33NO6S2	C14H27NO8S	C14H16O4	C17H31NO7	C19H21NO6
C10H19NO3	C29H19NO6S2	C20H37NO8S	C15H18O4	C9H15NO7	C20H23NO6
C11H21NO3	C32H25NO6S2	C32H61NO8S	C19H26O4	C21H39NO7	C21H25NO6
C8H15NO3	C10H21NO6S3	C20H33NO8S	C23H34O4	C22H41NO7	C17H15NO6
C9H17NO3	C12H25NO6S3	C20H15NO8S	C25H38O4	C12H19NO7	C18H17NO6
C10H17NO3	C19H35NO6S3	C23H21NO8S	C42H72O4	C10H15NO7	C19H19NO6
C11H19NO3	C27H51NO6S3	C34H37NO8S	C11H10O4	C11H17NO7	C20H21NO6
C12H21NO3	C23H41NO6S3	C17H35NO8S3	C16H20O4	C13H21NO7	C21H23NO6
C14H25NO3	C30H61NO7	C25H51NO8S3	C17H22O4	C14H23NO7	C23H25NO6
C15H27NO3	C14H27NO7	C33H29NO8S3	C18H24O4	C8H11NO7	C46H71NO6
C7H11NO3	C16H27NO7	C31H21NO8S3	C20H28O4	C9H13NO7	C18H11NO6
C8H13NO3	C15H23NO7	C14H29NO9	C17H20O4	C11H15NO7	C19H13NO6
C9H15NO3	C18H29NO7	C14H25NO9	C20H26O4	C15H23NO7	C21H17NO6
C11H17NO3	C11H15NO7	C18H25NO9	C23H32O4	C10H13NO7	C46H67NO6
C12H19NO3	C12H17NO7	C17H21NO9	C25H36O4	C12H17NO7	C19H11NO6
C13H21NO3	C13H19NO7	C18H23NO9	C12H10O4	C13H19NO7	C20H13NO6
C10H15NO3	C14H21NO7	C20H27NO9	C13H12O4	C14H21NO7	C46H63NO6
C8H11NO3	C16H25NO7	C23H33NO9	C14H14O4	C9H11NO7	C28H27NO6
C9H13NO3	C11H13NO7	C15H15NO9	C15H16O4	C11H13NO7	C30H31NO6
C10H13NO3	C12H15NO7	C17H19NO9	C16H18O4	C12H15NO7	C32H35NO6
C11H15NO3	C13H17NO7	C21H27NO9	C18H20O4	C13H17NO7	C30H29NO6
C12H17NO3	C14H19NO7	C22H29NO9	C15H14O4	C14H19NO7	C22H45NO6S2
C13H19NO3	C15H21NO7	C23H31NO9	C18H18O4	C15H21NO7	C29H19NO6S2
C9H11NO3	C16H23NO7	C20H23NO9	C16H14O4	C17H25NO7	C18H37NO7
C11H13NO3	C17H25NO7	C23H29NO9	C18H16O4	C39H67NO7	C17H33NO7
C12H15NO3	C18H27NO7	C19H19NO9	C22H22O4	C12H13NO7	C18H35NO7
C13H17NO3	C17H23NO7	C21H23NO9	C23H24O4	C13H15NO7	C19H37NO7
C14H19NO3	C12H13NO7	C21H21NO9	C25H28O4	C14H17NO7	C22H43NO7
C10H11NO3	C13H15NO7	C22H23NO9	C30H38O4	C15H19NO7	C24H47NO7
C13H15NO3	C14H17NO7	C23H25NO9	C23H22O4	C23H33NO7	C25H49NO7
C11H11NO3	C15H19NO7	C24H27NO9	C24H24O4	C13H13NO7	C15H27NO7
C12H13NO3	C16H21NO7	C30H37NO9	C25H26O4	C14H15NO7	C17H31NO7
C14H17NO3	C18H25NO7	C23H21NO9	C26H28O4	C16H19NO7	C14H23NO7
C12H11NO3	C19H27NO7	C25H23NO9	C27H30O4	C17H21NO7	C15H25NO7
C14H15NO3	C12H11NO7	C28H23NO9	C28H32O4	C18H23NO7	C16H27NO7
C15H17NO3	C13H13NO7	C42H43NO9	C29H34O4	C14H13NO7	C17H29NO7

C13H11NO3	C14H15NO7	C42H39NO9	C30H36O4	C15H15NO7	C18H31NO7
C14H13NO3	C15H17NO7	C32H13NO9	C31H38O4	C17H19NO7	C11H17NO7
C15H11NO3	C16H19NO7	C34H17NO9	C32H40O4	C22H27NO7	C12H19NO7
C17H13NO3	C17H21NO7	C18H27NO9S2	C33H42O4	C20H13NO7	C13H21NO7
C30H39NO3	C18H23NO7	C31H23NO9S2	C34H44O4	C22H17NO7	C15H23NO7
C20H17NO3	C19H25NO7	C39H71NO9S3	C35H46O4	C29H25NO7	C11H15NO7
C34H45NO3	C20H27NO7	C27H41NO9S3	C36H48O4	C25H17NO7	C12H17NO7
C24H23NO3	C21H29NO7	C11H17NOS	C37H50O4	C32H29NO7	C13H19NO7
C26H25NO3	C14H13NO7	C29H45NOS	C19H12O4	C28H17NO7	C14H21NO7
C26H23NO3	C39H63NO7	C29H37NOS	C21H16O4	C35H27NO7	C16H25NO7
C26H21NO3	C13H11NO7	C17H11NOS	C22H18O4	C39H33NO7	C11H13NO7
C26H19NO3	C15H15NO7	C22H19NOS	C23H20O4	C45H41NO7	C12H15NO7
C33H33NO3	C16H17NO7	C36H43NOS	C26H26O4	C38H21NO7	C13H17NO7
C37H41NO3	C17H19NO7	C37H33NOS	C27H28O4	C38H19NO7	C14H19NO7
C33H31NO3	C18H21NO7	C12H27NOS2	C28H30O4	C46H29NO7	C15H21NO7
C34H33NO3	C19H23NO7	C18H33NOS2	C29H32O4	C32H63NO7S	C16H23NO7
C33H29NO3	C20H25NO7	C13H21NOS2	C30H34O4	C19H31NO7S	C17H25NO7
C34H31NO3	C21H27NO7	C28H47NOS2	C31H36O4	C21H31NO7S	C18H27NO7
C35H33NO3	C15H13NO7	C40H71NOS2	C32H38O4	C15H29NO7S2	C39H67NO7
C48H59NO3	C16H15NO7	C22H33NOS2	C33H40O4	C17H23NO7S2	C12H13NO7
C33H27NO3	C17H17NO7	C30H43NOS2	C34H42O4	C12H27NO7S3	C13H15NO7
C36H29NO3	C18H19NO7	C22H19NOS2	C35H44O4	C12H25NO7S3	C14H17NO7
C44H35NO3	C19H21NO7	C37H21NOS2	C36H46O4	C13H25NO7S3	C15H19NO7
C42H23NO3	C20H23NO7	C10H21NOS3	C37H48O4	C20H39NO7S3	C16H21NO7
C42H19NO3	C21H25NO7	C15H31NOS3	C38H50O4	C20H37NO7S3	C17H23NO7
C8H15NO3S	C16H13NO7	C7H15NOS3	C39H52O4	C21H39NO7S3	C18H25NO7
C8H13NO3S	C17H15NO7	C8H17NOS3	C24H22O4	C22H29NO7S3	C19H27NO7
C17H21NO3S	C18H17NO7	C9H19NOS3	C22H16O4	C18H13NO7S3	C20H29NO7
C19H25NO3S	C19H19NO7	C21H41NOS3	C23H18O4	C10H21NO8	C13H13NO7
C27H41NO3S	C20H21NO7	C15H27NOS3	C24H20O4	C11H23NO8	C14H15NO7
C17H19NO3S	C21H23NO7	C13H21NOS3	C25H22O4	C12H25NO8	C15H17NO7
C25H35NO3S	C22H25NO7	C33H61NOS3	C26H24O4	C13H27NO8	C16H19NO7
C19H21NO3S	C17H13NO7	C15H19NOS3	C27H26O4	C14H29NO8	C17H21NO7
C25H33NO3S	C18H15NO7	C44H77NOS3	C28H28O4	C8H17NO8	C18H23NO7
C26H35NO3S	C19H17NO7	C33H27NOS3	C29H30O4	C9H19NO8	C19H25NO7
C27H37NO3S	C20H19NO7	C38H37NOS3	C30H32O4	C10H19NO8	C20H27NO7
C41H65NO3S	C21H21NO7	C48H35NOS3	C31H34O4	C11H21NO8	C39H63NO7
C26H33NO3S	C22H23NO7	C11H22O	C32H36O4	C12H23NO8	C14H13NO7
C27H35NO3S	C30H37NO7	C14H28O	C33H38O4	C13H25NO8	C15H15NO7
C25H29NO3S	C20H17NO7	C15H30O	C34H40O4	C9H17NO8	C16H17NO7
C26H31NO3S	C22H21NO7	C16H32O	C35H42O4	C10H17NO8	C17H19NO7
C27H33NO3S	C20H15NO7	C17H34O	C36H44O4	C11H19NO8	C18H21NO7
C41H61NO3S	C24H23NO7	C8H16O	C37H46O4	C13H23NO8	C19H23NO7
C49H77NO3S	C20H13NO7	C9H18O	C38H48O4	C11H17NO8	C20H25NO7

C26H29NO3S	C22H17NO7	C11H20O	C39H50O4	C13H21NO8	C21H27NO7
C27H29NO3S	C23H19NO7	C15H28O	C21H14O4	C15H25NO8	C22H29NO7
C41H57NO3S	C46H65NO7	C17H32O	C24H18O4	C19H33NO8	C15H13NO7
C26H25NO3S	C28H27NO7	C8H14O	C26H22O4	C31H57NO8	C16H15NO7
C27H27NO3S	C39H49NO7	C9H16O	C32H34O4	C10H15NO8	C17H17NO7
C30H57NO3S2	C25H21NO7	C16H28O	C34H38O4	C12H19NO8	C18H19NO7
C18H29NO3S2	C42H51NO7	C18H32O	C36H42O4	C9H13NO8	C19H21NO7
C30H53NO3S2	C44H55NO7	C10H16O	C37H44O4	C11H15NO8	C20H23NO7
C16H23NO3S2	C25H13NO7	C11H18O	C23H14O4	C12H17NO8	C21H25NO7
C26H43NO3S2	C37H27NO7	C12H20O	C24H16O4	C13H19NO8	C17H15NO7
C26H41NO3S2	C43H19NO7	C13H22O	C25H18O4	C14H21NO8	C18H17NO7
C26H39NO3S2	C49H25NO7	C17H30O	C26H20O4	C15H23NO8	C19H19NO7
C26H35NO3S2	C22H35NO7S2	C8H12O	C27H22O4	C12H15NO8	C20H21NO7
C32H41NO3S2	C18H21NO7S2	C9H14O	C28H24O4	C13H17NO8	C21H23NO7
C19H37NO3S3	C21H27NO7S2	C14H22O	C30H28O4	C14H19NO8	C22H25NO7
C13H23NO3S3	C33H31NO7S2	C15H24O	C24H14O4	C27H43NO8	C18H15NO7
C19H33NO3S3	C34H21NO7S2	C10H14O	C27H20O4	C13H15NO8	C20H19NO7
C18H23NO3S3	C13H25NO7S3	C11H16O	C9H20O4S	C14H17NO8	C21H21NO7
C38H59NO3S3	C20H39NO7S3	C12H18O	C13H28O4S	C15H19NO8	C30H37NO7
C26H23NO3S3	C20H37NO7S3	C16H26O	C16H34O4S	C16H21NO8	C20H17NO7
C31H31NO3S3	C32H59NO7S3	C17H28O	C8H18O4S	C17H23NO8	C20H13NO7
C10H21NO4	C22H35NO7S3	C8H10O	C19H38O4S	C18H25NO8	C22H17NO7
C13H27NO4	C18H13NO7S3	C9H12O	C21H42O4S	C13H13NO8	C23H19NO7
C20H41NO4	C41H81NO8	C10H12O	C6H12O4S	C16H19NO8	C21H13NO7
C6H13NO4	C19H33NO8	C12H16O	C7H14O4S	C17H21NO8	C28H27NO7
C8H17NO4	C31H57NO8	C13H18O	C9H18O4S	C29H43NO8	C22H15NO7
C9H19NO4	C11H17NO8	C18H28O	C10H20O4S	C16H17NO8	C25H13NO7
C10H19NO4	C35H65NO8	C9H10O	C11H22O4S	C17H19NO8	C34H27NO7
C11H21NO4	C39H71NO8	C16H24O	C18H36O4S	C18H21NO8	C22H35NO7S2
C13H25NO4	C19H29NO8	C17H26O	C5H10O4S	C18H19NO8	C18H21NO7S2
C14H27NO4	C14H19NO8	C10H10O	C8H16O4S	C22H25NO8	C21H27NO7S2
C16H31NO4	C15H21NO8	C18H26O	C21H40O4S	C33H43NO8	C25H31NO7S2
C17H33NO4	C16H23NO8	C21H28O	C22H42O4S	C44H65NO8	C20H39NO7S3
C20H39NO4	C18H27NO8	C17H12O	C10H18O4S	C33H41NO8	C20H37NO7S3
C22H43NO4	C17H23NO8	C17H10O	C18H34O4S	C38H47NO8	C37H69NO7S3
C9H17NO4	C27H43NO8	C18H12O	C19H36O4S	C28H21NO8	C18H13NO7S3
C10H17NO4	C39H67NO8	C26H26O	C10H16O4S	C33H25NO8	C21H19NO7S3
C11H19NO4	C12H13NO8	C27H28O	C11H18O4S	C35H29NO8	C24H21NO7S3
C12H21NO4	C14H17NO8	C23H18O	C12H20O4S	C37H33NO8	C27H25NO7S3
C13H23NO4	C15H19NO8	C22H12O	C8H12O4S	C37H27NO8	C17H31NO8
C14H25NO4	C16H21NO8	C23H14O	C9H14O4S	C48H35NO8	C18H33NO8
C15H27NO4	C18H25NO8	C26H20O	C15H26O4S	C11H23NO8S	C17H29NO8
C16H29NO4	C19H27NO8	C34H26O	C18H32O4S	C18H33NO8S	C19H33NO8
C22H41NO4	C14H15NO8	C40H28O	C20H36O4S	C35H31NO8S	C31H57NO8

C9H15NO4	C15H17NO8	C38H20O	C7H10O4S	C11H23NO8S2	C11H17NO8
C7H11NO4	C16H19NO8	C11H22O10	C10H14O4S	C43H33NO8S2	C13H21NO8
C8H13NO4	C17H21NO8	C14H28O10	C11H16O4S	C13H25NO8S3	C35H65NO8
C10H15NO4	C18H23NO8	C15H30O10	C12H18O4S	C22H37NO8S3	C18H29NO8
C11H17NO4	C19H25NO8	C16H32O10	C15H24O4S	C41H31NO8S3	C14H19NO8
C12H19NO4	C20H27NO8	C17H34O10	C17H28O4S	C10H21NO9	C15H21NO8
C13H21NO4	C42H71NO8	C18H36O10	C18H30O4S	C9H19NO9	C16H23NO8
C14H23NO4	C39H63NO8	C19H38O10	C19H32O4S	C11H21NO9	C17H23NO8
C15H25NO4	C15H15NO8	C20H40O10	C8H10O4S	C13H25NO9	C27H43NO8
C8H11NO4	C16H17NO8	C21H42O10	C9H12O4S	C15H29NO9	C39H67NO8
C9H13NO4	C17H19NO8	C22H44O10	C10H12O4S	C11H19NO9	C14H17NO8
C10H13NO4	C18H21NO8	C24H48O10	C11H14O4S	C12H21NO9	C15H19NO8
C11H15NO4	C19H23NO8	C11H20O10	C12H16O4S	C13H23NO9	C16H21NO8
C12H17NO4	C20H25NO8	C14H26O10	C13H18O4S	C14H25NO9	C18H25NO8
C13H19NO4	C21H27NO8	C15H28O10	C14H20O4S	C15H27NO9	C19H27NO8
C14H21NO4	C16H15NO8	C16H30O10	C15H22O4S	C18H33NO9	C14H15NO8
C15H23NO4	C17H17NO8	C17H32O10	C18H28O4S	C10H17NO9	C15H17NO8
C16H25NO4	C18H19NO8	C18H34O10	C27H46O4S	C17H29NO9	C16H19NO8
C27H47NO4	C19H21NO8	C19H36O10	C9H10O4S	C11H17NO9	C17H21NO8
C9H11NO4	C20H23NO8	C20H38O10	C11H12O4S	C12H19NO9	C18H23NO8
C11H13NO4	C21H25NO8	C21H40O10	C12H14O4S	C18H29NO9	C19H25NO8
C12H15NO4	C22H27NO8	C22H42O10	C13H16O4S	C12H17NO9	C20H27NO8
C13H17NO4	C44H69NO8	C23H44O10	C15H20O4S	C13H19NO9	C42H71NO8
C14H19NO4	C17H15NO8	C24H46O10	C11H10O4S	C15H23NO9	C27H39NO8
C15H21NO4	C19H19NO8	C15H26O10	C15H14O4S	C17H25NO9	C39H63NO8
C16H23NO4	C20H21NO8	C16H28O10	C28H34O4S	C12H15NO9	C14H13NO8
C10H11NO4	C21H23NO8	C17H30O10	C26H28O4S	C13H17NO9	C16H17NO8
C12H13NO4	C22H25NO8	C18H32O10	C30H36O4S	C14H19NO9	C17H19NO8
C13H15NO4	C23H27NO8	C19H34O10	C30H34O4S	C28H33NO9	C18H21NO8
C14H17NO4	C24H29NO8	C20H36O10	C26H24O4S	C30H33NO9	C19H23NO8
C15H19NO4	C19H17NO8	C22H40O10	C40H32O4S	C28H25NO9	C20H25NO8
C16H21NO4	C20H19NO8	C23H42O10	C17H28O4S2	C28H23NO9	C22H29NO8
C11H11NO4	C21H21NO8	C24H44O10	C13H18O4S2	C33H15NO9	C23H31NO8
C17H23NO4	C22H23NO8	C30H56O10	C12H12O4S2	C42H27NO9	C15H13NO8
C37H61NO4	C23H25NO8	C44H84O10	C12H10O4S2	C46H33NO9	C16H15NO8
C12H11NO4	C29H35NO8	C16H26O10	C15H16O4S2	C20H33NO9S2	C17H17NO8
C13H13NO4	C44H65NO8	C17H28O10	C24H34O4S2	C27H35NO9S2	C18H19NO8
C14H15NO4	C19H15NO8	C18H30O10	C26H38O4S2	C31H23NO9S2	C19H21NO8
C15H17NO4	C20H17NO8	C19H32O10	C24H32O4S2	C39H33NO9S2	C20H23NO8
C16H19NO4	C23H23NO8	C20H34O10	C26H36O4S2	C15H29NO9S3	C22H27NO8
C13H11NO4	C33H41NO8	C21H36O10	C16H10O4S2	C30H43NO9S3	C27H35NO8
C14H13NO4	C22H19NO8	C22H38O10	C17H12O4S2	C32H43NO9S3	C44H69NO8
C15H15NO4	C23H21NO8	C23H40O10	C21H20O4S2	C18H15NOS	C17H15NO8
C16H17NO4	C24H23NO8	C24H42O10	C10H22O4S3	C22H21NOS	C19H19NO8

C17H19NO4	C26H17NO8	C25H44O10	C11H24O4S3	C22H19NOS	C20H21NO8
C18H21NO4	C29H17NO8	C11H14O10	C12H26O4S3	C33H41NOS	C21H23NO8
C31H45NO4	C41H39NO8	C13H18O10	C14H30O4S3	C30H25NOS	C22H25NO8
C14H11NO4	C41H35NO8	C14H20O10	C9H20O4S3	C40H23NOS	C20H19NO8
C15H13NO4	C43H39NO8	C15H22O10	C11H22O4S3	C13H21NOS2	C22H23NO8
C16H15NO4	C14H27NO8S	C16H24O10	C12H24O4S3	C47H83NOS2	C44H65NO8
C17H17NO4	C27H45NO8S	C17H26O10	C26H52O4S3	C14H15NOS2	C23H23NO8
C18H19NO4	C20H15NO8S	C18H28O10	C27H54O4S3	C23H29NOS2	C26H29NO8
C24H31NO4	C23H21NO8S	C19H30O10	C13H24O4S3	C22H19NOS2	C33H41NO8
C15H11NO4	C25H47NO8S3	C20H32O10	C15H28O4S3	C29H27NOS2	C22H19NO8
C16H13NO4	C31H21NO8S3	C21H34O10	C16H30O4S3	C30H29NOS2	C25H25NO8
C17H15NO4	C11H21NO9	C22H36O10	C17H32O4S3	C34H29NOS2	C27H29NO8
C18H17NO4	C14H25NO9	C23H38O10	C20H38O4S3	C35H27NOS2	C29H31NO8
C17H13NO4	C16H29NO9	C24H40O10	C18H32O4S3	C10H21NOS3	C14H27NO8S
C18H13NO4	C16H25NO9	C25H42O10	C11H16O4S3	C15H31NOS3	C20H37NO8S
C19H11NO4	C28H49NO9	C26H44O10	C32H50O4S3	C8H17NOS3	C20H15NO8S
C20H13NO4	C18H25NO9	C9H10O10	C20H22O4S3	C9H19NOS3	C23H21NO8S
C23H19NO4	C15H17NO9	C12H14O10	C22H24O4S3	C14H27NOS3	C35H29NO8S
C24H21NO4	C16H19NO9	C14H18O10	C32H44O4S3	C21H41NOS3	C20H41NO8S2
C25H23NO4	C18H23NO9	C15H20O10	C34H46O4S3	C12H21NOS3	C32H55NO8S2
C24H19NO4	C19H25NO9	C16H22O10	C34H44O4S3	C23H41NOS3	C25H51NO8S3
C25H21NO4	C23H33NO9	C17H24O10	C10H22O5	C20H33NOS3	C30H61NO8S3
C32H31NO4	C16H17NO9	C18H26O10	C11H24O5	C23H33NOS3	C29H37NO8S3
C33H33NO4	C17H19NO9	C19H28O10	C14H30O5	C25H37NOS3	C31H21NO8S3
C31H27NO4	C18H21NO9	C20H30O10	C15H32O5	C19H23NOS3	C14H29NO9
C32H29NO4	C19H23NO9	C21H32O10	C9H20O5	C30H21NOS3	C16H33NO9
C33H31NO4	C20H25NO9	C22H34O10	C14H28O5	C34H27NOS3	C11H21NO9
C32H27NO4	C21H27NO9	C23H36O10	C15H30O5	C40H35NOS3	C14H25NO9
C33H29NO4	C22H29NO9	C24H38O10	C16H32O5	C35H19NOS3	C37H69NO9
C29H19NO4	C16H15NO9	C26H42O10	C17H34O5	C47H33NOS3	C16H25NO9
C33H27NO4	C17H17NO9	C27H44O10	C18H36O5	C48H35NOS3	C17H23NO9
C33H25NO4	C18H19NO9	C13H16O10	C19H38O5	C44H88O	C22H31NO9
C33H23NO4	C19H21NO9	C12H12O10	C20H40O5	C18H32O	C23H33NO9
C29H11NO4	C21H25NO9	C20H28O10	C21H42O5	C18H28O	C25H37NO9
C36H15NO4	C22H27NO9	C21H30O10	C22H44O5	C21H28O	C16H17NO9
C20H41NO4S	C24H31NO9	C22H32O10	C23H46O5	C23H30O	C17H19NO9
C27H51NO4S	C18H17NO9	C23H34O10	C24H48O5	C17H12O	C18H21NO9
C10H15NO4S	C19H19NO9	C24H36O10	C25H50O5	C19H14O	C19H23NO9
C12H19NO4S	C20H21NO9	C25H38O10	C26H52O5	C21H18O	C21H27NO9
C13H19NO4S	C21H23NO9	C26H40O10	C5H10O5	C18H10O	C22H29NO9
C10H13NO4S	C22H25NO9	C14H16O10	C10H20O5	C26H26O	C18H19NO9
C9H11NO4S	C23H27NO9	C15H18O10	C11H22O5	C25H24O	C21H25NO9
C22H35NO4S	C19H17NO9	C16H20O10	C12H24O5	C20H12O	C18H17NO9
C11H13NO4S	C20H19NO9	C17H22O10	C8H16O5	C21H14O	C19H19NO9

$C_{12}H_{15}NO_4S$	$C_{21}H_{21}NO_9$	$C_{18}H_{24}O_{10}$	$C_9H_{18}O_5$	$C_{22}H_{16}O$	$C_{20}H_{21}NO_9$
$C_{12}H_{13}NO_4S$	$C_{22}H_{23}NO_9$	$C_{19}H_{26}O_{10}$	$C_{17}H_{32}O_5$	$C_{26}H_{24}O$	$C_{21}H_{23}NO_9$
$C_{13}H_{15}NO_4S$	$C_{25}H_{29}NO_9$	$C_{20}H_{26}O_{10}$	$C_{23}H_{44}O_5$	$C_{24}H_{20}O$	$C_{22}H_{25}NO_9$
$C_{13}H_{13}NO_4S$	$C_{30}H_{37}NO_9$	$C_{21}H_{28}O_{10}$	$C_{24}H_{46}O_5$	$C_{23}H_{12}O$	$C_{21}H_{21}NO_9$
$C_{14}H_{15}NO_4S$	$C_{21}H_{19}NO_9$	$C_{22}H_{30}O_{10}$	$C_{25}H_{48}O_5$	$C_{28}H_{22}O$	$C_{22}H_{23}NO_9$
$C_{18}H_{21}NO_4S$	$C_{22}H_{21}NO_9$	$C_{23}H_{32}O_{10}$	$C_{26}H_{50}O_5$	$C_{28}H_{14}O$	$C_{23}H_{21}NO_9$
$C_{24}H_{31}NO_4S$	$C_{23}H_{23}NO_9$	$C_{14}H_{14}O_{10}$	$C_{27}H_{52}O_5$	$C_{29}H_{16}O$	$C_{44}H_{49}NO_9$
$C_{25}H_{33}NO_4S$	$C_{25}H_{27}NO_9$	$C_{15}H_{16}O_{10}$	$C_{28}H_{54}O_5$	$C_{38}H_{24}O$	$C_{42}H_{39}NO_9$
$C_{25}H_{31}NO_4S$	$C_{22}H_{19}NO_9$	$C_{16}H_{18}O_{10}$	$C_{10}H_{18}O_5$	$C_{34}H_{14}O$	$C_{37}H_{15}NO_9$
$C_{26}H_{31}NO_4S$	$C_{24}H_{23}NO_9$	$C_{17}H_{20}O_{10}$	$C_{11}H_{20}O_5$	$C_{37}H_{18}O$	$C_{39}H_{71}NO_9S_3$
$C_{26}H_{29}NO_4S$	$C_{25}H_{25}NO_9$	$C_{18}H_{22}O_{10}$	$C_{15}H_{28}O_5$	$C_{44}H_{20}O$	$C_{11}H_{17}NOS$
$C_{26}H_{25}NO_4S$	$C_{40}H_{53}NO_9$	$C_{19}H_{24}O_{10}$	$C_{16}H_{30}O_5$	$C_{48}H_{24}O$	$C_{17}H_{17}NOS$
$C_{26}H_{21}NO_4S$	$C_{25}H_{21}NO_9$	$C_{24}H_{34}O_{10}$	$C_{19}H_{36}O_5$	$C_{10}H_{20}O_{10}$	$C_{20}H_{21}NOS$
$C_{43}H_{27}NO_4S$	$C_{42}H_{39}NO_9$	$C_{15}H_{14}O_{10}$	$C_{20}H_{38}O_5$	$C_{11}H_{22}O_{10}$	$C_{17}H_{11}NOS$
$C_{10}H_{21}NO_4S_2$	$C_{49}H_{39}NO_9$	$C_{16}H_{16}O_{10}$	$C_{21}H_{40}O_5$	$C_{12}H_{24}O_{10}$	$C_{22}H_{21}NOS$
$C_{11}H_{23}NO_4S_2$	$C_{37}H_{15}NO_9$	$C_{17}H_{18}O_{10}$	$C_{22}H_{42}O_5$	$C_{13}H_{26}O_{10}$	$C_{29}H_{31}NOS$
$C_{29}H_{37}NO_4S_2$	$C_{43}H_{21}NO_9$	$C_{18}H_{20}O_{10}$	$C_6H_{10}O_5$	$C_{14}H_{28}O_{10}$	$C_{36}H_{43}NOS$
$C_{39}H_{55}NO_4S_2$	$C_{12}H_{23}NO_9S$	$C_{19}H_{22}O_{10}$	$C_7H_{12}O_5$	$C_{15}H_{30}O_{10}$	$C_{12}H_{27}NOS_2$
$C_{25}H_{23}NO_4S_2$	$C_9H_{17}NO_9S$	$C_{20}H_{24}O_{10}$	$C_8H_{14}O_5$	$C_{16}H_{32}O_{10}$	$C_{12}H_{23}NOS_2$
$C_{39}H_{47}NO_4S_2$	$C_{43}H_{75}NO_9S$	$C_{21}H_{26}O_{10}$	$C_9H_{16}O_5$	$C_{17}H_{34}O_{10}$	$C_{26}H_{51}NOS_2$
$C_{48}H_{39}NO_4S_2$	$C_{11}H_{17}NOS$	$C_{22}H_{28}O_{10}$	$C_{23}H_{42}O_5$	$C_{18}H_{36}O_{10}$	$C_{13}H_{21}NOS_2$
$C_{26}H_{51}NO_4S_3$	$C_{19}H_{31}NOS$	$C_{23}H_{30}O_{10}$	$C_{24}H_{44}O_5$	$C_{19}H_{38}O_{10}$	$C_{40}H_{75}NOS_2$
$C_{30}H_{39}NO_4S_3$	$C_{11}H_{11}NOS$	$C_{16}H_{14}O_{10}$	$C_{25}H_{46}O_5$	$C_9H_{18}O_{10}$	$C_{24}H_{41}NOS_2$
$C_{30}H_{35}NO_4S_3$	$C_{19}H_{25}NOS$	$C_{17}H_{16}O_{10}$	$C_{26}H_{48}O_5$	$C_{12}H_{22}O_{10}$	$C_{40}H_{71}NOS_2$
$C_{40}H_{43}NO_4S_3$	$C_{29}H_{45}NOS$	$C_{18}H_{18}O_{10}$	$C_{27}H_{50}O_5$	$C_{14}H_{26}O_{10}$	$C_{22}H_{33}NOS_2$
$C_{10}H_{21}NO_5$	$C_{29}H_{37}NOS$	$C_{19}H_{20}O_{10}$	$C_{28}H_{52}O_5$	$C_{15}H_{28}O_{10}$	$C_{40}H_{67}NOS_2$
$C_{11}H_{23}NO_5$	$C_{17}H_{11}NOS$	$C_{20}H_{22}O_{10}$	$C_{10}H_{16}O_5$	$C_{16}H_{30}O_{10}$	$C_{28}H_{39}NOS_2$
$C_{13}H_{27}NO_5$	$C_{22}H_{19}NOS$	$C_{21}H_{24}O_{10}$	$C_{11}H_{18}O_5$	$C_{17}H_{32}O_{10}$	$C_{30}H_{43}NOS_2$
$C_{18}H_{37}NO_5$	$C_{34}H_{39}NOS$	$C_{22}H_{26}O_{10}$	$C_{12}H_{20}O_5$	$C_{19}H_{36}O_{10}$	$C_{22}H_{19}NOS_2$
$C_{20}H_{41}NO_5$	$C_{13}H_{21}NOS_2$	$C_{23}H_{28}O_{10}$	$C_{14}H_{24}O_5$	$C_{28}H_{54}O_{10}$	$C_{37}H_{21}NOS_2$
$C_{22}H_{45}NO_5$	$C_{18}H_{31}NOS_2$	$C_{24}H_{30}O_{10}$	$C_{15}H_{26}O_5$	$C_{30}H_{58}O_{10}$	$C_{33}H_{69}NOS_3$
$C_{24}H_{49}NO_5$	$C_{24}H_{41}NOS_2$	$C_{25}H_{32}O_{10}$	$C_{16}H_{28}O_5$	$C_{10}H_{18}O_{10}$	$C_{10}H_{21}NOS_3$
$C_8H_{17}NO_5$	$C_{28}H_{47}NOS_2$	$C_{18}H_{16}O_{10}$	$C_{19}H_{34}O_5$	$C_{11}H_{20}O_{10}$	$C_{15}H_{31}NOS_3$
$C_9H_{19}NO_5$	$C_{40}H_{71}NOS_2$	$C_{19}H_{18}O_{10}$	$C_{20}H_{36}O_5$	$C_{13}H_{24}O_{10}$	$C_7H_{15}NOS_3$
$C_{11}H_{21}NO_5$	$C_{30}H_{43}NOS_2$	$C_{20}H_{20}O_{10}$	$C_{21}H_{38}O_5$	$C_{12}H_{20}O_{10}$	$C_8H_{17}NOS_3$
$C_{12}H_{23}NO_5$	$C_{17}H_{15}NOS_2$	$C_{21}H_{22}O_{10}$	$C_{22}H_{40}O_5$	$C_{13}H_{22}O_{10}$	$C_9H_{19}NOS_3$
$C_{13}H_{25}NO_5$	$C_{47}H_{73}NOS_2$	$C_{22}H_{24}O_{10}$	$C_7H_{10}O_5$	$C_{14}H_{24}O_{10}$	$C_{21}H_{41}NOS_3$
$C_{14}H_{27}NO_5$	$C_{22}H_{19}NOS_2$	$C_{23}H_{26}O_{10}$	$C_9H_{14}O_5$	$C_{15}H_{26}O_{10}$	$C_{15}H_{27}NOS_3$
$C_{15}H_{29}NO_5$	$C_{47}H_{63}NOS_2$	$C_{24}H_{28}O_{10}$	$C_{13}H_{20}O_5$	$C_{16}H_{28}O_{10}$	$C_{13}H_{21}NOS_3$
$C_{16}H_{31}NO_5$	$C_{47}H_{59}NOS_2$	$C_{25}H_{30}O_{10}$	$C_{23}H_{40}O_5$	$C_{10}H_{16}O_{10}$	$C_{44}H_{83}NOS_3$
$C_{17}H_{33}NO_5$	$C_{37}H_{21}NOS_2$	$C_{20}H_{18}O_{10}$	$C_{24}H_{42}O_5$	$C_{11}H_{18}O_{10}$	$C_{15}H_{19}NOS_3$
$C_{18}H_{35}NO_5$	$C_{35}H_{11}NOS_2$	$C_{21}H_{20}O_{10}$	$C_{26}H_{46}O_5$	$C_{24}H_{44}O_{10}$	$C_{44}H_{77}NOS_3$
$C_{19}H_{37}NO_5$	$C_{33}H_{69}NOS_3$	$C_{22}H_{22}O_{10}$	$C_8H_{10}O_5$	$C_{14}H_{22}O_{10}$	$C_{44}H_{73}NOS_3$
$C_{20}H_{39}NO_5$	$C_{10}H_{21}NOS_3$	$C_{23}H_{24}O_{10}$	$C_9H_{12}O_5$	$C_{15}H_{24}O_{10}$	$C_{48}H_{35}NOS_3$

C21H41NO5	C15H31NOS3	C24H26O10	C10H14O5	C16H26O10	C11H20O
C22H43NO5	C7H15NOS3	C25H28O10	C11H16O5	C17H28O10	C16H30O
C24H47NO5	C8H17NOS3	C26H30O10	C12H18O5	C11H16O10	C8H14O
C9H17NO5	C9H19NOS3	C18H12O10	C17H28O5	C12H18O10	C9H16O
C10H17NO5	C21H41NOS3	C20H16O10	C18H30O5	C13H20O10	C11H18O
C11H19NO5	C15H27NOS3	C22H20O10	C20H34O5	C15H22O10	C15H26O
C12H21NO5	C15H19NOS3	C23H22O10	C21H36O5	C12H16O10	C16H28O
C13H23NO5	C30H49NOS3	C24H24O10	C10H12O5	C14H18O10	C17H30O
C14H25NO5	C40H67NOS3	C25H26O10	C11H14O5	C22H34O10	C18H32O
C15H27NO5	C40H63NOS3	C26H28O10	C12H16O5	C23H36O10	C9H14O
C16H29NO5	C33H27NOS3	C23H20O10	C13H18O5	C24H38O10	C12H18O
C17H31NO5	C18H38O	C24H22O10	C15H22O5	C26H42O10	C13H20O
C18H33NO5	C13H26O	C25H24O10	C18H28O5	C27H44O10	C14H22O
C19H35NO5	C14H28O	C26H24O10	C21H34O5	C32H54O10	C15H24O
C20H37NO5	C15H30O	C25H50O10S	C24H40O5	C16H22O10	C16H26O
C21H39NO5	C16H32O	C20H36O10S	C26H44O5	C15H18O10	C8H10O
C22H41NO5	C17H34O	C13H20O10S	C14H20O5	C24H36O10	C10H12O
C24H45NO5	C18H36O	C16H24O10S	C16H24O5	C26H40O10	C12H16O
C8H13NO5	C8H16O	C17H24O10S	C17H26O5	C16H20O10	C13H18O
C9H15NO5	C13H24O	C15H18O10S	C19H30O5	C36H60O10	C18H28O
C10H15NO5	C14H26O	C17H22O10S	C22H36O5	C36H58O10	C9H10O
C11H17NO5	C15H28O	C19H26O10S	C9H10O5	C21H20O10	C10H10O
C12H19NO5	C17H32O	C19H20O10S	C12H14O5	C22H20O10	C18H26O
C13H21NO5	C7H12O	C23H26O10S	C13H16O5	C23H20O10	C11H12O
C14H23NO5	C8H14O	C20H12O10S	C14H18O5	C36H30O10	C21H28O
C15H25NO5	C9H16O	C11H24O11	C15H20O5	C43H32O10	C23H30O
C8H11NO5	C15H26O	C12H26O11	C16H22O5	C42H26O10	C16H10O
C9H13NO5	C17H30O	C11H22O11	C17H24O5	C48H28O10	C17H12O
C12H17NO5	C18H32O	C15H30O11	C18H26O5	C22H44O10S	C19H14O
C13H19NO5	C10H16O	C16H32O11	C19H28O5	C9H18O10S	C26H26O
C14H21NO5	C11H18O	C17H34O11	C20H30O5	C20H36O10S	C27H28O
C15H23NO5	C12H20O	C18H36O11	C21H32O5	C22H26O10S	C23H18O
C16H25NO5	C13H22O	C19H38O11	C10H10O5	C37H48O10S	C26H24O
C10H13NO5	C7H10O	C20H40O11	C11H12O5	C42H38O10S	C26H22O
C11H15NO5	C8H12O	C21H42O11	C12H12O5	C13H26O10S3	C24H14O
C9H11NO5	C9H14O	C22H44O11	C13H14O5	C15H30O10S3	C26H18O
C13H17NO5	C14H22O	C23H46O11	C14H16O5	C19H38O10S3	C28H10O
C14H19NO5	C10H14O	C24H48O11	C15H18O5	C14H28O10S3	C37H24O
C15H21NO5	C11H16O	C16H30O11	C16H20O5	C18H28O10S3	C46H36O
C16H23NO5	C12H18O	C17H32O11	C17H22O5	C19H30O10S3	C36H14O
C17H25NO5	C13H20O	C18H34O11	C18H24O5	C19H28O10S3	C12H26O10
C10H11NO5	C17H28O	C19H36O11	C19H26O5	C26H34O10S3	C20H42O10
C11H13NO5	C8H10O	C20H38O11	C20H28O5	C19H18O10S3	C21H44O10
C12H15NO5	C9H12O	C21H40O11	C22H32O5	C30H30O10S3	C22H46O10

C18H27NO5	C18H28O	C22H42O11	C23H34O5	C11H24O11	C11H22O10
C12H13NO5	C16H24O	C23H44O11	C24H36O5	C12H26O11	C12H24O10
C13H15NO5	C17H26O	C24H46O11	C25H38O5	C14H30O11	C14H28O10
C14H17NO5	C9H10O	C19H34O11	C11H10O5	C10H20O11	C15H30O10
C15H19NO5	C17H12O	C20H36O11	C15H16O5	C12H24O11	C16H32O10
C16H21NO5	C17H10O	C21H38O11	C17H20O5	C13H26O11	C17H34O10
C17H23NO5	C18H12O	C22H40O11	C18H22O5	C14H28O11	C18H36O10
C11H11NO5	C26H26O	C23H42O11	C19H24O5	C15H30O11	C19H38O10
C18H25NO5	C27H28O	C19H32O11	C20H26O5	C16H32O11	C20H40O10
C19H27NO5	C23H18O	C20H34O11	C21H28O5	C17H34O11	C21H42O10
C16H19NO5	C26H24O	C21H36O11	C22H30O5	C13H24O11	C22H44O10
C17H21NO5	C27H26O	C22H38O11	C25H36O5	C14H26O11	C24H48O10
C18H23NO5	C27H24O	C23H40O11	C12H10O5	C15H28O11	C25H50O10
C12H11NO5	C35H18O	C24H42O11	C13H12O5	C16H30O11	C11H20O10
C13H13NO5	C40H28O	C17H28O11	C14H14O5	C22H42O11	C14H26O10
C14H15NO5	C34H12O	C20H32O11	C16H18O5	C26H50O11	C15H28O10
C15H17NO5	C20H42O10	C21H34O11	C23H30O5	C11H20O11	C16H30O10
C19H25NO5	C21H44O10	C22H36O11	C13H10O5	C14H24O11	C17H32O10
C13H11NO5	C22H46O10	C23H38O11	C14H12O5	C19H34O11	C18H34O10
C14H13NO5	C14H28O10	C24H40O11	C15H14O5	C26H48O11	C19H36O10
C15H15NO5	C16H32O10	C25H42O11	C16H16O5	C11H18O11	C20H38O10
C16H17NO5	C17H34O10	C14H18O11	C17H18O5	C12H20O11	C21H40O10
C17H19NO5	C18H36O10	C16H22O11	C18H20O5	C13H22O11	C30H58O10
C18H21NO5	C19H38O10	C21H32O11	C19H22O5	C16H28O11	C10H18O10
C19H23NO5	C20H40O10	C24H38O11	C17H16O5	C15H24O11	C12H22O10
C14H11NO5	C21H42O10	C17H24O11	C15H12O5	C12H18O11	C23H44O10
C15H13NO5	C22H44O10	C22H32O11	C16H14O5	C14H22O11	C15H26O10
C16H15NO5	C23H46O10	C24H36O11	C18H18O5	C15H22O11	C16H28O10
C17H17NO5	C24H48O10	C25H38O11	C20H22O5	C16H24O11	C18H32O10
C18H19NO5	C25H50O10	C15H18O11	C15H10O5	C17H26O11	C19H34O10
C19H21NO5	C15H28O10	C16H20O11	C17H14O5	C15H20O11	C20H36O10
C20H23NO5	C16H30O10	C17H22O11	C18H16O5	C24H38O11	C21H38O10
C15H11NO5	C17H32O10	C18H24O11	C19H18O5	C26H38O11	C22H40O10
C16H13NO5	C18H34O10	C19H26O11	C20H20O5	C27H40O11	C23H42O10
C17H15NO5	C19H36O10	C21H30O11	C35H50O5	C28H42O11	C31H58O10
C18H17NO5	C20H38O10	C16H18O11	C22H20O5	C22H22O11	C32H60O10
C19H19NO5	C21H40O10	C17H20O11	C23H22O5	C30H34O11	C44H84O10
C20H21NO5	C22H42O10	C18H22O11	C24H24O5	C22H16O11	C25H46O10
C28H35NO5	C23H44O10	C19H24O11	C25H26O5	C34H24O11	C18H30O10
C16H11NO5	C24H46O10	C20H26O11	C26H28O5	C43H30O11	C19H32O10
C17H13NO5	C12H22O10	C21H28O11	C27H30O5	C45H34O11	C21H36O10
C18H15NO5	C16H28O10	C22H30O11	C29H34O5	C25H48O11S	C22H38O10
C19H17NO5	C17H30O10	C17H18O11	C30H36O5	C27H46O11S	C23H40O10
C21H21NO5	C18H32O10	C18H20O11	C31H38O5	C37H58O11S	C24H42O10

C18H13NO5	C19H34O10	C19H22O11	C39H54O5	C22H26O11S	C25H44O10
C32H41NO5	C20H36O10	C20H24O11	C19H12O5	C23H28O11S	C30H54O10
C19H15NO5	C21H38O10	C21H26O11	C20H14O5	C24H30O11S	C13H20O10
C18H11NO5	C22H40O10	C22H28O11	C21H16O5	C27H28O11S	C14H22O10
C19H13NO5	C23H42O10	C23H30O11	C23H20O5	C35H30O11S	C15H24O10
C20H15NO5	C25H46O10	C24H32O11	C24H22O5	C37H24O11S	C16H26O10
C21H17NO5	C30H56O10	C18H18O11	C25H24O5	C18H26O11S2	C17H28O10
C22H19NO5	C32H60O10	C19H20O11	C26H26O5	C18H24O11S2	C11H14O10
C48H69NO5	C44H84O10	C20H22O11	C27H28O5	C20H28O11S2	C21H34O10
C20H13NO5	C17H28O10	C21H24O11	C28H30O5	C24H24O11S2	C22H36O10
C28H29NO5	C18H30O10	C22H26O11	C30H34O5	C14H30O11S3	C23H38O10
C20H11NO5	C19H32O10	C23H28O11	C31H36O5	C31H62O11S3	C24H40O10
C21H13NO5	C20H34O10	C24H30O11	C33H40O5	C21H38O11S3	C27H46O10
C28H27NO5	C21H36O10	C18H16O11	C34H42O5	C25H40O11S3	C33H58O10
C22H15NO5	C22H38O10	C19H18O11	C35H44O5	C25H30O11S3	C42H76O10
C48H65NO5	C23H40O10	C20H20O11	C22H16O5	C34H46O11S3	C12H16O10
C28H25NO5	C24H42O10	C21H22O11	C24H20O5	C35H48O11S3	C13H18O10
C31H29NO5	C25H44O10	C22H24O11	C25H22O5	C30H32O11S3	C14H20O10
C46H59NO5	C42H78O10	C23H26O11	C26H24O5	C12H26O12	C15H22O10
C31H27NO5	C44H82O10	C24H28O11	C27H26O5	C13H28O12	C16H24O10
C34H33NO5	C14H22O10	C25H30O11	C31H34O5	C14H30O12	C17H26O10
C31H25NO5	C16H26O10	C20H18O11	C33H38O5	C12H24O12	C18H28O10
C46H55NO5	C11H14O10	C21H20O11	C34H40O5	C15H30O12	C19H30O10
C32H25NO5	C13H18O10	C22H22O11	C35H42O5	C16H32O12	C20H32O10
C48H57NO5	C14H20O10	C23H24O11	C37H46O5	C17H34O12	C19H28O10
C46H51NO5	C16H24O10	C24H26O11	C29H30O5	C13H26O12	C21H32O10
C38H25NO5	C17H26O10	C25H28O11	C30H32O5	C14H26O12	C22H34O10
C40H29NO5	C19H30O10	C26H30O11	C29H28O5	C16H30O12	C23H36O10
C39H21NO5	C20H32O10	C26H28O11	C33H36O5	C18H34O12	C24H38O10
C40H15NO5	C21H34O10	C21H18O11	C34H38O5	C12H22O12	C26H42O10
C12H25NO5S	C22H36O10	C22H20O11	C35H40O5	C13H24O12	C14H18O10
C22H43NO5S	C23H38O10	C23H22O11	C36H42O5	C14H24O12	C15H20O10
C9H13NO5S	C24H40O10	C24H24O11	C37H44O5	C15H26O12	C16H22O10
C11H15NO5S	C25H42O10	C25H26O11	C38H46O5	C12H20O12	C17H24O10
C14H21NO5S	C9H10O10	C27H30O11	C30H30O5	C13H22O12	C18H26O10
C21H33NO5S	C15H22O10	C25H24O11	C25H18O5	C12H18O12	C20H30O10
C11H13NO5S	C18H28O10	C27H28O11	C27H22O5	C14H22O12	C20H28O10
C12H15NO5S	C14H18O10	C23H12O11	C32H32O5	C19H30O12	C21H30O10
C13H17NO5S	C16H22O10	C36H16O11	C35H24O5	C17H20O12	C22H32O10
C14H19NO5S	C17H24O10	C42H10O11	C37H26O5	C23H28O12	C23H34O10
C15H21NO5S	C18H26O10	C24H46O11S	C10H22O5S	C31H36O12	C24H36O10
C12H13NO5S	C19H28O10	C25H48O11S	C11H24O5S	C37H38O12	C14H16O10
C13H15NO5S	C20H30O10	C13H20O11S	C12H26O5S	C38H38O12	C15H18O10
C14H17NO5S	C21H32O10	C17H20O11S	C13H28O5S	C34H26O12	C16H20O10

C15H19NO5S	C22H34O10	C21H10O11S	C15H32O5S	C38H24O12	C17H22O10
C13H13NO5S	C23H36O10	C33H20O11S	C8H18O5S	C11H22O13	C18H24O10
C14H15NO5S	C24H38O10	C37H16O11S	C9H20O5S	C12H24O13	C19H26O10
C15H17NO5S	C25H40O10	C30H24O11S2	C10H20O5S	C13H26O13	C20H26O10
C39H65NO5S	C26H42O10	C12H26O12	C11H22O5S	C14H28O13	C22H30O10
C14H13NO5S	C27H44O10	C14H30O12	C13H26O5S	C15H30O13	C24H34O10
C16H17NO5S	C15H20O10	C15H32O12	C14H28O5S	C16H32O13	C15H16O10
C25H33NO5S	C12H12O10	C12H24O12	C17H34O5S	C18H36O13	C16H18O10
C15H13NO5S	C20H28O10	C19H38O12	C18H36O5S	C14H26O13	C17H20O10
C48H65NO5S	C21H30O10	C20H40O12	C19H38O5S	C16H30O13	C18H22O10
C16H29NO5S2	C22H32O10	C21H42O12	C20H40O5S	C13H24O13	C19H24O10
C40H73NO5S2	C23H34O10	C22H44O12	C5H10O5S	C15H28O13	C21H28O10
C24H39NO5S2	C25H38O10	C12H22O12	C6H12O5S	C28H54O13	C23H32O10
C24H35NO5S2	C26H40O10	C19H36O12	C7H14O5S	C30H58O13	C25H36O10
C24H31NO5S2	C14H16O10	C20H38O12	C8H16O5S	C14H24O13	C16H16O10
C26H35NO5S2	C15H18O10	C21H40O12	C9H18O5S	C15H26O13	C17H18O10
C24H27NO5S2	C16H20O10	C22H36O12	C10H18O5S	C16H28O13	C18H20O10
C21H15NO5S2	C17H22O10	C23H38O12	C11H20O5S	C17H30O13	C19H22O10
C35H39NO5S2	C18H24O10	C24H40O12	C13H24O5S	C19H34O13	C20H24O10
C35H35NO5S2	C19H26O10	C25H42O12	C14H26O5S	C20H36O13	C21H26O10
C37H37NO5S2	C15H16O10	C15H20O12	C15H28O5S	C28H52O13	C22H28O10
C38H39NO5S2	C16H18O10	C24H38O12	C18H34O5S	C30H56O13	C23H30O10
C39H41NO5S2	C17H20O10	C19H24O12	C19H36O5S	C14H22O13	C25H34O10
C35H27NO5S2	C18H22O10	C20H26O12	C20H38O5S	C30H54O13	C15H12O10
C38H27NO5S2	C19H24O10	C20H24O12	C21H40O5S	C30H52O13	C16H14O10
C17H33NO5S3	C20H26O10	C21H26O12	C6H10O5S	C21H28O13	C17H16O10
C17H29NO5S3	C21H28O10	C22H28O12	C7H12O5S	C18H12O13	C18H18O10
C16H13NO5S3	C22H30O10	C20H22O12	C8H14O5S	C26H26O13	C19H20O10
C36H51NO5S3	C23H32O10	C21H24O12	C9H16O5S	C23H18O13	C20H22O10
C17H13NO5S3	C15H14O10	C22H26O12	C10H16O5S	C24H20O13	C21H24O10
C17H35NO6	C16H16O10	C23H28O12	C11H18O5S	C42H36O13	C22H26O10
C19H39NO6	C17H18O10	C20H20O12	C12H20O5S	C45H34O13	C23H28O10
C17H33NO6	C18H20O10	C22H24O12	C13H22O5S	C23H34O13S2	C24H30O10
C18H35NO6	C19H22O10	C23H26O12	C14H24O5S	C27H28O13S2	C25H32O10
C20H39NO6	C20H24O10	C24H28O12	C15H26O5S	C29H32O13S2	C17H14O10
C21H41NO6	C21H26O10	C23H24O12	C18H32O5S	C12H24O14	C18H16O10
C22H43NO6	C22H28O10	C24H26O12	C19H34O5S	C14H28O14	C19H18O10
C12H21NO6	C23H30O10	C26H30O12	C20H36O5S	C17H32O14	C20H20O10
C14H25NO6	C16H14O10	C24H24O12	C21H38O5S	C14H26O14	C21H22O10
C15H27NO6	C17H16O10	C26H28O12	C7H10O5S	C15H28O14	C22H24O10
C17H31NO6	C18H18O10	C25H24O12	C8H12O5S	C16H30O14	C24H28O10
C18H33NO6	C19H20O10	C23H14O12	C9H14O5S	C18H34O14	C26H32O10
C19H35NO6	C20H22O10	C31H12O12	C12H18O5S	C19H36O14	C20H18O10
C20H37NO6	C21H24O10	C16H30O12S	C13H20O5S	C14H24O14	C21H20O10

C21H39NO6	C22H26O10	C18H32O12S	C14H22O5S	C15H26O14	C22H22O10
C22H41NO6	C23H28O10	C15H18O12S	C15H24O5S	C16H28O14	C23H24O10
C23H43NO6	C24H30O10	C12H24O13	C17H28O5S	C17H30O14	C24H26O10
C24H45NO6	C18H16O10	C13H26O13	C8H10O5S	C21H38O14	C25H28O10
C13H21NO6	C19H18O10	C14H28O13	C13H18O5S	C30H56O14	C26H30O10
C15H25NO6	C20H20O10	C15H30O13	C10H12O5S	C16H26O14	C18H12O10
C16H27NO6	C21H22O10	C17H34O13	C11H14O5S	C17H28O14	C21H18O10
C17H29NO6	C22H24O10	C23H26O13	C12H16O5S	C30H54O14	C22H20O10
C18H31NO6	C23H26O10	C17H12O13	C14H20O5S	C13H18O14	C23H22O10
C19H33NO6	C24H28O10	C23H24O13	C15H22O5S	C21H32O14	C24H24O10
C20H35NO6	C25H30O10	C24H26O13	C16H24O5S	C22H28O14	C25H26O10
C21H37NO6	C26H32O10	C25H28O13	C17H26O5S	C26H28O14	C22H18O10
C10H15NO6	C18H14O10	C27H30O13	C9H10O5S	C26H22O14	C23H20O10
C11H17NO6	C20H18O10	C26H26O13	C10H10O5S	C35H38O14	C25H24O10
C12H19NO6	C21H20O10	C15H18O15S	C11H12O5S	C12H22O14S	C34H18O10
C14H23NO6	C22H22O10	C17H12O16	C12H14O5S	C25H36O14S	C25H50O10S
C14H21NO6	C23H24O10	C31H18O16	C13H16O5S	C20H20O14S	C26H52O10S
C19H31NO6	C24H26O10	C18H38O2	C14H18O5S	C29H14O14S	C26H50O10S
C27H47NO6	C25H28O10	C5H10O2	C15H20O5S	C14H26O15	C27H52O10S
C39H71NO6	C26H30O10	C6H12O2	C16H22O5S	C21H40O15	C29H56O10S
C10H13NO6	C17H10O10	C7H14O2	C18H26O5S	C15H28O15	C24H44O10S
C11H15NO6	C18H12O10	C11H22O2	C12H12O5S	C16H30O15	C25H46O10S
C12H17NO6	C19H14O10	C8H16O2	C14H16O5S	C17H32O15	C18H32O10S
C13H19NO6	C20H16O10	C9H18O2	C16H20O5S	C18H34O15	C20H36O10S
C15H23NO6	C21H18O10	C13H24O2	C13H12O5S	C19H36O15	C22H40O10S
C16H25NO6	C22H20O10	C6H10O2	C15H16O5S	C14H24O15	C23H40O10S
C18H29NO6	C23H22O10	C7H12O2	C16H16O5S	C16H28O15	C13H20O10S
C9H11NO6	C24H24O10	C10H18O2	C17H16O5S	C17H30O15	C15H24O10S
C15H21NO6	C25H26O10	C11H20O2	C19H20O5S	C18H32O15	C20H34O10S
C16H23NO6	C26H28O10	C12H22O2	C16H12O5S	C19H34O15	C14H20O10S
C17H25NO6	C22H18O10	C8H14O2	C26H28O5S	C20H36O15	C15H22O10S
C33H57NO6	C23H20O10	C9H16O2	C28H32O5S	C21H38O15	C17H22O10S
C10H11NO6	C24H22O10	C11H18O2	C26H26O5S	C22H40O15	C16H18O10S
C11H13NO6	C25H24O10	C12H20O2	C28H30O5S	C30H56O15	C18H22O10S
C12H15NO6	C39H14O10	C14H24O2	C36H20O5S	C16H26O15	C19H24O10S
C13H17NO6	C25H50O10S	C15H26O2	C18H34O5S2	C17H28O15	C20H26O10S
C14H19NO6	C20H38O10S	C17H30O2	C20H38O5S2	C18H30O15	C19H22O10S
C18H27NO6	C22H40O10S	C7H10O2	C12H20O5S2	C19H32O15	C12H26O11
C15H19NO6	C18H24O10S	C8H12O2	C14H24O5S2	C30H54O15	C13H28O11
C19H27NO6	C16H18O10S	C10H16O2	C10H14O5S2	C18H28O15	C14H30O11
C11H11NO6	C18H22O10S	C13H22O2	C24H42O5S2	C20H30O15	C22H46O11
C12H13NO6	C12H24O11	C9H14O2	C9H12O5S2	C29H48O15	C24H50O11
C13H15NO6	C13H26O11	C12H18O2	C13H16O5S2	C19H24O15	C11H22O11
C14H17NO6	C15H30O11	C13H20O2	C28H40O5S2	C20H26O15	C12H24O11

C16H21NO6	C16H32O11	C14H22O2	C18H20O5S2	C21H28O15	C13H26O11
C17H23NO6	C17H34O11	C10H14O2	C29H42O5S2	C14H28O16	C14H28O11
C18H25NO6	C18H36O11	C11H16O2	C16H12O5S2	C15H30O16	C15H30O11
C20H29NO6	C19H38O11	C17H28O2	C10H22O5S3	C16H32O16	C16H32O11
C17H21NO6	C20H40O11	C8H10O2	C12H26O5S3	C14H26O16	C17H34O11
C18H23NO6	C21H42O11	C9H12O2	C13H28O5S3	C16H30O16	C18H36O11
C19H25NO6	C22H44O11	C12H16O2	C23H48O5S3	C17H32O16	C19H38O11
C12H11NO6	C23H46O11	C13H18O2	C26H54O5S3	C18H34O16	C20H40O11
C13H13NO6	C24H48O11	C18H28O2	C8H18O5S3	C18H32O16	C21H42O11
C14H15NO6	C16H30O11	C9H10O2	C13H26O5S3	C17H30O16	C22H44O11
C15H17NO6	C17H32O11	C10H12O2	C16H32O5S3	C19H34O16	C23H46O11
C16H19NO6	C18H34O11	C11H14O2	C17H34O5S3	C20H36O16	C24H48O11
C20H27NO6	C19H36O11	C17H26O2	C23H46O5S3	C21H38O16	C25H50O11
C31H47NO6	C20H38O11	C20H32O2	C26H52O5S3	C30H56O16	C26H52O11
C13H11NO6	C21H40O11	C10H10O2	C17H32O5S3	C32H60O16	C18H34O11
C14H13NO6	C22H42O11	C11H12O2	C20H38O5S3	C18H30O16	C19H36O11
C15H15NO6	C23H44O11	C12H14O2	C13H22O5S3	C19H32O16	C20H38O11
C16H17NO6	C26H50O11	C13H16O2	C15H26O5S3	C20H34O16	C21H40O11
C17H19NO6	C19H34O11	C16H22O2	C20H36O5S3	C30H54O16	C23H44O11
C18H21NO6	C20H36O11	C18H26O2	C19H20O5S3	C32H58O16	C24H46O11
C19H23NO6	C21H38O11	C12H12O2	C19H18O5S3	C15H22O16	C26H50O11
C20H25NO6	C20H34O11	C13H14O2	C22H24O5S3	C17H26O16	C14H26O11
C21H27NO6	C21H36O11	C23H32O2	C25H30O5S3	C30H40O16S	C22H42O11
C18H19NO6	C22H38O11	C13H10O2	C33H40O5S3	C22H12O16S	C12H20O11
C14H11NO6	C23H40O11	C23H30O2	C25H22O5S3	C15H30O17	C21H36O11
C15H13NO6	C24H42O11	C16H10O2	C11H24O6	C18H36O17	C22H38O11
C16H15NO6	C16H24O11	C17H12O2	C12H26O6	C18H34O17	C24H42O11
C17H17NO6	C20H32O11	C18H14O2	C13H28O6	C20H38O17	C17H28O11
C19H21NO6	C21H34O11	C19H16O2	C14H30O6	C18H32O17	C16H24O11
C20H23NO6	C22H36O11	C20H18O2	C15H32O6	C19H34O17	C21H34O11
C21H25NO6	C23H38O11	C18H12O2	C18H38O6	C20H36O17	C22H36O11
C22H27NO6	C24H40O11	C22H18O2	C9H20O6	C21H38O17	C23H38O11
C19H19NO6	C16H22O11	C22H16O2	C11H22O6	C22H40O17	C24H40O11
C21H23NO6	C22H34O11	C22H14O2	C13H26O6	C18H30O17	C25H42O11
C15H11NO6	C23H36O11	C29H28O2	C15H30O6	C19H32O17	C42H76O11
C16H13NO6	C24H38O11	C31H32O2	C16H32O6	C20H34O17	C17H26O11
C17H15NO6	C25H40O11	C38H10O2	C17H34O6	C21H36O17	C19H30O11
C18H17NO6	C19H28O11	C10H16O2S	C18H36O6	C22H38O17	C16H22O11
C20H21NO6	C32H54O11	C13H22O2S	C19H38O6	C22H28O17	C22H34O11
C22H25NO6	C18H24O11	C14H24O2S	C20H40O6	C35H52O17	C23H36O11
C23H25NO6	C19H26O11	C16H28O2S	C21H42O6	C30H42O17S	C24H38O11
C46H71NO6	C20H28O11	C7H10O2S	C22H44O6	C18H36O18	C27H44O11
C16H11NO6	C12H10O11	C9H14O2S	C8H16O6	C17H32O18	C17H24O11
C17H13NO6	C17H20O11	C9H12O2S	C9H18O6	C18H34O18	C18H26O11

C18H15NO6	C18H22O11	C17H24O2S	C17H32O6	C18H32O18	C19H28O11
C19H17NO6	C19H24O11	C18H22O2S	C19H36O6	C20H36O18	C20H30O11
C20H19NO6	C20H26O11	C20H26O2S	C20H38O6	C21H38O18	C21H32O11
C21H21NO6	C21H28O11	C12H10O2S	C21H40O6	C20H34O18	C25H38O11
C22H23NO6	C22H30O11	C13H12O2S	C23H44O6	C21H36O18	C16H20O11
C32H41NO6	C17H18O11	C15H16O2S	C25H48O6	C22H38O18	C17H22O11
C18H13NO6	C18H20O11	C25H36O2S	C26H50O6	C23H40O18	C18H24O11
C19H15NO6	C19H22O11	C13H10O2S	C9H16O6	C32H36O18	C19H26O11
C20H17NO6	C20H24O11	C14H12O2S	C10H18O6	C33H38O18	C20H28O11
C21H19NO6	C21H26O11	C15H14O2S	C11H20O6	C33H36O18	C21H30O11
C22H21NO6	C22H28O11	C16H16O2S	C12H22O6	C16H30O19	C22H32O11
C23H23NO6	C23H30O11	C17H18O2S	C14H26O6	C21H38O19	C23H34O11
C18H11NO6	C24H32O11	C18H20O2S	C15H28O6	C26H26O19	C24H36O11
C19H13NO6	C18H18O11	C25H34O2S	C16H30O6	C18H38O2	C17H20O11
C20H15NO6	C19H20O11	C27H38O2S	C18H34O6	C6H12O2	C18H22O11
C21H17NO6	C20H22O11	C14H10O2S	C22H42O6	C7H14O2	C19H24O11
C46H67NO6	C21H24O11	C15H12O2S	C24H46O6	C8H16O2	C20H26O11
C22H19NO6	C22H26O11	C16H14O2S	C6H10O6	C9H18O2	C21H28O11
C19H11NO6	C23H28O11	C18H18O2S	C8H14O6	C12H20O2	C22H30O11
C20H13NO6	C24H30O11	C27H36O2S	C10H16O6	C15H26O2	C23H32O11
C21H15NO6	C18H16O11	C15H10O2S	C11H18O6	C21H38O2	C24H34O11
C22H17NO6	C20H20O11	C16H12O2S	C22H40O6	C12H18O2	C25H36O11
C23H19NO6	C21H22O11	C17H14O2S	C24H44O6	C13H20O2	C27H40O11
C20H11NO6	C22H24O11	C26H30O2S	C25H46O6	C13H18O2	C18H20O11
C50H71NO6	C23H26O11	C19H14O2S	C26H48O6	C13H12O2	C19H22O11
C22H15NO6	C24H28O11	C23H16O2S	C12H20O6	C23H32O2	C20H24O11
C24H19NO6	C25H30O11	C17H24O2S2	C13H22O6	C25H36O2	C21H26O11
C28H27NO6	C20H18O11	C20H28O2S2	C17H30O6	C23H30O2	C22H28O11
C30H31NO6	C22H22O11	C11H10O2S2	C18H32O6	C18H12O2	C23H30O11
C32H35NO6	C23H24O11	C12H12O2S2	C23H42O6	C22H16O2	C24H32O11
C22H13NO6	C24H26O11	C20H26O2S2	C8H12O6	C22H14O2	C19H20O11
C30H29NO6	C25H28O11	C21H28O2S2	C9H14O6	C30H18O2	C20H22O11
C31H29NO6	C26H30O11	C12H10O2S2	C11H16O6	C29H10O2	C21H24O11
C30H25NO6	C24H24O11	C13H12O2S2	C16H26O6	C33H12O2	C22H26O11
C39H31NO6	C25H26O11	C19H22O2S2	C24H42O6	C35H16O2	C23H28O11
C31H11NO6	C26H28O11	C21H24O2S2	C25H44O6	C37H18O2	C24H30O11
C16H31NO6S	C25H24O11	C25H18O2S2	C26H46O6	C39H22O2	C26H34O11
C9H17NO6S	C26H26O11	C34H22O2S2	C27H48O6	C50H42O2	C28H38O11
C22H41NO6S	C27H28O11	C38H30O2S2	C29H52O6	C36H10O2	C20H20O11
C21H33NO6S	C29H30O11	C34H20O2S2	C10H14O6	C44H26O2	C21H22O11
C12H15NO6S	C24H18O11	C33H18O2S2	C12H18O6	C46H30O2	C22H24O11
C13H17NO6S	C28H26O11	C11H22O2S3	C13H20O6	C43H18O2	C23H26O11
C16H23NO6S	C23H12O11	C12H24O2S3	C14H22O6	C18H36O20	C24H28O11
C12H13NO6S	C42H10O11	C13H26O2S3	C15H24O6	C21H38O20	C20H18O11

C13H15NO6S	C24H46O11S	C14H28O2S3	C17H28O6	C23H40O20	C23H24O11
C14H17NO6S	C21H10O11S	C15H30O2S3	C18H30O6	C23H38O20	C24H26O11
C16H21NO6S	C19H40O11S2	C16H32O2S3	C21H36O6	C24H40O20	C25H28O11
C12H11NO6S	C30H24O11S2	C14H26O2S3	C22H38O6	C25H34O20	C26H30O11
C13H13NO6S	C20H40O12	C18H24O2S3	C23H40O6	C13H22O2S	C23H22O11
C14H15NO6S	C21H42O12	C18H18O2S3	C8H10O6	C14H24O2S	C24H24O11
C15H17NO6S	C19H24O12	C16H12O2S3	C9H12O6	C16H28O2S	C25H26O11
C17H21NO6S	C19H22O12	C22H18O2S3	C11H14O6	C16H24O2S	C30H34O11
C14H13NO6S	C20H24O12	C34H18O2S3	C13H18O6	C28H40O2S	C29H30O11
C17H19NO6S	C21H26O12	C36H22O2S3	C14H20O6	C30H40O2S	C20H40O11S
C19H23NO6S	C22H28O12	C11H24O3	C16H24O6	C17H12O2S	C21H42O11S
C17H17NO6S	C20H22O12	C12H26O3	C17H26O6	C17H10O2S	C24H46O11S
C18H19NO6S	C21H22O12	C13H28O3	C18H28O6	C19H14O2S	C26H50O11S
C22H45NO6S2	C22H24O12	C13H26O3	C19H30O6	C21H18O2S	C28H54O11S
C25H45NO6S2	C23H26O12	C14H28O3	C22H36O6	C23H18O2S	C29H56O11S
C44H81NO6S2	C24H28O12	C15H30O3	C23H38O6	C28H28O2S	C25H46O11S
C34H59NO6S2	C24H26O12	C19H38O3	C24H40O6	C35H26O2S	C26H48O11S
C30H35NO6S2	C26H26O12	C20H40O3	C29H50O6	C36H28O2S	C17H22O11S
C39H43NO6S2	C23H14O12	C21H42O3	C10H12O6	C10H18O2S2	C11H24O12
C29H19NO6S2	C31H12O12	C10H20O3	C12H16O6	C21H28O2S2	C12H26O12
C19H41NO6S3	C47H24O12	C11H22O3	C15H22O6	C24H32O2S2	C13H28O12
C19H39NO6S3	C45H18O12	C12H24O3	C20H32O6	C24H30O2S2	C14H30O12
C10H21NO6S3	C12H24O13	C5H10O3	C21H34O6	C25H32O2S2	C15H32O12
C12H25NO6S3	C13H26O13	C6H12O3	C9H10O6	C32H26O2S2	C16H32O12
C19H35NO6S3	C14H28O13	C7H14O3	C13H16O6	C29H18O2S2	C13H22O12
C29H49NO6S3	C17H12O13	C8H16O3	C14H18O6	C35H28O2S2	C23H38O12
C18H37NO7	C18H12O13	C13H24O3	C15H20O6	C33H18O2S2	C25H42O12
C22H41NO7	C34H18O15	C15H28O3	C16H22O6	C39H24O2S2	C27H46O12
C16H27NO7	C36H26O15S3	C6H10O3	C17H24O6	C38H20O2S2	C28H48O12
C17H29NO7	C17H12O16	C14H26O3	C18H26O6	C38H16O2S2	C16H22O12
C18H31NO7	C33H62O18S	C7H12O3	C20H30O6	C33H66O2S3	C24H38O12
C19H33NO7	C20H18O19	C9H16O3	C21H32O6	C13H26O2S3	C40H70O12
C20H35NO7	C23H24O19	C15H26O3	C22H34O6	C18H24O2S3	C36H60O12
C21H37NO7	C18H38O2	C17H30O3	C10H10O6	C24H36O2S3	C38H64O12
C22H39NO7	C5H10O2	C22H40O3	C11H12O6	C28H26O2S3	C21H30O12
C23H41NO7	C6H12O2	C11H18O3	C12H14O6	C34H30O2S3	C20H26O12
C25H45NO7	C7H14O2	C16H28O3	C19H28O6	C36H28O2S3	C21H28O12
C10H15NO7	C11H22O2	C20H36O3	C25H40O6	C43H40O2S3	C22H30O12
C11H17NO7	C8H16O2	C7H10O3	C14H16O6	C35H20O2S3	C24H34O12
C12H19NO7	C9H18O2	C8H12O3	C15H18O6	C11H24O3	C20H24O12
C15H25NO7	C13H24O2	C9H14O3	C16H20O6	C12H26O3	C22H28O12
C15H23NO7	C6H10O2	C11H16O3	C17H22O6	C13H28O3	C20H22O12
C10H13NO7	C7H12O2	C12H18O3	C18H24O6	C13H26O3	C21H24O12
C11H15NO7	C10H18O2	C22H38O3	C19H26O6	C20H40O3	C23H28O12

C12H17NO7	C11H20O2	C8H10O3	C20H28O6	C14H28O3	C22H24O12
C13H19NO7	C12H22O2	C10H14O3	C21H30O6	C22H44O3	C23H26O12
C14H21NO7	C8H14O2	C13H20O3	C11H10O6	C24H48O3	C24H28O12
C16H25NO7	C9H16O2	C15H24O3	C12H12O6	C5H10O3	C26H30O12
C17H27NO7	C11H18O2	C20H34O3	C13H14O6	C6H12O3	C23H22O12
C11H13NO7	C12H20O2	C9H12O3	C22H32O6	C7H14O3	C24H24O12
C12H15NO7	C14H24O2	C12H16O3	C25H38O6	C13H24O3	C25H24O12
C13H17NO7	C15H26O2	C13H18O3	C14H14O6	C15H28O3	C31H12O12
C14H19NO7	C17H30O2	C14H20O3	C16H18O6	C22H42O3	C45H18O12
C15H21NO7	C21H38O2	C16H24O3	C17H20O6	C6H10O3	C20H42O12S
C16H23NO7	C7H10O2	C17H26O3	C18H22O6	C9H16O3	C19H38O12S
C17H25NO7	C8H12O2	C18H28O3	C19H24O6	C15H26O3	C20H40O12S
C18H27NO7	C10H16O2	C22H36O3	C12H10O6	C22H40O3	C19H36O12S
C19H29NO7	C13H22O2	C10H12O3	C13H12O6	C11H18O3	C20H38O12S
C20H31NO7	C9H14O2	C11H14O3	C15H16O6	C20H36O3	C24H46O12S
C11H11NO7	C12H18O2	C15H22O3	C20H26O6	C8H12O3	C26H50O12S
C12H13NO7	C13H20O2	C20H32O3	C22H30O6	C9H14O3	C36H70O12S
C13H15NO7	C14H22O2	C9H10O3	C16H16O6	C11H16O3	C19H32O12S
C14H17NO7	C10H14O2	C10H10O3	C14H12O6	C12H18O3	C15H18O12S
C15H19NO7	C11H16O2	C11H12O3	C15H14O6	C22H38O3	C12H24O13
C16H21NO7	C17H28O2	C13H16O3	C17H18O6	C8H10O3	C13H26O13
C17H23NO7	C8H10O2	C14H18O3	C18H20O6	C18H28O3	C14H28O13
C18H25NO7	C9H12O2	C15H20O3	C19H22O6	C12H16O3	C15H30O13
C19H27NO7	C12H16O2	C16H22O3	C20H24O6	C15H22O3	C22H44O13
C20H29NO7	C18H28O2	C18H26O3	C21H26O6	C9H10O3	C15H28O13
C12H11NO7	C10H12O2	C20H30O3	C15H12O6	C12H14O3	C26H32O13
C13H13NO7	C11H14O2	C12H14O3	C16H14O6	C18H26O3	C17H12O13
C14H15NO7	C13H18O2	C17H24O3	C17H16O6	C20H30O3	C18H12O13
C15H17NO7	C17H26O2	C19H28O3	C18H18O6	C23H34O3	C32H24O13
C16H19NO7	C20H32O2	C11H10O3	C19H20O6	C25H38O3	C15H30O14
C17H21NO7	C9H10O2	C18H24O3	C20H22O6	C30H48O3	C15H28O14
C18H23NO7	C11H12O2	C23H34O3	C21H24O6	C23H30O3	C17H32O14
C19H25NO7	C10H10O2	C12H12O3	C17H14O6	C24H32O3	C18H34O14
C20H27NO7	C12H14O2	C13H14O3	C18H16O6	C25H34O3	C32H48O14
C21H29NO7	C13H16O2	C14H16O3	C19H18O6	C18H16O3	C26H28O14
C39H63NO7	C15H20O2	C15H18O3	C20H20O6	C27H34O3	C25H14O14
C13H11NO7	C16H22O2	C13H12O3	C21H22O6	C22H18O3	C34H12O14
C14H13NO7	C17H24O2	C19H22O3	C24H26O6	C23H16O3	C18H38O2
C15H15NO7	C18H26O2	C14H12O3	C24H24O6	C24H18O3	C5H10O2
C16H17NO7	C20H30O2	C16H16O3	C25H26O6	C41H40O3	C6H12O2
C17H19NO7	C25H40O2	C19H20O3	C26H28O6	C31H18O3	C7H14O2
C18H21NO7	C23H34O2	C23H28O3	C27H30O6	C34H18O3	C8H16O2
C19H23NO7	C12H12O2	C15H12O3	C28H32O6	C34H16O3	C9H18O2
C20H25NO7	C23H32O2	C20H20O3	C29H34O6	C35H18O3	C13H24O2

C21H27NO7	C13H10O2	C18H14O3	C30H36O6	C47H42O3	C6H10O2
C22H29NO7	C23H30O2	C19H16O3	C31H38O6	C49H46O3	C7H12O2
C23H31NO7	C16H10O2	C24H26O3	C36H48O6	C33H14O3	C10H18O2
C14H11NO7	C17H12O2	C30H38O3	C38H52O6	C35H16O3	C12H22O2
C15H13NO7	C18H14O2	C31H40O3	C18H10O6	C49H44O3	C22H42O2
C16H15NO7	C19H16O2	C32H42O3	C20H14O6	C37H20O3	C8H14O2
C17H17NO7	C20H18O2	C33H44O3	C21H16O6	C49H42O3	C9H16O2
C18H19NO7	C31H40O2	C18H12O3	C22H18O6	C39H18O3	C11H18O2
C19H21NO7	C18H12O2	C19H14O3	C23H20O6	C43H20O3	C12H20O2
C20H23NO7	C22H18O2	C23H22O3	C24H22O6	C48H18O3	C14H24O2
C21H25NO7	C29H32O2	C25H26O3	C25H24O6	C12H26O3S	C15H26O2
C22H27NO7	C30H32O2	C26H28O3	C26H26O6	C6H12O3S	C17H30O2
C23H29NO7	C31H32O2	C27H30O3	C27H28O6	C8H16O3S	C21H38O2
C16H13NO7	C29H18O2	C31H38O3	C28H30O6	C13H24O3S	C7H10O2
C17H15NO7	C38H10O2	C32H40O3	C29H32O6	C39H68O3S	C8H12O2
C18H17NO7	C13H28O2S	C33H42O3	C38H50O6	C15H14O3S	C10H16O2
C19H19NO7	C13H26O2S	C29H34O3	C22H16O6	C16H16O3S	C13H22O2
C20H21NO7	C10H16O2S	C26H26O3	C38H48O6	C18H20O3S	C9H14O2
C21H23NO7	C13H22O2S	C27H28O3	C40H52O6	C27H38O3S	C12H18O2
C22H25NO7	C9H14O2S	C29H32O3	C48H68O6	C26H34O3S	C13H20O2
C23H27NO7	C17H20O2S	C31H36O3	C31H34O6	C18H12O3S	C14H22O2
C24H29NO7	C18H22O2S	C32H38O3	C36H24O6	C21H18O3S	C17H28O2
C16H11NO7	C20H26O2S	C33H40O3	C44H40O6	C20H16O3S	C21H36O2
C17H13NO7	C12H10O2S	C34H42O3	C46H42O6	C28H30O3S	C11H16O2
C18H15NO7	C13H12O2S	C35H44O3	C46H40O6	C32H38O3S	C8H10O2
C19H17NO7	C15H16O2S	C24H20O3	C9H20O6S	C38H50O3S	C9H12O2
C20H19NO7	C19H24O2S	C26H24O3	C10H20O6S	C44H62O3S	C12H16O2
C21H21NO7	C25H36O2S	C27H26O3	C11H22O6S	C20H14O3S	C13H18O2
C22H23NO7	C27H40O2S	C29H30O3	C12H24O6S	C26H24O3S	C18H28O2
C23H25NO7	C24H32O2S	C31H34O3	C13H26O6S	C28H28O3S	C9H10O2
C24H27NO7	C13H10O2S	C33H38O3	C14H28O6S	C29H30O3S	C10H12O2
C30H37NO7	C14H12O2S	C24H18O3	C15H30O6S	C27H24O3S	C11H14O2
C17H11NO7	C15H14O2S	C26H22O3	C16H32O6S	C41H52O3S	C17H26O2
C18H13NO7	C16H16O2S	C27H24O3	C17H34O6S	C38H42O3S	C10H10O2
C19H15NO7	C17H18O2S	C12H26O3S	C18H36O6S	C46H50O3S	C11H12O2
C20H17NO7	C18H20O2S	C8H18O3S	C19H38O6S	C46H32O3S	C12H14O2
C21H19NO7	C20H24O2S	C5H10O3S	C20H40O6S	C45H22O3S	C13H16O2
C22H21NO7	C27H38O2S	C6H12O3S	C22H44O6S	C16H20O3S2	C18H26O2
C23H23NO7	C14H10O2S	C7H14O3S	C6H12O6S	C21H24O3S2	C18H24O2
C24H25NO7	C15H12O2S	C8H16O3S	C7H14O6S	C23H28O3S2	C28H44O2
C19H13NO7	C16H14O2S	C9H18O3S	C8H16O6S	C27H34O3S2	C11H10O2
C20H15NO7	C27H36O2S	C8H14O3S	C9H18O6S	C26H18O3S2	C12H12O2
C22H19NO7	C30H42O2S	C12H20O3S	C10H18O6S	C29H22O3S2	C12H10O2
C23H21NO7	C15H10O2S	C8H12O3S	C11H20O6S	C29H20O3S2	C23H32O2

C24H23NO7	C16H12O2S	C9H14O3S	C12H22O6S	C31H24O3S2	C13H10O2
C19H11NO7	C17H14O2S	C7H10O3S	C13H24O6S	C30H20O3S2	C23H30O2
C20H13NO7	C18H16O2S	C10H14O3S	C14H26O6S	C42H40O3S2	C16H10O2
C21H15NO7	C16H10O2S	C8H10O3S	C15H28O6S	C39H24O3S2	C17H12O2
C22H17NO7	C26H30O2S	C9H12O3S	C16H30O6S	C12H26O3S3	C18H14O2
C23H19NO7	C27H32O2S	C11H14O3S	C18H34O6S	C12H24O3S3	C19H16O2
C25H23NO7	C19H14O2S	C12H16O3S	C19H36O6S	C16H32O3S3	C18H12O2
C46H65NO7	C29H34O2S	C9H10O3S	C20H38O6S	C21H18O3S3	C19H14O2
C24H21NO7	C23H16O2S	C12H14O3S	C21H40O6S	C29H32O3S3	C22H20O2
C23H17NO7	C27H12O2S	C21H28O3S	C22H42O6S	C21H16O3S3	C24H24O2
C24H19NO7	C40H38O2S	C14H14O3S	C23H44O6S	C40H46O3S3	C22H18O2
C25H21NO7	C37H12O2S	C17H20O3S	C24H46O6S	C30H24O3S3	C22H16O2
C27H25NO7	C20H28O2S2	C19H24O3S	C6H10O6S	C35H22O3S3	C25H22O2
C44H55NO7	C11H10O2S2	C20H26O3S	C7H12O6S	C36H22O3S3	C27H26O2
C25H17NO7	C12H12O2S2	C29H42O3S	C8H14O6S	C15H30O4	C28H28O2
C25H13NO7	C13H14O2S2	C30H44O3S	C9H16O6S	C16H32O4	C30H32O2
C37H27NO7	C21H28O2S2	C31H46O3S	C34H64O6S	C22H44O4	C32H36O2
C43H19NO7	C12H10O2S2	C13H10O3S	C36H68O6S	C46H92O4	C29H28O2
C10H17NO7S	C13H12O2S2	C14H12O3S	C10H16O6S	C10H20O4	C31H32O2
C15H19NO7S	C13H10O2S2	C15H14O3S	C11H18O6S	C13H26O4	C25H18O2
C16H21NO7S	C24H32O2S2	C16H16O3S	C12H20O6S	C20H40O4	C36H34O2
C16H17NO7S	C21H22O2S2	C17H18O3S	C13H22O6S	C5H10O4	C28H14O2
C19H23NO7S	C22H24O2S2	C18H20O3S	C14H24O6S	C6H12O4	C29H10O2
C17H17NO7S	C23H26O2S2	C19H22O3S	C15H26O6S	C7H14O4	C20H16O20S3
C47H75NO7S	C20H12O2S2	C20H24O3S	C17H30O6S	C8H16O4	C10H16O2S
C35H49NO7S	C24H16O2S2	C21H26O3S	C18H32O6S	C21H40O4	C11H18O2S
C31H29NO7S	C25H18O2S2	C23H30O3S	C20H36O6S	C19H36O4	C13H22O2S
C22H35NO7S2	C31H16O2S2	C25H34O3S	C23H42O6S	C20H38O4	C16H28O2S
C24H33NO7S2	C34H22O2S2	C27H38O3S	C24H44O6S	C6H10O4	C12H20O2S
C18H21NO7S2	C35H24O2S2	C32H48O3S	C25H46O6S	C7H12O4	C9H14O2S
C25H31NO7S2	C37H28O2S2	C14H10O3S	C7H10O6S	C20H36O4	C14H22O2S
C30H35NO7S2	C34H20O2S2	C15H12O3S	C8H12O6S	C21H38O4	C10H14O2S
C33H31NO7S2	C33H18O2S2	C16H14O3S	C9H14O6S	C22H40O4	C31H50O2S
C29H13NO7S2	C36H24O2S2	C17H16O3S	C14H22O6S	C24H44O4	C18H22O2S
C34H21NO7S2	C14H30O2S3	C18H18O3S	C34H62O6S	C11H18O4	C20H26O2S
C36H23NO7S2	C15H32O2S3	C21H24O3S	C11H16O6S	C17H30O4	C12H10O2S
C18H37NO7S3	C17H36O2S3	C27H36O3S	C12H18O6S	C7H10O4	C13H12O2S
C13H25NO7S3	C11H22O2S3	C29H40O3S	C13H20O6S	C8H12O4	C14H14O2S
C20H39NO7S3	C12H24O2S3	C15H10O3S	C15H24O6S	C9H14O4	C15H16O2S
C20H37NO7S3	C13H26O2S3	C16H12O3S	C16H26O6S	C11H16O4	C27H40O2S
C40H77NO7S3	C14H28O2S3	C17H14O3S	C17H28O6S	C22H38O4	C13H10O2S
C32H59NO7S3	C15H30O2S3	C18H16O3S	C18H30O6S	C24H42O4	C14H12O2S
C22H35NO7S3	C16H32O2S3	C19H18O3S	C22H38O6S	C8H10O4	C15H14O2S
C27H45NO7S3	C17H34O2S3	C19H16O3S	C8H10O6S	C9H12O4	C16H16O2S

C18H13NO7S3	C20H40O2S3	C21H20O3S	C9H12O6S	C10H14O4	C17H18O2S
C21H19NO7S3	C25H50O2S3	C19H14O3S	C10H12O6S	C12H18O4	C18H20O2S
C18H11NO7S3	C18H24O2S3	C20H16O3S	C11H14O6S	C15H24O4	C27H38O2S
C19H33NO8	C18H18O2S3	C21H18O3S	C12H16O6S	C16H26O4	C29H42O2S
C31H57NO8	C16H12O2S3	C22H20O3S	C13H18O6S	C11H14O4	C30H44O2S
C11H17NO8	C22H18O2S3	C20H14O3S	C14H20O6S	C15H22O4	C14H10O2S
C19H29NO8	C21H14O2S3	C29H20O3S	C15H22O6S	C22H36O4	C15H12O2S
C31H53NO8	C25H14O2S3	C9H18O3S2	C16H24O6S	C10H12O4	C16H14O2S
C12H15NO8	C30H22O2S3	C9H14O3S2	C17H26O6S	C12H16O4	C17H16O2S
C13H17NO8	C34H18O2S3	C11H12O3S2	C26H44O6S	C13H18O4	C18H18O2S
C14H19NO8	C36H22O2S3	C16H20O3S2	C9H10O6S	C16H24O4	C27H36O2S
C15H21NO8	C11H24O3	C17H22O3S2	C11H12O6S	C18H28O4	C29H40O2S
C16H23NO8	C12H26O3	C18H24O3S2	C12H14O6S	C9H10O4	C16H12O2S
C17H25NO8	C13H28O3	C21H28O3S2	C13H16O6S	C11H12O4	C17H14O2S
C18H27NO8	C13H26O3	C22H30O3S2	C14H18O6S	C17H24O4	C30H40O2S
C17H23NO8	C15H30O3	C23H32O3S2	C15H20O6S	C10H10O4	C33H44O2S
C27H43NO8	C19H38O3	C24H34O3S2	C16H22O6S	C12H14O4	C16H10O2S
C39H67NO8	C20H40O3	C12H10O3S2	C17H24O6S	C13H16O4	C26H30O2S
C12H13NO8	C10H20O3	C13H12O3S2	C12H12O6S	C14H18O4	C27H32O2S
C13H15NO8	C11H22O3	C25H36O3S2	C13H14O6S	C15H20O4	C19H14O2S
C14H17NO8	C12H24O3	C27H40O3S2	C15H18O6S	C16H22O4	C27H14O2S
C15H19NO8	C14H28O3	C13H10O3S2	C16H20O6S	C18H26O4	C40H40O2S
C16H21NO8	C17H34O3	C14H12O3S2	C17H22O6S	C11H10O4	C42H44O2S
C18H25NO8	C5H10O3	C15H12O3S2	C18H24O6S	C23H34O4	C31H20O2S
C19H27NO8	C6H12O3	C24H14O3S2	C19H26O6S	C25H38O4	C16H20O2S2
C20H29NO8	C7H14O3	C26H18O3S2	C15H16O6S	C13H14O4	C11H10O2S2
C31H49NO8	C8H16O3	C35H32O3S2	C16H18O6S	C21H28O4	C12H12O2S2
C13H13NO8	C13H24O3	C37H26O3S2	C18H22O6S	C23H32O4	C18H22O2S2
C14H15NO8	C15H28O3	C38H28O3S2	C18H20O6S	C25H36O4	C20H26O2S2
C15H17NO8	C22H42O3	C10H22O3S3	C31H36O6S	C19H18O4	C21H28O2S2
C16H19NO8	C6H10O3	C11H24O3S3	C39H14O6S	C17H14O4	C12H10O2S2
C17H21NO8	C14H26O3	C13H28O3S3	C13H26O6S2	C23H18O4	C13H12O2S2
C18H23NO8	C19H36O3	C14H30O3S3	C26H50O6S2	C24H20O4	C13H10O2S2
C19H25NO8	C7H12O3	C8H18O3S3	C13H20O6S2	C25H22O4	C24H32O2S2
C20H27NO8	C8H14O3	C10H20O3S3	C9H12O6S2	C23H16O4	C25H18O2S2
C21H29NO8	C9H16O3	C11H22O3S3	C12H16O6S2	C22H10O4	C38H38O2S2
C22H31NO8	C15H26O3	C12H24O3S3	C13H18O6S2	C24H14O4	C37H28O2S2
C42H71NO8	C17H30O3	C13H26O3S3	C15H22O6S2	C31H20O4	C33H18O2S2
C17H19NO8	C22H40O3	C14H28O3S3	C16H16O6S2	C31H18O4	C36H24O2S2
C27H39NO8	C11H18O3	C15H30O3S3	C19H18O6S2	C34H18O4	C13H28O2S3
C14H13NO8	C16H28O3	C8H16O3S3	C22H24O6S2	C34H16O4	C14H30O2S3
C15H15NO8	C19H34O3	C9H18O3S3	C18H12O6S2	C36H20O4	C15H32O2S3
C16H17NO8	C20H36O3	C11H20O3S3	C43H14O6S2	C37H22O4	C20H42O2S3
C18H21NO8	C7H10O3	C13H24O3S3	C12H26O6S3	C34H14O4	C12H24O2S3

C19H23NO8	C8H12O3	C14H26O3S3	C14H30O6S3	C38H22O4	C13H26O2S3
C20H25NO8	C9H14O3	C15H28O3S3	C15H32O6S3	C37H18O4	C14H28O2S3
C21H27NO8	C11H16O3	C16H30O3S3	C16H34O6S3	C35H14O4	C16H32O2S3
C22H29NO8	C12H18O3	C17H32O3S3	C15H30O6S3	C36H16O4	C20H40O2S3
C23H31NO8	C22H38O3	C16H14O3S3	C20H38O6S3	C40H18O4	C38H66O2S3
C31H45NO8	C8H10O3	C17H12O3S3	C14H24O6S3	C41H18O4	C18H24O2S3
C15H13NO8	C10H14O3	C12H26O4	C17H30O6S3	C47H30O4	C18H18O2S3
C16H15NO8	C13H20O3	C15H30O4	C11H16O6S3	C8H18O4S	C16H12O2S3
C17H17NO8	C15H24O3	C16H32O4	C10H12O6S3	C9H16O4S	C34H18O2S3
C18H19NO8	C20H34O3	C22H44O4	C24H34O6S3	C15H26O4S	C35H20O2S3
C19H21NO8	C9H12O3	C10H20O4	C26H34O6S3	C11H16O4S	C36H22O2S3
C20H23NO8	C12H16O3	C12H24O4	C28H38O6S3	C12H16O4S	C11H24O3
C21H25NO8	C13H18O3	C13H26O4	C18H14O6S3	C15H22O4S	C12H26O3
C22H27NO8	C14H20O3	C14H28O4	C21H20O6S3	C15H16O4S	C13H28O3
C23H29NO8	C16H24O3	C17H34O4	C33H28O6S3	C32H40O4S	C13H26O3
C24H31NO8	C17H26O3	C20H40O4	C34H28O6S3	C22H20O4S	C14H28O3
C44H69NO8	C18H28O3	C5H10O4	C11H22O7	C22H18O4S	C15H30O3
C15H11NO8	C10H12O3	C6H12O4	C13H26O7	C24H22O4S	C19H38O3
C16H13NO8	C11H14O3	C7H14O4	C14H28O7	C25H24O4S	C20H40O3
C17H15NO8	C15H22O3	C8H16O4	C15H30O7	C29H32O4S	C10H20O3
C18H17NO8	C20H32O3	C9H18O4	C16H32O7	C23H18O4S	C12H24O3
C19H19NO8	C9H10O3	C15H28O4	C17H34O7	C24H20O4S	C17H34O3
C20H21NO8	C10H10O3	C17H32O4	C18H36O7	C28H22O4S	C5H10O3
C21H23NO8	C11H12O3	C19H36O4	C19H38O7	C32H14O4S	C6H12O3
C22H25NO8	C13H16O3	C20H38O4	C11H20O7	C45H24O4S	C7H14O3
C23H27NO8	C14H18O3	C21H40O4	C12H22O7	C46H24O4S	C8H16O3
C24H29NO8	C15H20O3	C32H62O4	C13H24O7	C20H38O4S2	C13H24O3
C25H31NO8	C16H22O3	C6H10O4	C15H28O7	C12H18O4S2	C15H28O3
C26H33NO8	C18H26O3	C7H12O4	C16H30O7	C20H28O4S2	C22H42O3
C16H11NO8	C20H30O3	C20H36O4	C17H32O7	C18H24O4S2	C6H10O3
C17H13NO8	C23H36O3	C21H38O4	C20H38O7	C22H30O4S2	C14H26O3
C18H15NO8	C12H14O3	C22H40O4	C21H40O7	C19H22O4S2	C7H12O3
C19H17NO8	C17H24O3	C24H44O4	C22H42O7	C21H26O4S2	C9H16O3
C20H19NO8	C19H28O3	C11H18O4	C24H46O7	C25H34O4S2	C15H26O3
C21H21NO8	C16H20O3	C17H30O4	C26H50O7	C27H24O4S2	C17H30O3
C22H23NO8	C11H10O3	C19H34O4	C14H26O7	C43H22O4S2	C21H38O3
C23H25NO8	C12H12O3	C7H10O4	C18H34O7	C10H22O4S3	C22H40O3
C24H27NO8	C13H14O3	C8H12O4	C10H16O7	C11H24O4S3	C11H18O3
C25H29NO8	C14H16O3	C9H14O4	C11H18O7	C9H20O4S3	C16H28O3
C26H31NO8	C15H18O3	C11H16O4	C13H22O7	C12H18O4S3	C20H36O3
C44H65NO8	C17H22O3	C22H38O4	C14H24O7	C14H22O4S3	C7H10O3
C18H13NO8	C18H24O3	C8H10O4	C15H26O7	C16H26O4S3	C8H12O3
C19H15NO8	C19H26O3	C9H12O4	C22H40O7	C22H30O4S3	C9H14O3
C20H17NO8	C20H28O3	C10H14O4	C23H42O7	C31H36O4S3	C11H16O3

C21H19NO8	C13H12O3	C12H18O4	C24H44O7	C25H22O4S3	C12H18O3
C22H21NO8	C23H32O3	C15H24O4	C25H46O7	C36H24O4S3	C22H38O3
C23H23NO8	C25H36O3	C16H26O4	C27H50O7	C38H28O4S3	C8H10O3
C24H25NO8	C23H30O3	C17H28O4	C29H54O7	C10H22O5	C10H14O3
C25H27NO8	C13H10O3	C21H36O4	C8H12O7	C11H24O5	C13H20O3
C26H29NO8	C14H12O3	C10H12O4	C12H20O7	C9H20O5	C15H24O3
C33H41NO8	C15H14O3	C11H14O4	C16H28O7	C14H28O5	C20H34O3
C20H15NO8	C23H28O3	C15H22O4	C17H30O7	C15H30O5	C9H12O3
C21H17NO8	C27H34O3	C16H24O4	C18H32O7	C16H32O5	C12H16O3
C22H19NO8	C29H36O3	C22H36O4	C19H34O7	C17H34O5	C13H18O3
C23H21NO8	C30H38O3	C9H10O4	C20H36O7	C18H36O5	C14H20O3
C24H23NO8	C31H40O3	C12H16O4	C21H38O7	C19H38O5	C16H24O3
C25H25NO8	C32H42O3	C13H18O4	C26H48O7	C21H42O5	C17H26O3
C23H19NO8	C33H44O3	C18H28O4	C11H16O7	C23H46O5	C18H28O3
C24H21NO8	C18H12O3	C20H32O4	C12H18O7	C5H10O5	C22H36O3
C25H23NO8	C19H14O3	C11H12O4	C14H22O7	C10H20O5	C10H12O3
C27H27NO8	C23H22O3	C14H18O4	C15H24O7	C6H12O5	C11H14O3
C28H29NO8	C25H26O3	C17H24O4	C17H28O7	C8H16O5	C15H22O3
C21H13NO8	C30H36O3	C18H26O4	C18H30O7	C9H18O5	C20H32O3
C22H15NO8	C31H38O3	C20H30O4	C24H42O7	C17H32O5	C9H10O3
C23H17NO8	C32H40O3	C22H34O4	C25H44O7	C23H44O5	C10H10O3
C26H23NO8	C33H42O3	C10H10O4	C26H46O7	C24H46O5	C11H12O3
C26H21NO8	C34H44O3	C12H14O4	C10H14O7	C25H48O5	C13H16O3
C27H23NO8	C35H46O3	C13H16O4	C13H20O7	C10H18O5	C14H18O3
C32H31NO8	C27H30O3	C15H20O4	C16H26O7	C11H20O5	C15H20O3
C26H17NO8	C26H26O3	C16H22O4	C20H34O7	C16H30O5	C16H22O3
C41H39NO8	C27H28O3	C19H28O4	C21H36O7	C20H38O5	C18H26O3
C43H43NO8	C29H32O3	C11H10O4	C22H38O7	C22H42O5	C20H30O3
C41H35NO8	C31H36O3	C12H12O4	C23H40O7	C6H10O5	C23H36O3
C43H39NO8	C32H38O3	C13H14O4	C9H12O7	C7H12O5	C12H14O3
C31H13NO8	C33H40O3	C14H16O4	C14H20O7	C8H14O5	C17H24O3
C42H31NO8	C34H42O3	C15H18O4	C16H24O7	C9H16O5	C14H16O3
C14H27NO8S	C35H44O3	C19H26O4	C17H26O7	C23H42O5	C15H18O3
C32H61NO8S	C23H20O3	C21H30O4	C18H28O7	C24H44O5	C18H24O3
C32H57NO8S	C26H24O3	C23H34O4	C19H30O7	C7H10O5	C20H28O3
C27H45NO8S	C27H26O3	C25H38O4	C20H32O7	C8H12O5	C21H30O3
C17H21NO8S	C31H34O3	C27H42O4	C25H42O7	C10H16O5	C23H34O3
C21H25NO8S	C33H38O3	C16H20O4	C26H44O7	C11H18O5	C28H44O3
C20H15NO8S	C34H40O3	C17H22O4	C10H12O7	C12H20O5	C11H10O3
C23H21NO8S	C22H16O3	C18H24O4	C11H14O7	C14H24O5	C12H12O3
C25H53NO8S3	C29H30O3	C12H10O4	C12H16O7	C15H26O5	C13H14O3
C17H35NO8S3	C24H18O3	C13H12O4	C13H18O7	C16H28O5	C16H20O3
C25H51NO8S3	C25H20O3	C14H14O4	C15H22O7	C19H34O5	C17H22O3
C25H47NO8S3	C26H22O3	C15H16O4	C21H34O7	C20H36O5	C13H12O3

C31H21NO8S3	C27H24O3	C17H20O4	C22H36O7	C9H14O5	C23H32O3
C14H25NO9	C29H26O3	C19H24O4	C18H26O7	C13H20O5	C25H36O3
C16H29NO9	C12H26O3S	C21H28O4	C20H30O7	C24H42O5	C12H10O3
C16H25NO9	C5H10O3S	C23H32O4	C21H32O7	C9H12O5	C13H10O3
C15H21NO9	C6H12O3S	C25H36O4	C22H34O7	C10H14O5	C25H34O3
C17H25NO9	C7H14O3S	C27H40O4	C10H10O7	C11H16O5	C14H12O3
C14H17NO9	C8H16O3S	C16H18O4	C11H12O7	C12H18O5	C21H24O3
C15H19NO9	C9H18O3S	C14H12O4	C12H14O7	C18H30O5	C23H28O3
C16H21NO9	C11H20O3S	C25H34O4	C13H16O7	C20H34O5	C23H24O3
C17H23NO9	C10H16O3S	C13H10O4	C14H18O7	C8H10O5	C30H38O3
C18H25NO9	C12H20O3S	C15H14O4	C15H20O7	C12H16O5	C31H40O3
C19H27NO9	C9H14O3S	C16H16O4	C16H22O7	C13H18O5	C32H42O3
C15H17NO9	C10H14O3S	C15H12O4	C17H24O7	C15H22O5	C33H44O3
C16H19NO9	C8H10O3S	C14H10O4	C19H28O7	C18H28O5	C17H10O3
C17H21NO9	C9H12O3S	C16H14O4	C23H36O7	C9H10O5	C18H12O3
C18H23NO9	C11H16O3S	C16H12O4	C25H40O7	C10H12O5	C19H14O3
C19H25NO9	C10H12O3S	C17H14O4	C15H18O7	C11H14O5	C21H18O3
C20H27NO9	C11H14O3S	C24H26O4	C11H10O7	C14H20O5	C23H22O3
C21H29NO9	C9H10O3S	C25H28O4	C12H12O7	C16H24O5	C29H34O3
C22H31NO9	C10H10O3S	C18H12O4	C13H14O7	C17H26O5	C30H36O3
C23H33NO9	C13H14O3S	C19H14O4	C14H16O7	C14H18O5	C31H38O3
C25H37NO9	C21H28O3S	C20H16O4	C16H20O7	C15H20O5	C32H40O3
C15H15NO9	C14H14O3S	C21H18O4	C17H22O7	C16H22O5	C33H42O3
C16H17NO9	C17H20O3S	C23H22O4	C18H24O7	C10H10O5	C34H44O3
C17H19NO9	C19H24O3S	C25H26O4	C19H26O7	C11H12O5	C35H46O3
C18H21NO9	C18H20O3S	C26H28O4	C20H28O7	C12H14O5	C23H20O3
C19H23NO9	C29H42O3S	C27H30O4	C22H32O7	C13H16O5	C25H24O3
C20H25NO9	C30H44O3S	C29H34O4	C24H36O7	C17H24O5	C27H28O3
C21H27NO9	C31H46O3S	C30H36O4	C12H10O7	C18H26O5	C29H32O3
C22H29NO9	C13H10O3S	C31H38O4	C13H12O7	C19H28O5	C31H36O3
C23H31NO9	C15H14O3S	C32H40O4	C14H14O7	C20H30O5	C32H38O3
C16H15NO9	C16H16O3S	C33H42O4	C15H16O7	C20H28O5	C33H40O3
C17H17NO9	C17H18O3S	C34H44O4	C16H18O7	C22H32O5	C34H42O3
C18H19NO9	C19H22O3S	C35H46O4	C17H20O7	C11H10O5	C35H44O3
C19H21NO9	C20H24O3S	C36H48O4	C18H22O7	C12H12O5	C27H26O3
C20H23NO9	C21H26O3S	C21H16O4	C19H24O7	C13H14O5	C29H30O3
C21H25NO9	C23H30O3S	C22H18O4	C20H26O7	C14H16O5	C31H34O3
C22H27NO9	C27H38O3S	C24H22O4	C21H28O7	C15H18O5	C33H38O3
C23H29NO9	C32H48O3S	C25H24O4	C22H30O7	C16H20O5	C28H28O3
C24H31NO9	C31H44O3S	C26H26O4	C14H12O7	C17H22O5	C26H22O3
C17H15NO9	C14H10O3S	C27H28O4	C15H14O7	C19H24O5	C23H14O3
C18H17NO9	C15H12O3S	C29H32O4	C16H16O7	C13H12O5	C29H26O3
C19H19NO9	C16H14O3S	C30H34O4	C17H18O7	C15H16O5	C28H24O3
C20H21NO9	C17H16O3S	C31H36O4	C18H20O7	C23H30O5	C41H24O3

C21H23NO9	C18H18O3S	C32H38O4	C19H22O7	C17H16O5	C12H26O3S
C22H25NO9	C21H24O3S	C33H40O4	C20H24O7	C20H22O5	C8H18O3S
C23H27NO9	C23H28O3S	C34H42O4	C21H26O7	C17H10O5	C20H40O3S
C24H29NO9	C27H36O3S	C35H44O4	C22H28O7	C21H16O5	C22H44O3S
C25H31NO9	C29H40O3S	C36H46O4	C16H14O7	C29H30O5	C5H10O3S
C26H33NO9	C30H42O3S	C24H20O4	C17H16O7	C24H16O5	C6H12O3S
C18H15NO9	C32H46O3S	C25H22O4	C18H18O7	C24H12O5	C7H14O3S
C19H17NO9	C16H12O3S	C26H24O4	C19H20O7	C28H12O5	C8H16O3S
C20H19NO9	C17H14O3S	C27H26O4	C20H22O7	C34H24O5	C9H18O3S
C21H21NO9	C18H16O3S	C28H28O4	C21H24O7	C34H16O5	C8H14O3S
C22H23NO9	C18H14O3S	C29H30O4	C22H26O7	C35H18O5	C12H20O3S
C23H25NO9	C21H20O3S	C30H32O4	C18H16O7	C37H22O5	C21H38O3S
C24H27NO9	C32H40O3S	C31H34O4	C19H18O7	C35H16O5	C9H14O3S
C25H29NO9	C19H14O3S	C32H36O4	C20H20O7	C37H20O5	C8H10O3S
C26H31NO9	C21H18O3S	C33H38O4	C21H22O7	C37H18O5	C9H12O3S
C30H37NO9	C20H14O3S	C34H40O4	C21H20O7	C39H22O5	C11H16O3S
C19H15NO9	C26H14O3S	C35H42O4	C19H16O7	C43H28O5	C11H14O3S
C20H17NO9	C29H20O3S	C36H44O4	C20H18O7	C48H34O5	C12H16O3S
C21H19NO9	C42H40O3S	C37H46O4	C23H22O7	C22H46O5S	C18H28O3S
C22H21NO9	C9H14O3S2	C23H16O4	C25H26O7	C8H18O5S	C9H10O3S
C23H23NO9	C11H12O3S2	C24H18O4	C27H30O7	C31H62O5S	C10H10O3S
C24H25NO9	C20H28O3S2	C25H20O4	C34H20O7	C10H20O5S	C19H24O3S
C25H27NO9	C21H28O3S2	C26H22O4	C35H22O7	C11H22O5S	C14H14O3S
C26H29NO9	C12H10O3S2	C27H24O4	C33H16O7	C5H10O5S	C15H16O3S
C21H17NO9	C13H12O3S2	C28H26O4	C46H38O7	C8H16O5S	C17H20O3S
C22H19NO9	C14H14O3S2	C29H28O4	C11H24O7S3	C9H18O5S	C20H26O3S
C23H21NO9	C25H36O3S2	C31H32O4	C14H30O7S3	C9H16O5S	C29H42O3S
C24H23NO9	C27H40O3S2	C32H34O4	C9H20O7S3	C16H28O5S	C30H44O3S
C25H25NO9	C13H10O3S2	C34H38O4	C12H22O7S3	C17H30O5S	C31H46O3S
C26H27NO9	C14H12O3S2	C23H14O4	C20H38O7S3	C8H12O5S	C33H50O3S
C27H29NO9	C24H32O3S2	C25H18O4	C23H28O7S3	C9H14O5S	C13H10O3S
C28H31NO9	C27H38O3S2	C26H20O4	C25H32O7S3	C8H10O5S	C14H12O3S
C40H53NO9	C24H14O3S2	C27H22O4	C25H30O7S3	C10H12O5S	C15H14O3S
C23H19NO9	C26H18O3S2	C28H24O4	C27H34O7S3	C11H14O5S	C16H16O3S
C24H21NO9	C24H12O3S2	C29H26O4	C19H38O8	C11H12O5S	C17H18O3S
C25H23NO9	C37H36O3S2	C24H14O4	C21H42O8	C16H16O5S	C18H20O3S
C27H27NO9	C33H18O3S2	C39H26O4	C14H26O8	C17H16O5S	C19H22O3S
C44H49NO9	C36H24O3S2	C8H18O4S	C17H32O8	C18H18O5S	C20H24O3S
C42H43NO9	C37H26O3S2	C9H20O4S	C18H34O8	C19H20O5S	C21H26O3S
C42H39NO9	C10H22O3S3	C6H12O4S	C19H36O8	C27H32O5S	C23H30O3S
C32H13NO9	C11H24O3S3	C7H14O4S	C21H40O8	C28H34O5S	C27H38O3S
C49H39NO9	C13H28O3S3	C9H18O4S	C22H42O8	C28H32O5S	C32H48O3S
C43H21NO9	C15H32O3S3	C11H22O4S	C14H24O8	C36H48O5S	C31H44O3S
C11H17NOS	C16H34O3S3	C19H38O4S	C15H26O8	C23H20O5S	C36H54O3S

C11H15NOS	C8H18O3S3	C5H10O4S	C16H28O8	C30H28O5S	C14H10O3S
C19H25NOS	C12H26O3S3	C8H16O4S	C17H30O8	C37H42O5S	C15H12O3S
C29H45NOS	C10H20O3S3	C10H18O4S	C18H32O8	C42H50O5S	C16H14O3S
C16H17NOS	C11H22O3S3	C18H34O4S	C21H38O8	C30H20O5S	C17H16O3S
C20H21NOS	C12H24O3S3	C8H14O4S	C22H40O8	C30H12O5S	C18H18O3S
C29H37NOS	C13H26O3S3	C10H16O4S	C23H42O8	C10H18O5S2	C19H20O3S
C17H11NOS	C14H28O3S3	C11H18O4S	C24H44O8	C11H20O5S2	C20H22O3S
C22H19NOS	C15H30O3S3	C12H20O4S	C20H36O8	C17H24O5S2	C21H24O3S
C34H39NOS	C16H32O3S3	C21H38O4S	C13H20O8	C18H24O5S2	C22H26O3S
C37H33NOS	C8H16O3S3	C8H12O4S	C14H22O8	C19H26O5S2	C27H36O3S
C7H15NOS2	C9H18O3S3	C9H14O4S	C15H24O8	C12H12O5S2	C29H40O3S
C18H33NOS2	C11H20O3S3	C7H10O4S	C16H26O8	C20H16O5S2	C30H42O3S
C13H21NOS2	C13H24O3S3	C10H14O4S	C18H30O8	C22H20O5S2	C32H46O3S
C28H47NOS2	C14H26O3S3	C11H16O4S	C24H42O8	C24H24O5S2	C16H12O3S
C40H71NOS2	C15H28O3S3	C19H32O4S	C12H18O8	C18H12O5S2	C17H14O3S
C16H19NOS2	C16H30O3S3	C8H10O4S	C17H28O8	C36H32O5S2	C18H16O3S
C28H43NOS2	C17H32O3S3	C9H12O4S	C19H32O8	C32H24O5S2	C19H18O3S
C28H39NOS2	C23H44O3S3	C10H12O4S	C20H34O8	C36H24O5S2	C29H38O3S
C30H43NOS2	C9H16O3S3	C11H14O4S	C22H38O8	C49H38O5S2	C19H16O3S
C31H45NOS2	C13H22O3S3	C12H16O4S	C10H12O8	C43H20O5S2	C21H20O3S
C47H67NOS2	C16H14O3S3	C15H22O4S	C16H24O8	C10H22O5S3	C31H38O3S
C37H21NOS2	C17H12O3S3	C18H28O4S	C17H26O8	C11H24O5S3	C32H40O3S
C37H19NOS2	C31H40O3S3	C9H10O4S	C19H30O8	C12H26O5S3	C19H14O3S
C10H21NOS3	C22H20O3S3	C10H10O4S	C20H32O8	C15H32O5S3	C20H16O3S
C12H25NOS3	C22H10O3S3	C11H12O4S	C22H36O8	C16H34O5S3	C21H18O3S
C15H31NOS3	C23H12O3S3	C12H14O4S	C24H40O8	C23H48O5S3	C33H40O3S
C7H15NOS3	C11H24O4	C13H16O4S	C11H14O8	C8H18O5S3	C20H14O3S
C8H17NOS3	C12H26O4	C12H12O4S	C12H16O8	C12H24O5S3	C22H18O3S
C9H19NOS3	C22H46O4	C17H20O4S	C13H18O8	C23H46O5S3	C27H14O3S
C21H41NOS3	C15H30O4	C28H40O4S	C14H20O8	C15H26O5S3	C39H36O3S
C15H27NOS3	C16H32O4	C15H14O4S	C15H22O8	C13H20O5S3	C9H18O3S2
C30H49NOS3	C22H44O4	C16H16O4S	C18H28O8	C17H24O5S3	C9H14O3S2
C40H63NOS3	C36H72O4	C17H18O4S	C20H30O8	C18H20O5S3	C11H12O3S2
C15H30O	C10H20O4	C18H20O4S	C22H34O8	C19H18O5S3	C17H22O3S2
C17H34O	C12H24O4	C19H22O4S	C12H14O8	C22H22O5S3	C20H26O3S2
C11H20O	C13H26O4	C21H26O4S	C13H16O8	C28H32O5S3	C22H30O3S2
C10H18O	C14H28O4	C27H38O4S	C14H18O8	C29H34O5S3	C25H36O3S2
C12H22O	C17H34O4	C29H42O4S	C15H20O8	C24H22O5S3	C27H40O3S2
C7H12O	C19H38O4	C29H40O4S	C16H22O8	C42H40O5S3	C29H44O3S2
C8H14O	C20H40O4	C31H44O4S	C17H24O8	C39H34O5S3	C12H10O3S2
C9H16O	C5H10O4	C16H14O4S	C18H26O8	C38H28O5S3	C13H12O3S2
C18H32O	C6H12O4	C17H16O4S	C19H28O8	C38H26O5S3	C14H14O3S2
C10H16O	C7H14O4	C18H18O4S	C21H32O8	C12H26O6	C13H10O3S2
C11H18O	C8H16O4	C19H20O4S	C24H38O8	C13H28O6	C14H12O3S2

C7H10O	C9H18O4	C20H22O4S	C24H36O8	C15H32O6	C25H34O3S2
C8H12O	C24H46O4	C21H24O4S	C13H14O8	C9H20O6	C27H38O3S2
C9H14O	C15H28O4	C27H36O4S	C14H16O8	C11H22O6	C15H12O3S2
C14H22O	C17H32O4	C28H38O4S	C15H18O8	C13H26O6	C17H16O3S2
C15H24O	C19H36O4	C30H42O4S	C16H20O8	C15H30O6	C24H14O3S2
C11H16O	C20H38O4	C16H12O4S	C17H22O8	C16H32O6	C26H18O3S2
C8H10O	C21H40O4	C18H16O4S	C18H24O8	C17H34O6	C10H22O3S3
C9H12O	C22H42O4	C19H18O4S	C19H26O8	C5H10O6	C13H28O3S3
C13H18O	C32H62O4	C20H20O4S	C20H28O8	C8H16O6	C11H24O3S3
C18H28O	C6H10O4	C27H34O4S	C21H30O8	C17H32O6	C8H18O3S3
C10H12O	C7H12O4	C29H38O4S	C22H32O8	C19H36O6	C10H20O3S3
C11H14O	C20H36O4	C30H40O4S	C13H12O8	C20H38O6	C11H22O3S3
C32H46O	C21H38O4	C18H14O4S	C14H14O8	C21H40O6	C14H28O3S3
C17H12O	C22H40O4	C19H16O4S	C15H16O8	C6H10O6	C16H32O3S3
C17H10O	C23H42O4	C20H18O4S	C16H18O8	C7H12O6	C8H16O3S3
C18H12O	C24H44O4	C22H20O4S	C17H20O8	C9H16O6	C9H18O3S3
C19H14O	C11H18O4	C23H18O4S	C18H22O8	C10H18O6	C11H20O3S3
C26H26O	C17H30O4	C10H18O4S2	C19H24O8	C11H20O6	C13H24O3S3
C27H28O	C19H34O4	C7H10O4S2	C20H26O8	C12H22O6	C15H28O3S3
C23H18O	C7H10O4	C9H14O4S2	C21H28O8	C14H26O6	C16H30O3S3
C26H24O	C8H12O4	C8H12O4S2	C22H30O8	C15H28O6	C25H48O3S3
C27H26O	C9H14O4	C10H14O4S2	C15H14O8	C16H30O6	C30H58O3S3
C26H22O	C11H16O4	C12H18O4S2	C16H16O8	C18H34O6	C9H16O3S3
C27H24O	C22H38O4	C16H26O4S2	C17H18O8	C22H42O6	C13H22O3S3
C23H14O	C23H40O4	C15H22O4S2	C18H20O8	C8H14O6	C40H74O3S3
C24H14O	C24H42O4	C18H24O4S2	C19H22O8	C10H16O6	C15H18O3S3
C34H26O	C8H10O4	C22H30O4S2	C20H24O8	C11H18O6	C16H14O3S3
C40H28O	C9H12O4	C23H32O4S2	C21H26O8	C22H40O6	C17H12O3S3
C38H22O	C10H14O4	C12H10O4S2	C22H28O8	C23H42O6	C31H40O3S3
C40H26O	C12H18O4	C13H12O4S2	C24H32O8	C24H44O6	C11H24O4
C39H20O	C15H24O4	C14H14O4S2	C16H14O8	C12H20O6	C12H26O4
C40H22O	C16H26O4	C24H34O4S2	C17H16O8	C13H22O6	C14H30O4
C40H20O	C17H28O4	C26H38O4S2	C18H18O8	C17H30O6	C22H46O4
C39H14O	C21H36O4	C13H10O4S2	C19H20O8	C18H32O6	C15H30O4
C19H40O10	C10H12O4	C14H12O4S2	C20H22O8	C7H10O6	C16H32O4
C20H42O10	C11H14O4	C26H36O4S2	C21H24O8	C8H12O6	C22H44O4
C21H44O10	C15H22O4	C33H48O4S2	C22H26O8	C9H14O6	C10H20O4
C22H46O10	C16H24O4	C36H22O4S2	C23H28O8	C10H14O6	C12H24O4
C23H48O10	C22H36O4	C10H22O4S3	C24H30O8	C16H26O6	C13H26O4
C33H68O10	C9H10O4	C11H24O4S3	C25H32O8	C11H16O6	C14H28O4
C11H22O10	C12H16O4	C12H26O4S3	C18H16O8	C12H18O6	C17H34O4
C14H28O10	C13H18O4	C14H30O4S3	C19H18O8	C13H20O6	C19H38O4
C15H30O10	C18H28O4	C15H32O4S3	C20H20O8	C14H22O6	C20H40O4
C16H32O10	C20H32O4	C17H36O4S3	C21H22O8	C15H24O6	C5H10O4

C17H34O10	C11H12O4	C9H20O4S3	C22H24O8	C18H30O6	C6H12O4
C18H36O10	C14H18O4	C10H20O4S3	C20H16O8	C8H10O6	C7H14O4
C19H38O10	C17H24O4	C11H22O4S3	C22H20O8	C9H12O6	C8H16O4
C20H40O10	C18H26O4	C12H24O4S3	C24H24O8	C13H18O6	C9H18O4
C21H42O10	C19H28O4	C13H26O4S3	C33H16O8	C14H20O6	C21H40O4
C22H44O10	C20H30O4	C17H34O4S3	C42H18O8	C16H24O6	C23H44O4
C23H46O10	C22H34O4	C8H16O4S3	C45H24O8	C18H28O6	C24H46O4
C24H48O10	C10H10O4	C20H40O4S3	C28H58O8S	C22H36O6	C15H28O4
C12H22O10	C12H14O4	C11H20O4S3	C20H42O8S	C10H12O6	C17H32O4
C15H28O10	C13H16O4	C12H22O4S3	C22H46O8S	C11H14O6	C19H36O4
C16H30O10	C15H20O4	C13H24O4S3	C28H56O8S	C12H16O6	C20H38O4
C17H32O10	C16H22O4	C14H26O4S3	C30H60O8S	C15H22O6	C22H42O4
C18H34O10	C11H10O4	C15H28O4S3	C12H24O8S	C17H26O6	C6H10O4
C19H36O10	C12H12O4	C16H30O4S3	C13H26O8S	C20H32O6	C7H12O4
C20H38O10	C13H14O4	C17H32O4S3	C18H36O8S	C9H10O6	C20H36O4
C21H40O10	C14H16O4	C14H24O4S3	C20H40O8S	C15H20O6	C21H38O4
C22H42O10	C15H18O4	C16H28O4S3	C22H44O8S	C16H22O6	C22H40O4
C23H44O10	C19H26O4	C14H22O4S3	C10H18O8S	C20H30O6	C24H44O4
C24H46O10	C21H30O4	C20H22O4S3	C24H46O8S	C34H58O6	C11H18O4
C16H28O10	C23H34O4	C22H26O4S3	C11H20O8S	C10H10O6	C17H30O4
C17H30O10	C25H38O4	C18H16O4S3	C12H22O8S	C11H12O6	C19H34O4
C18H32O10	C27H42O4	C19H18O4S3	C13H24O8S	C12H14O6	C7H10O4
C19H34O10	C16H20O4	C17H12O4S3	C14H26O8S	C13H16O6	C8H12O4
C20H36O10	C17H22O4	C19H16O4S3	C15H28O8S	C14H18O6	C9H14O4
C21H38O10	C18H24O4	C31H40O4S3	C16H30O8S	C17H24O6	C11H16O4
C22H40O10	C20H28O4	C37H20O4S3	C17H32O8S	C18H26O6	C22H38O4
C23H42O10	C13H12O4	C10H22O5	C18H34O8S	C19H28O6	C24H42O4
C24H44O10	C14H14O4	C11H24O5	C20H38O8S	C12H12O6	C8H10O4
C25H46O10	C15H16O4	C14H30O5	C21H40O8S	C17H22O6	C9H12O4
C16H26O10	C19H24O4	C9H20O5	C22H42O8S	C36H60O6	C10H14O4
C17H28O10	C23H32O4	C14H28O5	C9H16O8S	C11H10O6	C12H18O4
C18H30O10	C25H36O4	C15H30O5	C10H16O8S	C13H14O6	C15H24O4
C19H32O10	C27H40O4	C16H32O5	C11H18O8S	C14H16O6	C16H26O4
C20H34O10	C12H10O4	C17H34O5	C12H20O8S	C15H18O6	C17H28O4
C21H36O10	C16H18O4	C18H36O5	C13H22O8S	C16H20O6	C21H36O4
C22H38O10	C17H20O4	C19H38O5	C14H24O8S	C18H24O6	C10H12O4
C23H40O10	C14H12O4	C23H46O5	C15H26O8S	C19H26O6	C11H14O4
C24H42O10	C18H20O4	C5H10O5	C16H28O8S	C20H28O6	C15H22O4
C25H44O10	C23H30O4	C10H20O5	C18H32O8S	C36H58O6	C16H24O4
C14H22O10	C25H34O4	C11H22O5	C20H36O8S	C12H10O6	C21H34O4
C11H14O10	C13H10O4	C12H24O5	C22H40O8S	C13H12O6	C22H36O4
C13H18O10	C15H14O4	C20H40O5	C10H14O8S	C15H16O6	C9H10O4
C14H20O10	C16H16O4	C6H12O5	C11H16O8S	C16H18O6	C12H16O4
C15H22O10	C17H18O4	C7H14O5	C12H18O8S	C17H20O6	C13H18O4

C16H24O10	C19H22O4	C8H16O5	C13H20O8S	C17H18O6	C18H28O4
C17H26O10	C18H18O4	C9H18O5	C14H22O8S	C16H16O6	C20H32O4
C18H28O10	C23H28O4	C17H32O5	C15H24O8S	C18H20O6	C11H12O4
C19H30O10	C15H12O4	C23H44O5	C18H30O8S	C16H14O6	C14H18O4
C20H32O10	C16H14O4	C24H46O5	C20H34O8S	C17H16O6	C17H24O4
C21H34O10	C17H16O4	C25H48O5	C13H18O8S	C26H28O6	C18H26O4
C22H36O10	C15H10O4	C10H18O5	C14H20O8S	C21H16O6	C19H28O4
C23H38O10	C18H16O4	C11H20O5	C15H22O8S	C23H18O6	C20H30O4
C24H40O10	C24H26O4	C15H28O5	C16H24O8S	C24H20O6	C10H10O4
C25H42O10	C25H28O4	C16H30O5	C17H26O8S	C24H18O6	C12H14O4
C26H44O10	C26H30O4	C19H36O5	C11H12O8S	C28H14O6	C13H16O4
C27H46O10	C18H12O4	C20H38O5	C13H16O8S	C31H12O6	C15H20O4
C28H48O10	C25H26O4	C22H42O5	C14H18O8S	C37H22O6	C16H22O4
C9H10O10	C26H28O4	C6H10O5	C15H20O8S	C35H16O6	C11H10O4
C14H18O10	C27H30O4	C7H12O5	C16H22O8S	C37H20O6	C12H12O4
C15H20O10	C29H34O4	C8H14O5	C14H16O8S	C39H24O6	C13H14O4
C16H22O10	C30H36O4	C9H16O5	C16H20O8S	C33H12O6	C14H16O4
C17H24O10	C31H38O4	C23H42O5	C20H26O8S	C10H20O6S	C15H18O4
C18H26O10	C32H40O4	C24H44O5	C18H20O8S	C11H22O6S	C19H26O4
C19H28O10	C33H42O4	C25H46O5	C27H16O8S	C12H24O6S	C20H28O4
C21H32O10	C34H44O4	C8H12O5	C25H52O8S3	C16H32O6S	C21H30O4
C22H34O10	C35H46O4	C10H16O5	C27H52O8S3	C18H36O6S	C23H34O4
C23H36O10	C36H48O4	C11H18O5	C22H24O8S3	C20H40O6S	C25H38O4
C24H38O10	C21H18O4	C12H20O5	C25H30O8S3	C22H44O6S	C27H42O4
C25H40O10	C21H16O4	C14H24O5	C26H32O8S3	C6H12O6S	C16H20O4
C26H42O10	C22H18O4	C15H26O5	C19H16O8S3	C7H14O6S	C17H22O4
C27H44O10	C24H22O4	C16H28O5	C9H18O9	C8H16O6S	C18H24O4
C30H50O10	C25H24O4	C19H34O5	C10H14O9	C9H18O6S	C13H12O4
C32H54O10	C26H26O4	C20H36O5	C17H28O9	C11H20O6S	C14H14O4
C33H56O10	C27H28O4	C21H38O5	C15H24O9	C12H22O6S	C15H16O4
C13H16O10	C29H32O4	C22H40O5	C18H30O9	C18H34O6S	C16H18O4
C20H30O10	C30H34O4	C7H10O5	C20H34O9	C6H10O6S	C17H20O4
C28H46O10	C31H36O4	C9H14O5	C18H28O9	C8H14O6S	C19H24O4
C29H48O10	C32H38O4	C13H20O5	C15H22O9	C9H16O6S	C20H26O4
C12H12O10	C33H40O4	C23H40O5	C16H24O9	C14H24O6S	C21H28O4
C19H26O10	C34H42O4	C24H42O5	C17H26O9	C18H32O6S	C23H32O4
C20H28O10	C35H44O4	C8H10O5	C11H12O9	C20H36O6S	C25H36O4
C21H30O10	C36H46O4	C9H12O5	C13H16O9	C11H16O6S	C27H40O4
C22H32O10	C37H48O4	C10H14O5	C14H18O9	C14H22O6S	C12H10O4
C23H34O10	C28H30O4	C11H16O5	C24H38O9	C23H40O6S	C18H20O4
C24H36O10	C24H20O4	C12H18O5	C15H20O9	C9H12O6S	C21H26O4
C26H40O10	C25H22O4	C17H28O5	C16H22O9	C17H22O6S	C23H30O4
C27H42O10	C28H28O4	C18H30O5	C17H24O9	C16H16O6S	C25H34O4
C28H44O10	C29H30O4	C20H34O5	C18H26O9	C34H50O6S	C27H38O4

C32H52O10	C30H32O4	C21H36O5	C19H28O9	C31H36O6S	C14H12O4
C33H54O10	C31H34O4	C10H12O5	C20H30O9	C27H26O6S	C15H14O4
C13H14O10	C32H36O4	C11H14O5	C22H34O9	C23H18O6S	C16H16O4
C14H16O10	C33H38O4	C12H16O5	C24H36O9	C25H22O6S	C18H18O4
C15H18O10	C34H40O4	C13H18O5	C26H40O9	C27H16O6S	C23H28O4
C16H20O10	C35H42O4	C15H22O5	C14H16O9	C23H38O6S2	C25H32O4
C17H22O10	C36H44O4	C18H28O5	C15H18O9	C30H28O6S2	C14H10O4
C18H24O10	C37H46O4	C21H34O5	C16H20O9	C33H26O6S2	C15H12O4
C31H50O10	C27H26O4	C9H10O5	C17H22O9	C38H28O6S2	C16H14O4
C21H28O10	C23H16O4	C14H20O5	C18H24O9	C47H36O6S2	C22H22O4
C22H30O10	C24H18O4	C16H24O5	C19H26O9	C10H22O6S3	C23H24O4
C24H34O10	C25H20O4	C17H26O5	C20H28O9	C12H26O6S3	C18H12O4
C25H36O10	C26H22O4	C19H30O5	C21H30O9	C13H28O6S3	C19H14O4
C26H38O10	C27H24O4	C22H36O5	C22H32O9	C14H30O6S3	C20H16O4
C14H14O10	C28H26O4	C11H12O5	C23H34O9	C15H32O6S3	C23H22O4
C15H16O10	C29H28O4	C12H14O5	C15H16O9	C20H42O6S3	C24H24O4
C16H18O10	C31H32O4	C13H16O5	C16H18O9	C15H30O6S3	C25H26O4
C17H20O10	C32H34O4	C14H18O5	C17H20O9	C19H38O6S3	C26H28O4
C18H22O10	C34H38O4	C15H20O5	C18H22O9	C14H26O6S3	C27H30O4
C19H24O10	C25H18O4	C16H22O5	C19H24O9	C8H14O6S3	C28H32O4
C20H26O10	C26H20O4	C17H24O5	C20H26O9	C14H24O6S3	C30H36O4
C23H32O10	C27H22O4	C18H26O5	C21H28O9	C19H34O6S3	C31H38O4
C27H40O10	C29H26O4	C19H28O5	C22H30O9	C22H38O6S3	C32H40O4
C23H30O10	C24H14O4	C20H30O5	C23H32O9	C23H40O6S3	C33H42O4
C14H12O10	C35H22O4	C10H10O5	C16H16O9	C25H38O6S3	C34H44O4
C15H14O10	C8H18O4S	C11H10O5	C17H18O9	C21H26O6S3	C35H46O4
C16H16O10	C9H20O4S	C12H12O5	C18H20O9	C30H40O6S3	C21H16O4
C17H18O10	C11H22O4S	C13H14O5	C19H22O9	C35H44O6S3	C22H18O4
C18H20O10	C6H12O4S	C14H16O5	C20H24O9	C24H22O6S3	C23H20O4
C19H22O10	C7H14O4S	C15H18O5	C21H26O9	C29H20O6S3	C24H22O4
C20H24O10	C8H16O4S	C16H20O5	C22H28O9	C10H20O7	C25H24O4
C21H26O10	C9H18O4S	C17H22O5	C24H32O9	C11H22O7	C26H26O4
C22H28O10	C19H38O4S	C18H24O5	C18H18O9	C12H24O7	C27H28O4
C25H34O10	C5H10O4S	C19H26O5	C19H20O9	C13H26O7	C29H32O4
C26H36O10	C10H18O4S	C20H28O5	C20H22O9	C14H28O7	C30H34O4
C15H12O10	C18H34O4S	C21H30O5	C21H24O9	C8H16O7	C31H36O4
C16H14O10	C10H16O4S	C22H32O5	C22H26O9	C9H18O7	C32H38O4
C17H16O10	C11H18O4S	C23H34O5	C20H20O9	C6H12O7	C33H40O4
C18H18O10	C12H20O4S	C12H10O5	C21H22O9	C7H14O7	C34H42O4
C19H20O10	C14H24O4S	C13H12O5	C23H26O9	C10H18O7	C35H44O4
C20H22O10	C21H38O4S	C14H14O5	C24H28O9	C11H20O7	C36H46O4
C21H24O10	C23H42O4S	C15H16O5	C16H10O9	C13H24O7	C37H48O4
C22H26O10	C9H14O4S	C16H18O5	C21H20O9	C15H28O7	C28H30O4
C23H28O10	C18H32O4S	C17H20O5	C26H54O9S	C16H30O7	C22H16O4

C24H30O10	C19H34O4S	C18H22O5	C27H56O9S	C21H40O7	C24H20O4
C25H32O10	C8H12O4S	C19H24O5	C22H46O9S	C8H14O7	C25H22O4
C27H36O10	C10H14O4S	C23H32O5	C26H52O9S	C6H10O7	C26H24O4
C16H12O10	C11H16O4S	C20H26O5	C28H56O9S	C7H12O7	C27H26O4
C17H14O10	C9H12O4S	C14H12O5	C29H58O9S	C9H16O7	C28H28O4
C18H16O10	C14H22O4S	C15H14O5	C20H40O9S	C10H16O7	C29H30O4
C19H18O10	C19H32O4S	C16H16O5	C27H52O9S	C11H18O7	C30H32O4
C20H20O10	C8H10O4S	C19H22O5	C29H56O9S	C13H22O7	C31H34O4
C21H22O10	C10H12O4S	C21H26O5	C37H72O9S	C14H24O7	C32H36O4
C22H24O10	C11H14O4S	C23H30O5	C13H24O9S	C15H26O7	C33H38O4
C23H26O10	C12H16O4S	C25H34O5	C18H34O9S	C22H40O7	C34H40O4
C24H28O10	C13H18O4S	C13H10O5	C20H38O9S	C24H44O7	C35H42O4
C25H30O10	C14H20O4S	C17H18O5	C22H42O9S	C8H12O7	C36H44O4
C26H32O10	C15H22O4S	C18H20O5	C37H70O9S	C9H14O7	C37H46O4
C27H34O10	C18H28O4S	C17H16O5	C39H74O9S	C12H20O7	C22H14O4
C18H14O10	C9H10O4S	C27H36O5	C18H32O9S	C16H28O7	C23H16O4
C19H16O10	C11H12O4S	C14H10O5	C20H36O9S	C17H30O7	C24H18O4
C20H18O10	C12H14O4S	C15H12O5	C22H40O9S	C18H32O7	C25H20O4
C21H20O10	C13H16O4S	C16H14O5	C23H42O9S	C20H36O7	C26H22O4
C22H22O10	C11H10O4S	C18H18O5	C12H16O9S	C21H38O7	C29H28O4
C23H24O10	C12H12O4S	C20H22O5	C16H24O9S	C7H10O7	C31H32O4
C24H26O10	C12H10O4S	C21H24O5	C16H22O9S	C26H46O7	C32H34O4
C25H28O10	C15H16O4S	C31H42O5	C19H24O9S	C10H14O7	C33H36O4
C26H30O10	C17H20O4S	C16H12O5	C18H16O9S	C11H16O7	C34H38O4
C27H32O10	C28H40O4S	C17H14O5	C23H12O9S	C12H18O7	C28H26O4
C28H34O10	C15H14O4S	C18H16O5	C26H18O9S	C13H20O7	C23H14O4
C31H40O10	C16H16O4S	C19H18O5	C47H26O9S2	C14H22O7	C25H18O4
C18H12O10	C17H18O4S	C20H20O5	C12H22OS	C15H24O7	C26H20O4
C19H14O10	C18H20O4S	C21H20O5	C24H44OS	C17H28O7	C29H26O4
C20H16O10	C27H38O4S	C22H22O5	C26H48OS	C18H30O7	C23H12O4
C21H18O10	C16H14O4S	C18H14O5	C24H42OS	C20H34O7	C24H14O4
C22H20O10	C17H16O4S	C19H16O5	C17H26OS	C8H10O7	C27H20O4
C23H22O10	C18H18O4S	C21H18O5	C23H38OS	C9H12O7	C39H26O4
C24H24O10	C19H20O4S	C22H20O5	C28H48OS	C16H24O7	C8H18O4S
C25H26O10	C20H22O4S	C24H24O5	C24H38OS	C18H28O7	C9H20O4S
C26H28O10	C21H24O4S	C25H26O5	C12H10OS	C20H32O7	C16H34O4S
C27H30O10	C27H36O4S	C26H28O5	C15H16OS	C26H44O7	C11H22O4S
C21H16O10	C29H40O4S	C27H30O5	C16H18OS	C10H12O7	C21H42O4S
C22H18O10	C30H42O4S	C28H32O5	C18H22OS	C11H14O7	C6H12O4S
C23H20O10	C16H12O4S	C19H12O5	C19H24OS	C12H16O7	C7H14O4S
C24H22O10	C33H46O4S	C20H14O5	C25H36OS	C13H18O7	C9H18O4S
C25H24O10	C18H16O4S	C21H16O5	C36H56OS	C14H20O7	C10H20O4S
C26H26O10	C19H18O4S	C22H18O5	C15H14OS	C15H22O7	C19H38O4S
C28H30O10	C21H22O4S	C23H20O5	C27H38OS	C17H26O7	C5H10O4S

C22H16O10	C27H34O4S	C24H22O5	C14H10OS	C9H10O7	C8H16O4S
C23H18O10	C29H38O4S	C25H24O5	C34H48OS	C12H14O7	C21H40O4S
C24H20O10	C18H14O4S	C26H26O5	C21H20OS	C21H32O7	C10H18O4S
C25H22O10	C19H16O4S	C27H28O5	C28H30OS	C23H36O7	C12H22O4S
C26H24O10	C20H18O4S	C28H30O5	C20H12OS	C24H38O7	C18H34O4S
C27H26O10	C21H20O4S	C29H32O5	C42H56OS	C11H12O7	C19H36O4S
C28H28O10	C21H18O4S	C31H36O5	C32H24OS	C13H16O7	C6H10O4S
C24H18O10	C30H34O4S	C33H40O5	C19H36OS2	C14H18O7	C8H14O4S
C34H28O10	C10H18O4S2	C24H20O5	C15H20OS2	C15H20O7	C10H16O4S
C25H50O10S	C8H14O4S2	C25H22O5	C12H12OS2	C16H22O7	C11H18O4S
C25H48O10S	C9H14O4S2	C26H24O5	C13H14OS2	C17H24O7	C12H20O4S
C20H36O10S	C7H10O4S2	C27H26O5	C22H32OS2	C18H26O7	C21H38O4S
C23H40O10S	C8H12O4S2	C28H28O5	C13H12OS2	C15H18O7	C23H42O4S
C14H20O10S	C10H14O4S2	C29H30O5	C24H34OS2	C16H20O7	C18H32O4S
C15H22O10S	C12H18O4S2	C30H32O5	C16H16OS2	C17H22O7	C19H34O4S
C16H24O10S	C16H26O4S2	C31H34O5	C18H20OS2	C23H34O7	C7H10O4S
C14H18O10S	C10H12O4S2	C32H36O5	C20H24OS2	C13H14O7	C8H12O4S
C15H20O10S	C17H24O4S2	C33H38O5	C27H38OS2	C14H16O7	C9H14O4S
C16H22O10S	C12H12O4S2	C34H40O5	C29H42OS2	C18H24O7	C10H14O4S
C17H24O10S	C13H14O4S2	C35H42O5	C24H32OS2	C16H18O7	C11H16O4S
C15H18O10S	C14H16O4S2	C36H44O5	C24H30OS2	C24H34O7	C14H22O4S
C17H22O10S	C22H30O4S2	C23H16O5	C20H20OS2	C26H38O7	C15H24O4S
C18H24O10S	C12H10O4S2	C24H18O5	C16H12OS2	C36H58O7	C18H30O4S
C15H16O10S	C14H14O4S2	C25H20O5	C26H32OS2	C38H62O7	C19H32O4S
C16H18O10S	C24H34O4S2	C26H22O5	C27H34OS2	C13H12O7	C8H10O4S
C17H20O10S	C26H38O4S2	C27H24O5	C20H18OS2	C14H14O7	C9H12O4S
C18H22O10S	C14H12O4S2	C28H26O5	C22H22OS2	C15H16O7	C10H12O4S
C19H24O10S	C26H36O4S2	C29H28O5	C24H26OS2	C17H20O7	C11H14O4S
C20H26O10S	C16H14O4S2	C30H30O5	C49H76OS2	C18H22O7	C12H16O4S
C16H16O10S	C16H10O4S2	C31H32O5	C18H14OS2	C19H24O7	C14H20O4S
C17H18O10S	C17H12O4S2	C32H34O5	C21H20OS2	C36H56O7	C15H22O4S
C18H20O10S	C10H22O4S3	C33H36O5	C26H28OS2	C16H16O7	C18H28O4S
C19H22O10S	C11H24O4S3	C34H38O5	C37H46OS2	C17H18O7	C29H50O4S
C20H24O10S	C12H26O4S3	C35H40O5	C35H18OS2	C18H20O7	C9H10O4S
C21H26O10S	C13H28O4S3	C23H14O5	C17H36OS3	C27H36O7	C10H10O4S
C22H28O10S	C14H30O4S3	C24H16O5	C23H46OS3	C16H14O7	C11H12O4S
C19H20O10S	C15H32O4S3	C25H18O5	C35H70OS3	C18H18O7	C12H14O4S
C20H22O10S	C16H34O4S3	C26H20O5	C26H48OS3	C20H14O7	C14H18O4S
C21H24O10S	C17H36O4S3	C27H22O5	C37H70OS3	C22H18O7	C15H20O4S
C23H28O10S	C18H38O4S3	C28H24O5	C17H14OS3	C24H20O7	C11H10O4S
C19H18O10S	C22H46O4S3	C23H12O5	C25H16OS3	C35H40O7	C15H16O4S
C20H20O10S	C24H50O4S3	C25H16O5	C33H18OS3	C25H18O7	C17H20O4S
C20H12O10S	C9H20O4S3	C26H18O5	C36H24OS3	C23H12O7	C28H40O4S
C28H12O10S	C20H42O4S3	C27H20O5	C22H14N2O10S	C31H12O7	C15H14O4S

C12H26O11	C10H20O4S3	C28H22O5	C30H12N2O10S	C37H22O7	C16H16O4S
C14H30O11	C11H22O4S3	C25H12O5	C32H14N2O10S3	C39H26O7	C17H18O4S
C21H44O11	C12H24O4S3	C8H18O5S	C25H32N2O11	C39H22O7	C18H20O4S
C22H46O11	C13H26O4S3	C9H20O5S	C17H14N2O11	C42H20O7	C19H22O4S
C12H24O11	C15H30O4S3	C10H20O5S	C25H30N2O11	C18H38O7S	C22H28O4S
C13H26O11	C17H34O4S3	C11H22O5S	C24H22N2O11	C10H20O7S	C24H32O4S
C14H28O11	C22H44O4S3	C12H24O5S	C26H16N2O11S3	C18H36O7S	C27H38O4S
C16H32O11	C24H48O4S3	C5H10O5S	C16H16N2O12	C6H12O7S	C29H42O4S
C17H34O11	C8H16O4S3	C6H12O5S	C25H18N2O12	C10H18O7S	C15H12O4S
C18H36O11	C20H40O4S3	C7H14O5S	C28H24N2O12	C11H20O7S	C16H14O4S
C19H38O11	C11H20O4S3	C8H16O5S	C23H16N2O12S	C14H26O7S	C17H16O4S
C20H40O11	C12H22O4S3	C9H18O5S	C14H12N2O12S2	C16H30O7S	C18H18O4S
C21H42O11	C13H24O4S3	C10H18O5S	C31H12N2O12S2	C18H34O7S	C19H20O4S
C22H44O11	C14H26O4S3	C11H20O5S	C31H12N2O13S	C20H38O7S	C23H28O4S
C23H46O11	C15H28O4S3	C18H34O5S	C18H30N2O14	C22H42O7S	C27H36O4S
C13H24O11	C16H30O4S3	C19H36O5S	C14H12N2O14	C6H10O7S	C28H38O4S
C14H26O11	C17H32O4S3	C20H38O5S	C17H18N2O14	C7H12O7S	C29H40O4S
C15H28O11	C18H34O4S3	C21H40O5S	C25H14N2O14S	C8H14O7S	C30H42O4S
C16H30O11	C12H20O4S3	C6H10O5S	C37H22N2O14S	C12H20O7S	C31H44O4S
C17H32O11	C16H28O4S3	C7H12O5S	C18H22N2O14S2	C14H24O7S	C32H46O4S
C18H34O11	C18H32O4S3	C9H16O5S	C18H22N2O15S	C18H32O7S	C32H44O4S
C19H36O11	C14H20O4S3	C10H16O5S	C20H16N2O15S	C20H36O7S	C16H12O4S
C20H38O11	C19H20O4S3	C11H18O5S	C23H22N2O15S	C22H40O7S	C18H16O4S
C21H40O11	C20H22O4S3	C12H20O5S	C15H18N2O15S3	C13H20O7S	C19H18O4S
C22H42O11	C22H26O4S3	C13H22O5S	C32H66N2O16	C15H24O7S	C21H22O4S
C23H44O11	C18H16O4S3	C15H26O5S	C20H16N2O16	C17H28O7S	C27H34O4S
C26H50O11	C19H18O4S3	C18H32O5S	C28H18N2O16	C14H22O7S	C28H36O4S
C16H28O11	C32H44O4S3	C19H34O5S	C19H12N2O16S	C23H40O7S	C29H38O4S
C19H34O11	C17H12O4S3	C20H36O5S	C23H12N2O16S	C12H16O7S	C30H40O4S
C20H36O11	C19H16O4S3	C7H10O5S	C26H16N2O16S	C23H30O7S	C26H30O4S
C21H38O11	C31H40O4S3	C8H12O5S	C21H42N2O16S2	C23H20O7S	C27H32O4S
C22H40O11	C33H44O4S3	C9H14O5S	C21H40N2O16S2	C25H24O7S	C29H36O4S
C23H42O11	C49H32O4S3	C12H18O5S	C17H12N2O16S2	C27H28O7S	C30H38O4S
C24H44O11	C10H22O5	C13H20O5S	C17H12N2O17S	C49H30O7S	C31H40O4S
C15H24O11	C11H24O5	C14H22O5S	C15H20N2O17S2	C14H30O7S3	C18H14O4S
C19H32O11	C14H30O5	C15H24O5S	C23H22N2O18S	C15H32O7S3	C20H18O4S
C21H36O11	C9H20O5	C8H10O5S	C25H16N2O19	C10H22O7S3	C21H20O4S
C22H38O11	C14H28O5	C10H12O5S	C17H24N2O20	C11H24O7S3	C28H34O4S
C23H40O11	C15H30O5	C11H14O5S	C20H16N2O23	C17H36O7S3	C31H38O4S
C24H42O11	C16H32O5	C12H16O5S	C17H20N2O6S2	C18H38O7S3	C33H42O4S
C26H46O11	C17H34O5	C13H18O5S	C14H12N2O6S2	C9H20O7S3	C34H44O4S
C17H28O11	C18H36O5	C14H20O5S	C37H20N2O6S2	C12H24O7S3	C21H18O4S
C16H24O11	C19H38O5	C15H22O5S	C11H12N2O6S3	C10H20O7S3	C29H34O4S
C20H32O11	C20H40O5	C9H10O5S	C19H12N2O6S3	C11H22O7S3	C30H36O4S

C21H34O11	C21H42O5	C10H10O5S	C22H18N2O6S3	C9H18O7S3	C31H36O4S
C23H38O11	C22H44O5	C11H12O5S	C37H14N2O7S2	C11H20O7S3	C30H34O4S
C24H40O11	C23H46O5	C12H14O5S	C8H14N2O7S3	C19H36O7S3	C23H18O4S
C25H42O11	C5H10O5	C13H16O5S	C13H14N2O8S2	C15H24O7S3	C10H18O4S2
C27H46O11	C10H20O5	C14H18O5S	C29H20N2O8S2	C17H28O7S3	C7H10O4S2
C26H44O11	C11H22O5	C15H20O5S	C21H46N2O8S3	C25H40O7S3	C9H14O4S2
C14H18O11	C12H24O5	C11H10O5S	C17H12N2O9S	C19H24O7S3	C10H16O4S2
C16H22O11	C6H12O5	C12H12O5S	C26H18N2O9S	C39H30O7S3	C8H12O4S2
C24H38O11	C7H14O5	C13H14O5S	C26H36N2O9S2	C40H12O7S3	C12H18O4S2
C25H40O11	C8H16O5	C14H16O5S	C19H14N2O9S2	C10H22O8	C16H26O4S2
C26H42O11	C9H18O5	C15H18O5S	C23H18N2O9S2	C12H26O8	C8H10O4S2
C27H44O11	C17H32O5	C29H44O5S	C14H10N2O9S3	C14H30O8	C9H12O4S2
C17H24O11	C23H44O5	C13H12O5S	C15H12N2O9S3	C9H20O8	C28H46O4S2
C18H26O11	C24H46O5	C14H14O5S	C34H45N3O12S	C8H18O8	C12H10O4S2
C19H28O11	C25H48O5	C15H16O5S	C29H17N3O12S	C12H24O8	C13H12O4S2
C20H30O11	C10H18O5	C15H14O5S	C11H13N3O13S2	C14H28O8	C14H14O4S2
C28H46O11	C11H20O5	C16H16O5S	C18H23N3O19S	C16H32O8	C24H34O4S2
C24H36O11	C15H28O5	C18H20O5S	C44H25N3O4S3	C18H36O8	C26H38O4S2
C25H38O11	C16H30O5	C27H36O5S	C15H17N3O6S3	C10H20O8	C28H42O4S2
C26H40O11	C19H36O5	C16H14O5S	C14H13N3O7S3	C22H44O8	C14H12O4S2
C15H18O11	C20H38O5	C17H16O5S	C39H79N3O9S2	C24H48O8	C24H32O4S2
C16H20O11	C22H42O5	C19H20O5S	C17H33N3O9S2	C8H16O8	C26H36O4S2
C17H22O11	C6H10O5	C20H22O5S	C11H13NO11S	C9H18O8	C28H40O4S2
C18H24O11	C7H12O5	C30H42O5S	C33H15NO15S	C10H18O8	C16H10O4S2
C19H26O11	C8H14O5	C17H14O5S	C15H17NO5S3	C11H20O8	C17H12O4S2
C20H28O11	C9H16O5	C18H16O5S	C21H33NO6S2	C13H24O8	C36H32O4S2
C21H30O11	C23H42O5	C21H22O5S	C29H19NO6S2	C15H28O8	C38H36O4S2
C23H34O11	C24H44O5	C22H24O5S	C32H25NO6S2	C26H50O8	C35H12O4S2
C16H18O11	C25H46O5	C18H14O5S	C12H25NO6S3	C9H16O8	C44H12O4S2
C17H20O11	C8H12O5	C20H18O5S	C30H57NO6S3	C24H46O8	C10H22O4S3
C18H22O11	C10H16O5	C31H38O5S	C18H21NO7S2	C8H14O8	C11H24O4S3
C19H24O11	C11H18O5	C22H20O5S	C21H27NO7S2	C11H18O8	C14H30O4S3
C20H26O11	C12H20O5	C28H32O5S	C21H33NO8S3	C12H20O8	C17H36O4S3
C21H28O11	C14H24O5	C26H26O5S	C22H27NO8S3	C13H22O8	C9H20O4S3
C22H30O11	C15H26O5	C12H20O5S2	C12H23NO9S	C14H24O8	C12H26O4S3
C23H32O11	C16H28O5	C14H24O5S2	C9H17NO9S	C22H40O8	C20H42O4S3
C24H34O11	C19H34O5	C9H14O5S2	C10H12O10S2	C26H48O8	C11H22O4S3
C25H36O11	C20H36O5	C15H24O5S2	C35H20O12S2	C28H52O8	C17H34O4S3
C17H18O11	C21H38O5	C10H14O5S2	C34H12O12S2	C10H16O8	C18H36O4S3
C18H20O11	C22H40O5	C11H16O5S2	C12H14O12S3	C18H32O8	C22H44O4S3
C19H22O11	C7H10O5	C12H18O5S2	C27H12O12S3	C20H36O8	C12H22O4S3
C20H24O11	C9H14O5	C13H20O5S2	C19H16O13S	C24H44O8	C13H24O4S3
C21H26O11	C13H20O5	C8H10O5S2	C23H20O13S	C8H12O8	C15H28O4S3
C22H28O11	C23H40O5	C9H12O5S2	C20H20O13S3	C9H14O8	C17H32O4S3

C23H30O11	C24H42O5	C11H14O5S2	C32H16O13S3	C11H16O8	C13H22O4S3
C24H32O11	C8H10O5	C13H16O5S2	C15H28O14	C12H18O8	C14H24O4S3
C25H34O11	C9H12O5	C14H18O5S2	C28H14O14S	C18H30O8	C16H28O4S3
C26H36O11	C10H14O5	C14H16O5S2	C29H14O14S	C10H14O8	C14H22O4S3
C27H38O11	C11H16O5	C12H10O5S2	C29H16O14S3	C13H20O8	C20H22O4S3
C17H16O11	C12H18O5	C13H12O5S2	C15H18O15S	C14H22O8	C22H26O4S3
C18H18O11	C17H28O5	C15H16O5S2	C31H26O15S	C16H26O8	C18H16O4S3
C19H20O11	C18H30O5	C15H14O5S2	C14H22O15S2	C20H34O8	C19H18O4S3
C20H22O11	C20H34O5	C15H10O5S2	C13H16O15S2	C24H42O8	C32H44O4S3
C21H24O11	C21H36O5	C16H12O5S2	C23H12O15S2	C12H16O8	C17H12O4S3
C22H26O11	C10H12O5	C17H14O5S2	C22H12O16S	C16H24O8	C19H16O4S3
C23H28O11	C11H14O5	C38H24O5S2	C31H14O16S	C17H26O8	C31H40O4S3
C24H30O11	C12H16O5	C10H22O5S3	C34H20O16S	C10H12O8	C33H44O4S3
C25H32O11	C13H18O5	C11H24O5S3	C29H12O16S2	C13H18O8	C34H46O4S3
C26H34O11	C15H22O5	C12H26O5S3	C16H20O16S3	C14H20O8	C34H44O4S3
C18H16O11	C18H28O5	C13H28O5S3	C23H14O16S3	C15H22O8	C10H22O5
C19H18O11	C21H34O5	C14H30O5S3	C17H14O17S	C22H34O8	C11H24O5
C20H20O11	C24H40O5	C15H32O5S3	C29H22O17S	C23H36O8	C14H30O5
C21H22O11	C9H10O5	C16H34O5S3	C24H24O17S3	C24H38O8	C15H32O5
C22H24O11	C14H20O5	C18H38O5S3	C23H14O18	C25H40O8	C9H20O5
C23H26O11	C16H24O5	C8H18O5S3	C25H16O18	C27H44O8	C14H28O5
C24H28O11	C17H26O5	C9H20O5S3	C37H24O18	C29H48O8	C15H30O5
C25H30O11	C19H30O5	C20H42O5S3	C23H12O18S	C12H14O8	C16H32O5
C26H32O11	C22H36O5	C22H46O5S3	C26H18O18S2	C13H16O8	C17H34O5
C27H34O11	C11H12O5	C10H20O5S3	C30H34O19S2	C14H18O8	C18H36O5
C28H36O11	C12H14O5	C11H22O5S3	C26H12O19S2	C15H20O8	C19H38O5
C20H18O11	C13H16O5	C12H24O5S3	C19H14O20	C20H30O8	C20H40O5
C21H20O11	C14H18O5	C13H26O5S3	C26H18O20	C14H16O8	C21H42O5
C22H22O11	C15H20O5	C14H28O5S3	C17H14O20S	C15H18O8	C22H44O5
C23H24O11	C16H22O5	C16H32O5S3	C20H20O20S	C23H34O8	C23H46O5
C24H26O11	C17H24O5	C17H34O5S3	C27H24O21S3	C24H36O8	C5H10O5
C25H28O11	C18H26O5	C18H36O5S3	C23H12O23S	C27H42O8	C10H20O5
C26H30O11	C19H28O5	C9H18O5S3	C18H38O7S	C29H46O8	C11H22O5
C27H32O11	C20H30O5	C20H40O5S3	C20H42O7S	C13H14O8	C12H24O5
C28H34O11	C21H32O5	C23H46O5S3	C10H20O7S	C16H20O8	C6H12O5
C20H16O11	C24H38O5	C8H16O5S3	C11H22O7S	C17H22O8	C7H14O5
C21H18O11	C10H10O5	C10H18O5S3	C12H24O7S	C18H24O8	C8H16O5
C22H20O11	C22H34O5	C11H20O5S3	C13H26O7S	C14H14O8	C9H18O5
C23H22O11	C11H10O5	C12H22O5S3	C14H28O7S	C15H16O8	C17H32O5
C24H24O11	C12H12O5	C13H24O5S3	C15H30O7S	C16H18O8	C23H44O5
C25H26O11	C13H14O5	C15H28O5S3	C16H32O7S	C17H20O8	C24H46O5
C26H28O11	C14H16O5	C16H30O5S3	C18H36O7S	C18H22O8	C25H48O5
C27H30O11	C15H18O5	C17H32O5S3	C20H40O7S	C17H18O8	C10H18O5
C22H18O11	C16H20O5	C18H34O5S3	C22H44O7S	C14H12O8	C11H20O5

C23H20O11	C17H22O5	C19H36O5S3	C6H12O7S	C16H16O8	C15H28O5
C24H22O11	C18H24O5	C13H22O5S3	C8H16O7S	C18H20O8	C16H30O5
C25H24O11	C19H26O5	C15H26O5S3	C9H18O7S	C19H22O8	C19H36O5
C26H26O11	C20H28O5	C16H28O5S3	C10H18O7S	C17H16O8	C20H38O5
C27H28O11	C21H30O5	C19H34O5S3	C11H20O7S	C19H20O8	C21H40O5
C28H30O11	C22H32O5	C19H20O5S3	C12H22O7S	C19H18O8	C22H42O5
C22H16O11	C24H36O5	C19H18O5S3	C13H24O7S	C19H16O8	C6H10O5
C23H18O11	C25H38O5	C21H22O5S3	C14H26O7S	C18H14O8	C7H12O5
C24H20O11	C23H34O5	C22H24O5S3	C15H28O7S	C20H16O8	C8H14O5
C25H22O11	C13H12O5	C23H26O5S3	C16H30O7S	C22H20O8	C9H16O5
C26H24O11	C14H14O5	C24H28O5S3	C17H32O7S	C24H22O8	C23H42O5
C27H26O11	C15H16O5	C18H16O5S3	C18H34O7S	C31H18O8	C24H44O5
C28H28O11	C16H18O5	C25H30O5S3	C19H36O7S	C34H16O8	C25H46O5
C24H18O11	C17H20O5	C18H14O5S3	C20H38O7S	C39H22O8	C26H48O5
C27H24O11	C18H22O5	C19H16O5S3	C21H40O7S	C40H24O8	C27H50O5
C28H26O11	C19H24O5	C18H12O5S3	C22H42O7S	C41H26O8	C8H12O5
C27H22O11	C21H28O5	C20H16O5S3	C23H44O7S	C20H42O8S	C10H16O5
C23H12O11	C23H32O5	C21H18O5S3	C6H10O7S	C10H20O8S	C11H18O5
C25H50O11S	C25H36O5	C25H22O5S3	C7H12O7S	C7H14O8S	C12H20O5
C24H46O11S	C12H10O5	C11H24O6	C8H14O7S	C8H16O8S	C14H24O5
C26H50O11S	C20H26O5	C12H26O6	C9H16O7S	C9H18O8S	C15H26O5
C25H46O11S	C19H22O5	C13H28O6	C10H16O7S	C15H28O8S	C16H28O5
C26H48O11S	C20H24O5	C14H30O6	C11H18O7S	C12H22O8S	C19H34O5
C13H20O11S	C23H30O5	C15H32O6	C12H20O7S	C18H34O8S	C20H36O5
C21H34O11S	C25H34O5	C8H18O6	C13H22O7S	C20H38O8S	C21H38O5
C18H22O11S	C13H10O5	C9H20O6	C14H24O7S	C22H42O8S	C22H40O5
C22H24O11S	C14H12O5	C11H22O6	C15H26O7S	C15H26O8S	C7H10O5
C37H46O11S	C15H14O5	C13H26O6	C17H30O7S	C16H28O8S	C9H14O5
C37H16O11S	C16H16O5	C15H30O6	C18H32O7S	C17H30O8S	C13H20O5
C12H26O12	C17H18O5	C16H32O6	C20H36O7S	C19H34O8S	C23H40O5
C14H30O12	C18H20O5	C17H34O6	C22H40O7S	C14H24O8S	C24H42O5
C16H34O12	C17H16O5	C18H36O6	C25H46O7S	C18H32O8S	C8H10O5
C17H36O12	C14H10O5	C19H38O6	C7H10O7S	C20H36O8S	C9H12O5
C16H32O12	C15H12O5	C20H40O6	C9H14O7S	C10H14O8S	C10H14O5
C22H44O12	C16H14O5	C5H10O6	C10H14O7S	C16H20O8S	C11H16O5
C23H46O12	C18H18O5	C10H20O6	C11H16O7S	C20H22O8S	C12H18O5
C24H48O12	C20H22O5	C8H16O6	C12H18O7S	C26H24O8S	C17H28O5
C12H22O12	C21H24O5	C9H18O6	C13H20O7S	C18H38O8S2	C18H30O5
C25H42O12	C22H26O5	C17H32O6	C14H22O7S	C18H36O8S2	C20H34O5
C20H28O12	C31H42O5	C19H36O6	C15H24O7S	C16H22O8S2	C10H12O5
C19H24O12	C16H12O5	C20H38O6	C16H26O7S	C15H18O8S2	C11H14O5
C20H26O12	C17H14O5	C21H40O6	C17H28O7S	C21H18O8S2	C12H16O5
C21H28O12	C18H16O5	C23H44O6	C18H30O7S	C35H30O8S2	C13H18O5
C22H30O12	C19H18O5	C6H10O6	C20H34O7S	C11H24O8S3	C15H22O5

C19H22O12	C21H20O5	C7H12O6	C9H12O7S	C12H26O8S3	C18H28O5
C20H24O12	C22H22O5	C9H16O6	C10H12O7S	C14H30O8S3	C21H34O5
C21H26O12	C18H14O5	C10H18O6	C11H14O7S	C23H48O8S3	C24H40O5
C22H28O12	C24H24O5	C11H20O6	C12H16O7S	C19H38O8S3	C14H20O5
C24H32O12	C25H26O5	C12H22O6	C13H18O7S	C21H42O8S3	C16H24O5
C25H34O12	C26H28O5	C14H26O6	C14H20O7S	C14H22O8S3	C17H26O5
C19H20O12	C27H30O5	C15H28O6	C15H22O7S	C22H24O8S3	C19H30O5
C20H22O12	C28H32O5	C16H30O6	C16H24O7S	C27H28O8S3	C22H36O5
C21H24O12	C22H18O5	C18H34O6	C17H26O7S	C27H26O8S3	C9H10O5
C22H26O12	C25H24O5	C22H42O6	C18H28O7S	C41H32O8S3	C11H12O5
C23H28O12	C26H26O5	C32H62O6	C12H14O7S	C10H22O9	C12H14O5
C24H30O12	C27H28O5	C8H14O6	C13H16O7S	C12H26O9	C13H16O5
C25H32O12	C28H30O5	C10H16O6	C14H18O7S	C13H28O9	C14H18O5
C20H20O12	C29H32O5	C11H18O6	C15H20O7S	C14H30O9	C15H20O5
C21H22O12	C30H34O5	C22H40O6	C17H24O7S	C15H32O9	C16H22O5
C22H24O12	C31H36O5	C24H44O6	C14H16O7S	C16H34O9	C17H24O5
C23H26O12	C32H38O5	C8H12O6	C15H18O7S	C9H20O9	C18H26O5
C24H28O12	C33H40O5	C12H20O6	C16H20O7S	C10H20O9	C19H28O5
C25H30O12	C35H44O5	C13H22O6	C18H24O7S	C11H22O9	C20H30O5
C26H32O12	C23H20O5	C17H30O6	C15H16O7S	C12H24O9	C21H32O5
C27H34O12	C24H20O5	C18H32O6	C16H16O7S	C13H26O9	C10H10O5
C21H20O12	C25H22O5	C23H42O6	C18H20O7S	C14H28O9	C22H34O5
C22H22O12	C26H24O5	C7H10O6	C19H22O7S	C15H30O9	C25H40O5
C23H24O12	C28H28O5	C9H14O6	C18H34O7S2	C16H32O9	C11H10O5
C24H26O12	C29H30O5	C10H14O6	C20H38O7S2	C9H18O9	C12H12O5
C25H28O12	C30H32O5	C11H16O6	C18H18O7S2	C22H44O9	C13H14O5
C26H30O12	C31H34O5	C12H18O6	C32H16O7S2	C10H18O9	C14H16O5
C27H32O12	C32H36O5	C16H26O6	C11H16O8S2	C11H20O9	C15H18O5
C28H34O12	C33H38O5	C24H42O6	C16H22O8S2	C12H22O9	C16H20O5
C22H20O12	C34H40O5	C26H46O6	C29H46O8S2	C13H24O9	C17H22O5
C23H22O12	C35H42O5	C13H20O6	C25H24O8S2	C15H28O9	C18H24O5
C24H24O12	C23H18O5	C14H22O6	C27H20O9S3	C16H30O9	C19H26O5
C25H26O12	C27H26O5	C15H24O6	C31H12O9S3	C17H32O9	C20H28O5
C26H28O12	C23H16O5	C17H28O6		C8H14O9	C21H30O5
C27H30O12	C24H18O5	C18H30O6		C12H20O9	C22H32O5
C28H32O12	C26H22O5	C21H36O6		C13H22O9	C23H34O5
C24H22O12	C28H26O5	C22H38O6		C14H24O9	C24H36O5
C25H24O12	C29H28O5	C23H40O6		C16H28O9	C25H38O5
C26H26O12	C30H30O5	C8H10O6		C18H32O9	C27H42O5
C27H28O12	C31H32O5	C9H12O6		C26H48O9	C13H12O5
C28H30O12	C33H36O5	C10H12O6		C10H16O9	C14H14O5
C30H34O12	C35H40O5	C11H14O6		C11H18O9	C15H16O5
C32H38O12	C36H42O5	C13H18O6		C24H44O9	C16H18O5
C25H22O12	C23H14O5	C14H20O6		C12H18O9	C17H20O5

C26H24O12	C24H16O5	C16H24O6		C14H22O9	C18H22O5
C27H26O12	C25H18O5	C17H26O6		C11H16O9	C19H24O5
C29H30O12	C26H20O5	C18H28O6		C16H26O9	C20H26O5
C23H14O12	C27H22O5	C19H30O6		C17H26O9	C21H28O5
C29H26O12	C28H24O5	C22H36O6		C11H14O9	C23H32O5
C20H40O12S	C25H16O5	C24H40O6		C12H16O9	C25H36O5
C21H42O12S	C26H18O5	C12H16O6		C13H18O9	C27H40O5
C22H44O12S	C27H20O5	C15H22O6		C15H22O9	C12H10O5
C22H42O12S	C8H18O5S	C20H32O6		C17H24O9	C15H14O5
C30H52O12S	C10H20O5S	C21H34O6		C23H36O9	C16H16O5
C15H18O12S	C11H22O5S	C23H38O6		C24H38O9	C19H22O5
C28H30O12S	C5H10O5S	C9H10O6		C25H40O9	C21H26O5
C27H28O12S2	C6H12O5S	C11H12O6		C27H44O9	C23H30O5
C12H24O13	C7H14O5S	C13H16O6		C12H14O9	C25H34O5
C13H26O13	C8H16O5S	C14H18O6		C13H16O9	C13H10O5
C14H28O13	C9H18O5S	C15H20O6		C14H18O9	C14H12O5
C15H30O13	C10H18O5S	C16H22O6		C16H22O9	C17H18O5
C17H34O13	C11H20O5S	C17H24O6		C15H18O9	C18H20O5
C21H26O13	C12H22O5S	C18H26O6		C22H32O9	C20H24O5
C21H24O13	C18H34O5S	C19H28O6		C24H36O9	C16H14O5
C22H26O13	C19H36O5S	C20H30O6		C14H16O9	C17H16O5
C23H28O13	C20H38O5S	C21H32O6		C16H20O9	C15H12O5
C24H30O13	C21H40O5S	C22H34O6		C17H22O9	C18H18O5
C26H34O13	C7H12O5S	C10H10O6		C36H60O9	C20H22O5
C22H24O13	C9H16O5S	C12H14O6		C19H24O9	C21H24O5
C23H26O13	C10H16O5S	C23H36O6		C16H18O9	C31H42O5
C24H28O13	C11H18O5S	C45H80O6		C36H58O9	C17H14O5
C25H30O13	C12H20O5S	C12H12O6		C19H22O9	C18H16O5
C26H32O13	C15H26O5S	C13H14O6		C21H24O9	C19H18O5
C17H12O13	C18H32O5S	C14H16O6		C16H14O9	C20H20O5
C22H22O13	C19H34O5S	C15H18O6		C19H14O9	C22H20O5
C24H26O13	C7H10O5S	C16H20O6		C23H20O9	C23H22O5
C25H28O13	C8H12O5S	C17H22O6		C22H16O9	C24H24O5
C26H30O13	C9H14O5S	C18H24O6		C23H16O9	C25H26O5
C27H32O13	C12H18O5S	C19H26O6		C40H26O9	C26H28O5
C24H24O13	C13H20O5S	C20H28O6		C42H30O9	C27H30O5
C25H26O13	C14H22O5S	C21H30O6		C12H24O9S	C19H12O5
C26H28O13	C15H24O5S	C22H32O6		C31H62O9S	C20H14O5
C27H30O13	C8H10O5S	C11H10O6		C8H16O9S	C21H16O5
C28H32O13	C10H12O5S	C14H14O6		C31H60O9S	C22H18O5
C26H26O13	C11H14O5S	C15H16O6		C20H38O9S	C23H20O5
C27H28O13	C12H16O5S	C16H18O6		C10H16O9S	C24H22O5
C28H30O13	C13H18O5S	C17H20O6		C18H32O9S	C25H24O5
C20H34O17	C14H20O5S	C18H22O6		C20H36O9S	C26H26O5

C29H20O17	C15H22O5S	C19H24O6	C23H30O9S	C27H28O5
C18H38O2	C17H26O5S	C20H26O6	C21H26O9S	C28H30O5
C5H10O2	C9H10O5S	C25H36O6	C19H16O9S	C29H32O5
C6H12O2	C10H10O5S	C12H10O6	C28H30O9S	C32H38O5
C7H14O2	C11H12O5S	C13H12O6	C28H28O9S	C31H36O5
C11H22O2	C12H14O5S	C22H30O6	C43H32O9S	C20H12O5
C8H16O2	C13H16O5S	C23H32O6	C37H20O9S	C22H16O5
C9H18O2	C14H18O5S	C15H14O6	C23H42O9S3	C23H18O5
C13H24O2	C15H20O5S	C16H16O6	C26H34O9S3	C24H20O5
C6H10O2	C11H10O5S	C17H18O6	C27H28O9S3	C25H22O5
C7H12O2	C12H12O5S	C18H20O6	C29H28O9S3	C26H24O5
C10H18O2	C13H14O5S	C19H22O6	C42H34O9S3	C27H26O5
C11H20O2	C14H16O5S	C20H24O6	C24H44OS	C28H28O5
C12H22O2	C15H18O5S	C21H26O6	C9H12OS	C29H30O5
C8H14O2	C12H10O5S	C13H10O6	C26H44OS	C30H32O5
C9H16O2	C13H12O5S	C14H12O6	C28H48OS	C31H34O5
C11H18O2	C14H14O5S	C16H14O6	C11H12OS	C32H36O5
C12H20O2	C15H16O5S	C18H18O6	C29H46OS	C33H38O5
C14H24O2	C16H18O5S	C20H22O6	C12H10OS	C34H40O5
C15H26O2	C15H14O5S	C14H10O6	C14H14OS	C35H42O5
C17H30O2	C16H16O5S	C15H12O6	C15H16OS	C36H44O5
C7H10O2	C17H18O5S	C17H16O6	C17H16OS	C37H46O5
C8H12O2	C18H20O5S	C19H20O6	C18H18OS	C22H14O5
C10H16O2	C16H14O5S	C21H24O6	C19H16OS	C23H16O5
C13H22O2	C20H22O5S	C15H10O6	C20H16OS	C24H18O5
C9H14O2	C29H40O5S	C16H12O6	C18H12OS	C25H20O5
C12H18O2	C24H26O5S	C17H14O6	C20H14OS	C26H22O5
C13H20O2	C20H18O5S	C18H16O6	C21H16OS	C27H24O5
C14H22O2	C28H32O5S	C19H18O6	C22H18OS	C28H26O5
C10H14O2	C30H34O5S	C20H20O6	C33H40OS	C29H28O5
C11H16O2	C23H20O5S	C21H22O6	C22H16OS	C31H32O5
C8H10O2	C48H28O5S	C16H10O6	C29H26OS	C32H34O5
C9H12O2	C14H24O5S2	C17H12O6	C30H28OS	C33H36O5
C12H16O2	C10H16O5S2	C18H14O6	C32H20OS	C34H38O5
C13H18O2	C15H24O5S2	C19H16O6	C33H22OS	C35H40O5
C18H28O2	C10H14O5S2	C20H18O6	C43H24OS	C36H42O5
C9H10O2	C11H16O5S2	C21H20O6	C10H18OS2	C37H44O5
C10H12O2	C12H18O5S2	C20H16O6	C11H20OS2	C30H30O5
C11H14O2	C8H10O5S2	C21H18O6	C9H16OS2	C23H14O5
C11H12O2	C9H12O5S2	C22H20O6	C16H16OS2	C24H16O5
C12H14O2	C12H14O5S2	C24H24O6	C18H20OS2	C25H18O5
C13H16O2	C13H16O5S2	C25H26O6	C20H24OS2	C26H20O5
C15H20O2	C26H42O5S2	C26H28O6	C26H34OS2	C27H22O5
C18H26O2	C14H16O5S2	C27H30O6	C20H18OS2	C28H24O5

C10H10O2	C13H12O5S2	C19H14O6		C22H22OS2	C29H26O5
C14H18O2	C15H16O5S2	C18H10O6		C24H26OS2	C25H16O5
C12H12O2	C15H14O5S2	C21H16O6		C26H30OS2	C27H20O5
C28H44O2	C16H16O5S2	C23H20O6		C18H14OS2	C22H10O5
C11H10O2	C20H22O5S2	C24H22O6		C27H26OS2	C23H12O5
C23H32O2	C15H10O5S2	C25H24O6		C36H30OS2	C28H22O5
C13H10O2	C16H12O5S2	C26H26O6		C39H26OS2	C25H14O5
C20H22O2	C18H16O5S2	C27H28O6		C35H18OS2	C8H18O5S
C16H10O2	C22H18O5S2	C28H30O6		C13H24OS3	C10H20O5S
C17H12O2	C36H14O5S2	C20H12O6		C15H28OS3	C5H10O5S
C18H14O2	C10H22O5S3	C22H16O6		C13H20OS3	C6H12O5S
C19H16O2	C11H24O5S3	C23H18O6		C19H18OS3	C7H14O5S
C20H18O2	C12H26O5S3	C24H20O6		C20H12OS3	C8H16O5S
C18H12O2	C13H28O5S3	C26H24O6		C30H28OS3	C9H18O5S
C19H14O2	C14H30O5S3	C27H26O6		C39H44OS3	C20H38O5S
C20H16O2	C15H32O5S3	C28H28O6		C35H32OS3	C10H18O5S
C25H26O2	C16H34O5S3	C29H30O6		C33H18OS3	C11H20O5S
C22H18O2	C18H38O5S3	C31H34O6		C35H22OS3	C12H22O5S
C27H28O2	C19H40O5S3	C32H36O6		C37H26OS3	C13H24O5S
C29H32O2	C20H42O5S3	C33H38O6		C41H26OS3	C18H34O5S
C22H16O2	C22H46O5S3	C34H40O6		C15H26N2O11	C19H36O5S
C28H28O2	C24H50O5S3	C36H44O6		C16H24N2O11	C21H40O5S
C30H32O2	C25H52O5S3	C25H22O6		C18H28N2O11	C6H10O5S
C22H14O2	C23H48O5S3	C30H32O6		C37H36N2O11	C7H12O5S
C29H28O2	C8H18O5S3	C21H12O6		C20H24N2O12S	C8H14O5S
C37H44O2	C9H20O5S3	C22H14O6		C17H36N2O12S2	C9H16O5S
C27H22O2	C10H20O5S3	C23H16O6		C18H36N2O13	C21H38O5S
C38H10O2	C12H24O5S3	C24H18O6		C24H34N2O13	C10H16O5S
C13H28O2S	C13H26O5S3	C25H20O6		C25H36N2O13	C11H18O5S
C13H26O2S	C14H28O5S3	C26H22O6		C23H16N2O13	C12H20O5S
C13H22O2S	C15H30O5S3	C27H24O6		C24H18N2O13	C13H22O5S
C14H24O2S	C16H32O5S3	C28H26O6		C27H24N2O13	C15H26O5S
C16H28O2S	C17H34O5S3	C30H30O6		C26H20N2O13	C18H32O5S
C7H10O2S	C18H36O5S3	C31H32O6		C30H50N2O13S	C19H34O5S
C9H14O2S	C20H40O5S3	C32H34O6		C16H30N2O14	C20H36O5S
C15H24O2S	C21H42O5S3	C22H12O6		C20H38N2O14	C23H42O5S
C17H28O2S	C23H46O5S3	C23H14O6		C28H22N2O14	C7H10O5S
C8H10O2S	C24H48O5S3	C25H18O6		C40H42N2O14	C8H12O5S
C9H12O2S	C25H50O5S3	C26H20O6		C13H20N2O15	C9H14O5S
C24H40O2S	C26H52O5S3	C27H22O6		C19H32N2O15	C12H18O5S
C17H22O2S	C8H16O5S3	C28H24O6		C20H34N2O15	C13H20O5S
C27H40O2S	C12H22O5S3	C29H26O6		C21H36N2O15	C14H22O5S
C24H32O2S	C13H24O5S3	C24H14O6		C22H38N2O15	C15H24O5S
C13H10O2S	C16H30O5S3	C25H16O6		C23H40N2O15	C18H30O5S

C14H12O2S	C17H32O5S3	C26H18O6	C21H34N2O15	C19H32O5S
C15H14O2S	C19H36O5S3	C27H20O6	C22H36N2O15	C8H10O5S
C16H16O2S	C22H42O5S3	C28H22O6	C25H36N2O15	C10H12O5S
C27H38O2S	C13H22O5S3	C30H26O6	C26H38N2O15	C11H14O5S
C14H10O2S	C14H24O5S3	C23H12O6	C26H20N2O15	C12H16O5S
C15H12O2S	C15H26O5S3	C25H14O6	C20H34N2O16	C13H18O5S
C27H36O2S	C16H28O5S3	C26H16O6	C21H36N2O16	C14H20O5S
C15H10O2S	C19H34O5S3	C27H18O6	C28H18N2O16	C15H22O5S
C16H12O2S	C13H20O5S3	C28H20O6	C24H12N2O16S	C16H24O5S
C16H10O2S	C19H18O5S3	C25H12O6	C21H34N2O17	C9H10O5S
C26H30O2S	C21H22O5S3	C26H14O6	C22H36N2O17	C10H10O5S
C27H32O2S	C22H24O5S3	C35H24O6	C23H38N2O17	C11H12O5S
C33H42O2S	C23H26O5S3	C33H18O6	C24H40N2O17	C12H14O5S
C19H14O2S	C24H28O5S3	C33H16O6	C21H28N2O17S2	C13H16O5S
C27H26O2S	C20H20O5S3	C10H22O6S	C21H14N2O18	C14H18O5S
C29H18O2S	C25H30O5S3	C8H18O6S	C22H40N2O19	C15H20O5S
C38H36O2S	C18H14O5S3	C10H20O6S	C21H16N2O19	C16H22O5S
C25H44O2S2	C19H16O5S3	C11H22O6S	C27H26N2O24	C11H10O5S
C15H22O2S2	C20H16O5S3	C13H26O6S	C14H24N2O4S3	C12H12O5S
C16H24O2S2	C21H18O5S3	C15H30O6S	C13H24N2O5S3	C13H14O5S
C19H30O2S2	C34H44O5S3	C16H32O6S	C26H12N2O5S3	C14H16O5S
C11H10O2S2	C25H22O5S3	C18H36O6S	C13H20N2O6S3	C29H44O5S
C12H12O2S2	C11H24O6	C20H40O6S	C8H14N2O7S3	C14H14O5S
C15H18O2S2	C12H26O6	C22H44O6S	C20H34N2O9S3	C15H16O5S
C19H26O2S2	C13H28O6	C6H12O6S	C15H17N3O10S	C16H18O5S
C21H28O2S2	C14H30O6	C7H14O6S	C14H19N3O10S3	C28H40O5S
C12H10O2S2	C15H32O6	C8H16O6S	C28H17N3O12S3	C29H42O5S
C13H12O2S2	C9H20O6	C9H18O6S	C29H41N3O16S	C16H16O5S
C28H38O2S2	C11H22O6	C10H18O6S	C18H23N3O19S	C17H18O5S
C29H40O2S2	C13H26O6	C11H20O6S	C29H29N3O22	C18H20O5S
C14H10O2S2	C15H30O6	C13H24O6S	C21H13N3O22S	C23H28O5S
C38H46O2S2	C16H32O6	C15H28O6S	C26H33N3O23	C36H54O5S
C24H16O2S2	C17H34O6	C16H30O6S	C23H41N3O26	C15H12O5S
C25H18O2S2	C18H36O6	C18H34O6S	C18H19N3O3S3	C16H14O5S
C36H34O2S2	C19H38O6	C19H36O6S	C12H17N3O6S3	C17H16O5S
C34H22O2S2	C20H40O6	C20H38O6S	C24H33N3O7S2	C18H18O5S
C38H30O2S2	C22H44O6	C21H40O6S	C9H17N3O7S3	C30H42O5S
C33H18O2S2	C10H20O6	C22H42O6S	C13H15N3O7S3	C31H44O5S
C36H24O2S2	C8H16O6	C6H10O6S	C25H19N3O8S	C32H46O5S
C33H14O2S2	C17H32O6	C7H12O6S	C23H19N3O8S2	C42H66O5S
C15H32O2S3	C19H36O6	C8H14O6S	C31H27N3O8S2	C26H32O5S
C17H36O2S3	C20H38O6	C9H16O6S	C9H17N3O9S3	C16H12O5S
C11H22O2S3	C21H40O6	C14H24O6S	C11H17NO10S	C18H16O5S
C12H24O2S3	C23H44O6	C10H16O6S	C22H27NO10S	C20H20O5S

C13H26O2S3	C25H48O6	C11H18O6S		C23H29NO10S	C21H22O5S
C14H28O2S3	C26H50O6	C12H20O6S		C26H35NO10S	C22H24O5S
C15H30O2S3	C6H10O6	C13H22O6S		C31H19NO10S	C30H40O5S
C16H32O2S3	C7H12O6	C15H26O6S		C27H21NO10S2	C32H44O5S
C17H34O2S3	C9H16O6	C18H32O6S		C38H69NO11S2	C26H30O5S
C20H40O2S3	C10H18O6	C20H36O6S		C12H23NO12S	C31H40O5S
C22H42O2S3	C11H20O6	C8H12O6S		C19H25NO12S	C18H14O5S
C18H24O2S3	C12H22O6	C9H14O6S		C26H33NO12S	C26H28O5S
C13H14O2S3	C14H26O6	C11H16O6S		C40H61NO12S	C22H20O5S
C16H12O2S3	C15H28O6	C12H18O6S		C25H17NO12S	C28H32O5S
C19H18O2S3	C16H30O6	C13H20O6S		C23H33NO12S2	C32H38O5S
C22H18O2S3	C18H34O6	C14H22O6S		C38H33NO12S2	C23H20O5S
C25H14O2S3	C22H42O6	C15H24O6S		C29H19NO13S3	C29H30O5S
C42H48O2S3	C24H46O6	C16H26O6S		C25H13NO15S	C14H24O5S2
C34H18O2S3	C8H14O6	C20H34O6S		C27H15NO15S3	C11H18O5S2
C37H24O2S3	C10H16O6	C8H10O6S		C32H39NO16S2	C15H24O5S2
C11H24O3	C11H18O6	C9H12O6S		C26H41NO20	C10H14O5S2
C13H28O3	C22H40O6	C10H12O6S		C20H21NO6S	C11H16O5S2
C13H26O3	C23H42O6	C11H14O6S		C33H41NO6S	C12H18O5S2
C14H28O3	C24H44O6	C12H16O6S		C45H37NO6S	C9H12O5S2
C15H30O3	C25H46O6	C13H18O6S		C49H27NO6S	C13H16O5S2
C17H34O3	C26H48O6	C14H20O6S		C11H23NO9S	C12H10O5S2
C19H38O3	C8H12O6	C15H22O6S		C9H17NO9S	C13H12O5S2
C20H40O3	C12H20O6	C9H10O6S		C28H31NO9S	C15H16O5S2
C10H20O3	C13H22O6	C10H10O6S		C10H12O10S2	C15H14O5S2
C11H22O3	C17H30O6	C11H12O6S		C18H22O10S2	C29H42O5S2
C12H24O3	C18H32O6	C12H14O6S		C21H20O10S2	C16H14O5S2
C21H42O3	C7H10O6	C13H16O6S		C33H36O10S2	C15H10O5S2
C23H46O3	C9H14O6	C14H18O6S		C13H22O12S	C16H12O5S2
C24H48O3	C10H14O6	C15H20O6S		C24H32O12S	C10H22O5S3
C5H10O3	C11H16O6	C16H22O6S		C29H30O12S	C11H24O5S3
C6H12O3	C12H18O6	C12H12O6S		C35H42O12S	C12H26O5S3
C7H14O3	C16H26O6	C13H14O6S		C36H40O12S2	C13H28O5S3
C8H16O3	C24H42O6	C14H16O6S		C32H26O12S2	C14H30O5S3
C13H24O3	C25H44O6	C15H18O6S		C25H46O12S3	C20H42O5S3
C15H28O3	C26H46O6	C16H20O6S		C17H36O14S3	C8H18O5S3
C22H42O3	C13H20O6	C17H22O6S		C34H40O15S	C9H20O5S3
C6H10O3	C14H22O6	C12H10O6S		C27H30O15S2	C22H46O5S3
C14H26O3	C15H24O6	C13H12O6S		C28H28O16S2	C23H48O5S3
C7H12O3	C17H28O6	C15H16O6S		C32H14O17S3	C24H50O5S3
C9H16O3	C18H30O6	C16H18O6S		C17H16O18S	C10H20O5S3
C15H26O3	C21H36O6	C17H18O6S		C35H42O18S	C13H26O5S3
C17H30O3	C22H38O6	C16H14O6S		C33H48O20S	C16H32O5S3
C21H38O3	C23H40O6	C17H16O6S		C17H32O7S2	C17H34O5S3

C22H40O3	C8H10O6	C18H18O6S	C20H34O7S2	C20H40O5S3
C11H18O3	C9H12O6	C31H42O6S	C28H32O7S2	C21H42O5S3
C16H28O3	C10H12O6	C18H16O6S	C29H30O9S2	C26H52O5S3
C20H36O3	C11H14O6	C29H34O6S	C37H30O9S2	C14H28O5S3
C7H10O3	C13H18O6	C18H12O6S		C22H44O5S3
C8H12O3	C14H20O6	C19H14O6S		C23H46O5S3
C9H14O3	C16H24O6	C29H32O6S		C35H70O5S3
C11H16O3	C17H26O6	C30H34O6S		C8H16O5S3
C12H18O3	C18H28O6	C12H22O6S2		C12H22O5S3
C22H38O3	C19H30O6	C10H16O6S2		C13H24O5S3
C8H10O3	C22H36O6	C13H20O6S2		C16H30O5S3
C10H14O3	C23H38O6	C12H18O6S2		C17H32O5S3
C13H20O3	C24H40O6	C19H18O6S2		C27H52O5S3
C15H24O3	C26H44O6	C18H12O6S2		C13H22O5S3
C20H34O3	C12H16O6	C23H12O6S2		C14H24O5S3
C9H12O3	C15H22O6	C10H22O6S3		C15H26O5S3
C12H16O3	C20H32O6	C12H26O6S3		C13H20O5S3
C13H18O3	C21H34O6	C13H28O6S3		C19H18O5S3
C14H20O3	C9H10O6	C14H30O6S3		C21H22O5S3
C16H24O3	C11H12O6	C15H32O6S3		C23H26O5S3
C17H26O3	C13H16O6	C16H34O6S3		C18H16O5S3
C18H28O3	C14H18O6	C17H36O6S3		C22H24O5S3
C22H36O3	C15H20O6	C19H40O6S3		C18H14O5S3
C10H12O3	C16H22O6	C21H44O6S3		C19H16O5S3
C11H14O3	C17H24O6	C9H20O6S3		C31H40O5S3
C15H22O3	C18H26O6	C11H22O6S3		C20H16O5S3
C20H32O3	C19H28O6	C12H24O6S3		C21H18O5S3
C9H10O3	C20H30O6	C13H26O6S3		C30H36O5S3
C10H10O3	C21H32O6	C14H28O6S3		C32H40O5S3
C11H12O3	C22H34O6	C15H30O6S3		C33H40O5S3
C12H14O3	C23H36O6	C17H34O6S3		C25H22O5S3
C13H16O3	C24H38O6	C19H38O6S3		C28H26O5S3
C14H18O3	C25H40O6	C10H20O6S3		C11H24O6
C15H20O3	C10H10O6	C9H18O6S3		C12H26O6
C16H22O3	C12H14O6	C12H22O6S3		C13H28O6
C18H26O3	C12H12O6	C13H24O6S3		C14H30O6
C20H30O3	C13H14O6	C15H28O6S3		C15H32O6
C23H36O3	C14H16O6	C16H30O6S3		C9H20O6
C15H18O3	C15H18O6	C17H32O6S3		C11H22O6
C16H20O3	C16H20O6	C19H36O6S3		C13H26O6
C18H24O3	C17H22O6	C10H18O6S3		C15H30O6
C21H30O3	C18H24O6	C20H38O6S3		C16H32O6
C23H34O3	C19H26O6	C15H26O6S3		C17H34O6
C25H38O3	C20H28O6	C14H24O6S3		C18H36O6

C12H12O3	C21H30O6	C23H30O6S3		C19H38O6
C13H14O3	C22H32O6	C24H32O6S3		C20H40O6
C14H16O3	C23H34O6	C23H26O6S3		C22H44O6
C12H10O3	C24H36O6	C25H30O6S3		C10H20O6
C13H12O3	C11H10O6	C22H22O6S3		C8H16O6
C23H32O3	C13H12O6	C23H24O6S3		C9H18O6
C25H36O3	C14H14O6	C24H26O6S3		C17H32O6
C19H22O3	C15H16O6	C18H14O6S3		C19H36O6
C13H10O3	C16H18O6	C20H18O6S3		C20H38O6
C14H12O3	C17H20O6	C21H20O6S3		C21H40O6
C16H16O3	C18H22O6	C20H16O6S3		C23H44O6
C23H28O3	C19H24O6	C21H18O6S3		C25H48O6
C14H10O3	C20H26O6	C34H44O6S3		C26H50O6
C15H12O3	C25H36O6	C12H26O7		C6H10O6
C17H16O3	C12H10O6	C13H28O7		C7H12O6
C20H20O3	C22H30O6	C14H30O7		C9H16O6
C27H34O3	C15H14O6	C15H32O7		C10H18O6
C26H30O3	C16H16O6	C10H20O7		C11H20O6
C30H38O3	C17H18O6	C11H22O7		C12H22O6
C31H40O3	C18H20O6	C12H24O7		C14H26O6
C32H42O3	C19H22O6	C13H26O7		C15H28O6
C33H44O3	C20H24O6	C14H28O7		C16H30O6
C17H10O3	C21H26O6	C15H30O7		C18H34O6
C18H12O3	C13H10O6	C16H32O7		C22H42O6
C19H14O3	C14H12O6	C17H34O7		C24H46O6
C20H16O3	C18H18O6	C18H36O7		C8H14O6
C21H18O3	C14H10O6	C19H38O7		C10H16O6
C23H22O3	C15H12O6	C20H40O7		C11H18O6
C25H26O3	C16H14O6	C8H16O7		C22H40O6
C26H28O3	C17H16O6	C10H18O7		C23H42O6
C27H30O3	C19H20O6	C11H20O7		C24H44O6
C30H36O3	C20H22O6	C12H22O7		C25H46O6
C31H38O3	C21H24O6	C13H24O7		C26H48O6
C32H40O3	C16H12O6	C15H28O7		C8H12O6
C33H42O3	C17H14O6	C16H30O7		C12H20O6
C34H44O3	C18H16O6	C17H32O7		C13H22O6
C35H46O3	C19H18O6	C20H38O7		C17H30O6
C21H16O3	C20H20O6	C21H40O7		C18H32O6
C25H24O3	C16H10O6	C22H42O7		C7H10O6
C26H26O3	C17H12O6	C23H44O7		C9H14O6
C27H28O3	C19H16O6	C24H46O7		C10H14O6
C28H30O3	C20H18O6	C9H16O7		C11H16O6
C29H32O3	C22H22O6	C14H26O7		C12H18O6
C30H34O3	C20H16O6	C18H34O7		C16H26O6

C31H36O3	C22H20O6	C19H36O7	C24H42O6
C32H38O3	C24H24O6	C6H10O7	C25H44O6
C33H40O3	C25H26O6	C7H12O7	C26H46O6
C34H42O3	C26H28O6	C8H14O7	C13H20O6
C35H44O3	C27H30O6	C10H16O7	C14H22O6
C23H18O3	C23H20O6	C11H18O7	C15H24O6
C24H20O3	C25H24O6	C13H22O7	C17H28O6
C26H24O3	C26H26O6	C14H24O7	C18H30O6
C27H26O3	C27H28O6	C15H26O7	C21H36O6
C28H28O3	C28H30O6	C22H40O7	C22H38O6
C29H30O3	C29H32O6	C23H42O7	C23H40O6
C30H32O3	C20H12O6	C24H44O7	C8H10O6
C31H34O3	C21H14O6	C25H46O7	C9H12O6
C33H38O3	C22H16O6	C26H48O7	C10H12O6
C32H36O3	C23H18O6	C9H14O7	C11H14O6
C24H18O3	C24H20O6	C12H20O7	C13H18O6
C26H22O3	C26H24O6	C16H28O7	C14H20O6
C28H26O3	C27H26O6	C17H30O7	C16H24O6
C29H28O3	C28H28O6	C18H32O7	C17H26O6
C31H32O3	C29H30O6	C19H34O7	C18H28O6
C24H16O3	C36H44O6	C20H36O7	C19H30O6
C29H26O3	C25H22O6	C21H38O7	C22H36O6
C40H30O3	C31H34O6	C7H10O7	C23H38O6
C12H26O3S	C22H14O6	C8H12O7	C24H40O6
C8H18O3S	C23H16O6	C10H14O7	C25H42O6
C5H10O3S	C24H18O6	C11H16O7	C27H46O6
C6H12O3S	C25H20O6	C12H18O7	C28H48O6
C7H14O3S	C26H22O6	C14H22O7	C29H50O6
C8H16O3S	C28H26O6	C15H24O7	C12H16O6
C18H34O3S	C30H30O6	C17H28O7	C15H22O6
C12H20O3S	C31H32O6	C18H30O7	C20H32O6
C9H14O3S	C23H14O6	C23H40O7	C21H34O6
C7H10O3S	C24H16O6	C24H42O7	C26H44O6
C10H14O3S	C25H18O6	C13H20O7	C9H10O6
C9H12O3S	C26H20O6	C16H26O7	C11H12O6
C8H10O3S	C27H22O6	C20H34O7	C13H16O6
C10H12O3S	C22H12O6	C21H36O7	C14H18O6
C11H14O3S	C25H16O6	C22H38O7	C15H20O6
C12H16O3S	C26H18O6	C8H10O7	C16H22O6
C18H28O3S	C27H20O6	C9H12O7	C17H24O6
C9H10O3S	C28H22O6	C12H16O7	C18H26O6
C12H12O3S	C30H26O6	C13H18O7	C19H28O6
C14H14O3S	C23H12O6	C14H20O7	C20H30O6
C17H20O3S	C26H16O6	C15H22O7	C21H32O6

C19H24O3S	C28H20O6	C16H24O7	C22H34O6
C30H44O3S	C23H10O6	C17H26O7	C23H36O6
C15H14O3S	C26H14O6	C18H28O7	C24H38O6
C16H16O3S	C8H18O6S	C19H30O7	C25H40O6
C17H18O3S	C10H20O6S	C20H32O7	C27H44O6
C18H20O3S	C11H22O6S	C26H44O7	C10H10O6
C20H24O3S	C16H32O6S	C10H12O7	C12H14O6
C21H26O3S	C17H34O6S	C11H14O7	C12H12O6
C23H30O3S	C18H36O6S	C21H34O7	C13H14O6
C27H38O3S	C20H40O6S	C22H36O7	C14H16O6
C29H42O3S	C22H44O6S	C23H38O7	C15H18O6
C15H12O3S	C6H12O6S	C24H40O7	C16H20O6
C16H14O3S	C7H14O6S	C9H10O7	C17H22O6
C17H16O3S	C8H16O6S	C13H16O7	C18H24O6
C18H18O3S	C10H18O6S	C15H20O7	C19H26O6
C21H24O3S	C11H20O6S	C16H22O7	C20H28O6
C27H36O3S	C15H28O6S	C17H24O7	C21H30O6
C29H40O3S	C18H34O6S	C18H26O7	C22H32O6
C33H46O3S	C19H36O6S	C19H28O7	C23H34O6
C16H12O3S	C20H38O6S	C20H30O7	C11H10O6
C17H14O3S	C21H40O6S	C21H32O7	C24H36O6
C18H16O3S	C22H42O6S	C22H34O7	C25H38O6
C19H18O3S	C6H10O6S	C24H38O7	C26H40O6
C33H44O3S	C7H12O6S	C10H10O7	C14H14O6
C37H52O3S	C8H14O6S	C11H12O7	C15H16O6
C17H12O3S	C9H16O6S	C12H14O7	C16H18O6
C27H32O3S	C14H24O6S	C14H18O7	C17H20O6
C17H10O3S	C10H16O6S	C23H36O7	C18H22O6
C27H30O3S	C11H18O6S	C13H14O7	C19H24O6
C35H44O3S	C12H20O6S	C14H16O7	C20H26O6
C40H54O3S	C13H22O6S	C15H18O7	C25H36O6
C20H14O3S	C15H26O6S	C16H20O7	C27H40O6
C27H14O3S	C18H32O6S	C17H22O7	C12H10O6
C39H36O3S	C20H36O6S	C18H24O7	C13H12O6
C9H14O3S2	C22H40O6S	C19H26O7	C22H30O6
C24H42O3S2	C8H12O6S	C20H28O7	C16H16O6
C37H66O3S2	C9H14O6S	C21H30O7	C17H18O6
C20H28O3S2	C11H16O6S	C22H32O7	C18H20O6
C12H10O3S2	C12H18O6S	C11H10O7	C19H22O6
C13H12O3S2	C14H22O6S	C12H12O7	C20H24O6
C25H36O3S2	C15H24O6S	C24H36O7	C21H26O6
C27H40O3S2	C8H10O6S	C15H16O7	C13H10O6
C13H10O3S2	C9H12O6S	C16H18O7	C14H12O6
C14H12O3S2	C10H12O6S	C17H20O7	C15H14O6

C14H10O3S2	C11H14O6S	C18H22O7	C22H28O6
C15H12O3S2	C12H16O6S	C19H24O7	C17H16O6
C24H14O3S2	C13H18O6S	C20H26O7	C18H18O6
C26H18O3S2	C14H20O6S	C21H28O7	C19H20O6
C29H24O3S2	C15H22O6S	C22H30O7	C20H22O6
C35H32O3S2	C16H24O6S	C12H10O7	C14H10O6
C37H36O3S2	C9H10O6S	C13H12O7	C15H12O6
C37H34O3S2	C10H10O6S	C14H14O7	C16H14O6
C36H24O3S2	C11H12O6S	C23H32O7	C21H24O6
C37H26O3S2	C12H14O6S	C24H34O7	C22H26O6
C10H22O3S3	C13H16O6S	C25H36O7	C15H10O6
C11H24O3S3	C14H18O6S	C15H14O7	C16H12O6
C13H28O3S3	C15H20O6S	C16H16O7	C17H14O6
C14H30O3S3	C16H22O6S	C17H18O7	C18H16O6
C16H34O3S3	C17H24O6S	C18H20O7	C19H18O6
C18H38O3S3	C18H26O6S	C19H22O7	C20H20O6
C8H18O3S3	C12H12O6S	C20H24O7	C22H24O6
C12H26O3S3	C13H14O6S	C21H26O7	C17H12O6
C10H20O3S3	C14H16O6S	C22H28O7	C19H16O6
C11H22O3S3	C15H18O6S	C13H10O7	C20H18O6
C12H24O3S3	C16H20O6S	C14H12O7	C21H20O6
C14H28O3S3	C17H22O6S	C23H30O7	C20H16O6
C16H32O3S3	C18H24O6S	C18H18O7	C21H18O6
C17H34O3S3	C14H14O6S	C19H20O7	C24H24O6
C18H36O3S3	C15H16O6S	C20H22O7	C25H26O6
C19H38O3S3	C16H18O6S	C21H24O7	C26H28O6
C8H16O3S3	C17H20O6S	C14H10O7	C18H10O6
C9H18O3S3	C18H22O6S	C15H12O7	C20H14O6
C11H20O3S3	C15H14O6S	C16H14O7	C21H16O6
C12H22O3S3	C16H16O6S	C17H16O7	C22H18O6
C13H24O3S3	C18H20O6S	C22H26O7	C23H20O6
C14H26O3S3	C16H14O6S	C15H10O7	C24H22O6
C15H28O3S3	C17H16O6S	C16H12O7	C25H24O6
C16H30O3S3	C18H12O6S	C17H14O7	C26H26O6
C17H32O3S3	C29H32O6S	C18H16O7	C27H28O6
C22H42O3S3	C30H34O6S	C19H18O7	C20H12O6
C23H44O3S3	C39H14O6S	C20H20O7	C21H14O6
C25H48O3S3	C13H20O6S2	C21H22O7	C22H16O6
C9H16O3S3	C10H14O6S2	C22H24O7	C23H18O6
C13H22O3S3	C12H18O6S2	C23H26O7	C24H20O6
C13H20O3S3	C11H14O6S2	C20H18O7	C26H24O6
C21H26O3S3	C12H16O6S2	C21H20O7	C27H26O6
C16H14O3S3	C19H18O6S2	C17H12O7	C29H30O6
C19H20O3S3	C22H24O6S2	C18H14O7	C31H34O6

C17H12O3S3	C18H12O6S2	C19H16O7	C33H38O6
C22H22O3S3	C20H16O6S2	C22H22O7	C34H40O6
C31H40O3S3	C23H14O6S2	C23H24O7	C36H44O6
C21H14O3S3	C23H12O6S2	C21H18O7	C48H68O6
C22H10O3S3	C33H14O6S2	C23H22O7	C28H28O6
C23H12O3S3	C41H18O6S2	C26H28O7	C30H32O6
C40H32O3S3	C10H22O6S3	C19H14O7	C32H36O6
C11H24O4	C11H24O6S3	C20H16O7	C23H16O6
C12H26O4	C12H26O6S3	C22H20O7	C24H18O6
C14H30O4	C13H28O6S3	C20H14O7	C25H20O6
C15H30O4	C14H30O6S3	C22H18O7	C26H22O6
C16H32O4	C15H32O6S3	C23H20O7	C28H26O6
C22H44O4	C16H34O6S3	C24H22O7	C30H30O6
C10H20O4	C17H36O6S3	C25H24O7	C31H32O6
C12H24O4	C19H40O6S3	C26H26O7	C32H34O6
C13H26O4	C20H42O6S3	C27H28O7	C34H38O6
C14H28O4	C21H44O6S3	C21H16O7	C23H14O6
C17H34O4	C23H48O6S3	C20H12O7	C24H16O6
C19H38O4	C9H20O6S3	C21H14O7	C25H18O6
C20H40O4	C11H22O6S3	C22H16O7	C26H20O6
C21H42O4	C13H26O6S3	C23H18O7	C27H22O6
C24H48O4	C14H28O6S3	C24H20O7	C29H26O6
C5H10O4	C15H30O6S3	C25H22O7	C31H30O6
C6H12O4	C17H34O6S3	C26H24O7	C22H10O6
C7H14O4	C18H36O6S3	C27H26O7	C23H12O6
C8H16O4	C19H38O6S3	C28H28O7	C24H14O6
C9H18O4	C22H44O6S3	C29H30O7	C25H16O6
C24H46O4	C24H48O6S3	C32H36O7	C24H12O6
C15H28O4	C26H52O6S3	C21H12O7	C36H36O6
C17H32O4	C10H20O6S3	C22H14O7	C25H12O6
C19H36O4	C23H46O6S3	C23H16O7	C45H40O6
C20H38O4	C9H18O6S3	C24H18O7	C8H18O6S
C21H40O4	C12H22O6S3	C25H20O7	C9H20O6S
C22H42O4	C14H26O6S3	C26H22O7	C10H20O6S
C23H44O4	C15H28O6S3	C27H24O7	C11H22O6S
C25H48O4	C16H30O6S3	C29H28O7	C12H24O6S
C6H10O4	C17H32O6S3	C32H34O7	C13H26O6S
C7H12O4	C19H36O6S3	C24H16O7	C15H30O6S
C20H36O4	C20H38O6S3	C25H18O7	C16H32O6S
C21H38O4	C22H42O6S3	C26H20O7	C17H34O6S
C22H40O4	C25H48O6S3	C27H22O7	C18H36O6S
C23H42O4	C10H18O6S3	C28H24O7	C19H38O6S
C24H44O4	C13H24O6S3	C29H26O7	C20H40O6S
C11H18O4	C16H28O6S3	C22H12O7	C22H44O6S

C17H30O4	C18H32O6S3	C23H14O7	C6H12O6S
C19H34O4	C21H38O6S3	C24H14O7	C7H14O6S
C7H10O4	C13H22O6S3	C25H16O7	C8H16O6S
C8H12O4	C14H24O6S3	C26H18O7	C9H18O6S
C9H14O4	C20H34O6S3	C27H20O7	C10H18O6S
C11H16O4	C13H20O6S3	C28H22O7	C11H20O6S
C22H38O4	C23H30O6S3	C29H24O7	C12H22O6S
C23H40O4	C24H32O6S3	C28H20O7	C13H24O6S
C24H42O4	C17H16O6S3	C27H18O7	C14H26O6S
C8H10O4	C24H28O6S3	C27H14O7	C15H28O6S
C9H12O4	C22H22O6S3	C35H22O7	C16H30O6S
C10H14O4	C24H26O6S3	C10H22O7S3	C17H32O6S
C12H18O4	C18H14O6S3	C11H24O7S3	C18H34O6S
C15H24O4	C20H18O6S3	C12H26O7S3	C19H36O6S
C16H26O4	C21H20O6S3	C14H30O7S3	C20H38O6S
C17H28O4	C20H16O6S3	C15H32O7S3	C21H40O6S
C21H36O4	C21H18O6S3	C17H36O7S3	C22H42O6S
C10H12O4	C12H26O7	C18H38O7S3	C6H10O6S
C11H14O4	C13H28O7	C19H40O7S3	C7H12O6S
C15H22O4	C14H30O7	C13H28O7S3	C8H14O6S
C16H24O4	C15H32O7	C23H48O7S3	C9H16O6S
C22H36O4	C16H34O7	C9H20O7S3	C14H24O6S
C9H10O4	C11H22O7	C11H22O7S3	C10H16O6S
C12H16O4	C12H24O7	C13H26O7S3	C11H18O6S
C13H18O4	C13H26O7	C14H28O7S3	C12H20O6S
C18H28O4	C14H28O7	C15H30O7S3	C13H22O6S
C20H32O4	C15H30O7	C17H34O7S3	C15H26O6S
C21H34O4	C16H32O7	C18H36O7S3	C18H32O6S
C11H12O4	C17H34O7	C10H20O7S3	C19H34O6S
C14H18O4	C18H36O7	C12H24O7S3	C20H36O6S
C17H24O4	C19H38O7	C23H46O7S3	C21H38O6S
C18H26O4	C20H40O7	C9H18O7S3	C22H40O6S
C19H28O4	C10H18O7	C14H26O7S3	C23H42O6S
C20H30O4	C11H20O7	C17H32O7S3	C7H10O6S
C22H34O4	C12H22O7	C11H20O7S3	C8H12O6S
C10H10O4	C13H24O7	C12H22O7S3	C9H14O6S
C12H14O4	C15H28O7	C13H24O7S3	C11H16O6S
C13H16O4	C16H30O7	C23H44O7S3	C12H18O6S
C15H20O4	C17H32O7	C16H28O7S3	C13H20O6S
C16H22O4	C20H38O7	C17H30O7S3	C14H22O6S
C21H32O4	C21H40O7	C18H30O7S3	C15H24O6S
C23H36O4	C22H42O7	C15H24O7S3	C16H26O6S
C28H46O4	C23H44O7	C22H26O7S3	C18H30O6S
C11H10O4	C24H46O7	C23H28O7S3	C21H36O6S

C12H12O4	C18H34O7	C25H32O7S3		C8H10O6S
C13H14O4	C19H36O7	C26H34O7S3		C9H12O6S
C14H16O4	C8H14O7	C24H28O7S3		C10H12O6S
C15H18O4	C9H16O7	C22H22O7S3		C11H14O6S
C19H26O4	C10H16O7	C21H18O7S3		C12H16O6S
C20H28O4	C11H18O7	C22H20O7S3		C13H18O6S
C21H30O4	C13H22O7	C24H18O7S3		C14H20O6S
C23H34O4	C14H24O7	C13H28O8		C15H22O6S
C25H38O4	C15H26O7	C14H30O8		C16H24O6S
C27H42O4	C22H40O7	C15H32O8		C10H10O6S
C16H20O4	C23H42O7	C17H36O8		C11H12O6S
C17H22O4	C24H44O7	C12H24O8		C12H14O6S
C18H24O4	C25H46O7	C13H26O8		C13H16O6S
C12H10O4	C26H48O7	C14H28O8		C14H18O6S
C13H12O4	C27H50O7	C15H30O8		C15H20O6S
C14H14O4	C29H54O7	C16H32O8		C16H22O6S
C15H16O4	C9H14O7	C17H34O8		C17H24O6S
C17H20O4	C12H20O7	C18H36O8		C29H46O6S
C19H24O4	C16H28O7	C19H38O8		C30H48O6S
C20H26O4	C17H30O7	C20H40O8		C12H12O6S
C21H28O4	C18H32O7	C21H42O8		C13H14O6S
C22H30O4	C19H34O7	C22H44O8		C14H16O6S
C23H32O4	C20H36O7	C11H20O8		C15H18O6S
C24H34O4	C21H38O7	C12H22O8		C16H20O6S
C25H36O4	C8H12O7	C13H24O8		C17H22O6S
C16H18O4	C10H14O7	C14H26O8		C29H44O6S
C14H12O4	C11H16O7	C15H28O8		C14H14O6S
C18H20O4	C12H18O7	C17H32O8		C15H16O6S
C23H30O4	C14H22O7	C18H34O8		C16H18O6S
C25H34O4	C15H24O7	C19H36O8		C20H24O6S
C27H38O4	C17H28O7	C20H38O8		C15H14O6S
C13H10O4	C18H30O7	C21H40O8		C18H20O6S
C15H14O4	C23H40O7	C22H42O8		C28H38O6S
C16H16O4	C24H42O7	C23H44O8		C15H12O6S
C17H18O4	C25H44O7	C10H18O8		C16H14O6S
C18H18O4	C26H46O7	C9H16O8		C18H18O6S
C23H28O4	C13H20O7	C10H16O8		C26H30O6S
C14H10O4	C16H26O7	C11H18O8		C32H42O6S
C15H12O4	C20H34O7	C12H20O8		C27H30O6S
C16H14O4	C21H36O7	C13H22O8		C32H40O6S
C15H10O4	C22H38O7	C14H24O8		C41H58O6S
C16H12O4	C8H10O7	C15H26O8		C30H34O6S
C17H14O4	C9H12O7	C16H28O8		C13H20O6S2
C24H26O4	C12H16O7	C17H30O8		C14H22O6S2

C25H28O4	C14H20O7	C18H32O8	C12H18O6S2
C26H30O4	C15H22O7	C21H38O8	C12H16O6S2
C20H16O4	C16H24O7	C22H40O8	C14H20O6S2
C23H22O4	C17H26O7	C23H42O8	C12H14O6S2
C25H26O4	C18H28O7	C24H44O8	C14H18O6S2
C26H28O4	C19H30O7	C26H48O8	C14H16O6S2
C27H30O4	C20H32O7	C20H36O8	C19H20O6S2
C29H34O4	C25H42O7	C9H14O8	C19H18O6S2
C30H36O4	C26H44O7	C11H16O8	C22H24O6S2
C31H38O4	C10H12O7	C12H18O8	C28H32O6S2
C32H40O4	C11H14O7	C13H20O8	C30H36O6S2
C33H42O4	C13H18O7	C14H22O8	C23H12O6S2
C34H44O4	C21H34O7	C15H24O8	C12H26O6S3
C35H46O4	C22H36O7	C16H26O8	C14H30O6S3
C36H48O4	C23H38O7	C17H28O8	C15H32O6S3
C21H16O4	C24H40O7	C18H30O8	C28H58O6S3
C22H18O4	C9H10O7	C24H42O8	C10H22O6S3
C23H20O4	C13H16O7	C10H14O8	C11H24O6S3
C24H22O4	C15H20O7	C19H32O8	C13H28O6S3
C25H24O4	C16H22O7	C20H34O8	C23H48O6S3
C26H26O4	C17H24O7	C21H36O8	C9H20O6S3
C27H28O4	C18H26O7	C22H38O8	C12H24O6S3
C28H30O4	C19H28O7	C10H12O8	C13H26O6S3
C29H32O4	C20H30O7	C11H14O8	C14H28O6S3
C30H34O4	C21H32O7	C12H16O8	C15H30O6S3
C31H36O4	C22H34O7	C13H18O8	C20H40O6S3
C32H38O4	C23H36O7	C14H20O8	C28H56O6S3
C33H40O4	C24H38O7	C15H22O8	C10H20O6S3
C34H42O4	C25H40O7	C16H24O8	C11H22O6S3
C35H44O4	C29H48O7	C17H26O8	C16H32O6S3
C36H46O4	C10H10O7	C18H28O8	C23H46O6S3
C23H18O4	C11H12O7	C19H30O8	C9H18O6S3
C24H20O4	C12H14O7	C20H32O8	C12H22O6S3
C25H22O4	C14H18O7	C21H34O8	C20H38O6S3
C26H24O4	C14H16O7	C22H36O8	C22H42O6S3
C27H26O4	C15H18O7	C23H38O8	C24H46O6S3
C28H28O4	C16H20O7	C24H40O8	C23H44O6S3
C29H30O4	C17H22O7	C9H10O8	C14H24O6S3
C30H32O4	C18H24O7	C15H20O8	C17H30O6S3
C31H34O4	C19H26O7	C16H22O8	C24H32O6S3
C32H36O4	C20H28O7	C17H24O8	C17H16O6S3
C33H38O4	C21H30O7	C18H26O8	C18H14O6S3
C34H40O4	C22H32O7	C19H28O8	C20H18O6S3
C35H42O4	C23H34O7	C20H30O8	C21H20O6S3

C36H44O4	C25H38O7	C21H32O8	C22H22O6S3
C37H46O4	C11H10O7	C22H34O8	C23H24O6S3
C22H14O4	C12H12O7	C23H36O8	C20H16O6S3
C23H16O4	C13H14O7	C24H38O8	C22H20O6S3
C24H18O4	C15H16O7	C25H40O8	C36H22O6S3
C25H20O4	C16H18O7	C11H12O8	C12H26O7
C26H22O4	C17H20O7	C12H14O8	C13H28O7
C27H24O4	C18H22O7	C13H16O8	C14H30O7
C28H26O4	C19H24O7	C14H18O8	C15H32O7
C29H28O4	C20H26O7	C15H18O8	C16H34O7
C30H30O4	C21H28O7	C16H20O8	C10H20O7
C31H32O4	C22H30O7	C17H22O8	C11H22O7
C32H34O4	C27H40O7	C18H24O8	C12H24O7
C33H36O4	C12H10O7	C19H26O8	C13H26O7
C34H38O4	C13H12O7	C20H28O8	C14H28O7
C25H18O4	C14H14O7	C21H30O8	C15H30O7
C26H20O4	C23H32O7	C22H32O8	C16H32O7
C27H22O4	C24H34O7	C23H34O8	C17H34O7
C28H24O4	C15H14O7	C24H36O8	C18H36O7
C29H26O4	C16H16O7	C11H10O8	C19H38O7
C24H14O4	C17H18O7	C12H12O8	C8H16O7
C25H16O4	C18H20O7	C13H14O8	C6H12O7
C27H20O4	C19H22O7	C14H16O8	C10H18O7
C35H22O4	C20H24O7	C17H20O8	C11H20O7
C8H18O4S	C21H26O7	C18H22O8	C12H22O7
C9H20O4S	C22H28O7	C19H24O8	C13H24O7
C13H28O4S	C13H10O7	C20H26O8	C15H28O7
C16H34O4S	C14H12O7	C21H28O8	C16H30O7
C11H22O4S	C23H30O7	C22H30O8	C17H32O7
C6H12O4S	C26H36O7	C23H32O8	C20H38O7
C7H14O4S	C19H20O7	C12H10O8	C21H40O7
C8H16O4S	C20H22O7	C13H12O8	C22H42O7
C9H18O4S	C21H24O7	C14H14O8	C23H44O7
C10H20O4S	C14H10O7	C15H16O8	C24H46O7
C17H34O4S	C15H12O7	C16H18O8	C26H50O7
C18H36O4S	C16H14O7	C24H34O8	C9H16O7
C19H38O4S	C17H16O7	C18H20O8	C14H26O7
C10H18O4S	C18H18O7	C19H22O8	C18H34O7
C18H34O4S	C22H26O7	C20H24O8	C19H36O7
C8H14O4S	C15H10O7	C21H26O8	C7H12O7
C10H16O4S	C16H12O7	C13H10O8	C8H14O7
C11H18O4S	C17H14O7	C14H12O8	C10H16O7
C21H38O4S	C18H16O7	C15H14O8	C11H18O7
C23H42O4S	C19H18O7	C16H16O8	C13H22O7

C18H32O4S	C20H20O7	C17H18O8	C14H24O7
C19H34O4S	C21H22O7	C22H28O8	C15H26O7
C20H36O4S	C22H24O7	C23H30O8	C22H40O7
C7H10O4S	C20H18O7	C20H22O8	C23H42O7
C8H12O4S	C17H12O7	C21H24O8	C24H44O7
C9H14O4S	C18H14O7	C14H10O8	C25H46O7
C10H14O4S	C19H16O7	C15H12O8	C26H48O7
C11H16O4S	C21H20O7	C16H14O8	C27H50O7
C12H18O4S	C22H22O7	C17H16O8	C9H14O7
C13H20O4S	C24H26O7	C18H18O8	C12H20O7
C15H24O4S	C26H28O7	C19H20O8	C16H28O7
C18H30O4S	C17H10O7	C22H26O8	C17H30O7
C8H10O4S	C18H12O7	C23H28O8	C18H32O7
C9H12O4S	C19H14O7	C15H10O8	C19H34O7
C11H14O4S	C20H16O7	C16H12O8	C20H36O7
C10H12O4S	C21H18O7	C17H14O8	C21H38O7
C12H16O4S	C22H20O7	C18H16O8	C7H10O7
C13H18O4S	C23H22O7	C19H18O8	C11H16O7
C27H46O4S	C24H22O7	C20H20O8	C12H18O7
C9H10O4S	C25H24O7	C21H22O8	C14H22O7
C10H10O4S	C26H26O7	C22H24O8	C15H24O7
C11H12O4S	C21H14O7	C23H26O8	C17H28O7
C12H14O4S	C22H16O7	C24H28O8	C18H30O7
C13H16O4S	C23H18O7	C17H12O8	C23H40O7
C14H18O4S	C24H20O7	C18H14O8	C24H42O7
C11H10O4S	C25H22O7	C19H16O8	C25H44O7
C13H14O4S	C26H24O7	C20H18O8	C26H46O7
C14H16O4S	C27H26O7	C21H20O8	C27H48O7
C12H10O4S	C28H28O7	C22H22O8	C10H14O7
C13H12O4S	C24H18O7	C23H24O8	C13H20O7
C17H20O4S	C25H20O7	C24H26O8	C16H26O7
C30H44O4S	C26H22O7	C20H16O8	C20H34O7
C15H14O4S	C22H14O7	C22H20O8	C21H36O7
C16H16O4S	C26H18O7	C24H24O8	C22H38O7
C17H18O4S	C23H10O7	C25H26O8	C8H10O7
C18H20O4S	C27H18O7	C19H14O8	C9H12O7
C20H24O4S	C26H14O7	C21H18O8	C12H16O7
C22H28O4S	C26H12O7	C23H22O8	C13H18O7
C16H14O4S	C27H14O7	C23H20O8	C14H20O7
C17H16O4S	C35H22O7	C23H18O8	C15H22O7
C18H18O4S	C34H14O7	C25H22O8	C16H24O7
C19H20O4S	C11H24O7S3	C22H16O8	C17H26O7
C20H22O4S	C12H26O7S3	C23H16O8	C18H28O7
C29H40O4S	C14H30O7S3	C24H18O8	C19H30O7

C30H42O4S	C15H32O7S3	C26H22O8	C20H32O7
C32H44O4S	C17H36O7S3	C28H18O8	C25H42O7
C33H46O4S	C18H38O7S3	C34H18O8	C26H44O7
C34H48O4S	C19H40O7S3	C42H18O8	C10H12O7
C18H16O4S	C10H22O7S3	C11H22O8S	C11H14O7
C19H18O4S	C13H28O7S3	C13H26O8S	C21H34O7
C27H34O4S	C16H34O7S3	C19H38O8S	C22H36O7
C29H38O4S	C9H20O7S3	C20H40O8S	C23H38O7
C32H42O4S	C12H24O7S3	C10H18O8S	C24H40O7
C36H50O4S	C13H26O7S3	C11H20O8S	C27H46O7
C19H16O4S	C14H28O7S3	C12H22O8S	C9H10O7
C20H18O4S	C15H30O7S3	C13H24O8S	C13H16O7
C28H34O4S	C16H32O7S3	C14H26O8S	C15H20O7
C21H18O4S	C17H34O7S3	C15H28O8S	C16H22O7
C27H30O4S	C18H36O7S3	C16H30O8S	C17H24O7
C29H34O4S	C19H38O7S3	C18H34O8S	C18H26O7
C30H36O4S	C10H20O7S3	C20H38O8S	C19H28O7
C31H36O4S	C11H22O7S3	C22H42O8S	C20H30O7
C37H48O4S	C23H46O7S3	C7H12O8S	C21H32O7
C40H54O4S	C25H50O7S3	C8H14O8S	C22H34O7
C30H34O4S	C9H18O7S3	C9H16O8S	C23H36O7
C23H18O4S	C15H28O7S3	C10H16O8S	C24H38O7
C29H28O4S	C16H30O7S3	C11H18O8S	C25H40O7
C9H16O4S2	C18H34O7S3	C12H20O8S	C10H10O7
C9H14O4S2	C11H20O7S3	C13H22O8S	C11H12O7
C10H16O4S2	C12H22O7S3	C14H24O8S	C12H14O7
C7H10O4S2	C13H24O7S3	C15H26O8S	C14H18O7
C8H12O4S2	C14H26O7S3	C16H28O8S	C26H42O7
C16H26O4S2	C16H28O7S3	C18H32O8S	C27H44O7
C8H10O4S2	C17H30O7S3	C20H36O8S	C13H14O7
C9H12O4S2	C18H32O7S3	C22H40O8S	C14H16O7
C15H22O4S2	C12H20O7S3	C8H12O8S	C15H18O7
C16H24O4S2	C19H32O7S3	C9H14O8S	C16H20O7
C17H26O4S2	C15H24O7S3	C10H14O8S	C17H22O7
C11H14O4S2	C22H26O7S3	C11H16O8S	C18H24O7
C9H10O4S2	C23H28O7S3	C12H18O8S	C19H26O7
C12H10O4S2	C26H34O7S3	C13H20O8S	C20H28O7
C13H12O4S2	C23H26O7S3	C14H22O8S	C21H30O7
C14H14O4S2	C25H30O7S3	C15H24O8S	C22H32O7
C24H34O4S2	C24H28O7S3	C18H30O8S	C23H34O7
C26H38O4S2	C22H22O7S3	C20H34O8S	C24H36O7
C14H12O4S2	C24H26O7S3	C8H10O8S	C25H38O7
C16H10O4S2	C22H20O7S3	C9H12O8S	C27H42O7
C17H12O4S2	C24H18O7S3	C10H12O8S	C11H10O7

C29H28O4S2	C25H14O7S3	C11H14O8S	C12H12O7
C36H32O4S2	C13H28O8	C12H16O8S	C16H18O7
C10H22O4S3	C14H30O8	C13H18O8S	C17H20O7
C11H24O4S3	C15H32O8	C14H20O8S	C18H22O7
C12H26O4S3	C16H34O8	C15H22O8S	C19H24O7
C13H28O4S3	C17H36O8	C10H10O8S	C20H26O7
C14H30O4S3	C18H38O8	C11H12O8S	C21H28O7
C17H36O4S3	C19H40O8	C12H14O8S	C22H30O7
C18H38O4S3	C12H24O8	C13H16O8S	C23H32O7
C19H40O4S3	C13H26O8	C14H18O8S	C25H36O7
C21H44O4S3	C14H28O8	C15H20O8S	C27H40O7
C22H46O4S3	C15H30O8	C16H22O8S	C29H44O7
C24H50O4S3	C16H32O8	C17H24O8S	C12H10O7
C25H52O4S3	C17H34O8	C13H14O8S	C13H12O7
C26H54O4S3	C18H36O8	C14H16O8S	C14H14O7
C9H20O4S3	C19H38O8	C15H18O8S	C15H16O7
C20H42O4S3	C20H40O8	C16H20O8S	C24H34O7
C8H18O4S3	C21H42O8	C17H22O8S	C15H14O7
C10H20O4S3	C22H44O8	C18H24O8S	C18H20O7
C11H22O4S3	C12H22O8	C13H12O8S	C19H22O7
C12H24O4S3	C13H24O8	C14H14O8S	C21H26O7
C13H26O4S3	C14H26O8	C15H16O8S	C23H30O7
C15H30O4S3	C15H28O8	C16H18O8S	C13H10O7
C17H34O4S3	C17H32O8	C17H20O8S	C14H12O7
C19H38O4S3	C18H34O8	C18H22O8S	C16H16O7
C20H40O4S3	C19H36O8	C16H16O8S	C17H18O7
C22H44O4S3	C20H38O8	C17H18O8S	C20H24O7
C8H16O4S3	C21H40O8	C18H20O8S	C22H28O7
C11H20O4S3	C22H42O8	C19H22O8S	C25H34O7
C12H22O4S3	C23H44O8	C16H14O8S	C19H20O7
C13H24O4S3	C25H48O8	C18H18O8S	C20H22O7
C14H26O4S3	C26H50O8	C27H16O8S	C21H24O7
C15H28O4S3	C8H14O8	C40H16O8S	C14H10O7
C17H32O4S3	C9H16O8	C16H34O8S3	C15H12O7
C19H36O4S3	C10H16O8	C17H36O8S3	C16H14O7
C20H38O4S3	C11H18O8	C19H40O8S3	C17H16O7
C22H42O4S3	C12H20O8	C10H22O8S3	C18H18O7
C12H20O4S3	C13H22O8	C11H24O8S3	C22H26O7
C13H22O4S3	C14H24O8	C12H26O8S3	C24H30O7
C14H24O4S3	C15H26O8	C13H28O8S3	C21H22O7
C16H28O4S3	C16H28O8	C14H30O8S3	C15H10O7
C18H32O4S3	C17H30O8	C23H48O8S3	C16H12O7
C20H36O4S3	C18H32O8	C25H52O8S3	C17H14O7
C22H40O4S3	C21H38O8	C13H26O8S3	C18H16O7

C14H20O4S3	C22H40O8	C14H28O8S3	C19H18O7
C19H20O4S3	C23H42O8	C17H34O8S3	C20H20O7
C20H22O4S3	C24H44O8	C18H36O8S3	C22H24O7
C22H26O4S3	C25H46O8	C10H20O8S3	C23H26O7
C18H16O4S3	C26H48O8	C11H22O8S3	C24H28O7
C19H18O4S3	C28H52O8	C12H24O8S3	C21H20O7
C32H44O4S3	C29H54O8	C23H46O8S3	C17H12O7
C35H50O4S3	C30H56O8	C25H50O8S3	C18H14O7
C17H12O4S3	C19H34O8	C17H32O8S3	C19H16O7
C18H14O4S3	C20H36O8	C18H34O8S3	C20H18O7
C19H16O4S3	C9H14O8	C12H22O8S3	C22H22O7
C20H18O4S3	C12H18O8	C13H24O8S3	C18H12O7
C31H40O4S3	C13H20O8	C22H24O8S3	C19H14O7
C33H44O4S3	C14H22O8	C18H12O8S3	C20H16O7
C34H46O4S3	C15H24O8	C9H20O9	C21H18O7
C22H20O4S3	C16H26O8	C12H24O9	C22H20O7
C34H44O4S3	C17H28O8	C13H26O9	C23H22O7
C28H22O4S3	C18H30O8	C14H28O9	C24H22O7
C33H22O4S3	C24H42O8	C15H30O9	C25H24O7
C10H22O5	C25H44O8	C16H32O9	C20H12O7
C11H24O5	C27H48O8	C17H34O9	C21H14O7
C9H20O5	C28H50O8	C18H36O9	C22H16O7
C14H28O5	C29H52O8	C19H38O9	C23H18O7
C15H30O5	C40H74O8	C20H40O9	C24H20O7
C16H32O5	C10H14O8	C21H42O9	C25H22O7
C17H34O5	C11H16O8	C22H44O9	C26H24O7
C18H36O5	C20H34O8	C23H46O9	C34H40O7
C19H38O5	C21H36O8	C9H18O9	C35H42O7
C20H40O5	C22H38O8	C12H22O9	C28H26O7
C21H42O5	C10H12O8	C13H24O9	C32H34O7
C22H44O5	C11H14O8	C14H26O9	C26H18O7
C23H46O5	C12H16O8	C15H28O9	C24H14O7
C5H10O5	C13H18O8	C16H30O9	C31H18O7
C10H20O5	C14H20O8	C17H32O9	C34H20O7
C11H22O5	C15H22O8	C18H34O9	C35H22O7
C12H24O5	C16H24O8	C19H36O9	C10H22O7S
C6H12O5	C17H26O8	C20H38O9	C18H38O7S
C7H14O5	C19H30O8	C21H40O9	C20H42O7S
C8H16O5	C20H32O8	C23H44O9	C10H20O7S
C9H18O5	C21H34O8	C13H22O9	C11H22O7S
C17H32O5	C22H36O8	C14H24O9	C12H24O7S
C23H44O5	C23H38O8	C15H26O9	C13H26O7S
C24H46O5	C24H40O8	C16H28O9	C14H28O7S
C25H48O5	C9H10O8	C17H30O9	C18H36O7S

C10H18O5	C18H28O8	C18H32O9	C20H40O7S
C11H20O5	C18H26O8	C19H34O9	C6H12O7S
C15H28O5	C19H28O8	C21H38O9	C7H14O7S
C16H30O5	C20H30O8	C22H40O9	C8H16O7S
C19H36O5	C21H32O8	C20H36O9	C9H18O7S
C20H38O5	C22H34O8	C23H42O9	C10H18O7S
C21H40O5	C23H36O8	C10H14O9	C11H20O7S
C22H42O5	C24H38O8	C11H16O9	C12H22O7S
C6H10O5	C25H40O8	C12H18O9	C13H24O7S
C7H12O5	C31H52O8	C13H20O9	C14H26O7S
C8H14O5	C32H54O8	C14H22O9	C16H30O7S
C9H16O5	C33H56O8	C15H24O9	C18H34O7S
C23H42O5	C10H10O8	C16H26O9	C19H36O7S
C24H44O5	C11H12O8	C17H28O9	C20H38O7S
C25H46O5	C12H14O8	C18H30O9	C21H40O7S
C26H48O5	C13H16O8	C19H32O9	C22H42O7S
C7H10O5	C14H18O8	C20H34O9	C23H44O7S
C8H12O5	C15H20O8	C21H36O9	C6H10O7S
C10H16O5	C16H22O8	C22H38O9	C7H12O7S
C11H18O5	C17H24O8	C23H40O9	C8H14O7S
C12H20O5	C15H18O8	C25H44O9	C9H16O7S
C14H24O5	C16H20O8	C9H12O9	C9H14O7S
C15H26O5	C17H22O8	C24H42O9	C10H16O7S
C16H28O5	C18H24O8	C12H16O9	C11H18O7S
C19H34O5	C19H26O8	C14H20O9	C12H20O7S
C20H36O5	C20H28O8	C15H22O9	C13H22O7S
C21H38O5	C21H30O8	C16H24O9	C14H24O7S
C22H40O5	C22H32O8	C17H26O9	C15H26O7S
C9H14O5	C23H34O8	C18H28O9	C16H28O7S
C13H20O5	C24H36O8	C19H30O9	C17H30O7S
C23H40O5	C25H38O8	C20H32O9	C18H32O7S
C24H42O5	C27H42O8	C21H34O9	C19H34O7S
C25H44O5	C11H10O8	C22H36O9	C20H36O7S
C26H46O5	C12H12O8	C24H40O9	C21H38O7S
C8H10O5	C13H14O8	C25H42O9	C22H40O7S
C9H12O5	C14H16O8	C11H14O9	C23H42O7S
C10H14O5	C16H18O8	C13H18O9	C24H44O7S
C11H16O5	C17H20O8	C11H12O9	C25H46O7S
C12H18O5	C18H22O8	C12H14O9	C7H10O7S
C17H28O5	C19H24O8	C13H16O9	C8H12O7S
C18H30O5	C20H26O8	C14H18O9	C10H14O7S
C20H34O5	C21H28O8	C18H26O9	C11H16O7S
C21H36O5	C22H30O8	C19H28O9	C12H18O7S
C10H12O5	C23H32O8	C20H30O9	C13H20O7S

C11H14O5	C24H34O8	C21H32O9	C14H22O7S
C12H16O5	C25H36O8	C22H34O9	C15H24O7S
C13H18O5	C27H40O8	C23H36O9	C16H26O7S
C15H22O5	C12H10O8	C24H38O9	C18H30O7S
C18H28O5	C13H12O8	C25H40O9	C19H32O7S
C21H34O5	C14H14O8	C27H44O9	C20H34O7S
C24H40O5	C15H16O8	C15H20O9	C21H36O7S
C25H42O5	C18H20O8	C16H22O9	C26H46O7S
C9H10O5	C19H22O8	C17H24O9	C8H10O7S
C14H20O5	C20H24O8	C18H24O9	C9H12O7S
C16H24O5	C21H26O8	C19H26O9	C11H14O7S
C17H26O5	C13H10O8	C20H28O9	C12H16O7S
C19H30O5	C14H12O8	C21H30O9	C13H18O7S
C22H36O5	C15H14O8	C22H32O9	C14H20O7S
C23H38O5	C16H16O8	C23H34O9	C15H22O7S
C11H12O5	C17H18O8	C24H36O9	C16H24O7S
C12H14O5	C22H28O8	C13H14O9	C17H26O7S
C13H16O5	C23H30O8	C14H16O9	C18H28O7S
C14H18O5	C25H34O8	C15H18O9	C26H44O7S
C15H20O5	C21H24O8	C16H20O9	C28H48O7S
C16H22O5	C14H10O8	C17H22O9	C9H10O7S
C17H24O5	C15H12O8	C18H22O9	C10H10O7S
C18H26O5	C16H14O8	C19H24O9	C11H12O7S
C19H28O5	C17H16O8	C20H26O9	C12H14O7S
C20H30O5	C18H18O8	C21H28O9	C13H16O7S
C21H32O5	C19H20O8	C22H30O9	C14H18O7S
C24H38O5	C20H22O8	C23H32O9	C15H20O7S
C10H10O5	C22H26O8	C14H14O9	C16H22O7S
C25H40O5	C23H28O8	C15H16O9	C17H24O7S
C11H10O5	C24H30O8	C16H18O9	C26H42O7S
C12H12O5	C16H12O8	C17H20O9	C28H46O7S
C13H14O5	C17H14O8	C24H34O9	C12H12O7S
C14H16O5	C18H16O8	C25H36O9	C14H16O7S
C15H18O5	C19H18O8	C20H24O9	C15H18O7S
C16H20O5	C20H20O8	C14H12O9	C16H20O7S
C17H22O5	C21H22O8	C15H14O9	C17H22O7S
C18H24O5	C22H24O8	C16H16O9	C18H24O7S
C19H26O5	C23H26O8	C17H18O9	C19H26O7S
C20H28O5	C24H28O8	C18H20O9	C12H10O7S
C21H30O5	C17H12O8	C19H22O9	C13H12O7S
C22H32O5	C18H14O8	C21H26O9	C14H14O7S
C23H34O5	C19H16O8	C22H28O9	C15H16O7S
C24H36O5	C20H18O8	C16H14O9	C16H18O7S
C25H38O5	C21H20O8	C17H16O9	C17H20O7S

C27H42O5	C22H22O8	C18H18O9	C18H22O7S
C13H12O5	C23H24O8	C19H20O9	C19H24O7S
C14H14O5	C24H26O8	C20H22O9	C15H14O7S
C15H16O5	C20H16O8	C21H24O9	C16H16O7S
C16H18O5	C22H20O8	C22H26O9	C17H18O7S
C17H20O5	C24H24O8	C23H28O9	C18H20O7S
C18H22O5	C19H14O8	C24H30O9	C19H22O7S
C19H24O5	C21H18O8	C16H12O9	C16H14O7S
C20H26O5	C23H22O8	C17H14O9	C30H38O7S
C21H28O5	C21H16O8	C18H16O9	C12H26O7S3
C22H30O5	C28H18O8	C19H18O9	C14H30O7S3
C23H32O5	C28H16O8	C20H20O9	C10H22O7S3
C25H36O5	C31H20O8	C21H22O9	C11H24O7S3
C12H10O5	C33H16O8	C22H24O9	C13H28O7S3
C14H12O5	C34H18O8	C23H26O9	C15H32O7S3
C15H14O5	C42H18O8	C24H28O9	C23H48O7S3
C17H18O5	C13H26O8S	C17H12O9	C25H52O7S3
C19H22O5	C20H40O8S	C18H14O9	C26H54O7S3
C21H26O5	C10H18O8S	C19H16O9	C9H20O7S3
C23H30O5	C11H20O8S	C20H18O9	C11H22O7S3
C24H32O5	C12H22O8S	C21H20O9	C13H26O7S3
C25H34O5	C13H24O8S	C22H22O9	C14H28O7S3
C26H36O5	C14H26O8S	C23H24O9	C10H20O7S3
C13H10O5	C18H34O8S	C24H26O9	C23H46O7S3
C16H16O5	C20H38O8S	C19H14O9	C25H50O7S3
C18H20O5	C22H42O8S	C20H16O9	C9H18O7S3
C20H24O5	C8H14O8S	C21H18O9	C11H20O7S3
C16H14O5	C9H16O8S	C22H20O9	C12H22O7S3
C17H16O5	C10H16O8S	C23H22O9	C13H24O7S3
C27H36O5	C11H18O8S	C24H24O9	C14H26O7S3
C14H10O5	C12H20O8S	C25H26O9	C15H26O7S3
C15H12O5	C13H22O8S	C26H28O9	C12H20O7S3
C18H18O5	C14H24O8S	C24H22O9	C15H24O7S3
C20H22O5	C16H28O8S	C25H24O9	C22H26O7S3
C21H24O5	C18H32O8S	C26H26O9	C23H28O7S3
C31H42O5	C20H36O8S	C31H12O9	C24H30O7S3
C15H10O5	C22H40O8S	C49H32O9	C25H32O7S3
C16H12O5	C8H12O8S	C42H12O9	C26H34O7S3
C17H14O5	C9H14O8S	C17H36O9S3	C24H28O7S3
C18H16O5	C10H14O8S	C11H24O9S3	C22H22O7S3
C19H18O5	C11H16O8S	C12H26O9S3	C21H18O7S3
C21H20O5	C12H18O8S	C13H28O9S3	C22H20O7S3
C22H22O5	C13H20O8S	C14H30O9S3	C13H28O8
C18H14O5	C14H22O8S	C12H22O9S3	C14H30O8

C22H20O5	C15H24O8S	C25H28O9S3	C15H32O8
C24H24O5	C18H30O8S	C12H22OS	C16H34O8
C25H26O5	C20H34O8S	C13H22OS	C17H36O8
C26H28O5	C9H12O8S	C15H26OS	C19H40O8
C27H30O5	C10H12O8S	C25H44OS	C12H24O8
C28H32O5	C11H14O8S	C9H12OS	C13H26O8
C22H18O5	C12H16O8S	C15H22OS	C14H28O8
C23H20O5	C13H18O8S	C17H26OS	C15H30O8
C24H22O5	C14H20O8S	C18H28OS	C16H32O8
C25H24O5	C15H22O8S	C17H24OS	C17H34O8
C26H26O5	C16H24O8S	C18H26OS	C18H36O8
C27H28O5	C11H12O8S	C18H24OS	C19H38O8
C28H30O5	C12H14O8S	C18H22OS	C20H40O8
C29H32O5	C13H16O8S	C12H10OS	C21H42O8
C30H34O5	C14H18O8S	C14H14OS	C22H44O8
C31H36O5	C15H20O8S	C15H16OS	C8H16O8
C32H38O5	C16H22O8S	C17H18OS	C11H20O8
C33H40O5	C17H24O8S	C18H20OS	C12H22O8
C34H42O5	C18H26O8S	C13H10OS	C13H24O8
C35H44O5	C12H12O8S	C14H12OS	C14H26O8
C22H16O5	C13H14O8S	C15H14OS	C15H28O8
C23H18O5	C14H16O8S	C19H20OS	C17H32O8
C24H20O5	C15H18O8S	C14H10OS	C18H34O8
C25H22O5	C16H20O8S	C15H12OS	C19H36O8
C26H24O5	C17H22O8S	C27H36OS	C20H38O8
C27H26O5	C18H24O8S	C24H28OS	C21H40O8
C28H28O5	C19H26O8S	C21H20OS	C22H42O8
C29H30O5	C14H14O8S	C25H24OS	C23H44O8
C30H32O5	C15H16O8S	C27H28OS	C25H48O8
C31H34O5	C16H18O8S	C28H30OS	C26H50O8
C32H36O5	C17H20O8S	C29H32OS	C10H18O8
C33H38O5	C18H22O8S	C20H12OS	C24H46O8
C34H40O5	C19H24O8S	C42H32OS	C8H14O8
C35H42O5	C16H16O8S	C10H18OS2	C9H16O8
C36H44O5	C17H18O8S	C12H12OS2	C11H18O8
C37H46O5	C18H20O8S	C19H26OS2	C12H20O8
C23H16O5	C19H22O8S	C13H14OS2	C13H22O8
C24H18O5	C18H18O8S	C15H16OS2	C14H24O8
C25H20O5	C19H20O8S	C16H18OS2	C15H26O8
C26H22O5	C17H14O8S	C17H20OS2	C16H28O8
C27H24O5	C24H10O8S	C16H16OS2	C17H30O8
C28H26O5	C27H16O8S	C17H18OS2	C18H32O8
C29H28O5	C40H16O8S	C18H20OS2	C21H38O8
C30H30O5	C15H32O8S3	C19H22OS2	C22H40O8

C31H32O5	C16H34O8S3	C20H24OS2		C23H42O8
C32H34O5	C17H36O8S3	C23H30OS2		C24H44O8
C33H36O5	C19H40O8S3	C27H38OS2		C25H46O8
C34H38O5	C11H24O8S3	C29H42OS2		C26H48O8
C35H40O5	C12H26O8S3	C18H18OS2		C10H16O8
C36H42O5	C13H28O8S3	C19H20OS2		C19H34O8
C37H44O5	C14H30O8S3	C20H22OS2		C20H36O8
C23H14O5	C23H48O8S3	C18H16OS2		C9H14O8
C24H16O5	C25H52O8S3	C19H18OS2		C12H18O8
C25H18O5	C27H56O8S3	C20H20OS2		C13H20O8
C26H20O5	C17H34O8S3	C20H18OS2		C14H22O8
C27H22O5	C10H20O8S3	C22H22OS2		C15H24O8
C28H24O5	C11H22O8S3	C24H26OS2		C16H26O8
C29H26O5	C12H24O8S3	C18H14OS2		C17H28O8
C30H28O5	C13H26O8S3	C21H20OS2		C18H30O8
C31H30O5	C14H28O8S3	C18H10OS2		C24H42O8
C25H16O5	C23H46O8S3	C36H42OS2		C30H54O8
C26H18O5	C25H50O8S3	C35H26OS2		C10H14O8
C27H20O5	C17H32O8S3	C30H12OS2		C11H16O8
C28H22O5	C18H34O8S3	C32H16OS2		C19H32O8
C24H14O5	C12H22O8S3	C32H14OS2		C20H34O8
C24H12O5	C13H24O8S3	C34H12OS2		C21H36O8
C25H14O5	C9H16O8S3	C9H14OS3		C22H38O8
C25H12O5	C15H24O8S3	C17H14OS3		C23H40O8
C32H14O5	C22H24O8S3	C19H18OS3		C10H12O8
C34H18O5	C24H28O8S3	C21H14OS3		C11H14O8
C9H20O5S	C25H30O8S3	C25H16OS3		C12H16O8
C10H20O5S	C28H36O8S3	C33H18OS3		C13H18O8
C11H22O5S	C18H12O8S3	C36H24OS3		C14H20O8
C12H24O5S	C36H12O8S3	C16H36N2O10S		C15H22O8
C20H40O5S	C17H36O9	C28H30N2O10S		C16H24O8
C5H10O5S	C18H38O9	C22H14N2O10S		C17H26O8
C6H12O5S	C19H40O9	C17H36N2O11		C18H28O8
C7H14O5S	C20H42O9	C19H40N2O11		C19H30O8
C8H16O5S	C9H20O9	C17H14N2O11		C20H32O8
C9H18O5S	C14H28O9	C25H22N2O11		C21H34O8
C10H18O5S	C15H30O9	C20H10N2O11		C22H36O8
C11H20O5S	C16H32O9	C21H12N2O11		C23H38O8
C13H24O5S	C17H34O9	C34H38N2O11		C24H40O8
C18H34O5S	C18H36O9	C16H36N2O11S		C9H10O8
C19H36O5S	C19H38O9	C17H38N2O11S		C15H20O8
C20H38O5S	C20H40O9	C34H14N2O11S		C17H24O8
C21H40O5S	C21H42O9	C12H14N2O11S3		C18H26O8
C6H10O5S	C22H44O9	C31H32N2O12		C19H28O8

C7H12O5S	C12H24O9	C25H18N2O12	C20H30O8
C8H14O5S	C9H18O9	C28H24N2O12	C21H32O8
C9H16O5S	C13H24O9	C23H16N2O12S	C22H34O8
C10H16O5S	C14H26O9	C22H12N2O12S	C23H36O8
C11H18O5S	C15H28O9	C31H16N2O12S	C24H38O8
C13H22O5S	C16H30O9	C13H24N2O12S2	C10H10O8
C15H26O5S	C17H32O9	C31H12N2O12S2	C11H12O8
C18H32O5S	C18H34O9	C14H12N2O14	C12H14O8
C19H34O5S	C19H36O9	C37H40N2O14	C13H16O8
C20H36O5S	C20H38O9	C25H14N2O14S	C14H18O8
C21H38O5S	C21H40O9	C37H22N2O14S	C16H22O8
C7H10O5S	C23H44O9	C22H44N2O14S2	C16H20O8
C8H12O5S	C25H48O9	C18H22N2O14S2	C17H22O8
C9H14O5S	C15H26O9	C29H12N2O14S2	C18H24O8
C12H18O5S	C16H28O9	C20H16N2O15S	C19H26O8
C13H20O5S	C17H30O9	C23H22N2O15S	C20H28O8
C14H22O5S	C18H32O9	C18H14N2O15S2	C21H30O8
C15H24O5S	C19H34O9	C20H16N2O16	C22H32O8
C8H10O5S	C21H38O9	C28H18N2O16	C23H34O8
C37H66O5S	C22H40O9	C31H24N2O16	C24H36O8
C10H12O5S	C30H56O9	C19H12N2O16S	C25H38O8
C11H14O5S	C31H58O9	C22H18N2O16S	C26H40O8
C12H16O5S	C23H42O9	C17H12N2O16S2	C29H46O8
C13H18O5S	C10H14O9	C15H20N2O17S2	C11H10O8
C14H20O5S	C11H16O9	C17H12N2O18	C12H12O8
C15H22O5S	C12H18O9	C25H16N2O19	C13H14O8
C18H28O5S	C13H20O9	C17H24N2O20	C14H16O8
C9H10O5S	C14H22O9	C24H12N2O20	C15H18O8
C10H10O5S	C15H24O9	C8H12N2O4S	C27H42O8
C11H12O5S	C16H26O9	C42H16N2O4S	C28H44O8
C12H14O5S	C17H28O9	C45H22N2O4S	C17H20O8
C13H16O5S	C18H30O9	C13H14N2O6S	C18H22O8
C14H18O5S	C19H32O9	C27H12N2O6S	C19H24O8
C15H20O5S	C20H34O9	C19H12N2O6S3	C20H26O8
C17H24O5S	C21H36O9	C8H14N2O7S3	C21H28O8
C11H10O5S	C22H38O9	C23H18N2O9S2	C22H30O8
C12H12O5S	C23H40O9	C12H16N2O9S3	C23H32O8
C13H14O5S	C25H44O9	C32H18N2O9S3	C24H34O8
C14H16O5S	C26H46O9	C10H17N3O10S3	C25H36O8
C15H18O5S	C28H50O9	C16H29N3O12S	C12H10O8
C12H10O5S	C29H52O9	C29H17N3O12S	C13H12O8
C13H12O5S	C44H82O9	C11H17N3O13S2	C14H14O8
C14H14O5S	C9H12O9	C34H43N3O13S2	C15H16O8
C16H18O5S	C12H16O9	C31H31N3O14S	C16H18O8

C17H20O5S	C14H20O9	C22H13N3O26S2		C26H38O8
C13H10O5S	C15H22O9	C22H29N3O4S		C27H40O8
C14H12O5S	C16H24O9	C18H37N3O7S3		C19H22O8
C15H14O5S	C17H26O9	C14H13N3O7S3		C20H24O8
C16H16O5S	C18H28O9	C28H15N3O8S3		C23H30O8
C17H18O5S	C19H30O9	C24H15NO11S2		C13H10O8
C18H20O5S	C20H32O9	C12H15NO11S3		C15H14O8
C16H14O5S	C21H34O9	C11H13NO12S2		C16H16O8
C17H16O5S	C22H36O9	C14H21NO14S		C17H18O8
C18H18O5S	C24H40O9	C18H19NO14S2		C18H20O8
C19H20O5S	C25H42O9	C22H13NO19S		C21H26O8
C18H16O5S	C28H48O9	C24H23NO23S2		C22H28O8
C22H24O5S	C42H76O9	C12H23NO9S		C14H10O8
C35H48O5S	C13H18O9	C9H17NO9S		C15H12O8
C20H18O5S	C11H12O9	C10H12O10S2		C16H14O8
C21H20O5S	C12H14O9	C15H14O10S3		C17H16O8
C26H28O5S	C13H16O9	C17H14O10S3		C18H18O8
C34H44O5S	C14H18O9	C17H12O13S		C19H20O8
C37H50O5S	C15H20O9	C20H20O13S3		C20H22O8
C41H58O5S	C16H22O9	C15H28O14		C21H24O8
C20H16O5S	C18H26O9	C29H12O14		C22H26O8
C21H18O5S	C19H28O9	C41H30O14		C23H28O8
C27H30O5S	C20H30O9	C34H12O14		C17H14O8
C28H32O5S	C21H32O9	C28H14O14S		C18H16O8
C31H36O5S	C22H34O9	C14H22O15S2		C19H18O8
C19H12O5S	C23H36O9	C22H12O16S		C20H20O8
C21H16O5S	C24H38O9	C29H16O16S		C21H22O8
C22H18O5S	C25H40O9	C31H14O16S		C22H24O8
C23H20O5S	C26H42O9	C18H22O16S3		C23H26O8
C30H34O5S	C27H44O9	C33H26O16S3		C24H28O8
C29H30O5S	C32H54O9	C22H44O17		C33H46O8
C12H20O5S2	C34H58O9	C29H22O17S		C17H12O8
C14H24O5S2	C35H60O9	C22H12O18		C18H14O8
C8H12O5S2	C42H74O9	C22H10O18		C19H16O8
C15H24O5S2	C17H24O9	C25H16O18		C20H18O8
C10H14O5S2	C18H24O9	C37H24O18		C21H20O8
C11H16O5S2	C19H26O9	C20H18O19		C22H22O8
C12H18O5S2	C20H28O9	C23H24O19		C23H24O8
C8H10O5S2	C21H30O9	C26H18O20		C33H44O8
C9H12O5S2	C22H32O9	C17H14O20S		C20H16O8
C15H22O5S2	C23H34O9	C25H16O23S3		C22H20O8
C11H14O5S2	C24H36O9	C10H20O7S		C24H24O8
C13H18O5S2	C25H38O9	C11H22O7S		C19H14O8
C16H22O5S2	C26H40O9	C13H26O7S		C21H18O8

C13H16O5S2	C13H14O9	C18H36O7S		C22H18O8
C13H12O5S2	C14H16O9	C20H40O7S		C24H22O8
C15H14O5S2	C15H18O9	C6H12O7S		C34H18O8
C15H10O5S2	C16H20O9	C7H14O7S		C42H18O8
C16H12O5S2	C17H22O9	C8H16O7S		C11H22O8S
C17H14O5S2	C18H22O9	C9H18O7S		C12H24O8S
C18H16O5S2	C19H24O9	C10H18O7S		C13H26O8S
C10H22O5S3	C20H26O9	C11H20O7S		C14H28O8S
C11H24O5S3	C21H28O9	C12H22O7S		C19H38O8S
C12H26O5S3	C22H30O9	C13H24O7S		C20H40O8S
C13H28O5S3	C23H32O9	C14H26O7S		C21H42O8S
C14H30O5S3	C24H34O9	C15H28O7S		C7H14O8S
C15H32O5S3	C14H14O9	C16H30O7S		C8H16O8S
C16H34O5S3	C15H16O9	C17H32O7S		C9H18O8S
C18H38O5S3	C16H18O9	C18H34O7S		C10H18O8S
C19H40O5S3	C17H20O9	C20H38O7S		C11H20O8S
C20H42O5S3	C20H24O9	C22H42O7S		C12H22O8S
C22H46O5S3	C21H26O9	C6H10O7S		C13H24O8S
C24H50O5S3	C14H12O9	C7H12O7S		C14H26O8S
C25H52O5S3	C15H14O9	C8H14O7S		C15H28O8S
C23H48O5S3	C16H16O9	C9H16O7S		C16H30O8S
C8H18O5S3	C17H18O9	C10H16O7S		C18H34O8S
C9H20O5S3	C18H20O9	C11H18O7S		C19H36O8S
C11H22O5S3	C19H22O9	C12H20O7S		C20H38O8S
C12H24O5S3	C22H28O9	C13H22O7S		C21H40O8S
C13H26O5S3	C24H32O9	C14H24O7S		C22H42O8S
C14H28O5S3	C16H14O9	C15H26O7S		C24H46O8S
C16H32O5S3	C17H16O9	C18H32O7S		C7H12O8S
C17H34O5S3	C18H18O9	C19H34O7S		C8H14O8S
C18H36O5S3	C19H20O9	C20H36O7S		C9H16O8S
C20H40O5S3	C20H22O9	C22H40O7S		C10H16O8S
C21H42O5S3	C21H24O9	C7H10O7S		C11H18O8S
C23H46O5S3	C22H26O9	C8H12O7S		C12H20O8S
C24H48O5S3	C24H30O9	C9H14O7S		C13H22O8S
C25H50O5S3	C16H12O9	C10H14O7S		C14H24O8S
C26H52O5S3	C17H14O9	C11H16O7S		C15H26O8S
C10H20O5S3	C18H16O9	C12H18O7S		C16H28O8S
C22H44O5S3	C19H18O9	C13H20O7S		C17H30O8S
C8H16O5S3	C20H20O9	C14H22O7S		C18H32O8S
C9H18O5S3	C21H22O9	C15H24O7S		C20H36O8S
C11H20O5S3	C22H24O9	C18H30O7S		C21H38O8S
C12H22O5S3	C23H26O9	C20H34O7S		C22H40O8S
C13H24O5S3	C24H28O9	C8H10O7S		C23H42O8S
C16H30O5S3	C16H10O9	C9H12O7S		C24H44O8S

C18H34O5S3	C17H12O9	C11H14O7S	C8H12O8S
C21H40O5S3	C18H14O9	C12H16O7S	C9H14O8S
C23H44O5S3	C19H16O9	C13H18O7S	C10H14O8S
C14H26O5S3	C20H18O9	C14H20O7S	C11H16O8S
C9H16O5S3	C21H20O9	C15H22O7S	C12H18O8S
C12H20O5S3	C22H22O9	C16H24O7S	C13H20O8S
C13H22O5S3	C23H24O9	C24H40O7S	C14H22O8S
C14H24O5S3	C24H26O9	C26H44O7S	C15H24O8S
C15H26O5S3	C20H16O9	C9H10O7S	C16H26O8S
C19H34O5S3	C21H18O9	C11H12O7S	C17H28O8S
C20H34O5S3	C22H20O9	C12H14O7S	C18H30O8S
C16H24O5S3	C23H22O9	C13H16O7S	C20H34O8S
C19H20O5S3	C24H24O9	C14H18O7S	C22H38O8S
C19H18O5S3	C25H26O9	C15H20O7S	C8H10O8S
C21H22O5S3	C21H16O9	C16H22O7S	C9H12O8S
C22H24O5S3	C23H20O9	C17H24O7S	C10H12O8S
C23H26O5S3	C24H22O9	C18H26O7S	C11H14O8S
C24H28O5S3	C25H22O9	C11H10O7S	C12H16O8S
C25H30O5S3	C31H12O9	C13H14O7S	C13H18O8S
C26H32O5S3	C42H12O9	C14H16O7S	C14H20O8S
C18H16O5S3	C42H10O9	C15H18O7S	C15H22O8S
C20H20O5S3	C11H24O9S3	C16H20O7S	C16H24O8S
C18H14O5S3	C12H26O9S3	C17H22O7S	C17H26O8S
C19H16O5S3	C14H30O9S3	C14H14O7S	C18H28O8S
C20H18O5S3	C16H28O9S3	C15H16O7S	C20H32O8S
C21H20O5S3	C25H28O9S3	C16H18O7S	C25H42O8S
C22H22O5S3	C14H30OS	C17H20O7S	C11H12O8S
C25H28O5S3	C14H28OS	C15H14O7S	C12H14O8S
C31H40O5S3	C12H22OS	C16H16O7S	C13H16O8S
C20H16O5S3	C15H24OS	C17H18O7S	C14H18O8S
C21H18O5S3	C23H40OS	C18H20O7S	C15H20O8S
C30H36O5S3	C9H12OS	C19H22O7S	C16H22O8S
C31H38O5S3	C15H22OS	C16H14O7S	C17H24O8S
C32H40O5S3	C17H26OS	C17H16O7S	C18H26O8S
C33H42O5S3	C17H24OS	C19H20O7S	C13H14O8S
C34H44O5S3	C18H26OS	C29H30O7S	C14H16O8S
C27H28O5S3	C17H22OS	C12H18O7S2	C15H18O8S
C33H40O5S3	C18H24OS	C18H18O7S2	C16H20O8S
C25H22O5S3	C18H22OS	C18H14O7S2	C17H22O8S
C11H24O6	C12H10OS	C12H18O8S2	C18H24O8S
C12H26O6	C14H14OS	C16H22O8S2	C19H26O8S
C13H28O6	C15H16OS	C47H30O8S2	C15H16O8S
C14H30O6	C16H18OS	C18H36O9S	C16H18O8S
C15H32O6	C17H20OS	C12H22O9S	C17H20O8S

C9H20O6	C25H36OS	C18H34O9S	C18H22O8S
C11H22O6	C16H16OS	C20H38O9S	C20H26O8S
C13H26O6	C17H18OS	C22H42O9S	C17H18O8S
C15H30O6	C14H12OS	C12H20O9S	C18H20O8S
C16H32O6	C15H14OS	C13H22O9S	C16H34O8S3
C17H34O6	C27H38OS	C14H24O9S	C17H36O8S3
C18H36O6	C14H10OS	C18H32O9S	C10H22O8S3
C19H38O6	C15H12OS	C20H36O9S	C11H24O8S3
C20H40O6	C27H36OS	C22H40O9S	C12H26O8S3
C22H44O6	C21H20OS	C12H18O9S	C13H28O8S3
C10H20O6	C23H20OS	C13H20O9S	C14H30O8S3
C8H16O6	C28H30OS	C14H22O9S	C23H48O8S3
C9H18O6	C20H12OS	C15H24O9S	C25H52O8S3
C17H32O6	C22H16OS	C9H12O9S	C26H54O8S3
C19H36O6	C50H72OS	C10H12O9S	C27H56O8S3
C20H38O6	C10H18OS2	C12H16O9S	C17H34O8S3
C21H40O6	C18H30OS2	C13H18O9S	C18H36O8S3
C23H44O6	C19H32OS2	C14H20O9S	C10H20O8S3
C25H48O6	C17H24OS2	C15H22O9S	C11H22O8S3
C26H50O6	C12H12OS2	C12H14O9S	C12H24O8S3
C6H10O6	C13H14OS2	C13H16O9S	C13H26O8S3
C7H12O6	C15H16OS2	C14H18O9S	C14H28O8S3
C9H16O6	C17H18OS2	C15H20O9S	C15H30O8S3
C10H18O6	C18H20OS2	C16H22O9S	C23H46O8S3
C11H20O6	C19H22OS2	C14H16O9S	C25H50O8S3
C12H22O6	C20H24OS2	C15H18O9S	C27H54O8S3
C14H26O6	C25H34OS2	C16H20O9S	C28H56O8S3
C15H28O6	C27H38OS2	C17H22O9S	C12H22O8S3
C16H30O6	C28H40OS2	C18H24O9S	C13H24O8S3
C18H34O6	C29H42OS2	C20H28O9S	C14H26O8S3
C22H42O6	C18H18OS2	C15H16O9S	C22H24O8S3
C24H46O6	C19H20OS2	C16H18O9S	C27H34O8S3
C8H14O6	C20H22OS2	C17H20O9S	C19H16O8S3
C10H16O6	C21H24OS2	C18H22O9S	C18H12O8S3
C11H18O6	C26H34OS2	C18H16O9S	C17H36O9
C22H40O6	C18H16OS2	C23H12O9S	C18H38O9
C23H42O6	C20H20OS2	C40H40O9S	C19H40O9
C24H44O6	C21H22OS2	C31H44O9S2	C20H42O9
C25H46O6	C16H12OS2		C21H44O9
C26H48O6	C20H18OS2		C9H20O9
C28H52O6	C22H22OS2		C11H22O9
C8H12O6	C24H26OS2		C12H24O9
C12H20O6	C18H14OS2		C13H26O9
C13H22O6	C21H20OS2		C14H28O9

C17H30O6	C18H10OS2	C15H30O9
C18H32O6	C34H42OS2	C16H32O9
C27H50O6	C24H18OS2	C17H34O9
C7H10O6	C36H42OS2	C18H36O9
C9H14O6	C38H46OS2	C19H38O9
C10H14O6	C35H26OS2	C20H40O9
C11H16O6	C36H28OS2	C21H42O9
C12H18O6	C35H24OS2	C22H44O9
C16H26O6	C32H14OS2	C10H20O9
C24H42O6	C17H14OS3	C9H18O9
C25H44O6	C19H18OS3	C13H24O9
C26H46O6	C21H14OS3	C14H26O9
C27H48O6	C25H16OS3	C15H28O9
C28H50O6	C33H18OS3	C16H30O9
C13H20O6	C35H22OS3	C17H32O9
C14H22O6	C37H26OS3	C18H34O9
C15H24O6	C36H24OS3	C19H36O9
C17H28O6	C45H40OS3	C20H38O9
C18H30O6	C39H22OS3	C21H40O9
C21H36O6	C18H12N2O10S	C23H44O9
C22H38O6	C22H14N2O10S	C14H24O9
C23H40O6	C30H12N2O10S	C15H26O9
C29H52O6	C17H36N2O11	C16H28O9
C30H54O6	C17H14N2O11	C17H30O9
C8H10O6	C34H38N2O11	C18H32O9
C9H12O6	C22H12N2O11S	C19H34O9
C10H12O6	C25H18N2O12	C21H38O9
C11H14O6	C28H24N2O12	C22H40O9
C13H18O6	C17H36N2O12S	C26H48O9
C14H20O6	C27H48N2O12S	C29H54O9
C16H24O6	C23H16N2O12S	C31H58O9
C17H26O6	C31H16N2O12S	C42H80O9
C18H28O6	C13H24N2O12S2	C11H18O9
C19H30O6	C31H12N2O12S2	C12H20O9
C22H36O6	C12H12N2O12S3	C20H36O9
C23H38O6	C38H16N2O12S3	C23H42O9
C24H40O6	C29H20N2O13S2	C10H14O9
C25H42O6	C14H12N2O14	C11H16O9
C26H44O6	C17H18N2O14	C12H18O9
C28H48O6	C24H14N2O14	C13H20O9
C29H50O6	C37H40N2O14	C14H22O9
C12H16O6	C26H12N2O14	C15H24O9
C15H22O6	C25H14N2O14S	C16H26O9
C20H32O6	C18H22N2O14S2	C17H28O9

C21H34O6	C29H26N2O14S2	C18H30O9
C27H46O6	C35H20N2O14S2	C19H32O9
C9H10O6	C24H18N2O14S3	C20H34O9
C11H12O6	C26H16N2O14S3	C21H36O9
C13H16O6	C36H34N2O15	C22H38O9
C14H18O6	C18H22N2O15S	C23H40O9
C15H20O6	C20H16N2O15S	C25H44O9
C16H22O6	C23H22N2O15S	C27H48O9
C17H24O6	C16H20N2O15S2	C29H52O9
C18H26O6	C19H22N2O15S2	C24H42O9
C19H28O6	C18H14N2O15S2	C16H24O9
C20H30O6	C31H28N2O15S2	C17H26O9
C21H32O6	C15H18N2O15S3	C18H28O9
C22H34O6	C20H16N2O16	C19H30O9
C23H36O6	C28H18N2O16	C22H36O9
C24H38O6	C31H24N2O16	C26H44O9
C25H40O6	C19H12N2O16S	C27H46O9
C10H10O6	C22H18N2O16S	C28H48O9
C12H14O6	C17H12N2O16S2	C29H50O9
C45H80O6	C20H18N2O16S2	C30H52O9
C12H12O6	C15H20N2O17S2	C40H72O9
C13H14O6	C17H12N2O18	C12H16O9
C14H16O6	C21H44N2O18S	C13H18O9
C15H18O6	C23H22N2O18S	C14H20O9
C16H20O6	C26H28N2O18S	C15H22O9
C17H22O6	C16H22N2O18S2	C20H32O9
C18H24O6	C21H22N2O18S2	C21H34O9
C19H26O6	C25H16N2O19	C23H38O9
C20H28O6	C32H32N2O19S	C11H12O9
C21H30O6	C17H24N2O20	C13H16O9
C22H32O6	C24H12N2O20	C14H18O9
C23H34O6	C15H18N2O6S3	C18H26O9
C24H36O6	C19H12N2O6S3	C19H28O9
C25H38O6	C8H14N2O7S3	C20H30O9
C11H10O6	C13H14N2O8S2	C21H32O9
C14H14O6	C28H52N2O9S2	C22H34O9
C15H16O6	C19H14N2O9S2	C23H36O9
C16H18O6	C23H18N2O9S2	C24H38O9
C17H20O6	C12H16N2O9S3	C26H42O9
C18H22O6	C32H18N2O9S3	C12H14O9
C19H24O6	C11H17N3O13S2	C15H20O9
C20H26O6	C11H13N3O13S2	C16H22O9
C25H36O6	C14H19N3O13S2	C17H24O9
C12H10O6	C24H25N3O15S	C19H26O9

C13H12O6	C20H19N3O17S3	C20H28O9
C22H30O6	C18H23N3O19S	C21H30O9
C23H32O6	C25H23N3O19S	C22H32O9
C16H16O6	C22H17N3O20S	C23H34O9
C17H18O6	C10H13N3O4S3	C24H36O9
C18H20O6	C19H19N3O4S3	C26H40O9
C19H22O6	C21H23N3O4S3	C14H16O9
C20H24O6	C20H13N3O4S3	C15H18O9
C21H26O6	C15H17N3O6S3	C16H20O9
C22H28O6	C18H23N3O6S3	C17H22O9
C23H30O6	C14H13N3O7S3	C18H24O9
C13H10O6	C16H33N3O9S	C19H24O9
C14H12O6	C28H49NO12S	C20H26O9
C15H14O6	C36H13NO12S2	C21H28O9
C16H14O6	C42H17NO13S	C22H30O9
C17H16O6	C34H25NO17S	C23H32O9
C18H18O6	C19H13NO21	C24H34O9
C20H22O6	C47H75NO7S	C14H14O9
C25H32O6	C18H15NO8S2	C15H16O9
C14H10O6	C27H21NO8S2	C16H18O9
C15H12O6	C27H17NO8S2	C17H20O9
C19H20O6	C29H52O10S2	C18H22O9
C21H24O6	C10H12O10S2	C25H36O9
C22H26O6	C19H22O11S3	C26H38O9
C15H10O6	C24H48O12S	C24H32O9
C17H14O6	C14H24O12S	C14H12O9
C18H16O6	C15H18O12S	C15H14O9
C19H18O6	C23H20O13S	C16H16O9
C20H20O6	C20H20O13S3	C17H18O9
C21H22O6	C15H28O14	C18H20O9
C22H24O6	C25H14O14	C19H22O9
C26H30O6	C29H12O14	C20H24O9
C16H10O6	C34H12O14	C21H26O9
C17H12O6	C45H16O14	C22H28O9
C18H14O6	C20H22O14S	C15H12O9
C19H16O6	C24H18O14S	C16H14O9
C20H18O6	C22H14O14S2	C17H16O9
C21H20O6	C13H24O14S3	C18H18O9
C22H22O6	C27H22O14S3	C19H20O9
C20H16O6	C27H14O14S3	C20H22O9
C21H18O6	C15H18O15S	C21H24O9
C22H20O6	C14H22O15S2	C22H26O9
C24H24O6	C22H22O15S2	C24H30O9
C25H26O6	C23H12O15S2	C16H12O9

C26H28O6	C37H32O15S2	C17H14O9
C27H30O6	C21H18O16S	C18H16O9
C19H14O6	C29H16O16S	C19H18O9
C21H16O6	C31H14O16S	C20H20O9
C23H20O6	C15H26O16S2	C21H22O9
C24H22O6	C29H12O16S2	C22H24O9
C25H24O6	C16H20O16S3	C23H26O9
C26H26O6	C29H20O17	C24H28O9
C27H28O6	C30H16O17	C18H14O9
C28H30O6	C29H22O17S	C19H16O9
C29H32O6	C24H24O17S3	C20H18O9
C31H36O6	C25H16O18	C21H20O9
C20H14O6	C37H24O18	C22H22O9
C33H40O6	C22H30O18S2	C23H24O9
C20H12O6	C32H44O18S2	C24H26O9
C22H16O6	C26H18O18S2	C20H16O9
C23H18O6	C18H16O19S	C21H18O9
C24H20O6	C32H24O19S	C22H20O9
C26H24O6	C28H12O19S	C23H22O9
C27H26O6	C26H12O19S2	C24H24O9
C28H28O6	C19H14O20	C23H20O9
C29H30O6	C26H18O20	C24H22O9
C30H32O6	C17H14O20S	C31H12O9
C31H34O6	C20H20O20S	C42H12O9
C32H36O6	C20H14O24S	C22H46O9S
C33H38O6	C18H38O7S	C22H44O9S
C34H40O6	C10H20O7S	C15H30O9S
C25H22O6	C11H22O7S	C18H36O9S
C21H12O6	C12H24O7S	C20H40O9S
C22H14O6	C18H36O7S	C8H16O9S
C23H16O6	C20H40O7S	C23H44O9S
C24H18O6	C6H12O7S	C11H20O9S
C25H20O6	C7H14O7S	C12H22O9S
C26H22O6	C8H16O7S	C13H24O9S
C28H26O6	C9H18O7S	C14H26O9S
C29H28O6	C10H18O7S	C18H34O9S
C30H30O6	C11H20O7S	C19H36O9S
C31H32O6	C12H22O7S	C20H38O9S
C32H34O6	C14H26O7S	C22H42O9S
C27H24O6	C16H30O7S	C24H46O9S
C33H36O6	C18H34O7S	C11H18O9S
C34H38O6	C20H38O7S	C12H20O9S
C36H42O6	C21H40O7S	C13H22O9S
C22H12O6	C22H42O7S	C14H24O9S

C23H14O6	C6H10O7S	C15H26O9S
C24H16O6	C7H12O7S	C16H28O9S
C25H18O6	C8H14O7S	C18H32O9S
C26H20O6	C9H16O7S	C19H34O9S
C27H22O6	C10H16O7S	C20H36O9S
C28H24O6	C11H18O7S	C21H38O9S
C29H26O6	C12H20O7S	C22H40O9S
C30H28O6	C13H22O7S	C23H42O9S
C31H30O6	C14H24O7S	C11H16O9S
C32H32O6	C15H26O7S	C12H18O9S
C34H36O6	C18H32O7S	C13H20O9S
C33H34O6	C20H36O7S	C14H22O9S
C24H14O6	C22H40O7S	C15H24O9S
C25H16O6	C23H42O7S	C16H26O9S
C26H18O6	C7H10O7S	C18H30O9S
C27H20O6	C8H12O7S	C12H16O9S
C28H22O6	C9H14O7S	C13H18O9S
C29H24O6	C10H14O7S	C14H20O9S
C30H26O6	C11H16O7S	C15H22O9S
C32H30O6	C12H18O7S	C16H24O9S
C38H42O6	C13H20O7S	C12H14O9S
C40H46O6	C14H22O7S	C13H16O9S
C25H14O6	C15H24O7S	C14H18O9S
C26H16O6	C18H30O7S	C15H20O9S
C27H18O6	C20H34O7S	C16H22O9S
C28H20O6	C8H10O7S	C18H26O9S
C27H14O6	C9H12O7S	C13H14O9S
C28H16O6	C11H14O7S	C14H16O9S
C33H20O6	C12H16O7S	C15H18O9S
C32H16O6	C13H18O7S	C16H20O9S
C34H16O6	C14H20O7S	C17H22O9S
C45H38O6	C15H22O7S	C18H24O9S
C36H14O6	C17H26O7S	C16H18O9S
C9H20O6S	C26H44O7S	C17H20O9S
C10H20O6S	C9H10O7S	C18H22O9S
C11H22O6S	C11H12O7S	C19H24O9S
C12H24O6S	C12H14O7S	C20H26O9S
C14H28O6S	C13H16O7S	C18H20O9S
C15H30O6S	C14H18O7S	C21H26O9S
C16H32O6S	C15H20O7S	C31H46O9S
C17H34O6S	C16H22O7S	C19H20O9S
C18H36O6S	C17H24O7S	C20H22O9S
C19H38O6S	C18H26O7S	C18H16O9S
C20H40O6S	C14H16O7S	C23H12O9S

C22H44O6S	C15H18O7S	C12H22OS
C6H12O6S	C16H20O7S	C13H22OS
C7H14O6S	C17H22O7S	C15H26OS
C8H16O6S	C18H24O7S	C15H24OS
C9H18O6S	C14H14O7S	C23H40OS
C10H18O6S	C15H16O7S	C25H44OS
C11H20O6S	C16H18O7S	C27H48OS
C12H22O6S	C17H20O7S	C15H22OS
C13H24O6S	C18H22O7S	C17H26OS
C14H26O6S	C15H14O7S	C18H28OS
C15H28O6S	C16H16O7S	C25H42OS
C16H30O6S	C17H18O7S	C17H24OS
C18H34O6S	C18H20O7S	C18H26OS
C19H36O6S	C16H14O7S	C18H24OS
C20H38O6S	C17H16O7S	C18H22OS
C21H40O6S	C18H18O7S	C19H24OS
C22H42O6S	C19H20O7S	C12H10OS
C6H10O6S	C12H18O7S2	C14H14OS
C7H12O6S	C13H16O7S2	C15H16OS
C8H14O6S	C14H18O7S2	C19H22OS
C9H16O6S	C33H52O7S2	C14H12OS
C14H24O6S	C18H18O7S2	C15H14OS
C10H16O6S	C18H14O7S2	C18H20OS
C11H18O6S	C32H16O7S2	C27H38OS
C12H20O6S	C11H16O8S2	C14H10OS
C13H22O6S	C16H22O8S2	C29H40OS
C15H26O6S	C18H36O9S	C24H28OS
C18H32O6S	C14H26O9S	C26H32OS
C20H36O6S	C18H34O9S	C28H36OS
C22H40O6S	C20H38O9S	C32H42OS
C7H10O6S	C22H42O9S	C21H20OS
C8H12O6S	C11H18O9S	C28H30OS
C9H14O6S	C15H26O9S	C29H32OS
C11H16O6S	C18H32O9S	C31H36OS
C12H18O6S	C20H36O9S	C20H12OS
C13H20O6S	C22H40O9S	C32H24OS
C14H22O6S	C10H12O9S	C38H34OS
C15H24O6S	C12H16O9S	C10H18OS2
C16H26O6S	C13H18O9S	C12H12OS2
C17H28O6S	C14H20O9S	C13H14OS2
C18H30O6S	C15H22O9S	C22H32OS2
C23H40O6S	C12H14O9S	C15H16OS2
C8H10O6S	C13H16O9S	C17H20OS2
C9H12O6S	C14H18O9S	C24H34OS2

C10H12O6S	C15H20O9S	C16H16OS2
C11H14O6S	C16H22O9S	C17H18OS2
C12H16O6S	C14H16O9S	C19H22OS2
C13H18O6S	C15H18O9S	C20H24OS2
C14H20O6S	C16H20O9S	C22H28OS2
C15H22O6S	C17H22O9S	C25H34OS2
C16H24O6S	C18H24O9S	C27H38OS2
C17H26O6S	C14H14O9S	C29H42OS2
C9H10O6S	C15H16O9S	C24H32OS2
C10H10O6S	C16H18O9S	C24H30OS2
C11H12O6S	C17H20O9S	C26H34OS2
C12H14O6S	C18H22O9S	C18H16OS2
C13H16O6S	C20H24O9S	C20H20OS2
C14H18O6S	C20H22O9S	C16H12OS2
C15H20O6S	C23H12O9S	C20H18OS2
C16H22O6S	C40H40O9S	C22H22OS2
C17H24O6S	C31H46O9S2	C24H26OS2
C12H12O6S		C18H14OS2
C29H46O6S		C21H20OS2
C30H48O6S		C37H46OS2
C31H50O6S		C24H18OS2
C11H10O6S		C36H42OS2
C13H14O6S		C38H46OS2
C14H16O6S		C32H16OS2
C15H18O6S		C31H12OS2
C16H20O6S		C44H88OS3
C17H22O6S		C42H82OS3
C18H24O6S		C32H60OS3
C12H10O6S		C33H62OS3
C13H12O6S		C9H14OS3
C14H14O6S		C17H14OS3
C15H16O6S		C19H18OS3
C16H18O6S		C32H40OS3
C17H20O6S		C25H16OS3
C18H22O6S		C33H18OS3
C13H10O6S		C36H24OS3
C15H14O6S		C33H16OS3
C18H20O6S		C16H36N2O10S
C15H12O6S		C22H14N2O10S
C16H14O6S		C32H20N2O10S2
C17H16O6S		C17H14N2O11
C18H16O6S		C20H20N2O11
C19H18O6S		C15H32N2O11S
C28H34O6S		C18H34N2O11S

C32H42O6S	C25H18N2O12
C28H32O6S	C28H24N2O12
C29H34O6S	C23H16N2O12S
C18H12O6S	C31H16N2O12S
C19H14O6S	C14H12N2O12S2
C28H30O6S	C31H12N2O12S2
C33H40O6S	C17H36N2O13S
C18H10O6S	C29H20N2O13S2
C20H14O6S	C14H12N2O14
C9H14O6S2	C17H18N2O14
C13H20O6S2	C25H32N2O14
C10H14O6S2	C27H36N2O14
C12H18O6S2	C26H12N2O14
C10H12O6S2	C25H14N2O14S
C12H16O6S2	C26H16N2O14S3
C14H20O6S2	C15H12N2O15
C14H18O6S2	C20H16N2O15S
C14H16O6S2	C23H22N2O15S
C14H14O6S2	C38H30N2O15S
C19H18O6S2	C18H14N2O15S2
C16H10O6S2	C15H18N2O15S3
C17H12O6S2	C17H14N2O16
C18H14O6S2	C20H16N2O16
C30H36O6S2	C28H18N2O16
C23H16O6S2	C31H24N2O16
C23H12O6S2	C19H12N2O16S
C10H22O6S3	C21H16N2O16S
C12H26O6S3	C17H12N2O16S2
C14H30O6S3	C23H22N2O18S
C15H32O6S3	C25H16N2O19
C16H34O6S3	C18H18N2O19S3
C17H36O6S3	C17H24N2O20
C19H40O6S3	C24H12N2O20
C20H42O6S3	C34H16N2O6S
C25H52O6S3	C19H12N2O6S3
C11H24O6S3	C8H14N2O7S3
C13H28O6S3	C15H12N2O8S3
C23H48O6S3	C19H14N2O9S2
C9H20O6S3	C23H18N2O9S2
C11H22O6S3	C22H40N2O9S3
C13H26O6S3	C12H16N2O9S3
C14H28O6S3	C24H16N2O9S3
C15H30O6S3	C15H29N3O11S2
C17H34O6S3	C21H41N3O11S2

C18H36O6S3	C30H55N3O11S2
C19H38O6S3	C25H15N3O16S2
C21H42O6S3	C23H15N3O18S3
C24H48O6S3	C18H23N3O19S
C10H20O6S3	C19H19N3O4S3
C12H24O6S3	C10H19N3O5S3
C23H46O6S3	C7H13N3O5S3
C9H18O6S3	C21H37N3O5S3
C10H18O6S3	C14H19N3O6S3
C12H22O6S3	C15H17N3O6S3
C13H24O6S3	C18H37N3O7S3
C15H28O6S3	C21H41NO10S2
C16H30O6S3	C12H15NO11S3
C17H32O6S3	C14H19NO11S3
C19H36O6S3	C27H17NO11S3
C24H46O6S3	C21H17NO12S3
C25H48O6S3	C21H21NO17S2
C28H54O6S3	C23H21NO19
C11H20O6S3	C9H17NO6S
C14H26O6S3	C22H41NO6S
C20H38O6S3	C20H21NO6S
C22H42O6S3	C31H21NO6S
C15H26O6S3	C10H21NO6S3
C20H36O6S3	C12H25NO6S3
C21H38O6S3	C33H21NO6S3
C23H42O6S3	C13H25NO7S
C24H44O6S3	C12H23NO9S
C30H56O6S3	C9H17NO9S
C13H22O6S3	C18H15NO9S
C14H24O6S3	C20H39NO9S2
C17H30O6S3	C28H29NO9S2
C18H30O6S3	C10H12O10S2
C20H34O6S3	C13H18O10S2
C14H22O6S3	C23H46O10S3
C23H30O6S3	C18H34O10S3
C24H32O6S3	C21H42O12S2
C22H24O6S3	C22H46O12S3
C23H26O6S3	C17H12O13S
C24H28O6S3	C23H20O13S
C25H30O6S3	C20H20O13S3
C19H18O6S3	C20H22O14S
C22H22O6S3	C22H14O14S2
C23H24O6S3	C14H22O15S2
C24H26O6S3	C18H32O16

C25H28O6S3	C29H16O16S
C26H30O6S3	C31H14O16S
C27H32O6S3	C25H16O18
C18H14O6S3	C37H24O18
C19H16O6S3	C21H38O19
C20H18O6S3	C20H18O19
C21H20O6S3	C28H12O19S
C20H16O6S3	C19H14O20
C21H18O6S3	C26H18O20
C22H20O6S3	C17H14O20S
C23H22O6S3	C18H14O7S2
C24H24O6S3	C30H38O7S2
C25H26O6S3	C11H16O8S2
C29H34O6S3	C12H18O8S2
C19H12O6S3	C24H40O8S2
C23H20O6S3	C15H20O8S2
C25H24O6S3	C16H22O8S2
C11H24O7	C16H20O8S2
C12H26O7	C15H16O8S2
C13H28O7	C16H22O9S2
C14H30O7	C40H26O9S2
C15H32O7	C11H24O9S3
C16H34O7	C12H26O9S3
C17H36O7	C14H30O9S3
C10H20O7	C12H24O9S3
C11H22O7	C14H26O9S3
C12H24O7	C17H30O9S3
C13H26O7	C21H36O9S3
C14H28O7	C27H42O9S3
C15H30O7	
C16H32O7	
C17H34O7	
C18H36O7	
C19H38O7	
C20H40O7	
C10H18O7	
C11H20O7	
C12H22O7	
C13H24O7	
C15H28O7	
C16H30O7	
C17H32O7	
C20H38O7	
C21H40O7	

$C_{22}H_{42}O_7$
$C_{23}H_{44}O_7$
$C_{24}H_{46}O_7$
$C_{25}H_{48}O_7$
$C_{26}H_{50}O_7$
$C_9H_{16}O_7$
$C_{14}H_{26}O_7$
$C_{18}H_{34}O_7$
$C_{19}H_{36}O_7$
$C_7H_{12}O_7$
$C_8H_{14}O_7$
$C_{10}H_{16}O_7$
$C_{11}H_{18}O_7$
$C_{13}H_{22}O_7$
$C_{14}H_{24}O_7$
$C_{15}H_{26}O_7$
$C_{22}H_{40}O_7$
$C_{23}H_{42}O_7$
$C_{24}H_{44}O_7$
$C_{25}H_{46}O_7$
$C_{26}H_{48}O_7$
$C_{27}H_{50}O_7$
$C_9H_{14}O_7$
$C_{12}H_{20}O_7$
$C_{16}H_{28}O_7$
$C_{17}H_{30}O_7$
$C_{18}H_{32}O_7$
$C_{19}H_{34}O_7$
$C_{20}H_{36}O_7$
$C_{21}H_{38}O_7$
$C_{28}H_{52}O_7$
$C_7H_{10}O_7$
$C_8H_{12}O_7$
$C_{10}H_{14}O_7$
$C_{11}H_{16}O_7$
$C_{12}H_{18}O_7$
$C_{14}H_{22}O_7$
$C_{15}H_{24}O_7$
$C_{17}H_{28}O_7$
$C_{18}H_{30}O_7$
$C_{23}H_{40}O_7$
$C_{24}H_{42}O_7$
$C_{25}H_{44}O_7$
$C_{26}H_{46}O_7$

$C_{27}H_{48}O_7$
$C_{28}H_{50}O_7$
$C_{29}H_{52}O_7$
$C_{13}H_{20}O_7$
$C_{16}H_{26}O_7$
$C_{20}H_{34}O_7$
$C_{21}H_{36}O_7$
$C_{22}H_{38}O_7$
$C_8H_{10}O_7$
$C_9H_{12}O_7$
$C_{11}H_{14}O_7$
$C_{12}H_{16}O_7$
$C_{13}H_{18}O_7$
$C_{14}H_{20}O_7$
$C_{15}H_{22}O_7$
$C_{16}H_{24}O_7$
$C_{17}H_{26}O_7$
$C_{18}H_{28}O_7$
$C_{19}H_{30}O_7$
$C_{20}H_{32}O_7$
$C_{25}H_{42}O_7$
$C_{26}H_{44}O_7$
$C_{27}H_{46}O_7$
$C_{29}H_{50}O_7$
$C_{30}H_{52}O_7$
$C_{31}H_{54}O_7$
$C_{10}H_{12}O_7$
$C_{21}H_{34}O_7$
$C_{22}H_{36}O_7$
$C_{23}H_{38}O_7$
$C_{24}H_{40}O_7$
$C_{28}H_{48}O_7$
$C_9H_{10}O_7$
$C_{13}H_{16}O_7$
$C_{15}H_{20}O_7$
$C_{16}H_{22}O_7$
$C_{17}H_{24}O_7$
$C_{18}H_{26}O_7$
$C_{19}H_{28}O_7$
$C_{20}H_{30}O_7$
$C_{21}H_{32}O_7$
$C_{22}H_{34}O_7$
$C_{23}H_{36}O_7$
$C_{24}H_{38}O_7$

$C_{25}H_{40}O_7$
$C_{26}H_{42}O_7$
$C_{27}H_{44}O_7$
$C_{28}H_{46}O_7$
$C_{29}H_{48}O_7$
$C_{30}H_{50}O_7$
$C_{32}H_{54}O_7$
$C_{10}H_{10}O_7$
$C_{11}H_{12}O_7$
$C_{12}H_{14}O_7$
$C_{14}H_{18}O_7$
$C_{13}H_{14}O_7$
$C_{14}H_{16}O_7$
$C_{15}H_{18}O_7$
$C_{16}H_{20}O_7$
$C_{17}H_{22}O_7$
$C_{18}H_{24}O_7$
$C_{19}H_{26}O_7$
$C_{20}H_{28}O_7$
$C_{21}H_{30}O_7$
$C_{22}H_{32}O_7$
$C_{23}H_{34}O_7$
$C_{24}H_{36}O_7$
$C_{27}H_{42}O_7$
$C_{11}H_{10}O_7$
$C_{12}H_{12}O_7$
$C_{26}H_{40}O_7$
$C_{15}H_{16}O_7$
$C_{16}H_{18}O_7$
$C_{17}H_{20}O_7$
$C_{18}H_{22}O_7$
$C_{19}H_{24}O_7$
$C_{20}H_{26}O_7$
$C_{21}H_{28}O_7$
$C_{22}H_{30}O_7$
$C_{23}H_{32}O_7$
$C_{24}H_{34}O_7$
$C_{12}H_{10}O_7$
$C_{13}H_{12}O_7$
$C_{14}H_{14}O_7$
$C_{26}H_{38}O_7$
$C_{15}H_{14}O_7$
$C_{16}H_{16}O_7$
$C_{17}H_{18}O_7$

$C_{18}H_{20}O_7$
$C_{19}H_{22}O_7$
$C_{21}H_{26}O_7$
$C_{22}H_{28}O_7$
$C_{13}H_{10}O_7$
$C_{14}H_{12}O_7$
$C_{20}H_{24}O_7$
$C_{23}H_{30}O_7$
$C_{24}H_{32}O_7$
$C_{25}H_{34}O_7$
$C_{18}H_{18}O_7$
$C_{19}H_{20}O_7$
$C_{20}H_{22}O_7$
$C_{21}H_{24}O_7$
$C_{14}H_{10}O_7$
$C_{15}H_{12}O_7$
$C_{16}H_{14}O_7$
$C_{17}H_{16}O_7$
$C_{22}H_{26}O_7$
$C_{23}H_{28}O_7$
$C_{25}H_{32}O_7$
$C_{21}H_{22}O_7$
$C_{15}H_{10}O_7$
$C_{16}H_{12}O_7$
$C_{17}H_{14}O_7$
$C_{18}H_{16}O_7$
$C_{19}H_{18}O_7$
$C_{20}H_{20}O_7$
$C_{22}H_{24}O_7$
$C_{23}H_{26}O_7$
$C_{21}H_{20}O_7$
$C_{16}H_{10}O_7$
$C_{17}H_{12}O_7$
$C_{18}H_{14}O_7$
$C_{19}H_{16}O_7$
$C_{20}H_{18}O_7$
$C_{22}H_{22}O_7$
$C_{23}H_{24}O_7$
$C_{26}H_{28}O_7$
$C_{18}H_{12}O_7$
$C_{19}H_{14}O_7$
$C_{20}H_{16}O_7$
$C_{21}H_{18}O_7$
$C_{22}H_{20}O_7$

$C_{23}H_{22}O_7$
$C_{22}H_{18}O_7$
$C_{24}H_{22}O_7$
$C_{25}H_{24}O_7$
$C_{26}H_{26}O_7$
$C_{21}H_{16}O_7$
$C_{23}H_{20}O_7$
$C_{21}H_{14}O_7$
$C_{22}H_{16}O_7$
$C_{23}H_{18}O_7$
$C_{24}H_{20}O_7$
$C_{25}H_{22}O_7$
$C_{26}H_{24}O_7$
$C_{27}H_{26}O_7$
$C_{28}H_{28}O_7$
$C_{29}H_{30}O_7$
$C_{30}H_{32}O_7$
$C_{31}H_{34}O_7$
$C_{32}H_{36}O_7$
$C_{34}H_{40}O_7$
$C_{20}H_{12}O_7$
$C_{33}H_{38}O_7$
$C_{23}H_{16}O_7$
$C_{24}H_{18}O_7$
$C_{25}H_{20}O_7$
$C_{26}H_{22}O_7$
$C_{27}H_{24}O_7$
$C_{28}H_{26}O_7$
$C_{29}H_{28}O_7$
$C_{30}H_{30}O_7$
$C_{31}H_{32}O_7$
$C_{32}H_{34}O_7$
$C_{24}H_{16}O_7$
$C_{25}H_{18}O_7$
$C_{26}H_{20}O_7$
$C_{27}H_{22}O_7$
$C_{28}H_{24}O_7$
$C_{29}H_{26}O_7$
$C_{22}H_{12}O_7$
$C_{30}H_{28}O_7$
$C_{31}H_{30}O_7$
$C_{32}H_{32}O_7$
$C_{24}H_{14}O_7$
$C_{26}H_{18}O_7$

$C_{27}H_{20}O_7$
$C_{28}H_{22}O_7$
$C_{29}H_{24}O_7$
$C_{30}H_{26}O_7$
$C_{22}H_{10}O_7$
$C_{25}H_{16}O_7$
$C_{24}H_{12}O_7$
$C_{27}H_{18}O_7$
$C_{25}H_{12}O_7$
$C_{27}H_{16}O_7$
$C_{28}H_{18}O_7$
$C_{29}H_{20}O_7$
$C_{26}H_{12}O_7$
$C_{27}H_{14}O_7$
$C_{29}H_{18}O_7$
$C_{27}H_{12}O_7$
$C_{27}H_{10}O_7$
$C_{29}H_{14}O_7$
$C_{31}H_{16}O_7$
$C_{32}H_{16}O_7$
$C_{34}H_{20}O_7$
$C_{35}H_{22}O_7$
$C_{36}H_{24}O_7$
$C_{35}H_{20}O_7$
$C_{18}H_{38}O_7S$
$C_{10}H_{20}O_7S$
$C_{16}H_{32}O_7S$
$C_{18}H_{36}O_7S$
$C_{20}H_{40}O_7S$
$C_6H_{12}O_7S$
$C_7H_{14}O_7S$
$C_8H_{16}O_7S$
$C_9H_{18}O_7S$
$C_{10}H_{18}O_7S$
$C_{11}H_{20}O_7S$
$C_{12}H_{22}O_7S$
$C_{14}H_{26}O_7S$
$C_{15}H_{28}O_7S$
$C_{16}H_{30}O_7S$
$C_{18}H_{34}O_7S$
$C_{20}H_{38}O_7S$
$C_{21}H_{40}O_7S$
$C_{22}H_{42}O_7S$
$C_6H_{10}O_7S$

$C_7H_{12}O_7S$
$C_8H_{14}O_7S$
$C_9H_{16}O_7S$
$C_9H_{14}O_7S$
$C_{10}H_{16}O_7S$
$C_{11}H_{18}O_7S$
$C_{12}H_{20}O_7S$
$C_{13}H_{22}O_7S$
$C_{14}H_{24}O_7S$
$C_{15}H_{26}O_7S$
$C_{16}H_{28}O_7S$
$C_{18}H_{32}O_7S$
$C_{20}H_{36}O_7S$
$C_{22}H_{40}O_7S$
$C_7H_{10}O_7S$
$C_8H_{12}O_7S$
$C_{10}H_{14}O_7S$
$C_{11}H_{16}O_7S$
$C_{12}H_{18}O_7S$
$C_{13}H_{20}O_7S$
$C_{14}H_{22}O_7S$
$C_{15}H_{24}O_7S$
$C_{16}H_{26}O_7S$
$C_{17}H_{28}O_7S$
$C_{18}H_{30}O_7S$
$C_{20}H_{34}O_7S$
$C_{24}H_{42}O_7S$
$C_{26}H_{46}O_7S$
$C_{27}H_{48}O_7S$
$C_{28}H_{50}O_7S$
$C_8H_{10}O_7S$
$C_9H_{12}O_7S$
$C_{11}H_{14}O_7S$
$C_{12}H_{16}O_7S$
$C_{13}H_{18}O_7S$
$C_{14}H_{20}O_7S$
$C_{15}H_{22}O_7S$
$C_{16}H_{24}O_7S$
$C_{17}H_{26}O_7S$
$C_{18}H_{28}O_7S$
$C_{24}H_{40}O_7S$
$C_{26}H_{44}O_7S$
$C_{28}H_{48}O_7S$
$C_9H_{10}O_7S$

$C_{10}H_{10}O_7S$
$C_{11}H_{12}O_7S$
$C_{12}H_{14}O_7S$
$C_{13}H_{16}O_7S$
$C_{14}H_{18}O_7S$
$C_{15}H_{20}O_7S$
$C_{16}H_{22}O_7S$
$C_{17}H_{24}O_7S$
$C_{18}H_{26}O_7S$
$C_{26}H_{42}O_7S$
$C_{30}H_{48}O_7S$
$C_{31}H_{50}O_7S$
$C_{32}H_{52}O_7S$
$C_{33}H_{54}O_7S$
$C_{11}H_{10}O_7S$
$C_{12}H_{12}O_7S$
$C_{13}H_{14}O_7S$
$C_{14}H_{16}O_7S$
$C_{15}H_{18}O_7S$
$C_{16}H_{20}O_7S$
$C_{17}H_{22}O_7S$
$C_{18}H_{24}O_7S$
$C_{19}H_{26}O_7S$
$C_{31}H_{48}O_7S$
$C_{13}H_{12}O_7S$
$C_{14}H_{14}O_7S$
$C_{15}H_{16}O_7S$
$C_{16}H_{18}O_7S$
$C_{17}H_{20}O_7S$
$C_{18}H_{22}O_7S$
$C_{19}H_{24}O_7S$
$C_{13}H_{10}O_7S$
$C_{14}H_{12}O_7S$
$C_{15}H_{14}O_7S$
$C_{16}H_{16}O_7S$
$C_{17}H_{18}O_7S$
$C_{18}H_{20}O_7S$
$C_{19}H_{22}O_7S$
$C_{20}H_{24}O_7S$
$C_{16}H_{14}O_7S$
$C_{17}H_{16}O_7S$
$C_{18}H_{18}O_7S$
$C_{19}H_{20}O_7S$
$C_{17}H_{14}O_7S$

C18H16O7S
C19H18O7S
C20H18O7S
C23H20O7S
C21H14O7S
C23H18O7S
C11H24O7S3
C12H26O7S3
C14H30O7S3
C15H32O7S3
C17H36O7S3
C18H38O7S3
C19H40O7S3
C10H22O7S3
C13H28O7S3
C16H34O7S3
C23H48O7S3
C25H52O7S3
C9H20O7S3
C13H26O7S3
C14H28O7S3
C15H30O7S3
C16H32O7S3
C18H36O7S3
C24H48O7S3
C10H20O7S3
C11H22O7S3
C12H24O7S3
C17H34O7S3
C19H38O7S3
C23H46O7S3
C9H18O7S3
C14H26O7S3
C15H28O7S3
C16H30O7S3
C17H32O7S3
C21H40O7S3
C25H48O7S3
C10H18O7S3
C11H20O7S3
C12H22O7S3
C13H24O7S3
C23H44O7S3
C16H28O7S3

$C_{18}H_{32}O_7S_3$
$C_{21}H_{38}O_7S_3$
$C_{22}H_{40}O_7S_3$
$C_{23}H_{42}O_7S_3$
$C_{13}H_{22}O_7S_3$
$C_{14}H_{24}O_7S_3$
$C_{15}H_{26}O_7S_3$
$C_{12}H_{18}O_7S_3$
$C_{15}H_{24}O_7S_3$
$C_{19}H_{28}O_7S_3$
$C_{22}H_{26}O_7S_3$
$C_{23}H_{28}O_7S_3$
$C_{25}H_{32}O_7S_3$
$C_{26}H_{34}O_7S_3$
$C_{23}H_{26}O_7S_3$
$C_{25}H_{30}O_7S_3$
$C_{26}H_{32}O_7S_3$
$C_{24}H_{28}O_7S_3$
$C_{29}H_{38}O_7S_3$
$C_{20}H_{18}O_7S_3$
$C_{22}H_{22}O_7S_3$
$C_{23}H_{24}O_7S_3$
$C_{24}H_{26}O_7S_3$
$C_{25}H_{28}O_7S_3$
$C_{27}H_{30}O_7S_3$
$C_{19}H_{14}O_7S_3$
$C_{21}H_{18}O_7S_3$
$C_{22}H_{20}O_7S_3$
$C_{23}H_{22}O_7S_3$
$C_{24}H_{24}O_7S_3$
$C_{25}H_{26}O_7S_3$
$C_{26}H_{28}O_7S_3$
$C_{30}H_{36}O_7S_3$
$C_{31}H_{38}O_7S_3$
$C_{22}H_{18}O_7S_3$
$C_{24}H_{22}O_7S_3$
$C_{24}H_{18}O_7S_3$
$C_{43}H_{56}O_7S_3$
$C_{13}H_{28}O_8$
$C_{14}H_{30}O_8$
$C_{15}H_{32}O_8$
$C_{16}H_{34}O_8$
$C_{17}H_{36}O_8$
$C_{18}H_{38}O_8$

$C_{19}H_{40}O_8$
$C_{12}H_{24}O_8$
$C_{13}H_{26}O_8$
$C_{14}H_{28}O_8$
$C_{15}H_{30}O_8$
$C_{16}H_{32}O_8$
$C_{17}H_{34}O_8$
$C_{18}H_{36}O_8$
$C_{19}H_{38}O_8$
$C_{20}H_{40}O_8$
$C_{21}H_{42}O_8$
$C_{22}H_{44}O_8$
$C_{24}H_{48}O_8$
$C_{11}H_{20}O_8$
$C_{12}H_{22}O_8$
$C_{13}H_{24}O_8$
$C_{14}H_{26}O_8$
$C_{15}H_{28}O_8$
$C_{17}H_{32}O_8$
$C_{18}H_{34}O_8$
$C_{19}H_{36}O_8$
$C_{20}H_{38}O_8$
$C_{21}H_{40}O_8$
$C_{22}H_{42}O_8$
$C_{23}H_{44}O_8$
$C_{25}H_{48}O_8$
$C_{26}H_{50}O_8$
$C_{27}H_{52}O_8$
$C_{10}H_{18}O_8$
$C_9H_{16}O_8$
$C_{11}H_{18}O_8$
$C_{12}H_{20}O_8$
$C_{13}H_{22}O_8$
$C_{14}H_{24}O_8$
$C_{15}H_{26}O_8$
$C_{16}H_{28}O_8$
$C_{17}H_{30}O_8$
$C_{18}H_{32}O_8$
$C_{21}H_{38}O_8$
$C_{22}H_{40}O_8$
$C_{23}H_{42}O_8$
$C_{24}H_{44}O_8$
$C_{25}H_{46}O_8$
$C_{26}H_{48}O_8$

$C_{27}H_{50}O_8$
$C_{28}H_{52}O_8$
$C_{10}H_{16}O_8$
$C_{19}H_{34}O_8$
$C_{20}H_{36}O_8$
$C_9H_{14}O_8$
$C_{12}H_{18}O_8$
$C_{13}H_{20}O_8$
$C_{14}H_{22}O_8$
$C_{15}H_{24}O_8$
$C_{16}H_{26}O_8$
$C_{17}H_{28}O_8$
$C_{18}H_{30}O_8$
$C_{24}H_{42}O_8$
$C_{25}H_{44}O_8$
$C_{28}H_{50}O_8$
$C_{29}H_{52}O_8$
$C_{30}H_{54}O_8$
$C_{10}H_{14}O_8$
$C_{11}H_{16}O_8$
$C_{19}H_{32}O_8$
$C_{20}H_{34}O_8$
$C_{21}H_{36}O_8$
$C_{22}H_{38}O_8$
$C_{23}H_{40}O_8$
$C_{26}H_{46}O_8$
$C_{27}H_{48}O_8$
$C_{10}H_{12}O_8$
$C_{11}H_{14}O_8$
$C_{12}H_{16}O_8$
$C_{13}H_{18}O_8$
$C_{14}H_{20}O_8$
$C_{15}H_{22}O_8$
$C_{16}H_{24}O_8$
$C_{17}H_{26}O_8$
$C_{18}H_{28}O_8$
$C_{19}H_{30}O_8$
$C_{20}H_{32}O_8$
$C_{21}H_{34}O_8$
$C_{22}H_{36}O_8$
$C_{23}H_{38}O_8$
$C_{24}H_{40}O_8$
$C_{25}H_{42}O_8$
$C_{28}H_{48}O_8$

$C_{29}H_{50}O_8$
$C_{30}H_{52}O_8$
$C_9H_{10}O_8$
$C_{26}H_{44}O_8$
$C_{27}H_{46}O_8$
$C_{15}H_{20}O_8$
$C_{17}H_{24}O_8$
$C_{18}H_{26}O_8$
$C_{19}H_{28}O_8$
$C_{20}H_{30}O_8$
$C_{21}H_{32}O_8$
$C_{22}H_{34}O_8$
$C_{23}H_{36}O_8$
$C_{24}H_{38}O_8$
$C_{25}H_{40}O_8$
$C_{26}H_{42}O_8$
$C_{27}H_{44}O_8$
$C_{28}H_{46}O_8$
$C_{29}H_{48}O_8$
$C_{30}H_{50}O_8$
$C_{31}H_{52}O_8$
$C_{32}H_{54}O_8$
$C_{33}H_{56}O_8$
$C_{36}H_{62}O_8$
$C_{10}H_{10}O_8$
$C_{11}H_{12}O_8$
$C_{12}H_{14}O_8$
$C_{13}H_{16}O_8$
$C_{14}H_{18}O_8$
$C_{16}H_{22}O_8$
$C_{16}H_{20}O_8$
$C_{17}H_{22}O_8$
$C_{18}H_{24}O_8$
$C_{19}H_{26}O_8$
$C_{20}H_{28}O_8$
$C_{21}H_{30}O_8$
$C_{22}H_{32}O_8$
$C_{23}H_{34}O_8$
$C_{24}H_{36}O_8$
$C_{25}H_{38}O_8$
$C_{26}H_{40}O_8$
$C_{27}H_{42}O_8$
$C_{28}H_{44}O_8$
$C_{31}H_{50}O_8$

$C_{32}H_{52}O_8$
$C_{33}H_{54}O_8$
$C_{35}H_{58}O_8$
$C_{36}H_{60}O_8$
$C_{37}H_{62}O_8$
$C_{11}H_{10}O_8$
$C_{12}H_{12}O_8$
$C_{13}H_{14}O_8$
$C_{14}H_{16}O_8$
$C_{15}H_{18}O_8$
$C_{16}H_{18}O_8$
$C_{17}H_{20}O_8$
$C_{18}H_{22}O_8$
$C_{19}H_{24}O_8$
$C_{20}H_{26}O_8$
$C_{21}H_{28}O_8$
$C_{22}H_{30}O_8$
$C_{23}H_{32}O_8$
$C_{25}H_{36}O_8$
$C_{26}H_{38}O_8$
$C_{12}H_{10}O_8$
$C_{13}H_{12}O_8$
$C_{14}H_{14}O_8$
$C_{15}H_{16}O_8$
$C_{24}H_{34}O_8$
$C_{27}H_{40}O_8$
$C_{28}H_{42}O_8$
$C_{29}H_{44}O_8$
$C_{18}H_{20}O_8$
$C_{19}H_{22}O_8$
$C_{20}H_{24}O_8$
$C_{21}H_{26}O_8$
$C_{22}H_{28}O_8$
$C_{23}H_{30}O_8$
$C_{13}H_{10}O_8$
$C_{14}H_{12}O_8$
$C_{15}H_{14}O_8$
$C_{16}H_{16}O_8$
$C_{17}H_{18}O_8$
$C_{25}H_{34}O_8$
$C_{27}H_{38}O_8$
$C_{20}H_{22}O_8$
$C_{21}H_{24}O_8$
$C_{14}H_{10}O_8$

C15H12O8
C16H14O8
C17H16O8
C18H18O8
C19H20O8
C22H26O8
C23H28O8
C24H30O8
C25H32O8
C15H10O8
C16H12O8
C17H14O8
C18H16O8
C19H18O8
C20H20O8
C21H22O8
C22H24O8
C23H26O8
C24H28O8
C25H30O8
C16H10O8
C17H12O8
C18H14O8
C19H16O8
C20H18O8
C21H20O8
C22H22O8
C23H24O8
C24H26O8
C25H28O8
C20H16O8
C22H20O8
C24H24O8
C18H12O8
C19H14O8
C21H18O8
C23H22O8
C25H26O8
C26H28O8
C20H14O8
C21H16O8
C22H18O8
C23H20O8
C24H22O8

$C_{25}H_{24}O_8$
$C_{25}H_{22}O_8$
$C_{22}H_{16}O_8$
$C_{23}H_{18}O_8$
$C_{24}H_{20}O_8$
$C_{25}H_{20}O_8$
$C_{26}H_{22}O_8$
$C_{29}H_{28}O_8$
$C_{24}H_{18}O_8$
$C_{27}H_{24}O_8$
$C_{28}H_{24}O_8$
$C_{24}H_{16}O_8$
$C_{25}H_{18}O_8$
$C_{27}H_{22}O_8$
$C_{32}H_{32}O_8$
$C_{24}H_{14}O_8$
$C_{27}H_{20}O_8$
$C_{28}H_{22}O_8$
$C_{25}H_{14}O_8$
$C_{26}H_{16}O_8$
$C_{27}H_{18}O_8$
$C_{28}H_{18}O_8$
$C_{25}H_{12}O_8$
$C_{27}H_{16}O_8$
$C_{25}H_{10}O_8$
$C_{26}H_{12}O_8$
$C_{27}H_{14}O_8$
$C_{28}H_{16}O_8$
$C_{29}H_{18}O_8$
$C_{30}H_{20}O_8$
$C_{28}H_{14}O_8$
$C_{29}H_{16}O_8$
$C_{27}H_{10}O_8$
$C_{29}H_{14}O_8$
$C_{31}H_{18}O_8$
$C_{29}H_{12}O_8$
$C_{33}H_{16}O_8$
$C_{34}H_{18}O_8$
$C_{42}H_{18}O_8$
$C_{20}H_{42}O_8S$
$C_{13}H_{26}O_8S$
$C_{18}H_{36}O_8S$
$C_{19}H_{38}O_8S$
$C_{20}H_{40}O_8S$

$C_{22}H_{44}O_8S$
$C_{10}H_{18}O_8S$
$C_{11}H_{20}O_8S$
$C_{12}H_{22}O_8S$
$C_{13}H_{24}O_8S$
$C_{14}H_{26}O_8S$
$C_{15}H_{28}O_8S$
$C_{16}H_{30}O_8S$
$C_{18}H_{34}O_8S$
$C_{19}H_{36}O_8S$
$C_{20}H_{38}O_8S$
$C_{21}H_{40}O_8S$
$C_{22}H_{42}O_8S$
$C_7H_{12}O_8S$
$C_8H_{14}O_8S$
$C_9H_{16}O_8S$
$C_{10}H_{16}O_8S$
$C_{11}H_{18}O_8S$
$C_{12}H_{20}O_8S$
$C_{13}H_{22}O_8S$
$C_{14}H_{24}O_8S$
$C_{15}H_{26}O_8S$
$C_{16}H_{28}O_8S$
$C_{18}H_{32}O_8S$
$C_{20}H_{36}O_8S$
$C_{22}H_{40}O_8S$
$C_8H_{12}O_8S$
$C_9H_{14}O_8S$
$C_{10}H_{14}O_8S$
$C_{11}H_{16}O_8S$
$C_{12}H_{18}O_8S$
$C_{13}H_{20}O_8S$
$C_{14}H_{22}O_8S$
$C_{15}H_{24}O_8S$
$C_{16}H_{26}O_8S$
$C_{18}H_{30}O_8S$
$C_{20}H_{34}O_8S$
$C_{22}H_{38}O_8S$
$C_{26}H_{46}O_8S$
$C_9H_{12}O_8S$
$C_{10}H_{12}O_8S$
$C_{11}H_{14}O_8S$
$C_{12}H_{16}O_8S$
$C_{13}H_{18}O_8S$

$C_{14}H_{20}O_8S$
$C_{15}H_{22}O_8S$
$C_{16}H_{24}O_8S$
$C_{17}H_{26}O_8S$
$C_{11}H_{12}O_8S$
$C_{12}H_{14}O_8S$
$C_{13}H_{16}O_8S$
$C_{14}H_{18}O_8S$
$C_{15}H_{20}O_8S$
$C_{16}H_{22}O_8S$
$C_{17}H_{24}O_8S$
$C_{24}H_{38}O_8S$
$C_{11}H_{10}O_8S$
$C_{12}H_{12}O_8S$
$C_{13}H_{14}O_8S$
$C_{14}H_{16}O_8S$
$C_{15}H_{18}O_8S$
$C_{16}H_{20}O_8S$
$C_{17}H_{22}O_8S$
$C_{18}H_{24}O_8S$
$C_{19}H_{26}O_8S$
$C_{13}H_{12}O_8S$
$C_{14}H_{14}O_8S$
$C_{15}H_{16}O_8S$
$C_{16}H_{18}O_8S$
$C_{17}H_{20}O_8S$
$C_{18}H_{22}O_8S$
$C_{19}H_{24}O_8S$
$C_{14}H_{12}O_8S$
$C_{15}H_{14}O_8S$
$C_{16}H_{16}O_8S$
$C_{17}H_{18}O_8S$
$C_{18}H_{20}O_8S$
$C_{19}H_{22}O_8S$
$C_{15}H_{12}O_8S$
$C_{16}H_{14}O_8S$
$C_{17}H_{16}O_8S$
$C_{18}H_{18}O_8S$
$C_{19}H_{20}O_8S$
$C_{20}H_{22}O_8S$
$C_{21}H_{24}O_8S$
$C_{17}H_{14}O_8S$
$C_{20}H_{20}O_8S$
$C_{21}H_{20}O_8S$

C27H16O8S
C14H30O8S3
C16H34O8S3
C17H36O8S3
C19H40O8S3
C11H24O8S3
C12H26O8S3
C13H28O8S3
C15H32O8S3
C23H48O8S3
C25H52O8S3
C28H58O8S3
C13H26O8S3
C14H28O8S3
C17H34O8S3
C10H20O8S3
C11H22O8S3
C12H24O8S3
C15H30O8S3
C16H32O8S3
C18H36O8S3
C19H38O8S3
C20H40O8S3
C23H46O8S3
C24H48O8S3
C25H50O8S3
C17H32O8S3
C18H34O8S3
C12H22O8S3
C13H24O8S3
C14H26O8S3
C15H28O8S3
C16H30O8S3
C19H36O8S3
C17H30O8S3
C20H36O8S3
C15H26O8S3
C17H28O8S3
C16H26O8S3
C22H24O8S3
C25H30O8S3
C23H24O8S3
C21H18O8S3
C25H26O8S3

$C_{27}H_{30}O_8S_3$
$C_{31}H_{18}O_8S_3$
$C_{16}H_{34}O_9$
$C_{17}H_{36}O_9$
$C_{18}H_{38}O_9$
$C_{19}H_{40}O_9$
$C_{20}H_{42}O_9$
$C_{21}H_{44}O_9$
$C_{12}H_{24}O_9$
$C_{14}H_{28}O_9$
$C_{15}H_{30}O_9$
$C_{16}H_{32}O_9$
$C_{17}H_{34}O_9$
$C_{18}H_{36}O_9$
$C_{19}H_{38}O_9$
$C_{20}H_{40}O_9$
$C_{21}H_{42}O_9$
$C_{22}H_{44}O_9$
$C_9H_{18}O_9$
$C_{13}H_{24}O_9$
$C_{14}H_{26}O_9$
$C_{15}H_{28}O_9$
$C_{16}H_{30}O_9$
$C_{17}H_{32}O_9$
$C_{18}H_{34}O_9$
$C_{19}H_{36}O_9$
$C_{20}H_{38}O_9$
$C_{21}H_{40}O_9$
$C_{22}H_{42}O_9$
$C_{23}H_{44}O_9$
$C_{25}H_{48}O_9$
$C_{26}H_{50}O_9$
$C_{14}H_{24}O_9$
$C_{15}H_{26}O_9$
$C_{16}H_{28}O_9$
$C_{17}H_{30}O_9$
$C_{18}H_{32}O_9$
$C_{19}H_{34}O_9$
$C_{21}H_{38}O_9$
$C_{22}H_{40}O_9$
$C_{23}H_{42}O_9$
$C_{26}H_{48}O_9$
$C_{10}H_{14}O_9$
$C_{11}H_{16}O_9$

$C_{12}H_{18}O_9$
$C_{13}H_{20}O_9$
$C_{14}H_{22}O_9$
$C_{15}H_{24}O_9$
$C_{16}H_{26}O_9$
$C_{17}H_{28}O_9$
$C_{18}H_{30}O_9$
$C_{19}H_{32}O_9$
$C_{20}H_{34}O_9$
$C_{21}H_{36}O_9$
$C_{22}H_{38}O_9$
$C_{23}H_{40}O_9$
$C_{25}H_{44}O_9$
$C_{29}H_{52}O_9$
$C_{30}H_{54}O_9$
$C_9H_{12}O_9$
$C_{24}H_{42}O_9$
$C_{26}H_{46}O_9$
$C_{27}H_{48}O_9$
$C_{14}H_{20}O_9$
$C_{15}H_{22}O_9$
$C_{16}H_{24}O_9$
$C_{17}H_{26}O_9$
$C_{18}H_{28}O_9$
$C_{19}H_{30}O_9$
$C_{20}H_{32}O_9$
$C_{21}H_{34}O_9$
$C_{22}H_{36}O_9$
$C_{24}H_{40}O_9$
$C_{25}H_{42}O_9$
$C_{27}H_{46}O_9$
$C_{28}H_{48}O_9$
$C_{29}H_{50}O_9$
$C_{11}H_{14}O_9$
$C_{12}H_{16}O_9$
$C_{13}H_{18}O_9$
$C_{23}H_{38}O_9$
$C_{26}H_{44}O_9$
$C_{11}H_{12}O_9$
$C_{12}H_{14}O_9$
$C_{13}H_{16}O_9$
$C_{14}H_{18}O_9$
$C_{15}H_{20}O_9$
$C_{18}H_{26}O_9$

C19H28O9
C20H30O9
C21H32O9
C22H34O9
C23H36O9
C24H38O9
C25H40O9
C26H42O9
C27H44O9
C28H46O9
C29H48O9
C32H54O9
C35H60O9
C16H22O9
C17H24O9
C30H50O9
C18H24O9
C19H26O9
C20H28O9
C21H30O9
C22H32O9
C23H34O9
C24H36O9
C27H42O9
C28H44O9
C29H46O9
C30H48O9
C32H52O9
C35H58O9
C12H12O9
C13H14O9
C14H16O9
C15H18O9
C16H20O9
C17H22O9
C25H38O9
C26H40O9
C18H22O9
C19H24O9
C20H26O9
C21H28O9
C22H30O9
C23H32O9
C24H34O9

C25H36O9
C26H38O9
C12H10O9
C13H12O9
C14H14O9
C15H16O9
C16H18O9
C17H20O9
C21H26O9
C14H12O9
C15H14O9
C16H16O9
C17H18O9
C18H20O9
C19H22O9
C20H24O9
C22H28O9
C24H32O9
C14H10O9
C15H12O9
C16H14O9
C17H16O9
C18H18O9
C19H20O9
C20H22O9
C21H24O9
C22H26O9
C23H28O9
C24H30O9
C25H32O9
C27H36O9
C15H10O9
C16H12O9
C17H14O9
C18H16O9
C19H18O9
C20H20O9
C21H22O9
C22H24O9
C23H26O9
C24H28O9
C25H30O9
C26H32O9
C17H12O9

$C_{18}H_{14}O_9$
$C_{19}H_{16}O_9$
$C_{20}H_{18}O_9$
$C_{21}H_{20}O_9$
$C_{22}H_{22}O_9$
$C_{23}H_{24}O_9$
$C_{24}H_{26}O_9$
$C_{25}H_{28}O_9$
$C_{26}H_{30}O_9$
$C_{18}H_{12}O_9$
$C_{19}H_{14}O_9$
$C_{20}H_{16}O_9$
$C_{21}H_{18}O_9$
$C_{22}H_{20}O_9$
$C_{23}H_{22}O_9$
$C_{24}H_{24}O_9$
$C_{25}H_{26}O_9$
$C_{26}H_{28}O_9$
$C_{20}H_{14}O_9$
$C_{21}H_{16}O_9$
$C_{22}H_{18}O_9$
$C_{23}H_{20}O_9$
$C_{24}H_{22}O_9$
$C_{25}H_{24}O_9$
$C_{26}H_{26}O_9$
$C_{27}H_{28}O_9$
$C_{22}H_{16}O_9$
$C_{23}H_{18}O_9$
$C_{24}H_{20}O_9$
$C_{25}H_{22}O_9$
$C_{26}H_{24}O_9$
$C_{26}H_{22}O_9$
$C_{27}H_{24}O_9$
$C_{31}H_{12}O_9$
$C_{42}H_{12}O_9$
$C_{22}H_{46}O_9S$
$C_{22}H_{44}O_9S$
$C_{18}H_{36}O_9S$
$C_8H_{16}O_9S$
$C_{23}H_{44}O_9S$
$C_{14}H_{26}O_9S$
$C_{18}H_{34}O_9S$
$C_{20}H_{38}O_9S$
$C_{22}H_{42}O_9S$

$C_{23}H_{42}O_9S$
$C_{12}H_{20}O_9S$
$C_{13}H_{22}O_9S$
$C_{14}H_{24}O_9S$
$C_{18}H_{32}O_9S$
$C_{20}H_{36}O_9S$
$C_{21}H_{38}O_9S$
$C_{22}H_{40}O_9S$
$C_{11}H_{16}O_9S$
$C_{12}H_{18}O_9S$
$C_{13}H_{20}O_9S$
$C_{18}H_{30}O_9S$
$C_{10}H_{12}O_9S$
$C_{11}H_{14}O_9S$
$C_{12}H_{16}O_9S$
$C_{13}H_{18}O_9S$
$C_{14}H_{20}O_9S$
$C_{15}H_{22}O_9S$
$C_{16}H_{24}O_9S$
$C_{17}H_{26}O_9S$
$C_{18}H_{28}O_9S$
$C_{12}H_{14}O_9S$
$C_{13}H_{16}O_9S$
$C_{14}H_{18}O_9S$
$C_{15}H_{20}O_9S$
$C_{16}H_{22}O_9S$
$C_{17}H_{24}O_9S$
$C_{18}H_{26}O_9S$
$C_{36}H_{60}O_9S$
$C_{12}H_{12}O_9S$
$C_{13}H_{14}O_9S$
$C_{14}H_{16}O_9S$
$C_{15}H_{18}O_9S$
$C_{16}H_{20}O_9S$
$C_{17}H_{22}O_9S$
$C_{18}H_{24}O_9S$
$C_{19}H_{26}O_9S$
$C_{20}H_{28}O_9S$
$C_{15}H_{16}O_9S$
$C_{16}H_{18}O_9S$
$C_{17}H_{20}O_9S$
$C_{18}H_{22}O_9S$
$C_{19}H_{24}O_9S$
$C_{15}H_{14}O_9S$

$C_{17}H_{18}O_9S$
$C_{18}H_{20}O_9S$
$C_{19}H_{22}O_9S$
$C_{20}H_{24}O_9S$
$C_{22}H_{28}O_9S$
$C_{19}H_{20}O_9S$
$C_{20}H_{22}O_9S$
$C_{21}H_{24}O_9S$
$C_{22}H_{26}O_9S$
$C_{18}H_{16}O_9S$
$C_{19}H_{18}O_9S$
$C_{21}H_{22}O_9S$
$C_{22}H_{24}O_9S$
$C_{23}H_{12}O_9S$
$C_{17}H_{36}O_9S_3$
$C_{11}H_{24}O_9S_3$
$C_{12}H_{26}O_9S_3$
$C_{13}H_{28}O_9S_3$
$C_{14}H_{30}O_9S_3$
$C_{16}H_{34}O_9S_3$
$C_{18}H_{36}O_9S_3$
$C_{25}H_{50}O_9S_3$
$C_{14}H_{26}O_9S_3$
$C_{15}H_{28}O_9S_3$
$C_{17}H_{32}O_9S_3$
$C_{23}H_{44}O_9S_3$
$C_{16}H_{28}O_9S_3$
$C_{18}H_{32}O_9S_3$
$C_{25}H_{28}O_9S_3$
$C_{26}H_{30}O_9S_3$
$C_{12}H_{22}OS$
$C_{13}H_{22}OS$
$C_{25}H_{44}OS$
$C_{15}H_{22}OS$
$C_{17}H_{26}OS$
$C_{17}H_{24}OS$
$C_{18}H_{26}OS$
$C_{18}H_{24}OS$
$C_{19}H_{24}OS$
$C_{15}H_{16}OS$
$C_{17}H_{20}OS$
$C_{18}H_{22}OS$
$C_{14}H_{12}OS$
$C_{14}H_{10}OS$

$C_{37}H_{54}OS$
$C_{24}H_{28}OS$
$C_{33}H_{44}OS$
$C_{21}H_{20}OS$
$C_{23}H_{20}OS$
$C_{25}H_{24}OS$
$C_{28}H_{30}OS$
$C_{20}H_{12}OS$
$C_{16}H_{32}OS_2$
$C_{10}H_{18}OS_2$
$C_{18}H_{32}OS_2$
$C_{20}H_{36}OS_2$
$C_{19}H_{32}OS_2$
$C_{12}H_{12}OS_2$
$C_{13}H_{14}OS_2$
$C_{15}H_{16}OS_2$
$C_{16}H_{16}OS_2$
$C_{17}H_{18}OS_2$
$C_{18}H_{20}OS_2$
$C_{19}H_{22}OS_2$
$C_{20}H_{24}OS_2$
$C_{25}H_{34}OS_2$
$C_{27}H_{38}OS_2$
$C_{29}H_{42}OS_2$
$C_{24}H_{32}OS_2$
$C_{18}H_{18}OS_2$
$C_{19}H_{20}OS_2$
$C_{20}H_{22}OS_2$
$C_{20}H_{18}OS_2$
$C_{22}H_{22}OS_2$
$C_{24}H_{26}OS_2$
$C_{34}H_{46}OS_2$
$C_{18}H_{14}OS_2$
$C_{21}H_{20}OS_2$
$C_{35}H_{26}OS_2$
$C_{36}H_{28}OS_2$
$C_{34}H_{22}OS_2$
$C_{30}H_{12}OS_2$
$C_{32}H_{16}OS_2$
$C_{34}H_{20}OS_2$
$C_{31}H_{12}OS_2$
$C_{32}H_{14}OS_2$
$C_{33}H_{16}OS_2$
$C_{34}H_{18}OS_2$

C33H14OS2
C34H12OS2
C44H16OS2
C9H14OS3
C21H30OS3
C17H14OS3
C19H18OS3
C25H16OS3
C33H18OS3
C35H22OS3
C37H26OS3
C32H16OS3
C34H20OS3
C36H24OS3
C39H30OS3
C32H14OS3
C33H16OS3
C39H22OS3
C40H20N2O10S2
C17H14N2O11
C20H20N2O11
C20H12N2O11
C44H48N2O11
C26H16N2O11S3
C24H20N2O12
C25H18N2O12
C20H40N2O12S
C19H32N2O12S
C23H16N2O12S
C31H16N2O12S
C35H16N2O12S
C14H26N2O12S2
C14H12N2O12S2
C21H16N2O12S2
C29H14N2O12S2
C31H12N2O12S2
C30H12N2O13S
C14H12N2O14
C37H40N2O14
C26H12N2O14
C23H24N2O14S
C25H14N2O14S
C37H22N2O14S
C28H16N2O14S2

C29H12N2O14S2
C24H18N2O14S3
C24H44N2O15
C38H56N2O15
C20H16N2O15S
C23H22N2O15S
C28H14N2O15S
C18H14N2O15S2
C15H18N2O15S3
C23H16N2O15S3
C20H16N2O16
C28H18N2O16
C31H24N2O16
C17H14N2O16S
C19H12N2O16S
C21H16N2O16S
C22H18N2O16S
C17H12N2O16S2
C20H18N2O16S2
C17H10N2O16S2
C17H38N2O17S
C17H12N2O17S
C23H20N2O17S
C15H20N2O17S2
C15H18N2O18
C17H12N2O18
C20H18N2O18
C21H22N2O18S2
C21H20N2O18S2
C29H44N2O19
C25H16N2O19
C28H22N2O19
C35H14N2O19
C20H22N2O19S
C18H18N2O19S3
C17H24N2O20
C20H30N2O20
C20H22N2O20
C24H12N2O20
C20H20N2O21S
C23H14N2O22
C20H34N2O6S3
C22H28N2O6S3
C19H12N2O6S3

$C_{19}H_{14}N_2O_9S_2$
$C_{23}H_{18}N_2O_9S_2$
$C_{26}H_{24}N_2O_9S_2$
$C_{22}H_{40}N_2O_9S_3$
$C_{24}H_{16}N_2O_9S_3$
$C_{32}H_{18}N_2O_9S_3$
$C_{10}H_{17}N_3O_{10}S_3$
$C_{21}H_{37}N_3O_{11}S_2$
$C_{29}H_{17}N_3O_{12}S$
$C_{15}H_{27}N_3O_{12}S_2$
$C_{11}H_{17}N_3O_{13}S_2$
$C_{22}H_{29}N_3O_{21}$
$C_9H_{17}N_3O_7S_3$
$C_{20}H_{37}N_3O_7S_3$
$C_{17}H_{27}N_3O_7S_3$
$C_{14}H_{13}N_3O_7S_3$
$C_{17}H_{29}NO_{12}S_2$
$C_{23}H_{15}NO_{12}S_2$
$C_{31}H_{17}NO_{13}S_2$
$C_{18}H_{19}NO_{14}S_2$
$C_{15}H_{21}NO_{14}S_3$
$C_{16}H_{17}NO_{14}S_3$
$C_{23}H_{21}NO_{19}$
$C_{18}H_{15}NO_8S_2$
$C_{27}H_{17}NO_8S_2$
$C_9H_{17}NO_9S$
$C_{23}H_{23}NO_9S$
$C_{38}H_{29}NO_9S$
$C_{34}H_{45}NO_9S_2$
$C_{10}H_{12}O_{10}S_2$
$C_{25}H_{50}O_{10}S_3$
$C_{19}H_{16}O_{10}S_3$
$C_{19}H_{22}O_{11}S_3$
$C_{22}H_{46}O_{12}S_3$
$C_{14}H_{28}O_{12}S_3$
$C_{33}H_{14}O_{12}S_3$
$C_{11}H_{20}O_{13}S_2$
$C_{14}H_{18}O_{13}S_3$
$C_{20}H_{20}O_{13}S_3$
$C_{15}H_{28}O_{14}$
$C_{30}H_{34}O_{14}$
$C_{25}H_{14}O_{14}$
$C_{34}H_{12}O_{14}$
$C_{22}H_{14}O_{14}S_2$

$C_{22}H_{16}O_{15}$
$C_{42}H_{16}O_{15}$
$C_{14}H_{22}O_{15}S_2$
$C_{35}H_{18}O_{15}S_3$
$C_{31}H_{18}O_{16}$
$C_{34}H_{24}O_{16}$
$C_{22}H_{12}O_{16}S$
$C_{31}H_{14}O_{16}S$
$C_{18}H_{18}O_{16}S_2$
$C_{22}H_{16}O_{16}S_3$
$C_{36}H_{44}O_{18}$
$C_{25}H_{16}O_{18}$
$C_{37}H_{24}O_{18}$
$C_{21}H_{38}O_{19}$
$C_{20}H_{18}O_{19}$
$C_{32}H_{60}O_{19}S$
$C_{18}H_{18}O_{19}S$
$C_{18}H_{16}O_{19}S$
$C_{28}H_{12}O_{19}S$
$C_{26}H_{12}O_{19}S_2$
$C_{19}H_{14}O_{20}$
$C_{26}H_{18}O_{20}$
$C_{17}H_{14}O_{20}S$
$C_{27}H_{20}O_{20}S_3$
$C_{22}H_{14}O_{24}$
$C_{20}H_{14}O_{24}S$
$C_{20}H_{12}O_{24}S$
$C_{11}H_{16}O_7S_2$
$C_{12}H_{18}O_7S_2$
$C_{15}H_{18}O_7S_2$
$C_{18}H_{18}O_7S_2$
$C_{17}H_{14}O_7S_2$
$C_{16}H_{10}O_7S_2$
$C_{17}H_{12}O_7S_2$
$C_{18}H_{14}O_7S_2$
$C_{17}H_{10}O_7S_2$
$C_{32}H_{16}O_7S_2$
$C_{11}H_{16}O_8S_2$
$C_{12}H_{18}O_8S_2$
$C_{16}H_{22}O_8S_2$
$C_{14}H_{16}O_8S_2$
$C_{10}H_{14}O_9S_2$
